# Subgroup identification in clinical trials via the predicted individual treatment effect

**DOI:** 10.1371/journal.pone.0205971

**Published:** 2018-10-18

**Authors:** Nicolás M. Ballarini, Gerd K. Rosenkranz, Thomas Jaki, Franz König, Martin Posch

**Affiliations:** 1 Section for Medical Statistics, Center for Medical Statistics, Informatics, and Intelligent Systems, Medical University of Vienna, Vienna, Austria; 2 Medical and Pharmaceutical Statistics Research Unit, Department of Mathematics and Statistics, Lancaster University, Lancaster, United Kingdom; University of California, Berkeley, UNITED STATES

## Abstract

Identifying subgroups of treatment responders through the different phases of clinical trials has the potential to increase success in drug development. Recent developments in subgroup analysis consider subgroups that are defined in terms of the predicted individual treatment effect, i.e. the difference between the predicted outcome under treatment and the predicted outcome under control for each individual, which in turn may depend on multiple biomarkers. In this work, we study the properties of different modelling strategies to estimate the predicted individual treatment effect. We explore linear models and compare different estimation methods, such as maximum likelihood and the Lasso with and without randomized response. For the latter, we implement confidence intervals based on the selective inference framework to account for the model selection stage. We illustrate the methods in a dataset of a treatment for Alzheimer disease (normal response) and in a dataset of a treatment for prostate cancer (survival outcome). We also evaluate via simulations the performance of using the predicted individual treatment effect to identify subgroups where a novel treatment leads to better outcomes compared to a control treatment.

## 1 Introduction

With the advent of personalized medicine, there has been an increasing interest in identifying baseline characteristics (biomarkers) of the subjects under study that are associated with a greater benefit of the treatment or better tolerance. Both regulatory agencies U.S. Food and Drug Administration and European Medicines Agency have recently released guidelines on the investigation of subgroups defined by such biomarkers in confirmatory trials; highlighting, among other issues, the problems of data-driven methods to identify subgroups [1, 2]. It is stated, for example, that subgroup selection when a signal of relevant efficacy is apparent in a subgroup may provide unreliable estimates of subgroup effects. Nevertheless, methods aimed at selecting the most promising subgroups in a data-driven manner may suggest trends that could be confirmed in subsequent clinical trials. Moreover, if treatment effect heterogeneity is a concern, exploratory subgroup analysis may lead to insights to regulators and payers when deciding on drug approvals or reimbursement.

It is widely accepted that subgroup analyses should rely not only on demonstrating a treatment effect in the subgroup but also on modelling the interaction effect between treatment and the biomarkers that define such subgroups [3]. In this sense, it is important to differentiate biomarkers that are prognostic, predictive or both. While **prognostic biomarkers** are associated with the outcome (independently of which treatment the patients receive), **predictive biomarkers** identify patients which are more likely to benefit from a treatment.

A vast amount of literature is dedicated to univariate subgroups, that is when the subgroups are defined by one baseline covariate or biomarker. In this case, a set of covariates may be investigated marginally by assessing their interactions with treatment. Methodological approaches in this situation may include standardization [4], Bayesian modelling [5], bias reduction via bootstrap [6], and model averaging [7, 8].

Over the last years, there has been an increasing interest in obtaining individualized treatment effect estimates from randomized controlled trials. Building on principles of causal inference, this framework uses a set of baseline covariates to predict the effect of the treatment being tested relative to the control on an individual patient level. These individual treatment effects can be useful to aid treatment decisions, but also can form the basis for identifying a subgroup of responders. There exists extensive literature on machine learning for targeting the PITE through the so-called blip-function [9]. For example, the Super Learner [10] method is a prediction method for creating a weighted combination of many candidate learners, both parametric and non-parametric, which is not limited to randomized clinical trials but could also be used in observational or registry datasets [11, 12]. An overview of several methods for obtaining the predicted individual treatment effect (PITE), including machine learning algorithms and non-parametric models such as tree-based methods is provided by Lamont et.al. [13].

Our work focuses on the multiple regression framework to estimate the individual treatment effects and of importance are those approaches that allow finding subgroups defined by a combination of multiple predictive biomarkers through their interactions with treatment. Lipkovich et.al. [14] classify these methods in the category of *subgroup discovery* while calling for “*principled* data-driven strategies, where all elements are explicitly stated and implemented using solid statistical principles”. Within this framework of parametric models, Zhao et.al. [15] study a parametric scoring system as a function of multiple baseline covariates to estimate subject-specific treatment differences. This work is followed by Li et.al. [16], who propose a two-step predictive enrichment procedure using such a scoring system. Schnell et.al. [17] provide a Bayesian framework for the scoring procedure making use of a Bayesian hierarchical model and propose investigating credible subgroup pairs, which allows controlling for the multiplicity. Most of these parametric procedures can be comprised in a general statistical framework [18]. Choosing a parametric model, however, can be a challenging task. If the true model is not contained in the assumed model class, biased estimators may result. The methods we proposed, however, are flexible and one may incorporate not only covariates linearly but also transformations of them or high order interactions.

The benefit of the PITE strategy to define subgroups by multiple factors can be doomed by the availability of “too many” biomarkers. For example, the analysis may result in subgroups that can be hard to interpret. In diseases like Alzheimer, the cost of obtaining biomarkers may be prohibitively high [19, 20], and therefore one may be interested in using treatment rules with as few biomarkers as possible. In this work, we use penalized regression with a Lasso-type penalty [21] as a model selection and estimation technique. This has the advantage of providing parsimonious models while also providing stable and accurate predictions via shrunken estimates of the model parameters, which becomes even more relevant in cases where the number of predictors to include in the analysis is large. We compare the performance of the Lasso to a maximum likelihood approach that considers all of the available biomarkers to estimate the PITE.

Another issue arising from most of the proposed methods in the literature is that only point estimates are considered, while the uncertainty or precision of such estimates is often unknown. Our aim is to provide confidence intervals for the individual treatment effect and account for the model selection stage. These confidence intervals may then be used to identify subgroups of patients as in [17], where the authors obtain ‘credible subgroup pairs’ in a Bayesian framework [17, 22]. For example, for a disease where no treatment is available, we may be interested in a subgroup in which we include patients with at least some chance of benefit. In this case, we may choose the subset of patients for which the upper bound of the confidence interval is larger than a clinically relevant threshold, even when their point estimate is not. In another scenario, when pharmaceutical companies are negotiating reimbursement with a payer, the identified subgroups may be used to set priorities. Subjects with exceptional benefit, which translates to having the lower bound of the confidence intervals for the individual treatment effect above a certain threshold, may be given the highest priority. Rather than establishing arbitrary cut-off points, the subsets based on confidence intervals allow one to quantify and control the uncertainty of the predictions due to sampling errors.

Specifically, we build on recent developments in inference after selection, also called selective inference [23–25]. We evaluate the performance of confidence intervals for the PITE conditioning on the selection stage or selected model by the Lasso. Since the model is chosen by the data, this conditioning often results in wide confidence intervals [26]. We also evaluate a state-of-the-art method that uses a randomized response for the Lasso [27] and proved to be more powerful, which is expected to translate to narrower confidence intervals. Using the Lasso is then attractive because of the available methods in selective inference. Other modelling techniques, such as forward selection and elastic net, also allow for inference using the same framework. We further explore using the PoSI framework [24, 28], which provides post-selection inference that is universally valid under all possible model selection procedures.

In summary, we make use of recently developed tools to cope with both mentioned issues: selecting the right biomarkers for defining the PITE and providing the uncertainty of the point estimates. Then, we identify subgroups of treatment responders based on estimates of the PITE and its uncertainty.

This article is structured as follow: In Section 2 we introduce the methodology, model assumptions, and estimation methods for a normally distributed outcome. Section 3 illustrates the procedure in a dataset from a clinical trial for a treatment for Alzheimer disease and show the results of a simulation study we perform to evaluate the properties of the methodology. Extensions to time to event and binary endpoints are considered in Section 4, while Section 5 concludes with a discussion and recommendations.

## 2 Methodology

Consider a randomized clinical trial with a parallel group design evaluating the effect of a treatment (*z* = 1) vs. control (*z* = −1), with *n*_*T*_ + *n*_*C*_ = *n* patients. Let *y* be the response variable and **X** = (*x*_1_, ⋯, *x*_*K*_) a set of covariates or biomarkers measured at baseline. The predicted individual treatment effect (PITE), for a subject with covariates **X** = **x** is then defined as the difference between the expected value under treatment and the expected value under control:

D(x)=E[y|z=1,X=x]-E[y|z=-1,X=x].

### 2.1 Subgroup identification

We wish to identify the set of subjects *B* with predicted individual treatment effect larger than a certain threshold of clinical interest, Δ. This set is defined by the characteristics of the subjects **x**, such that *B* = {**x**|*D*(**x**) > Δ}.

A point estimate $\hat{D}\left(x\right)$ of *D*(**x**) may be considered to identify the subgroup of subjects *B*. However, creating the subgroup in this manner ignores the uncertainty around the estimate $\hat{D}\left(x\right)$. We will therefore also consider ${\hat{B}}_{l,\alpha }$, the set of subjects for whom the lower bound of the two-sided (1 − *α*)100% confidence interval for *D*(**x**), ${\hat{D}}_{l,\alpha }\left(x\right)$, is larger than Δ; and the subgroup ${\hat{B}}_{u,\alpha }$ of subjects for whom the upper bound of the confidence interval for *D*(**x**), ${\hat{D}}_{u,\alpha }\left(x\right)$, is larger than Δ. That is:

$$\begin{array}{cc} \hat{B} & =\{x;\hat{D}\left(x\right)>\Delta \},\\ {\hat{B}}_{l,\alpha } & =\{x;{\hat{D}}_{l,\alpha }\left(x\right)>\Delta \},\;\text{and}\\ {\hat{B}}_{u,\alpha } & =\{x;{\hat{D}}_{u,\alpha }\left(x\right)>\Delta \}. \end{array}$$ (1)

The choice of the criterion will depend on the setting, the level of confidence required or the trade-off between overlooking a difference and falsely claiming one.

### 2.2 Model assumptions and estimation methods

We consider a multiple regression model in which the response *y* is related to the treatment received, *K* biomarkers and the treatment-biomarker interactions:

$$y=\alpha +\beta z+\sum_{k=1}^{K}\gamma_{k}x_{k}+z\sum_{k=1}^{K}\delta_{k}x_{k}+\epsilon .$$ (2)

We further assume that the error term *ϵ* is normally distributed with mean 0 and variance *σ*^2^. Under model (2), the PITE for a subject with **X** = **x** is given by

$$D\left(x\right)=2(\beta +\sum_{k=1}^{K}\delta_{k}x_{k}).$$

Denoting with ***θ*** = (*α*, *β*, *γ*_1_, …, *γ*_*K*_, *δ*_1_, …, *δ*_*K*_), the *p*-dimensional vector of all parameters of the linear predictor, and ***l***′ = (0, 2, 0, …, 0, 2*x*_1_, 2*x*_2_, …, 2*x*_*K*_), the PITE can be written in the matrix form ***l***′***θ***.

Below we consider the following approaches to estimate the PITE and its confidence intervals: Maximum Likelihood without model selection (Section 2.2.1), Lasso for model selection with post-selection confidence intervals (Section 2.2.2), Maximum Likelihood with the reduced model selected by the Lasso (Section 2.2.3), Scheffé confidence bounds with the reduced model selected by the Lasso (Section 2.2.4), and Lasso with randomized response for model selection (Section 2.2.5).

#### 2.2.1 Maximum likelihood under the full model (full)

Let **W** = [**1**, **Z**, **X**, **ZX**] denote the design matrix that includes treatment, prognostic, predictive effects, and $\hat{\theta }={\left(W^{\prime }W\right)}^{-1}W^{\prime }y$ the maximum likelihood estimator for ***θ***. Then, the maximum likelihood estimator for the PITE for a subject with **X** = **x** is

$$\hat{D}\left(x\right)=l^{\prime }\hat{\theta }=2(\hat{\beta }+\sum_{k=1}^{K}{\hat{\delta }}_{k}x_{k})∼N\left(D\left(x\right),\tau^{2}\left(x\right)\right),$$

where $\tau^{2}\left(x\right)=\text{Var}\left[\hat{D}\left(x\right)\right]=\sigma^{2}l^{\prime }{\left(W^{\prime }W\right)}^{-1}l.$

We therefore obtain an estimate for the variance of the PITE by estimating *σ*^2^ with

$$S^{2}=\frac{1}{n-d}\left(y^{\prime }y-\hat{\theta }\prime W^{\prime }y\right),$$ (3)

where *d* = 2*K* + 2. This allows to construct a 100(1 − *α*)% confidence intervals for *D*(**x**) using

$$CI_{ML}=[l^{\prime }\hat{\theta }\pm t_{n-d,1-\alpha /2}{\left(S^{2}l^{\prime }{\left(W^{\prime }W\right)}^{-1}l\right)}^{1/2}]$$

where *t*_*n*−*d*,1−*α*/2_ is the 100(1 − *α*/2) percentile of the *t*_*n*−*d*_ distribution [29]. The confidence intervals for the ML method have the property:

$${\text{P}}_{\theta }\left(l^{\prime }\theta \in CI_{ML}\right)=1-\alpha .$$

#### 2.2.2 The Lasso with post-selection confidence intervals (Lasso)

To improve the prediction accuracy and interpretation in the case where many biomarkers are investigated, one may perform model selection in the linear model. The Lasso is a regularization technique for simultaneous estimation and automatic variable selection [21]. The Lasso estimates are defined as

$$\hat{\theta }=\underset{\theta }{\text{arg}\;\text{min}}\{\frac{1}{n}∥y-{W\theta ∥}_{2}^{2}+{\lambda ∥\theta ∥}_{1}\}$$ (4)

where ∥.∥_*p*_ denotes the *L*^*p*^-norm and *λ* is a non-negative regularization parameter or penalty.

The Lasso starts with the same model as in (2) but provides automatic model selection through shrinkage of the estimates of the model coefficients to the extent that some coefficients will be set to zero, resulting in a set of active predictors *E* = {*i*|*θ*_*i*_ ≠ 0}. Obtaining confidence intervals for the parameters in the selected model *E*, and for the corresponding PITE is not straightforward. Recent developments in selective inference [23] have provided new tools for developing confidence intervals for Lasso estimates. The key idea is to perform inference based on the construction of post-selection reference distributions. Conditional on selection of a specific model *E*, **y** is an element of a polyhedron {**Ay** ≤ **b**}. Lee et.al. [25] provide expressions for **A** and **b** for linear regression models with L1 penalty and showed that tests for linear functions of **y** can be based on truncated normal distributions with known limits that depend on **A** and **b**. Confidence intervals that take into account the selection stage can be obtained by inverting such tests. In our work, we use the selective inference results in the context of estimating PITE.

It is important to note here the implications of performing such conditional inference. While conditioning on the event that a specific model *E* is selected, our target of inference changes [24]. Instead of considering the full model with linear predictor **W*θ*** as the generative model, we are now making inference towards a possibly reduced model **W**_*E*_***θ***_*E*_, where **W**_*E*_ is a submatrix of **W** and ***θ***_*E*_ a subvector of ***θ***, containing only the elements corresponding to the terms selected in the model. Note that in the event where no variables involved in the calculation of the PITE are selected, the inference target is equal to zero and the confidence interval reduces to {0}, thus always containing the target on this event [28]. These confidence intervals are designed to have a coverage such that

$${\text{P}}_{\theta_{E}}\left(l_{E}^{\prime }\theta_{E}\in CI_{Lasso}|E\right)=1-\alpha ,$$

that is, conditioning on the selected model *E*.

A usual approach for choosing the Lasso regularization parameter is to select the *λ* that yields to the minimum cross-validation error. However, the confidence intervals in [25] assume a fixed penalization parameter. For this work, we follow the approach in the selectiveInference package [30] and choose the penalization parameter to be determined by the design matrix using:

$$\lambda =\frac{l}{n}\text{E}(∥W^{\prime }{\epsilon ∥}_{\infty });\;\epsilon ∼N\left(0,\sigma^{2}I\right),$$ (5)

where ∥**x**∥_∞_ = max_*i*_ |*x*_*i*_| and *l* is a multiplier for λ. Taking *l* = 2 yields to a desirable consistency and known convergence rates in terms of the *ℓ*_2_-error [31, 32].

When implementing the Lasso, it is well-known that the predictors should be standardized so that they all have the same variance and therefore penalized equally. However, performing the Lasso with interactions poses an additional challenge. In this case, the variables should first be standardized before forming the interactions with treatments. Failing to do so, may lead to inconsistent results when using different coding strategies. Additionally, fitting the Lasso with interactions may not follow soft or hard hierarchy principles, that is, that one or both main effects, respectively, must be included if the interaction is in the model. Not respecting these hierarchy principles may lead to interpretation problems when there is a strong biological rationale that only prognostic biomarkers can be predictive. This may be solved by adding different penalizations to the parameters in the model and leaving the main effects unpenalized. In these cases the conditioning event change and this needs to be taken into account when performing inference.

#### 2.2.3 Maximum likelihood under the reduced model selected by the Lasso (reduced)

For this method, we first use the Lasso to select the predictors to be included in the model and then recalculate the estimates for the coefficients in the reduced model using maximum likelihood.

Naively calculating maximum likelihood boundaries for the estimates under the reduced model is not appropriate after selecting such a model based on the observed data. This is because the maximum likelihood estimates ignore the selection stage as if the predictors that we use for inference were selected before seeing the data. However, we include this method for comparison purposes. Specifically, we compare the width of the resulting intervals and quantify their coverage.

#### 2.2.4 Scheffé confidence bounds (reduced-Scheffe)

Another approach for constructing confidence intervals after selection is provided within the Post Selection Inference (PoSI) framework by [24]. In their work, they argue that to provide universally valid post selection inference, one should perform simultaneous inference in the form of family-wise error control for all parameters in all possible submodels. One way to accomplish this is using Scheffé-type confidence bounds, which provide simultaneous intervals for all possible linear combinations of the parameters.

Let

$$L=[\begin{array}{c} l_{1}^{\prime }\\ l_{2}^{\prime }\\ \vdots \\ l_{n}^{\prime } \end{array}]$$

and *d* = 2*K* + 2, the number of terms in the full model. The Scheffé confidence region fo ***Lθ*** is given by:

$$\{u:\frac{\left(L\hat{\theta }-u\right){}^{\prime }{\left[L{\left(W^{{}^{\prime }}W\right)}^{-1}L{}^{\prime }\right]}^{-1}\left(L\hat{\theta }-u\right)}{dS^{2}}\leq F_{d,n-d}^{\alpha }\},$$

where *S*^2^ is an estimate of *σ*^2^ and $F_{d,n-d}^{\alpha }$ corresponds to the *α*-quantile of the *F* distribution with *d*, *n* − *d* degrees of freedom. Therefore, the simultaneous confidence intervals for ***l***′***θ*** are:

$$\begin{array}{c} CI_{Sch}=[l^{\prime }\hat{\theta }\pm {\left(dF_{d,n-d}^{\alpha }S^{2}l^{\prime }{\left(W^{\prime }W\right)}^{-1}l\right)}^{1/2}] \end{array}$$

We use an estimate from the full model (3) for *S*^2^ as suggested in [24]. This implies that the Scheffé confidence intervals cannot be calculated when the number of predictors is larger than the sample size.

These confidence intervals are *simultaneous* and therefore have the advantage of also accounting for the fact that we are making inference for *n* estimable functions ***l***′***θ***, one for each subject in the dataset. That is,

$$\text{P}(\underset{l\in \text{span}(l)}{\text{sup}}\frac{{\left(l^{\prime }\hat{\theta }-l^{\prime }\theta \right)}^{2}}{S^{2}l^{\prime }{\left(W^{\prime }W\right)}^{-1}l}\leq dF_{d,n-d}^{\alpha })=1-\alpha .$$

#### 2.2.5 Randomized Lasso (rLasso)

The disadvantage of the selective inference framework in Section 2.2.2 is that it may result in very wide confidence intervals since the conditioning is a very stringent constraint. Recently, the use of a randomized response in the Lasso was proposed to obtain more powerful tests and narrower confidence intervals [27]. The gain in power, however, comes at the cost of the quality of the model selection, since it is less likely to select the right covariates when random noise is added in the model fitting process. Instead of using the original response *y* to select the model, the procedure involves drawing *ω* ∼ *Q* from a known distribution *Q* and choose the model using y* = *y* + *ω* via the Lasso. Specifically, the subset of predictors in the selected model *E* is obtained by solving

$$\hat{\theta }=\underset{\theta }{\text{arg}\;\text{min}}\{\frac{1}{n}∥y^{*}-{W\theta ∥}_{2}^{2}+{\lambda ∥\theta ∥}_{1}\}$$

and taking *E* = {*i*|*θ*_*i*_ ≠ 0}. Inference is then carried out using the original data, after adjusting for the selection by considering again the conditional distribution of the estimates in a similar fashion as in the Lasso in Section 2.2.2. However, now one has to condition on the event that a specific model *E* is selected and the added noise, which has a known distribution.

For this work, we considered the distribution of the noise *Q* to be Normal with zero mean and standard deviation *qσ*, with *q* > 0.

## 3 Results

### 3.1 Application: The Alzheimer dataset

To illustrate the proposed methods, we use a clinical trial of an Alzheimer’s disease treatment developed by AbbVie. The data was used in [17] and is available on its online supplementary materials. The dataset contains *n* = 41 subjects, where 25 received treatment and 16 received placebo, and four baseline covariates: severity of disease (continuous), age (from 58 to 90 years), sex (binary), and carrier (binary, the presence of a genetic biomarker). The response of interest is the negative change in severity from baseline to end of the study, where higher values indicate a better outcome. In this example, we also generate 6 normally distributed covariates with mean 0, standard deviation 1, and no effect on the response, having a total of 10 possible predictive biomarkers. The full model includes a term for the overall treatment effect, the main effects for the 10 covariates and their interactions with treatment. The treatment variable is coded (-1, 1) and we standardize the other variables so that they have zero mean and unit standard deviation.

We fit the Lasso and randomized Lasso using the glmnet package [33] in the R software [34]. For both cases, the shrinkage parameter λ = 0.1654 is obtained by (5) using *l* = 1/2 (For comparison purposes, the glmnet cross-validated λ_*min*_ for this example is 0.0865 and the λ_1*se*_ is 0.1661). In the randomized Lasso, the noise added to the response corresponds to *q* = 0.2. For constructing the post-selection confidence intervals for the PITE, we extended the functions in the selectiveInference package [30]. This modification allows the user to get the confidence intervals for an arbitrary input contrast vector, instead of only those vectors that yield to the coefficients of the selected model, as it is implemented in the original package. We use the default options except for the tol.beta parameter in the non-randomized Lasso, which is increased so that terms with estimates with an absolute value larger than 0.1/*N* are retained in the model. We show simulation results in the Supplementary Material with the default value of tol.beta as well, which leads to substantially wider confidence intervals (see Sections 9-12 in S1 File).

**Fig 1:**
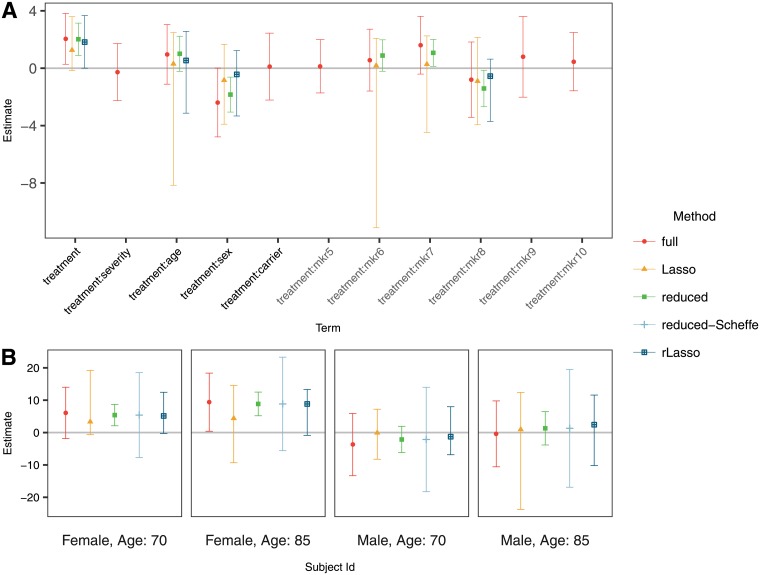
Estimates and confidence intervals for (A) standardized coefficients in the score and (B) four selected subjects (for models that include other covariates besides sex and age, they were set to the mean on the dataset for those covariates).

The estimated coefficients and the resulting two sided 95% confidence intervals are presented in Fig 1A. The variables sex and age are selected in the score after variable selection with the Lasso and randomized Lasso. The Lasso, however, also selected the interactions of three of the simulated variables that are not predictive. If using the full model, the ten covariates need to be considered to define the subgroups.

The PITE for selected combinations of the covariates age and sex is shown Fig 1B. The confidence intervals for the Lasso procedures are similar to those those constructed with ML under the full model in terms of their width. Under the reduced model, the confidence intervals are narrower, but we show in the simulations that they do not have the desired coverage.

### 3.2 Simulation study

We carry out a simulation study using a similar set up to the provided example. The methods described in Section 2 are applied to simulated datasets to evaluate their statistical properties. Usual choices to measure the validity of the PITE estimates are the bias and the mean squared error (MSE), which are defined as $E[E\left[\hat{D}\left(X\right)-D\left(X\right)\right]]$ and $E[E\left[{\left(\hat{D}\left(X\right)-D\left(X\right)\right)}^{2}\right]]$, respectively. The inner expectation is with respect to the distribution of the covariate vector **X** of a future patient and the outer expectation with respect to the (independent) distribution of the data set based on which the model is fit. The MSE is, in fact, the most relevant measure to assess the quality of prediction as it accounts for the error made by introducing too few predictors into the model (and thus shrinking the PITE estimates) as well as the variability of the predictions due to the variation in the estimates of the coefficients because of the limited sample size.

Also of interest are the properties of the confidence intervals. The selective inference procedure aims to control the conditional coverage probability $P({\hat{D}}_{l}\left(x\right)<D_{E}\left(x\right)<{\hat{D}}_{u}\left(x\right)|E)$ for all fixed covariate vectors **x**, being *D*_*E*_(**x**) the true value for the PITE under the selected model *E*, which may be different for each estimation method. In the results, we report the overall expected coverage probability, i.e. $P({\hat{D}}_{l}\left(X\right)<D_{E}\left(X\right)<{\hat{D}}_{u}\left(X\right))$ where we average over the distribution of the data the model is fitted with and also over the distribution of the covariate vector **X** of a future patient.

To give an idea on how well the methods perform to identify the subgroup *B*, we calculate sensitivities and specificities. These quantities are defined as $P(X\in \hat{B}|X\in B)$ and $P(X\notin \hat{B}|X\notin B)$, respectively. We take Δ = 0 to define the subgroups (1).

We use total sample sizes *n* = 40, 100, 220 and 350 with allocation ratio 1:1 (*n*_*C*_ = *n*_*T*_ = *n*/2). The response *y*_*i*_ for subject *i* is simulated such that *y*_*i*_ = *μ*_*i*_ + *ϵ*_*i*_, *i* = 1, …, *n*, with *ϵ*_*i*_ ∼ *N*(0, 1). The mean *μ*_*i*_ depends on the baseline covariates through:

$$\mu_{i}=\alpha +\beta z_{i}+\gamma_{1}x_{1i}+\gamma_{2}x_{2i}+\delta_{1}x_{1i}z_{i}+\delta_{2}x_{2i}z_{i}$$

where *z*_*i*_ ∈ (−1, 1) is the treatment variable (-1:control; 1:experimental treatment). The two covariates are simulated from standardized distributions with zero mean and unit variance, being *x*_1*i*_ a binary variable with equal probability coded as (−1, 1), and *x*_2*i*_ uniformly distributed $\left[-\sqrt{3},\sqrt{3}\right]$. In addition to these two covariates, 8 normally distributed biomarkers with zero mean and unit standard deviation are simulated, resulting in *K* = 10. These additional biomarkers have no effect on the response. The results section includes two choices for ***θ*** = (*α*, *β*, *γ*_1_, *γ*_2_, *δ*_1_, *δ*_2_): a predictive scenario and a null scenario (Table 1). In the Supplementary Material (S1 File), we show additional cases for ***θ*** and total number of biomarkers *K* = 20, 50, 100 and 1000. These scenarios illustrate cases where the number of predictors is larger than the sample size. We also include two scenarios in which the biomarkers are correlated (AR1 correlation with *ρ* = 0.9 and a block correlation structure with two blocks where the correlation within blocks is 0.5).

**Table 1:** Simulation scenarios: Parameters for model 2 used to simulate the datasets. In the null case there are no predictive biomarkers while in the predictive case *X*_1_ and *X*_2_ are predictive.

Case	*α*	*β*	*γ*_1_	*γ*_2_	*δ*_1_	*δ*_2_
1: Null	0.00	0.00	0.00	0.00	0.00	0.00
2: Predictive	0.12	0.12	0.12	0.29	0.12	0.29

The full model contains the treatment effect, the main effects for the covariates and their interactions with treatment. All confidence intervals are two-sided with 95% confidence (*α* = 0.05). We use two degrees of noise, *q* = 0.2 and *q* = 0.8, for the randomized Lasso and denote them rLasso-1 and rLasso-2, respectively.

In addition to the methods described in Section 2 and for comparison purposes, we include in our simulations a method for a model without interactions. In this method, the PITE is estimated using the adjusted average treatment effect (ATE), that is $2\hat{\beta }$ for all individuals, from the following model:

$$y=\alpha +\beta z+\sum_{k=1}^{K}\gamma_{k}x_{k}+\epsilon .$$ (6)

For each scenario, we simulate 1000 datasets and fit their respective models. To calculate the empirical counterparts of the diagnostic measures, we simulate an additional out-of-sample subject for each dataset.

**Fig 2:**
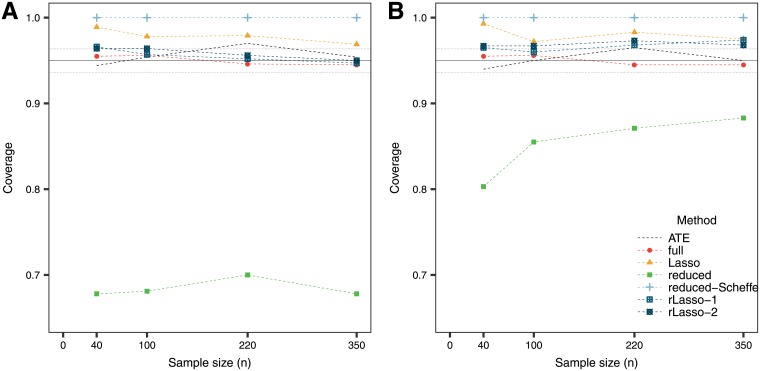
Average coverage of the confidence intervals for the PITE in (A) the null case and (B) the predictive case. The solid line at 95% indicates the target coverage and the bands (dotted lines below and above) indicate ±1.96 standard error for the simulations.

The simulation results are presented in Table 2. An important point to remark is that the MSE is smaller for the ATE method compared to more complicated models when having small sample sizes. Only for larger sample sizes, the models with interactions improve the estimate. While the full model provides unbiased estimates, the mean square error is larger and the confidence intervals wider compared to other methods. The fact that all the variables that one considers are included in the score and involved in the calculation of the confidence intervals is a disadvantage of this method. On the other hand, performing model selection with the Lasso provides biased estimates but with a gain in terms of the MSE. The selective inference confidence intervals for the Lasso can become quite wide, particularly when the sample size is small. In this sense, the randomized version of the Lasso provides a superior alternative. The reduced model provides the narrowest confidence intervals, although they should not be used as they do not have the desired coverage (Fig 2). For this reason, these confidence intervals are then not considered when evaluating sensitivity and specificity. The conservativeness of the reduced model with the Scheffé bounds is reflected in wide confidence intervals and their over-coverage for the PITE.

**Table 2:** Diagnostic measures for 1000 simulations in each null and predictive case. Columns 4 and 5 show the bias and the $\sqrt{MSE}$ for the point estimate of the PITE. The sixth column shows the median width of the confidence intervals for the PITE, and the last columns the proportion of subjects in the identified subgroup when considering the using the limits of the confidence intervals and the point estimates. Since methods reduced and reduced-Scheffe have the same point estimate, bias and MSE are equal.

Case	n	Method	Bias	$\sqrt{MSE}$	Width	Proportion of subjects in subgroup			
						% in ${\hat{B}}_{l}$	% in $\hat{B}$	% in ${\hat{B}}_{u}$	% in B
Null	40	ATE	0.01	0.38	1.48	1.6	53.8	96.0	0.0
	40	full	-0.01	1.65	6.35	2.0	48.5	97.5	0.0
	40	Lasso	-0.00	0.35	5.24	0.6	45.0	93.6	0.0
	40	reduced	0.01	0.87	1.56	15.9	45.5	77.8	0.0
	40	reduced-Scheffe	0.01	0.87	5.80	0.0	45.5	94.1	0.0
	40	rLasso-1	0.00	0.86	3.37	1.4	46.9	94.0	0.0
	40	rLasso-2	-0.01	0.78	2.49	1.6	45.3	93.5	0.0
	100	ATE	-0.00	0.21	0.84	2.1	49.3	97.5	0.0
	100	full	0.02	0.76	2.90	2.2	50.7	97.8	0.0
	100	Lasso	0.01	0.22	3.00	0.9	48.0	93.4	0.0
	100	reduced	0.02	0.56	1.02	17.1	47.2	79.9	0.0
	100	reduced-Scheffe	0.02	0.56	3.24	0.0	47.2	94.7	0.0
	100	rLasso-1	0.01	0.54	2.20	1.9	48.1	94.9	0.0
	100	rLasso-2	0.02	0.46	1.61	1.6	51.2	95.6	0.0
	220	ATE	0.01	0.13	0.54	1.3	52.2	98.3	0.0
	220	full	0.00	0.47	1.79	3.1	49.9	97.7	0.0
	220	Lasso	0.00	0.16	2.15	1.2	47.2	92.9	0.0
	220	reduced	-0.01	0.38	0.73	14.2	47.2	78.0	0.0
	220	reduced-Scheffe	-0.01	0.38	2.21	0.0	47.2	93.8	0.0
	220	rLasso-1	-0.01	0.37	1.48	2.7	49.5	94.7	0.0
	220	rLasso-2	0.00	0.34	1.11	2.1	47.6	93.8	0.0
	350	ATE	-0.01	0.11	0.43	1.7	46.7	97.1	0.0
	350	full	0.01	0.39	1.40	2.8	50.7	97.3	0.0
	350	Lasso	0.00	0.13	1.60	1.3	46.3	93.9	0.0
	350	reduced	0.00	0.31	0.56	16.0	46.4	79.5	0.0
	350	reduced-Scheffe	0.00	0.31	1.65	0.0	46.4	95.7	0.0
	350	rLasso-1	0.01	0.30	1.09	2.4	48.8	94.3	0.0
	350	rLasso-2	0.00	0.28	0.86	2.3	48.2	93.8	0.0
Predictive	40	ATE	0.01	0.75	1.57	10.9	75.2	98.8	64.7
	40	full	-0.03	1.67	6.37	4.8	55.3	97.7	64.7
	40	Lasso	-0.19	0.62	6.15	2.2	54.1	96.3	64.7
	40	reduced	-0.10	0.97	1.85	24.7	54.7	81.7	64.7
	40	reduced-Scheffe	-0.10	0.97	6.72	0.0	54.7	97.6	64.7
	40	rLasso-1	-0.10	0.97	4.02	3.4	54.8	96.2	64.7
	40	rLasso-2	-0.14	0.90	3.02	3.9	53.8	94.0	64.7
	100	ATE	0.00	0.68	0.88	18.9	86.2	100.0	62.3
	100	full	0.02	0.76	2.90	13.3	61.1	95.6	62.3
	100	Lasso	-0.14	0.47	3.00	11.4	59.6	93.9	62.3
	100	reduced	-0.03	0.61	1.31	33.1	59.0	83.4	62.3
	100	reduced-Scheffe	-0.03	0.61	4.10	0.1	59.0	99.8	62.3
	100	rLasso-1	-0.04	0.61	2.52	10.6	60.0	94.0	62.3
	100	rLasso-2	-0.08	0.59	1.94	13.5	57.7	91.1	62.3
	220	ATE	0.01	0.65	0.57	44.2	96.3	100.0	61.5
	220	full	-0.00	0.47	1.79	22.1	61.0	91.4	61.5
	220	Lasso	-0.14	0.34	1.97	16.5	58.6	89.9	61.5
	220	reduced	-0.02	0.41	0.99	39.2	60.7	81.7	61.5
	220	reduced-Scheffe	-0.02	0.41	2.96	2.3	60.7	99.6	61.5
	220	rLasso-1	-0.04	0.42	1.70	19.3	59.0	89.5	61.5
	220	rLasso-2	-0.07	0.43	1.36	24.6	60.0	85.6	61.5
	350	ATE	0.01	0.63	0.45	57.5	97.8	100.0	63.5
	350	full	0.01	0.39	1.40	27.5	63.4	90.9	63.5
	350	Lasso	-0.13	0.29	1.40	24.1	58.1	88.8	63.5
	350	reduced	-0.02	0.33	0.80	41.0	61.8	79.9	63.5
	350	reduced-Scheffe	-0.02	0.33	2.38	7.2	61.8	98.7	63.5
	350	rLasso-1	-0.02	0.33	1.28	27.6	60.8	88.0	63.5
	350	rLasso-2	-0.05	0.35	1.10	29.5	60.1	84.0	63.5

**Fig 3:**
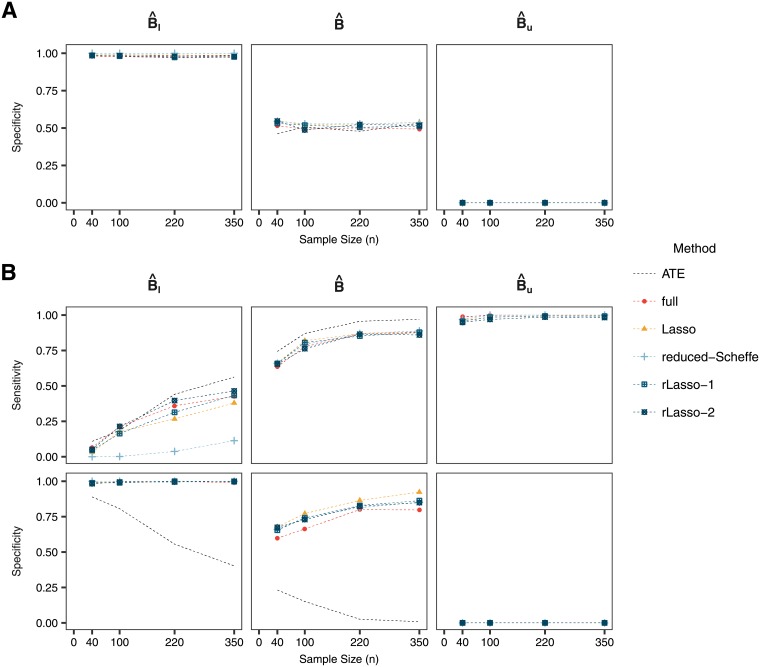
Sensitivity and specificity for (A) the null case and (B) the predictive case. The reduced method is not shown in this figure since the confidence intervals do not meet the desired coverage and the point estimate is the same as for the reduced-Scheffe method. In the null case, it is not possible to calculate sensitivity as no subjects have a positive PITE.

The sensitivity and specificity for the methods are presented in Fig 3. When sample size is small, all methods do not have much sensitivity to identify the subgroup of true treatment responders *B* if one uses the lower bound of the confidence intervals. The sensitivity increases when considering larger sample sizes, with the randomized Lasso achieving similar levels of sensitivity as the full model, and being worse for the Lasso. When considering subgroups obtained with the point estimate or the upper bound of the confidence intervals, shrinkage slightly improves the specificity of the subgroup while the sensitivity is similar across the methods.

The results in the Supplementary Material show that when the number of biomarkers is substantially larger than the sample size the shrinkage methods offer a clear advantage in the PITE estimation (see Section 6 in S1 File). The Lasso not only allows to obtain the estimates and their confidence intervals for the PITE in the less than full rank case, but it also provides better sensitivity and specificity across the different scenarios. It is worth noting that in the completely exploratory setting (very large number of biomarkers and small sample size), the uncertainty of the model selection, may lead to considerably wide confidence intervals that may not be informative. The results for the case of correlated biomarkers are similar to those in the independent case (see Section 7 and 8 in S1 File).

## 4 Extensions to time to event and binary endpoints

In many occasions, studies use either binary or time to event endpoints to determine efficacy. By choosing appropriate models relating outcomes to parameters and covariates, the Lasso is also applicable to these cases [35]. Moreover, both the glmnet and selectiveInference packages in R have the option to fit logistic and Cox proportional hazards models [36]. The randomized Lasso for binary response is considered in [27] by adding perturbation to the parameters in the objective Lasso function to minimize. However, we are not aware of a software implementation of randomized Lasso in time-to-event settings.

In these settings, there is a change in the interpretation of the models. In the survival case, the *PITE* will be defined in terms of the log hazard ratio. We consider a proportional hazards model that contains all covariates in the model and their interactions with treatment:

$$\lambda \left(t,x,z\right)=\lambda_{0}\left(t\right)\text{exp}\{\beta z+\sum_{k=1}^{K}\gamma_{k}x_{k}+z\sum_{k=1}^{K}\delta_{k}x_{k}\}.$$

An additional assumption of independent censoring is needed in this case to obtain a valid causal estimate. If this assumption is violated, one may need to consider methods such as doubly robust estimation [9, 37]. In what follows, we assume only administrative censoring occurs in the trial. Under this model the *PITE* is defined as:

$$D\left(x\right)=\text{log}\lambda \left(t,x,1\right)-\text{log}\lambda \left(t,x,-1\right)=2\times (\beta +\sum_{k=1}^{K}\delta_{k}x_{k}).$$

We wish to identify the set of subjects with covariate vector **X** = **x**, such that the PITE is below zero, that is with a reduction in the hazard:

B={x;D(x)<Δ}.

We consider the subgroup of subjects:

$$\begin{array}{cc} \hat{B} & =\{x;\hat{D}\left(x\right)<\Delta \}\\ {\hat{B}}_{l} & =\{x;{\hat{D}}_{l}\left(x\right)<\Delta \}\\ {\hat{B}}_{u} & =\{x;{\hat{D}}_{u}\left(x\right)<\Delta \} \end{array}$$ (7)

where ${\hat{D}}_{l}\left(x\right)$ and ${\hat{D}}_{u}\left(x\right)$ are the lower and upper bound of the confidence interval for *D*(**x**).

For the binary endpoint case, one may work with the *PITE* using the log odds ratio. Let *π*(**x**, *z*) = P(*Y* = 1|**x**, *z*) and logistic regression model:

$$\text{log}(\frac{\pi (x,z)}{1-\pi (x,z)})=\alpha +\beta z+\sum_{k=1}^{K}\gamma_{k}x_{k}+z\sum_{k=1}^{K}\delta_{k}x_{k}.$$

The *PITE* is then defined as:

$$D\left(x\right)=\text{log}OR\left(x\right)=\text{log}(\frac{\pi (x,1)}{1-\pi (x,1)})-\text{log}(\frac{\pi (x,-1)}{1-\pi (x,-1)})=2\times (\beta +\sum_{k=1}^{K}\delta_{k}x_{k})$$

In the following section, we present an example using time to event data and simulations to evaluate the performance with this type of endpoint.

### 4.1 Application: The prostate cancer dataset

We use a prostate carcinoma dataset from a clinical trial [38] which is available on the web [39]. The data has been analyzed several times in the literature before, and [6] used it to illustrate subgroup analysis by model selection. The dataset consists of 475 subjects randomized to a control group or diethylstilbestrol. The p-value of the log-rank test for the test of the difference in survival between treatment and control was 0.103.

We are interested in identifying subgroups of patients that may benefit from the treatment using the PITE. There are *K* = 6 variables to consider: existence of bone metastasis (bm), disease stage (3 or 4), performance (pf), history of cardiovascular events (hx), age, and weight (wt).

**Fig 4:**
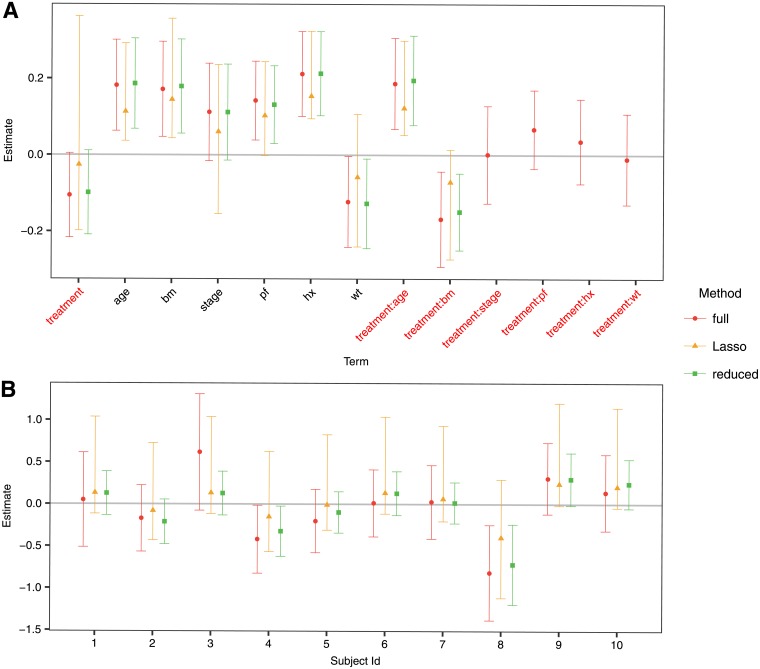
Estimates and confidence intervals for A. the coefficients in the score, and B. the first 10 subjects in the dataset. The ‘full’ legend corresponds to the full model without model selection, the ‘Lasso’ model implements model selection through shrinkage and Selective Inference to derive the confidence intervals, while the ‘reduced’ corresponds to the usual cox model using only the predictors that are selected by the Lasso, but without conditioning on the selection.

The estimated coefficients of the model and their confidence intervals for the proportional hazards model are presented in Fig 4A. The Lasso is fitted with λ = 0.0454, again following (5) with *l* = 1/2 (For comparison purposes, the cross-validated λ_*min*_ for this example is 0.0193 and the λ_1*se*_ is 0.0647). The only interactions that remain in the model after applying the Lasso are the ones of treatment with age and bone metastasis, a similar finding as in [6]. Fig 4B shows the PITE for each of the first 10 individuals in the dataset. In this case, the confidence intervals when using the selective inference framework have a width comparable to those of the full model. Again, the reduced model gives the narrower intervals, but these do not have the desired coverage, as we show in the simulations section.

**Fig 5:**
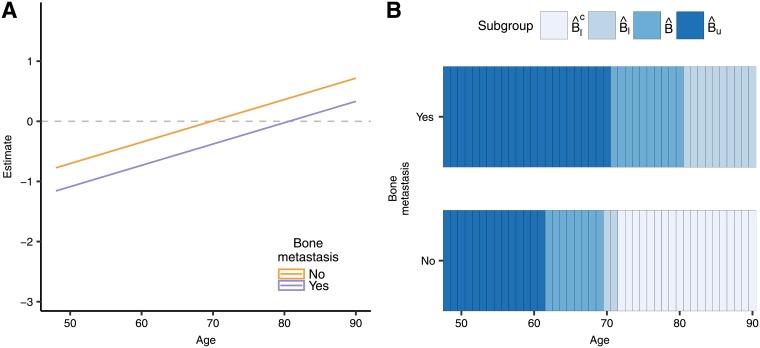
(A) PITE and confidence intervals for combination of levels of variables selected by the Lasso. (B) Identified subgroups by regions of the covariate space.

We restrict the analysis to the variables selected by the Lasso, age and bone metastasis. Fig 5A shows the PITE and confidence intervals when using the Lasso for the combinations of levels of these covariates and Fig 5B illustrates the regions of the covariate space for each identified subgroup. For a patient that already had bone metastasis, the treatment would provide a reduction in the hazard with a 95% confidence when they are younger than 70 years old. However, when they did not have bone metastasis, the treatment has a benefit only for patients younger than 61 years old.

### 4.2 Simulation study

We carry out a simulation study to evaluate whether the methods deliver reliable results regarding the selection of variables in the PITE, the coverage of confidence intervals and the performance of subgroup identification. We consider a trial with a parallel group design, survival endpoint and 6 covariates of interest (*x*_1_ binary and the rest normally distributed with zero mean and unit variance). The data is simulated to have a similar set up as in the prostate cancer data. We assume a proportional hazards following the true model

$$\begin{array}{cc} \lambda_{i}(t)=\lambda_{0}(t)\;\text{exp} & \{0.17x_{1i}+0.18x_{2i}+0.11x_{3i}+0.14x_{4i}+0.21x_{5i}-0.12x_{6i}+\\ & \beta z_{i}+z_{i}\delta_{1}x_{1i}+z_{i}\delta_{2}x_{2i}\}. \end{array}$$

The survival time depended on the administered treatment via *β*, *δ*_1_ and *δ*_2_. A non-informative administrative censoring is introduced by simulating a uniform study time accrual for each patient. We show results for a predictive case (scenario in which *β* = −0.10, *δ*_1_ = −0.08 and *δ*_2_ = 0.18); and for a null case in which there is an overall treatment effect, but no predictive biomarkers (*β* = −0.10, *δ*_1_ = *δ*_2_ = 0). We simulate sample sizes *n* = 100, 400, 700 and 1000, with equal allocation ratio to treatment and control. The number of simulations per scenario is 1000 and we take again Δ = 0 to define the subgroups (7).

**Fig 6:**
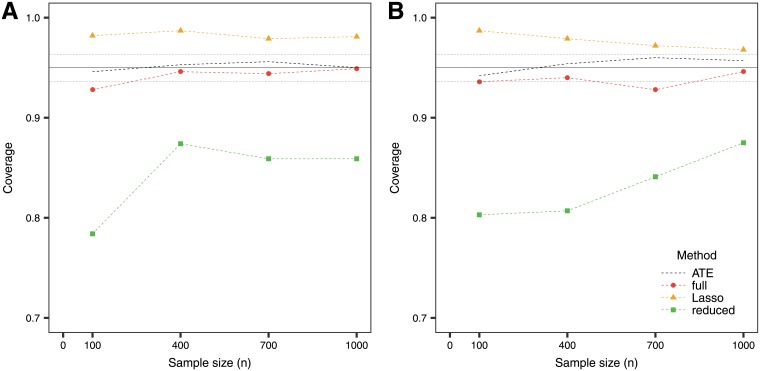
Average coverage of the confidence intervals for the PITE in (A) the null case and (B) the predictive case. The solid line at 95% indicates the target coverage and the bands (dotted lines below and above) indicate ±1.96 standard error for the simulations.

**Fig 7:**
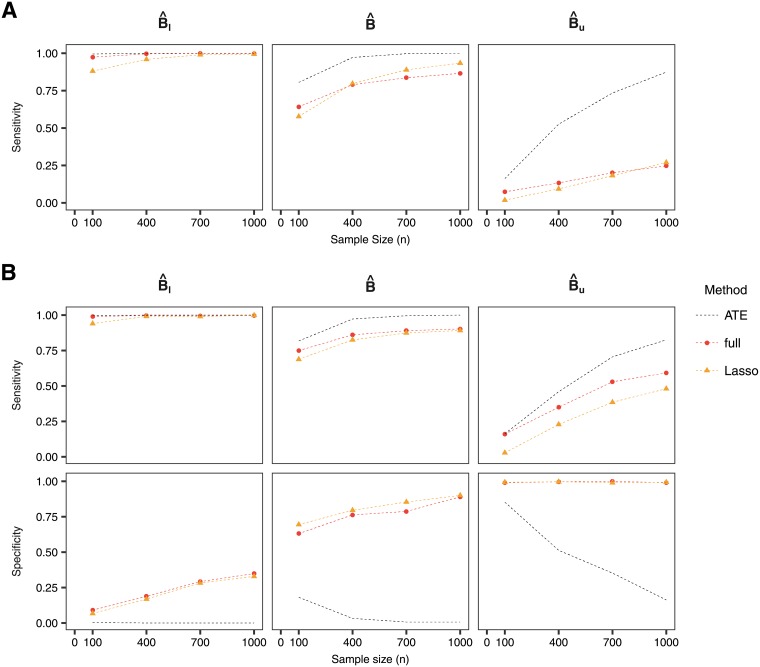
Sensitivity and specificity for (A) the null case and (B) the predictive case when using a survival outcome.

In general, the same trends as in the normal outcome case can be observed when looking at coverage, sensitivity and specificity. The confidence intervals derived with the selective inference framework attain the desired coverage for the PITE, while we show that using the Wald confidence intervals under the reduced model fail to do so (Fig 6). Sensitivity and specificity for the full model and Lasso are comparable (Fig 7), although sensitivity is lower when considering the subgroup defined by the upper bound of the confidence interval.

## 5 Discussion

An essential part of drug development is to provide information on potential treatment effect heterogeneity in a broad patient population to identify subgroups of patients that benefit the most. The current study investigates the use of the predicted individual treatment effect (PITE) to assess treatment heterogeneity, allowing for subgroups that are defined by multiple biomarkers. To ease interpretation of results and to increase prediction precision, reduction of the number of candidate biomarkers may be needed. This variable selection in the model fitting process requires special methods to obtain confidence intervals of parameters with the desired coverage. Selective inference [23] provides tools to achieve this objective. In this paper, we have investigated some of the finite sample properties of these methods for normally distributed data and time to event data. We also showed graphical representations that can help to understand the effect of the investigated drug across the levels of the covariates.

The examples considered in this paper contained a small to a moderate number of biomarkers. Even in this case, the use of model selection greatly improves the interpretation of the identified subgroups as they depend on only a few covariates. However, we have also seen that accounting for selection (uncertainties) in post-selection inference may increase the width of the confidence intervals as compared to the full model in case of moderate numbers of biomarkers which turns into the opposite when the number of markers gets large. In this situation, the tools implemented in this study become relevant as they increase the prediction accuracy and the sensibility and specificity of the identified subgroups.

The methods can also be extended to other model formulations. For example, one may consider a proportional interactions model [40], which assumes that the interaction terms are proportional to their respective main effects. After obtaining the parameter estimates, the PITE can be obtained using the resulting linear model. Confidence intervals could be obtained by extending the parametric bootstrap approach in [40]. However, the authors cautioned against covariate screening since it affects inference on the interaction terms under this model. Valid confidence intervals could be derived based on the conditional distribution of the estimators given a specific model is selected (similar to the approach by [23, 25, 27]). Alternatively, one could account for all possible models as in the Scheffé bounds discussed in Section 2.2.4 ([24]).

Another extensions are models containing higher order interactions or functionals of the baseline covariates may also be considered. In these cases, a small number of biomarkers may turn into a large number of predictors in the model, leading again to the cases where model selection is advantageous.

The proposed intervals are confidence intervals for the expected individual treatment effects, rather than prediction intervals for the subject-level difference in the potential outcomes under treatment and control, and this has implications in their interpretation in terms of the estimand of interest. Developing such prediction intervals in this setting requires an estimate of the subject-level correlation of prediction errors under both treatment and under control conditions. As pointed out by one reviewer, since no study participants are observed under both conditions in parallel study designs, we have no empirical information on this dependence. When no variable selection is performed, one may be able to obtain prediction intervals by assuming different degrees of correlation between the potential outcomes. A conservative approach would assume the situation of perfect correlation between the two potential outcomes [41]. However, combining this with the Selective Inference framework is challenging and a topic of future research.

Availability of the methods and extensions to Cox and logistic models have been discussed, showing that the properties observed in the normal response case remain valid. Since distributional properties of partial maximum likelihood estimators from the Cox model are themselves only asymptotically valid while those for the linear models are exact, larger sample sizes may be needed. Additionally, it should be reminded that deleting relevant covariates from a Cox or a logistic model can lead to biased estimates of the parameters staying in the model.

We have primarily investigated the Lasso and a variation (randomized Lasso), which provided the advantage that the subgroup-defining covariates need not be pre-specified, but only a list of possible covariates is needed. Selective inference applies to some other variable selection methods as well, for example, to forward selection, elastic net, marginal screening, principal components analysis, etc. The message is that no matter which selection technique was used to select biomarkers, the selection stage should be taken into account to provide valid statistical inference.

Finally, as the methods we propose in this study help to identify subgroups of treatment responders in an exploratory manner, it is important to mention that these subgroups should later be confirmed implementing so-called enrichment trials (see [42, 43] for reviews on these methods). Trial designs that incorporate subgroup identification and confirmation together in a comprehensive framework is a topic for future research. Nevertheless, when the subgroups arose from methods that control for overoptimistic results, such as those we used in this paper, they should have a higher chance to be confirmed in later studies.

## Supporting information

S1 File Supplementary material. (PDF)

S2 File PITE R package. Code to reproduce the results of this article.
(ZIP)
