# Supplementary material for: Subgroup identification in clinical trials via the predicted individual treatment effect
Source: PLoS One. 2018 Oct 18;13(10):e0205971. doi: 10.1371/journal.pone.0205971 (PMC6193713; doi:10.1371/journal.pone.0205971)
Supplement: S1 File — (PDF) [file pone.0205971.s001.pdf]

Supplementary Material for Subgroup Identification in Clinical Trials via the Predicted Individual Treatment Effect

Nicolás M. Ballarini[1], Gerd K. Rosenkranz[1], Thomas Jaki[2], Franz König[1] and Martin Posch[1]
[1]Section for Medical Statistics, Center for Medical Statistics, Informatics, and Intelligent Systems, Medical. University of Vienna, Vienna, Austria.
[2]Medical and Pharmaceutical Statistics Research Unit, Department of Mathematics and Statistics, Lancaster University, Lancaster, U.K.
E-mail address: nicolas.ballarini@meduniwien.ac.at

Contents

1 Simulation scenarios **3**

2 Results for 10 Biomarkers **4**
2.1 Percent of selection 4
2.2 Coverage of confidence intervals 5
2.3 Bias, MSE and width 6
2.4 Sensitivity and Specificity 12

3 Results for 20 Biomarkers **15**
3.1 Percent of selection 15
3.2 Coverage of confidence intervals 16
3.3 Bias, MSE and width 17
3.4 Sensitivity and Specificity 23

4 Results for 50 Biomarkers **26**
4.1 Percent of selection 26
4.2 Coverage of confidence intervals 27
4.3 Bias, MSE and width 28
4.4 Sensitivity and Specificity 34

5 Results for 100 Biomarkers **37**
5.1 Percent of selection 37
5.2 Coverage of confidence intervals 38
5.3 Bias, MSE and width 39
5.4 Sensitivity and Specificity 45

6 Results for 1000 Biomarkers **48**
6.1 Percent of selection 49
6.2 Coverage of confidence intervals 50
6.3 Bias, MSE and width 51
6.4 Sensitivity and Specificity 54

7 Results for AR1 correlation structure **57**
7.1 Percent of selection 58
7.2 Coverage of confidence intervals 59
7.3 Bias, MSE and width 60
7.4 Sensitivity and Specificity 66

8 Results for block correlation structure **69**

8.1 Percent of selection . . . 70
8.2 Coverage of confidence intervals . . . 71
8.3 Bias, MSE and width . . . 72
8.4 Sensitivity and Specificity . . . 78

9 Results for 10 Biomarkers. Default tol.beta parameter in selectiveInference 81
9.1 Percent of selection . . . 81
9.2 Coverage of confidence intervals . . . 82
9.3 Bias, MSE and width . . . 83
9.4 Sensitivity and Specificity . . . 89

10 Results for 20 Biomarkers. Default tol.beta parameter in selectiveInference 92
10.1 Percent of selection . . . 92
10.2 Coverage of confidence intervals . . . 93
10.3 Bias, MSE and width . . . 94
10.4 Sensitivity and Specificity . . . 100

11 Results for 50 Biomarkers. Default tol.beta parameter in selectiveInference 103
11.1 Percent of selection . . . 103
11.2 Coverage of confidence intervals . . . 104
11.3 Bias, MSE and width . . . 105
11.4 Sensitivity and Specificity . . . 111

12 Results for 100 Biomarkers. Default tol.beta parameter in selectiveInference 114
12.1 Percent of selection . . . 114
12.2 Coverage of confidence intervals . . . 115
12.3 Bias, MSE and width . . . 116
12.4 Sensitivity and Specificity . . . 122

1 Simulation scenarios

This document provides additional simulation results. The set up for this numerical study is the same as the presented in the manuscript, although here we explore the cases of increasing number of nuisance biomarkers (K=10, 20, 50 and 100) and more cases for the underlying generative model.

The response for subject i is simulated to have mean

$$\mu_i = \alpha + \beta z_i + \gamma_1 x_{1i} + \gamma_2 x_{2i} + \delta_1 x_{1i} z_i + \delta_2 x_{2i} z_i$$

where $z_i \in (-1, 1)$ (-1:control; 1:treatment) is the treatment variable, and the two covariates are simulated from standardized distributions with mean 0 and variance 1, being x_{1i} a binary variable with equal probability coded as $(-1, 1)$, and x_{2i} a uniformly distributed $[-\sqrt{3}, \sqrt{3}]$. In addition to these two covariates, normally distributed biomarkers were simulated with no effect on the response. We consider having a total of 10, 20, 50 and 100 biomarkers in the model. The full model contains the treatment effect, the main effects for all covariates under study and their interactions with treatment.

We simulate 1000 datasets for each of sample sizes ($n = 40, 100, 200$ and 350) and for six choices for $\boldsymbol{\theta} = (\alpha, \beta, \gamma_1, \gamma_2, \delta_1, \delta_2)$ (Table 1). The 'null' and 'predictive' scenarios from the main manuscript correspond here to cases 1 and 4, respectively. All confidence intervals are built using 95% confidence ($\alpha = 0.05$). We use two degrees of noise, $q = 0.2$ (rLasso-1) and $q = 0.8$ (rLasso-1), for the randomized Lasso.

We fit the Lasso and randomized Lasso using the glmnet package in the R software. For constructing the post-selection confidence intervals for the PITE, we extended the functions in the selectiveInference package. This modification allows the user to get the confidence intervals for an arbitrary input contrast vector, instead of only those vectors that yield to the coefficients of the selected model, as it is implemented in the original package. We use the default options except for the tol.beta parameter in the non-randomized Lasso, which is increased so that terms with estimates with an absolute value larger than 0.1/N are retained in the model (Sections 2-8). We show simulation results in Sections 9-12 with the default value of tol.beta as well, which leads to substantially wider confidence intervals.

Table 1: Choices of parameters for the simulation scenarios

Case	α	β	γ_1	γ_2	δ_1	δ_2
1: No predictive markers	0.000	0.000	0.000	0.000	0.000	0.000
2: X_2 is predictive	0.000	0.000	0.000	0.289	0.000	0.289
3: X_1 is predictive	0.125	0.125	0.125	0.000	0.125	0.000
4: X_1 and X_2 are predictive	0.125	0.125	0.125	0.289	0.125	0.289
5: Overall treatment effect	0.125	0.125	0.000	0.000	0.000	0.000
6: Overall treatment effect and X_1 and X_2 are predictive	0.250	0.250	0.125	0.289	0.125	0.289

2 Results for 10 Biomarkers

2.1 Percent of selection

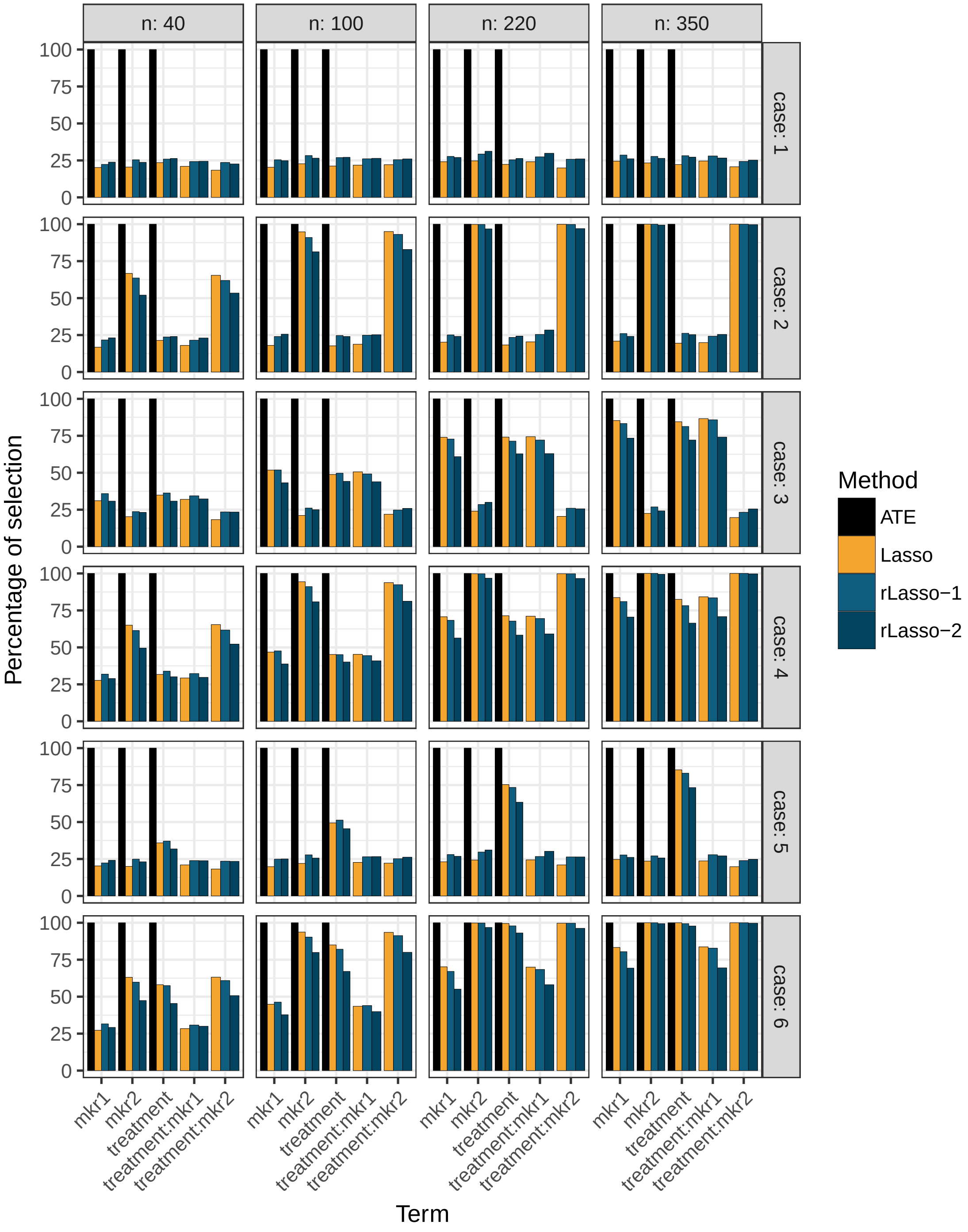

Figure 1: Selection probabilities for relevant terms in the model by method, case and sample size for 10 biomarkers. The full model is not represented here as all variables are included 100% of the times. The reduced model is not represented as it has the same values as the Lasso

2.2 Coverage of confidence intervals

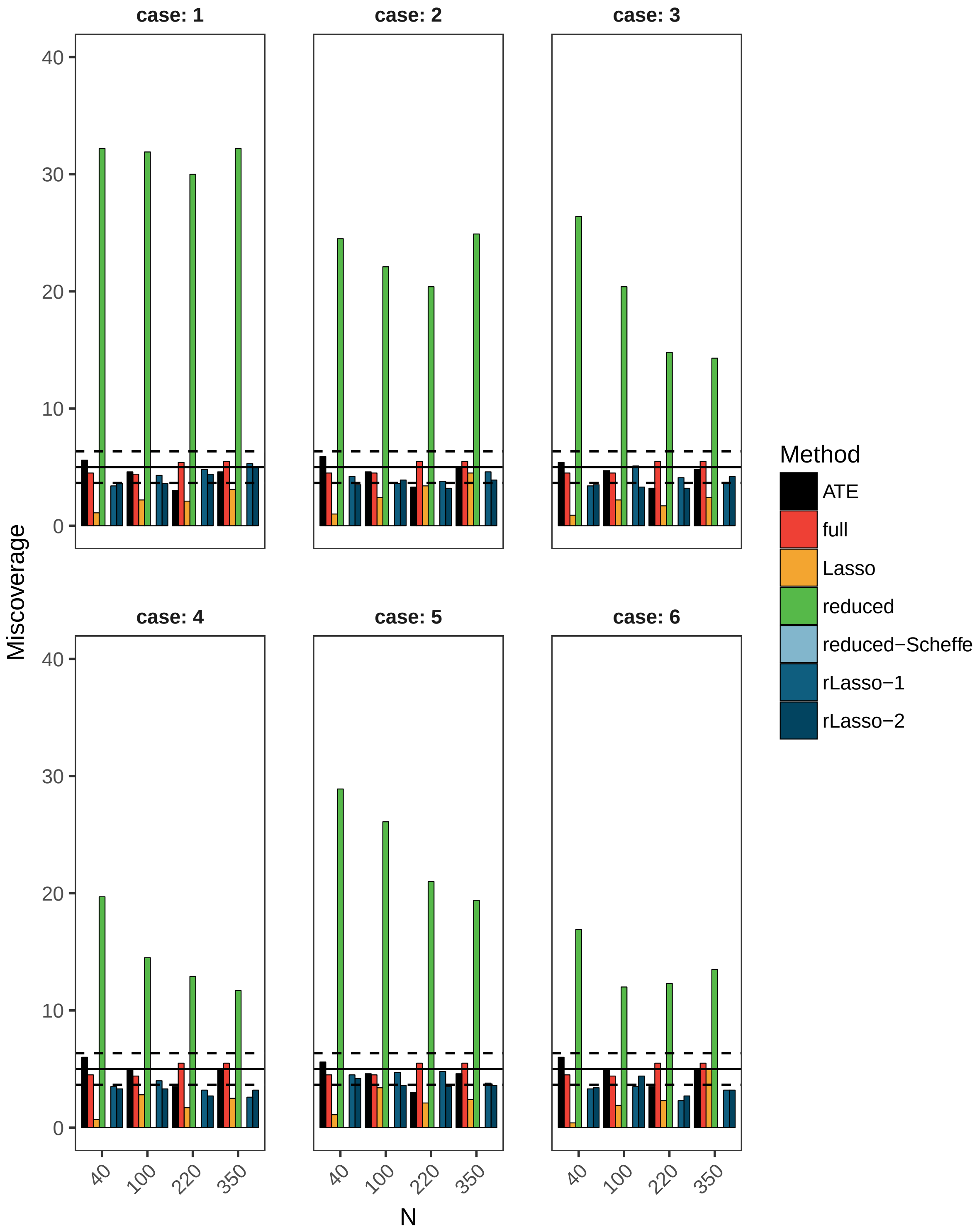

Figure 2: Average miscoverage of the confidence intervals for the PITE by method, case and sample size for 10 biomarkers. The line at 5% indicates the target miscoverage.

2.3 Bias, MSE and width

Table 2: Diagnostic measures for case 1 with 10 biomarkers. Columns 4 and 5 show the bias and the $\sqrt{MSE}$ for the point estimate of the PITE. The sixth column shows the median width of the confidence intervals for the PITE, and the last columns show the proportion of subjects in the identified subgroup when considering the using the limits of the confidence intervals and the point estimates. Since methods reduced and reduced-Scheffe have the same point estimate, bias and MSE are equal.

Case	N	Method	Bias	$\sqrt{MSE}$	Width	% in $\hat{B}_l$	% in $\hat{B}$	% in $\hat{B}_u$	% in B
1	40	ATE	0.01	0.38	1.48	1.6	53.8	96.0	0
1	40	full	-0.01	1.65	6.35	2.0	48.5	97.5	0
1	40	Lasso	0.00	0.35	5.24	0.6	45.0	93.6	0
1	40	reduced	0.01	0.87	1.56	15.9	45.5	77.8	0
1	40	reduced-Scheffe	0.01	0.87	5.80	0.0	45.5	94.1	0
1	40	rLasso-1	0.00	0.86	3.37	1.4	46.9	94.0	0
1	40	rLasso-2	-0.01	0.78	2.49	1.6	45.3	93.5	0
1	100	ATE	0.00	0.21	0.84	2.1	49.3	97.5	0
1	100	full	0.02	0.76	2.90	2.2	50.7	97.8	0
1	100	Lasso	0.01	0.22	3.00	0.9	48.0	93.4	0
1	100	reduced	0.02	0.56	1.02	17.1	47.2	79.9	0
1	100	reduced-Scheffe	0.02	0.56	3.24	0.0	47.2	94.7	0
1	100	rLasso-1	0.01	0.54	2.20	1.9	48.1	94.9	0
1	100	rLasso-2	0.02	0.46	1.61	1.6	51.2	95.6	0
1	220	ATE	0.01	0.13	0.54	1.3	52.2	98.3	0
1	220	full	0.00	0.47	1.79	3.1	49.9	97.7	0
1	220	Lasso	0.00	0.16	2.15	1.2	47.2	92.9	0
1	220	reduced	-0.01	0.38	0.73	14.2	47.2	78.0	0
1	220	reduced-Scheffe	-0.01	0.38	2.21	0.0	47.2	93.8	0
1	220	rLasso-1	-0.01	0.37	1.48	2.7	49.5	94.7	0
1	220	rLasso-2	0.00	0.34	1.11	2.1	47.6	93.8	0
1	350	ATE	-0.01	0.11	0.43	1.7	46.7	97.1	0
1	350	full	0.01	0.39	1.40	2.8	50.7	97.3	0
1	350	Lasso	0.00	0.13	1.60	1.3	46.3	93.9	0
1	350	reduced	0.00	0.31	0.56	16.0	46.4	79.5	0
1	350	reduced-Scheffe	0.00	0.31	1.65	0.0	46.4	95.7	0
1	350	rLasso-1	0.01	0.30	1.09	2.4	48.8	94.3	0
1	350	rLasso-2	0.00	0.28	0.86	2.3	48.2	93.8	0

Table 3: Diagnostic measures for case 2 with 10 biomarkers. Columns 4 and 5 show the bias and the $\sqrt{MSE}$ for the point estimate of the PITE. The sixth column shows the median width of the confidence intervals for the PITE, and the last columns show the proportion of subjects in the identified subgroup when considering the using the limits of the confidence intervals and the point estimates. Since methods reduced and reduced-Scheffe have the same point estimate, bias and MSE are equal.

Case	N	Method	Bias	$\sqrt{MSE}$	Width	% in $\hat{B}_l$	% in $\hat{B}$	% in $\hat{B}_u$	% in B
2	40	ATE	0.00	0.71	1.55	2.1	54.0	96.20	51.1
2	40	full	-0.03	1.67	6.37	3.2	49.7	97.10	51.1
2	40	Lasso	-0.02	0.55	5.99	1.1	45.8	95.10	51.1
2	40	reduced	-0.02	0.93	1.72	17.9	46.7	76.30	51.1
2	40	reduced-Scheffe	-0.02	0.93	6.32	0.0	46.7	96.60	51.1
2	40	rLasso-1	-0.01	0.94	3.77	2.9	48.6	94.69	51.1
2	40	rLasso-2	-0.02	0.88	2.91	3.5	49.1	93.40	51.1
2	100	ATE	0.00	0.63	0.88	2.1	50.4	97.50	49.2
2	100	full	0.02	0.76	2.90	6.4	50.1	93.80	49.2
2	100	Lasso	0.02	0.36	2.79	10.2	49.5	91.60	49.2
2	100	reduced	0.02	0.56	1.21	27.5	50.0	73.30	49.2
2	100	reduced-Scheffe	0.02	0.56	3.79	0.1	50.0	99.70	49.2
2	100	rLasso-1	0.01	0.55	2.42	9.0	49.6	90.80	49.2
2	100	rLasso-2	0.03	0.53	1.81	13.2	50.6	88.80	49.2
2	220	ATE	0.02	0.59	0.57	1.5	53.3	98.20	48.4
2	220	full	0.00	0.47	1.79	12.2	48.9	87.10	48.4
2	220	Lasso	0.00	0.24	1.87	15.8	49.0	83.30	48.4
2	220	reduced	0.00	0.38	0.84	32.6	49.8	66.10	48.4
2	220	reduced-Scheffe	0.00	0.38	2.53	0.5	49.8	98.80	48.4
2	220	rLasso-1	-0.01	0.37	1.57	18.6	47.1	82.60	48.4
2	220	rLasso-2	0.00	0.36	1.24	21.4	48.8	76.40	48.4
2	350	ATE	0.00	0.58	0.44	2.1	48.5	97.10	51.0
2	350	full	0.01	0.39	1.40	15.9	51.4	83.80	51.0
2	350	Lasso	0.00	0.20	1.32	20.9	49.8	79.40	51.0
2	350	reduced	0.00	0.31	0.65	34.5	50.0	64.50	51.0
2	350	reduced-Scheffe	0.00	0.31	1.92	5.0	50.0	94.20	51.0
2	350	rLasso-1	0.01	0.30	1.15	21.5	49.9	76.00	51.0
2	350	rLasso-2	0.00	0.28	0.98	26.2	50.5	74.20	51.0

Table 4: Diagnostic measures for case 3 with 10 biomarkers. Columns 4 and 5 show the bias and the $\sqrt{MSE}$ for the point estimate of the PITE. The sixth column shows the median width of the confidence intervals for the PITE, and the last columns show the proportion of subjects in the identified subgroup when considering the using the limits of the confidence intervals and the point estimates. Since methods reduced and reduced-Scheffe have the same point estimate, bias and MSE are equal.

Case	N	Method	Bias	$\sqrt{MSE}$	Width	% in $\hat{B}_l$	% in $\hat{B}$	% in $\hat{B}_u$	% in B
3	40	ATE	0.02	0.46	1.51	10.80	76.2	99.3	49.0
3	40	full	-0.01	1.67	6.37	4.30	56.5	98.7	49.0
3	40	Lasso	-0.17	0.45	5.50	1.10	56.1	95.8	49.0
3	40	reduced	-0.07	0.91	1.65	21.80	55.5	82.4	49.0
3	40	reduced-Scheffe	-0.07	0.91	6.17	0.00	55.5	96.1	49.0
3	40	rLasso-1	-0.08	0.90	3.59	2.30	54.2	95.9	49.0
3	40	rLasso-2	-0.13	0.83	2.73	3.00	54.3	94.4	49.0
3	100	ATE	0.00	0.33	0.84	19.80	88.1	100.0	50.0
3	100	full	0.01	0.77	2.92	5.80	62.4	99.1	50.0
3	100	Lasso	-0.15	0.35	3.34	3.70	63.2	96.6	50.0
3	100	reduced	-0.04	0.60	1.19	27.60	61.7	88.4	50.0
3	100	reduced-Scheffe	-0.04	0.60	3.72	0.00	61.7	97.9	50.0
3	100	rLasso-1	-0.05	0.60	2.32	5.30	61.1	96.2	50.0
3	100	rLasso-2	-0.07	0.52	1.75	5.40	64.6	97.3	50.0
3	220	ATE	0.00	0.29	0.55	46.60	96.9	100.0	50.7
3	220	full	0.00	0.47	1.79	11.20	66.6	98.8	50.7
3	220	Lasso	-0.13	0.27	2.09	8.60	71.0	98.3	50.7
3	220	reduced	-0.02	0.41	0.88	34.70	69.2	93.2	50.7
3	220	reduced-Scheffe	-0.02	0.41	2.63	0.00	69.2	99.4	50.7
3	220	rLasso-1	-0.04	0.41	1.55	11.11	67.5	97.8	50.7
3	220	rLasso-2	-0.05	0.40	1.20	13.30	68.0	96.3	50.7
3	350	ATE	0.00	0.27	0.43	61.10	98.3	100.0	48.1
3	350	full	0.01	0.39	1.40	17.70	69.6	98.8	48.1
3	350	Lasso	-0.12	0.24	1.58	13.00	70.2	97.7	48.1
3	350	reduced	-0.02	0.33	0.72	37.80	69.8	93.5	48.1
3	350	reduced-Scheffe	-0.02	0.33	2.14	0.10	69.8	99.9	48.1
3	350	rLasso-1	-0.01	0.33	1.19	18.00	69.9	97.6	48.1
3	350	rLasso-2	-0.04	0.34	0.98	21.40	67.7	95.9	48.1

Table 5: Diagnostic measures for case 4 with 10 biomarkers. Columns 4 and 5 show the bias and the $\sqrt{MSE}$ for the point estimate of the PITE. The sixth column shows the median width of the confidence intervals for the PITE, and the last columns show the proportion of subjects in the identified subgroup when considering the using the limits of the confidence intervals and the point estimates. Since methods reduced and reduced-Scheffe have the same point estimate, bias and MSE are equal.

Case	N	Method	Bias	$\sqrt{MSE}$	Width	% in $\hat{B}_l$	% in $\hat{B}$	% in $\hat{B}_u$	% in B
4	40	ATE	0.01	0.75	1.57	10.9	75.2	98.8	64.7
4	40	full	-0.03	1.67	6.37	4.8	55.3	97.7	64.7
4	40	Lasso	-0.19	0.62	6.15	2.2	54.1	96.3	64.7
4	40	reduced	-0.10	0.97	1.85	24.7	54.7	81.7	64.7
4	40	reduced-Scheffe	-0.10	0.97	6.72	0.0	54.7	97.6	64.7
4	40	rLasso-1	-0.10	0.97	4.02	3.4	54.8	96.2	64.7
4	40	rLasso-2	-0.14	0.90	3.02	3.9	53.8	94.0	64.7
4	100	ATE	0.00	0.68	0.88	18.9	86.2	100.0	62.3
4	100	full	0.02	0.76	2.90	13.3	61.1	95.6	62.3
4	100	Lasso	-0.14	0.47	3.00	11.4	59.6	93.9	62.3
4	100	reduced	-0.03	0.61	1.31	33.1	59.0	83.4	62.3
4	100	reduced-Scheffe	-0.03	0.61	4.10	0.1	59.0	99.8	62.3
4	100	rLasso-1	-0.04	0.61	2.52	10.6	60.0	94.0	62.3
4	100	rLasso-2	-0.08	0.59	1.94	13.5	57.7	91.1	62.3
4	220	ATE	0.01	0.65	0.57	44.2	96.3	100.0	61.5
4	220	full	0.00	0.47	1.79	22.1	61.0	91.4	61.5
4	220	Lasso	-0.14	0.34	1.97	16.5	58.6	89.9	61.5
4	220	reduced	-0.02	0.41	0.99	39.2	60.7	81.7	61.5
4	220	reduced-Scheffe	-0.02	0.41	2.96	2.3	60.7	99.6	61.5
4	220	rLasso-1	-0.04	0.42	1.70	19.3	59.0	89.5	61.5
4	220	rLasso-2	-0.07	0.43	1.36	24.6	60.0	85.6	61.5
4	350	ATE	0.01	0.63	0.45	57.5	97.8	100.0	63.5
4	350	full	0.01	0.39	1.40	27.5	63.4	90.9	63.5
4	350	Lasso	-0.13	0.29	1.40	24.1	58.1	88.8	63.5
4	350	reduced	-0.02	0.33	0.80	41.0	61.8	79.9	63.5
4	350	reduced-Scheffe	-0.02	0.33	2.38	7.2	61.8	98.7	63.5
4	350	rLasso-1	-0.02	0.33	1.28	27.6	60.8	88.0	63.5
4	350	rLasso-2	-0.05	0.35	1.10	29.5	60.1	84.0	63.5

Table 6: Diagnostic measures for case 5 with 10 biomarkers. Columns 4 and 5 show the bias and the $\sqrt{MSE}$ for the point estimate of the PITE. The sixth column shows the median width of the confidence intervals for the PITE, and the last columns show the proportion of subjects in the identified subgroup when considering the using the limits of the confidence intervals and the point estimates. Since methods reduced and reduced-Scheffe have the same point estimate, bias and MSE are equal.

Case	N	Method	Bias	$\sqrt{MSE}$	Width	% in $\hat{B}_l$	% in $\hat{B}$	% in $\hat{B}_u$	% in B
5	40	ATE	0.01	0.38	1.48	10.8	76.0	99.4	100
5	40	full	0.02	1.65	6.34	3.2	57.0	98.6	100
5	40	Lasso	-0.17	0.40	5.68	1.1	57.5	94.1	100
5	40	reduced	-0.06	0.90	1.61	21.5	56.6	83.0	100
5	40	reduced-Scheffe	-0.06	0.90	6.06	0.0	56.6	94.6	100
5	40	rLasso-1	-0.08	0.88	3.52	2.8	55.7	94.2	100
5	40	rLasso-2	-0.13	0.81	2.53	2.8	53.5	93.8	100
5	100	ATE	0.00	0.21	0.84	20.0	88.7	100.0	100
5	100	full	0.02	0.77	2.91	6.1	64.1	99.4	100
5	100	Lasso	-0.16	0.29	3.09	3.7	64.7	95.4	100
5	100	reduced	-0.04	0.58	1.10	27.5	62.7	88.4	100
5	100	reduced-Scheffe	-0.04	0.58	3.46	0.0	62.7	96.5	100
5	100	rLasso-1	-0.05	0.56	2.23	5.0	61.8	95.4	100
5	100	rLasso-2	-0.07	0.49	1.68	4.7	64.8	96.6	100
5	220	ATE	0.01	0.13	0.54	46.2	96.9	100.0	100
5	220	full	0.00	0.47	1.79	9.1	71.3	99.7	100
5	220	Lasso	-0.13	0.22	2.18	6.3	76.2	98.1	100
5	220	reduced	-0.02	0.40	0.81	34.5	72.3	93.4	100
5	220	reduced-Scheffe	-0.02	0.40	2.45	0.0	72.3	98.5	100
5	220	rLasso-1	-0.04	0.39	1.51	9.4	71.6	98.2	100
5	220	rLasso-2	-0.05	0.36	1.14	10.9	71.7	96.9	100
5	350	ATE	-0.01	0.11	0.43	61.2	98.3	100.0	100
5	350	full	0.01	0.39	1.40	12.4	75.1	99.7	100
5	350	Lasso	-0.12	0.19	1.62	9.7	82.0	98.9	100
5	350	reduced	-0.01	0.32	0.64	41.3	77.7	96.1	100
5	350	reduced-Scheffe	-0.01	0.32	1.91	0.0	77.7	99.3	100
5	350	rLasso-1	0.00	0.32	1.13	14.5	80.0	98.6	100
5	350	rLasso-2	-0.04	0.30	0.92	15.7	77.9	98.4	100

Table 7: Diagnostic measures for case 6 with 10 biomarkers. Columns 4 and 5 show the bias and the $\sqrt{MSE}$ for the point estimate of the PITE. The sixth column shows the median width of the confidence intervals for the PITE, and the last columns show the proportion of subjects in the identified subgroup when considering the using the limits of the confidence intervals and the point estimates. Since methods reduced and reduced-Scheffe have the same point estimate, bias and MSE are equal.

Case	N	Method	Bias	$\sqrt{MSE}$	Width	% in $\hat{B}_l$	% in $\hat{B}$	% in $\hat{B}_u$	% in B
6	40	ATE	0.01	0.75	1.57	26.9	89.9	99.8	75.9
6	40	full	-0.02	1.67	6.37	6.5	60.6	98.5	75.9
6	40	Lasso	-0.32	0.69	6.34	2.6	64.0	97.5	75.9
6	40	reduced	-0.11	0.99	1.92	31.7	62.4	88.3	75.9
6	40	reduced-Scheffe	-0.11	0.99	6.88	0.0	62.4	98.2	75.9
6	40	rLasso-1	-0.12	1.00	4.16	5.4	62.5	96.7	75.9
6	40	rLasso-2	-0.20	0.97	3.23	5.6	62.7	95.4	75.9
6	100	ATE	0.00	0.68	0.88	59.5	98.9	100.0	74.3
6	100	full	0.02	0.76	2.90	17.9	69.4	97.3	74.3
6	100	Lasso	-0.22	0.52	2.86	18.3	72.8	97.3	74.3
6	100	reduced	-0.01	0.64	1.40	43.6	70.4	91.1	74.3
6	100	reduced-Scheffe	-0.01	0.64	4.34	0.6	70.4	99.9	74.3
6	100	rLasso-1	-0.02	0.62	2.58	17.3	71.2	97.3	74.3
6	100	rLasso-2	-0.08	0.66	2.04	20.3	70.2	94.7	74.3
6	220	ATE	0.01	0.65	0.57	94.5	100.0	100.0	74.1
6	220	full	0.00	0.47	1.79	32.7	70.4	96.6	74.1
6	220	Lasso	-0.16	0.37	1.72	32.7	74.3	96.4	74.1
6	220	reduced	0.00	0.40	1.02	51.5	73.7	92.4	74.1
6	220	reduced-Scheffe	0.00	0.40	3.06	8.3	73.7	99.9	74.1
6	220	rLasso-1	-0.01	0.40	1.69	32.5	73.2	96.6	74.1
6	220	rLasso-2	-0.01	0.42	1.41	38.5	73.8	95.9	74.1
6	350	ATE	0.01	0.63	0.45	98.6	100.0	100.0	75.1
6	350	full	0.01	0.39	1.40	39.6	74.7	96.7	75.1
6	350	Lasso	-0.14	0.31	1.25	37.9	74.4	94.7	75.1
6	350	reduced	-0.01	0.32	0.81	54.9	74.5	91.2	75.1
6	350	reduced-Scheffe	-0.01	0.32	2.39	17.1	74.5	100.0	75.1
6	350	rLasso-1	0.00	0.32	1.25	40.5	73.4	95.0	75.1
6	350	rLasso-2	-0.01	0.34	1.11	45.7	74.4	94.1	75.1

2.4 Sensitivity and Specificity

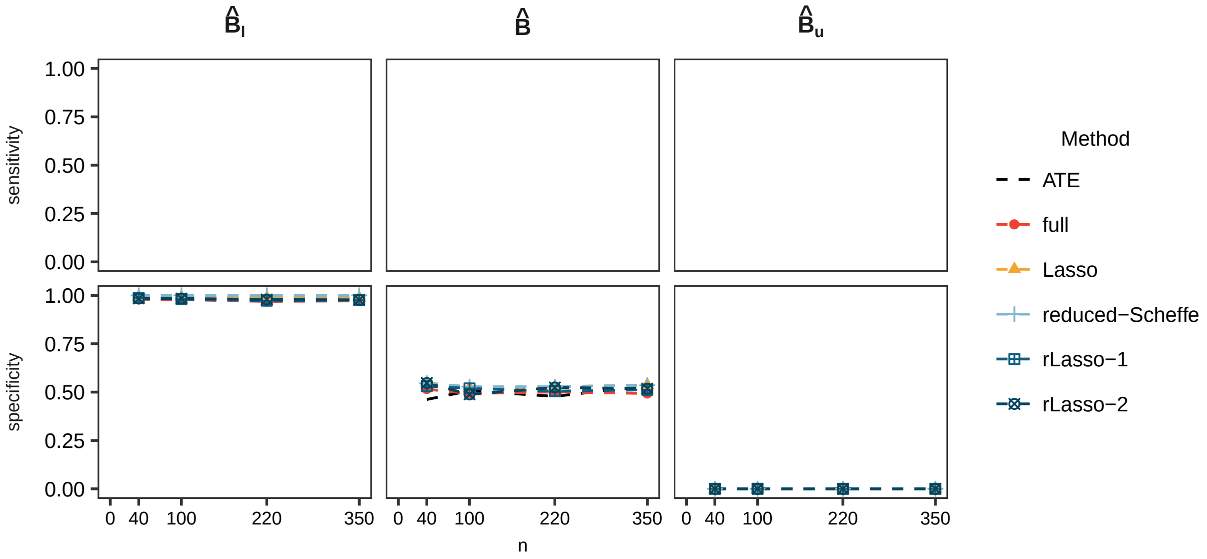

Figure 3: Specificity for identified subgroup by method and sample size for Case 1 and 10 biomarkers. Sensitivity is not shown as there are no patients with $D(X) > 0$

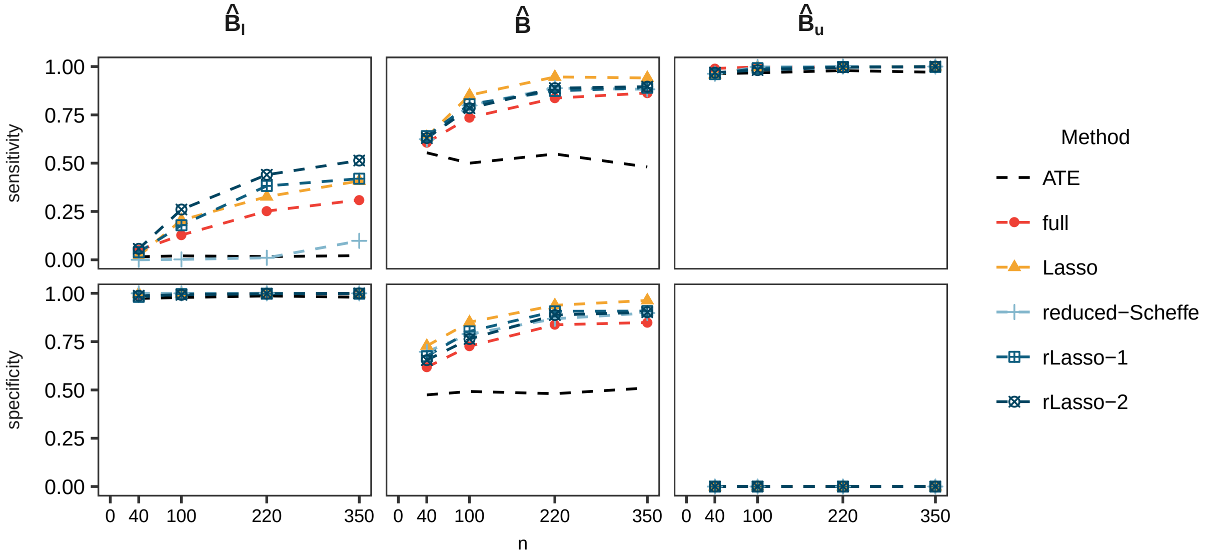

Figure 4: Sensitivity and specificity for identified subgroup by method and sample size for Case 2 and 10 biomarkers.

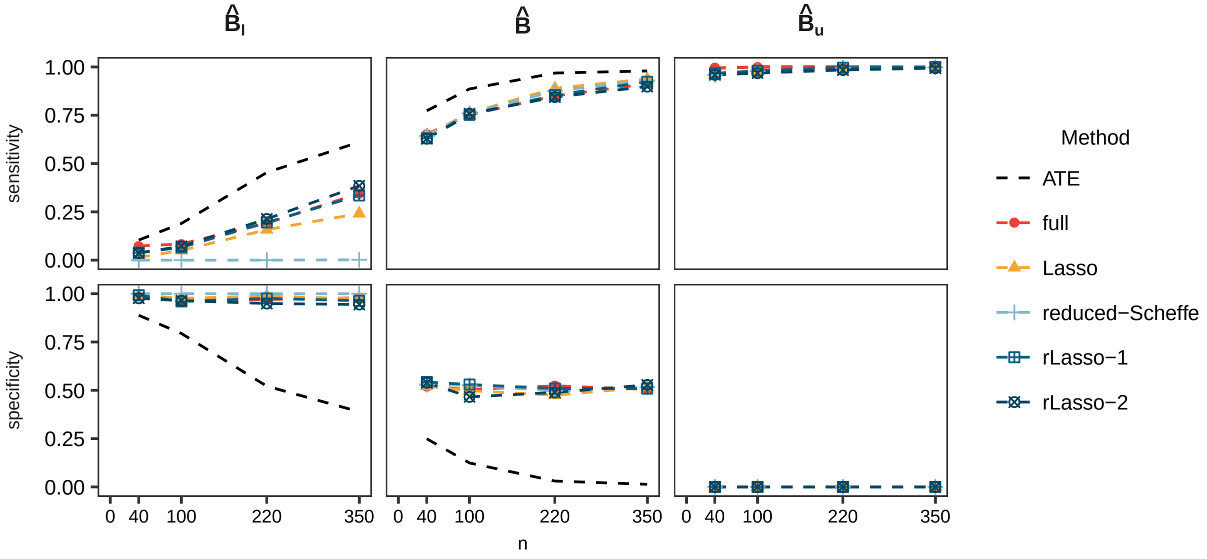

Figure 5: Sensitivity and specificity for identified subgroup by method and sample size for Case 3 and 10 biomarkers.

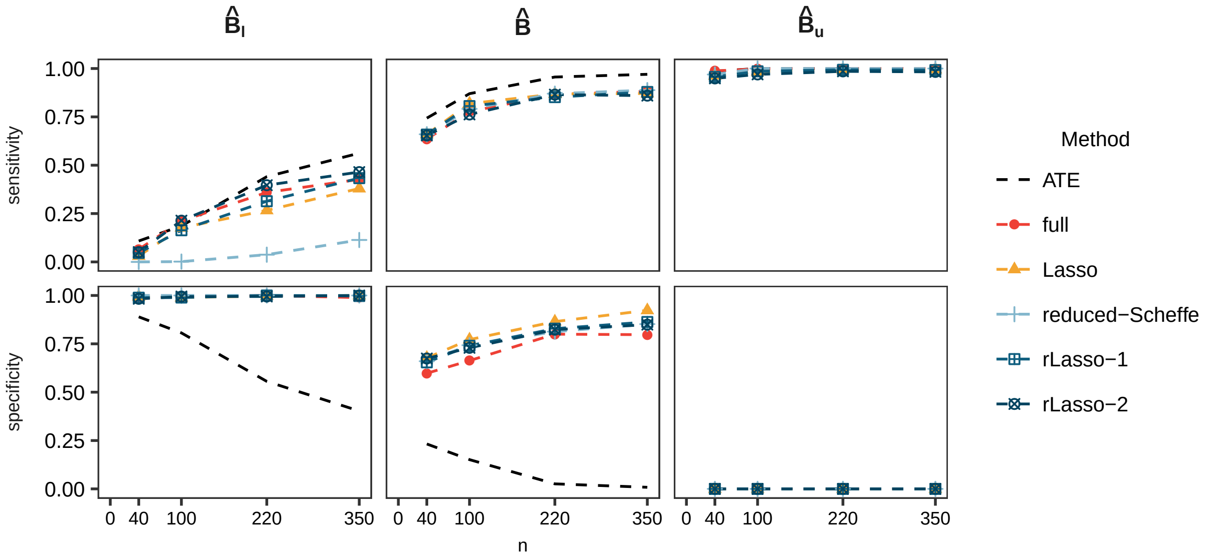

Figure 6: Sensitivity and specificity for identified subgroup by method and sample size for Case 4 and 10 biomarkers.

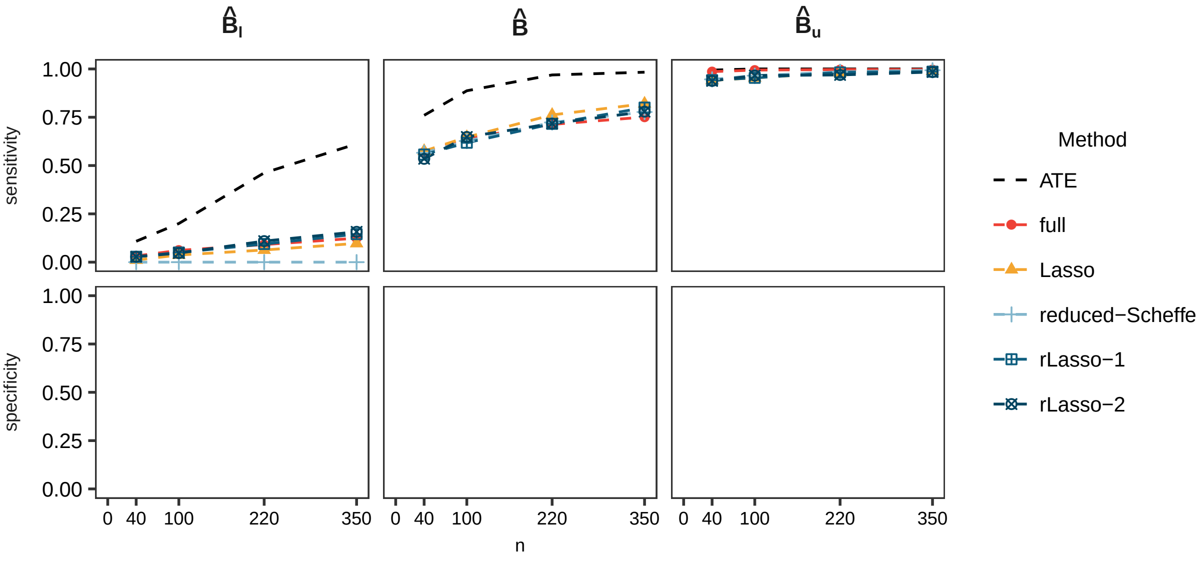

Figure 7: Sensitivity for identified subgroup by method and sample size for Case 5 and 10 biomarkers. Specificity is not shown as all patients have $D(X) > 0$

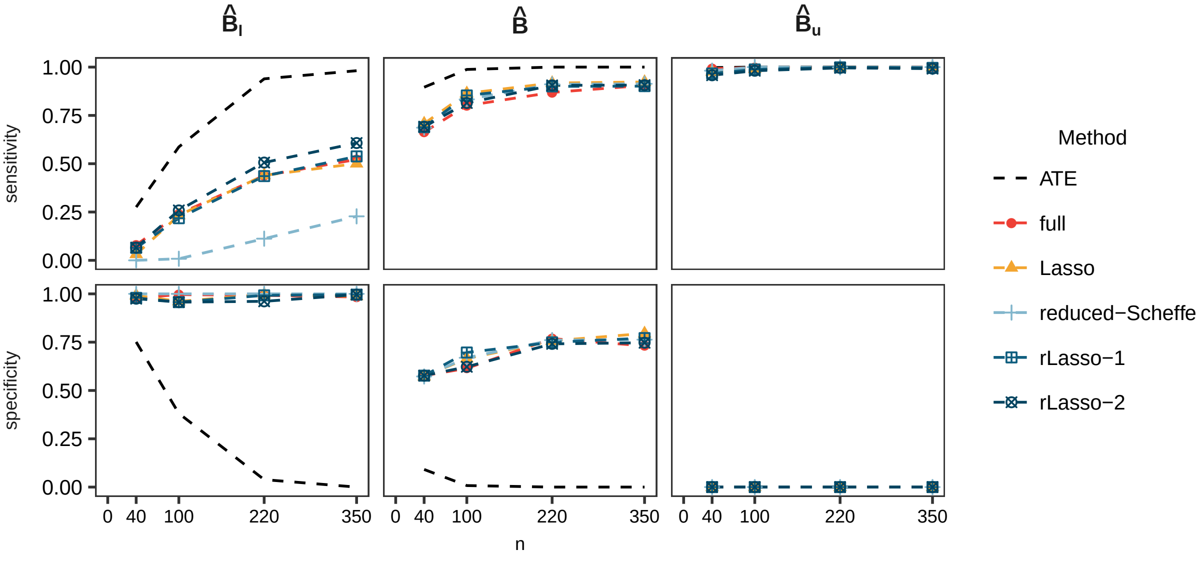

Figure 8: Sensitivity and specificity for identified subgroup by method and sample size for Case 6 and 10 biomarkers.

3 Results for 20 Biomarkers

3.1 Percent of selection

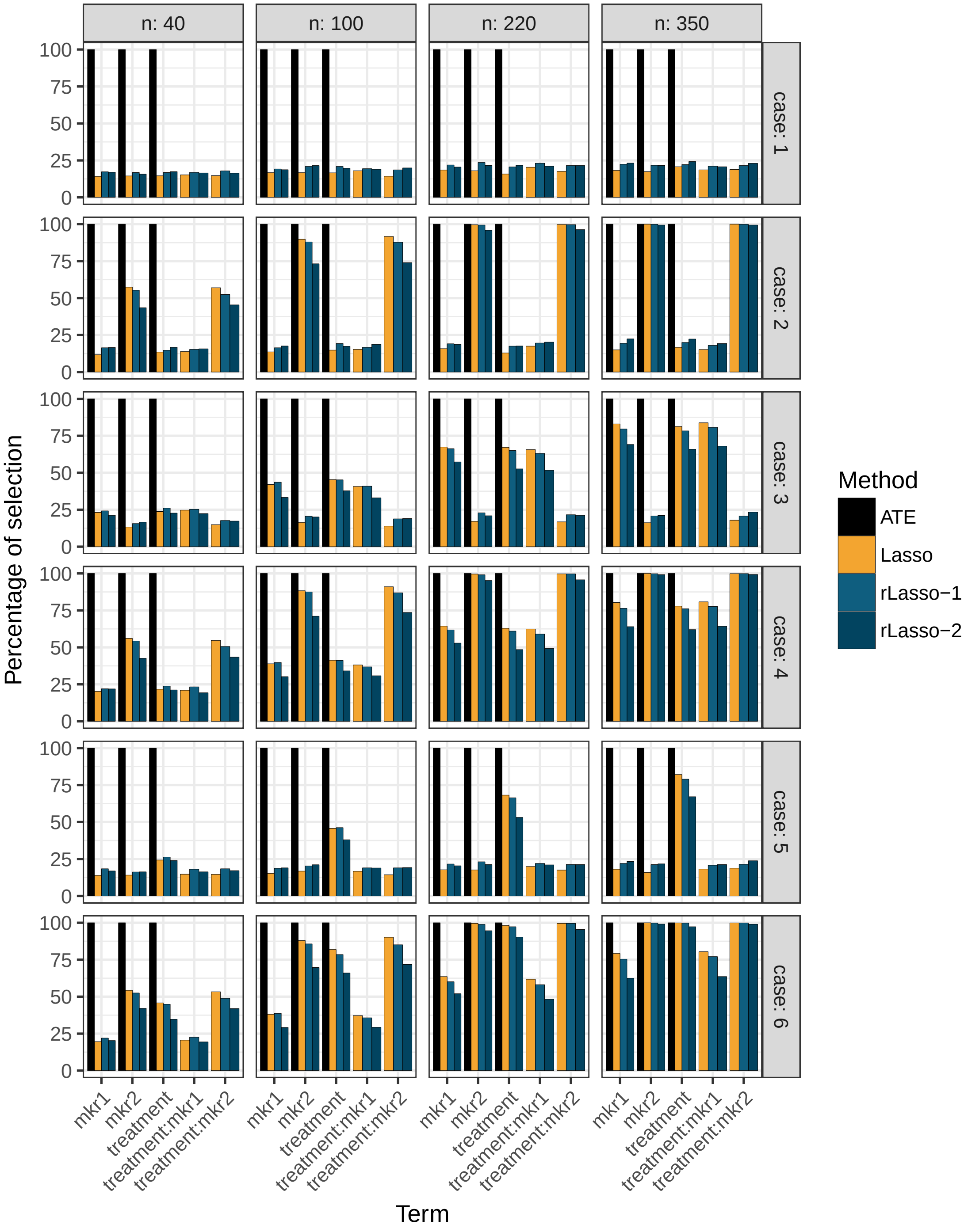

Figure 9: Selection probabilities for relevant terms in the model by method, case and sample size for 20 biomarkers. The full model is not represented here as all variables are included 100% of the times. The reduced model is not represented as it has the same values as the Lasso

3.2 Coverage of confidence intervals

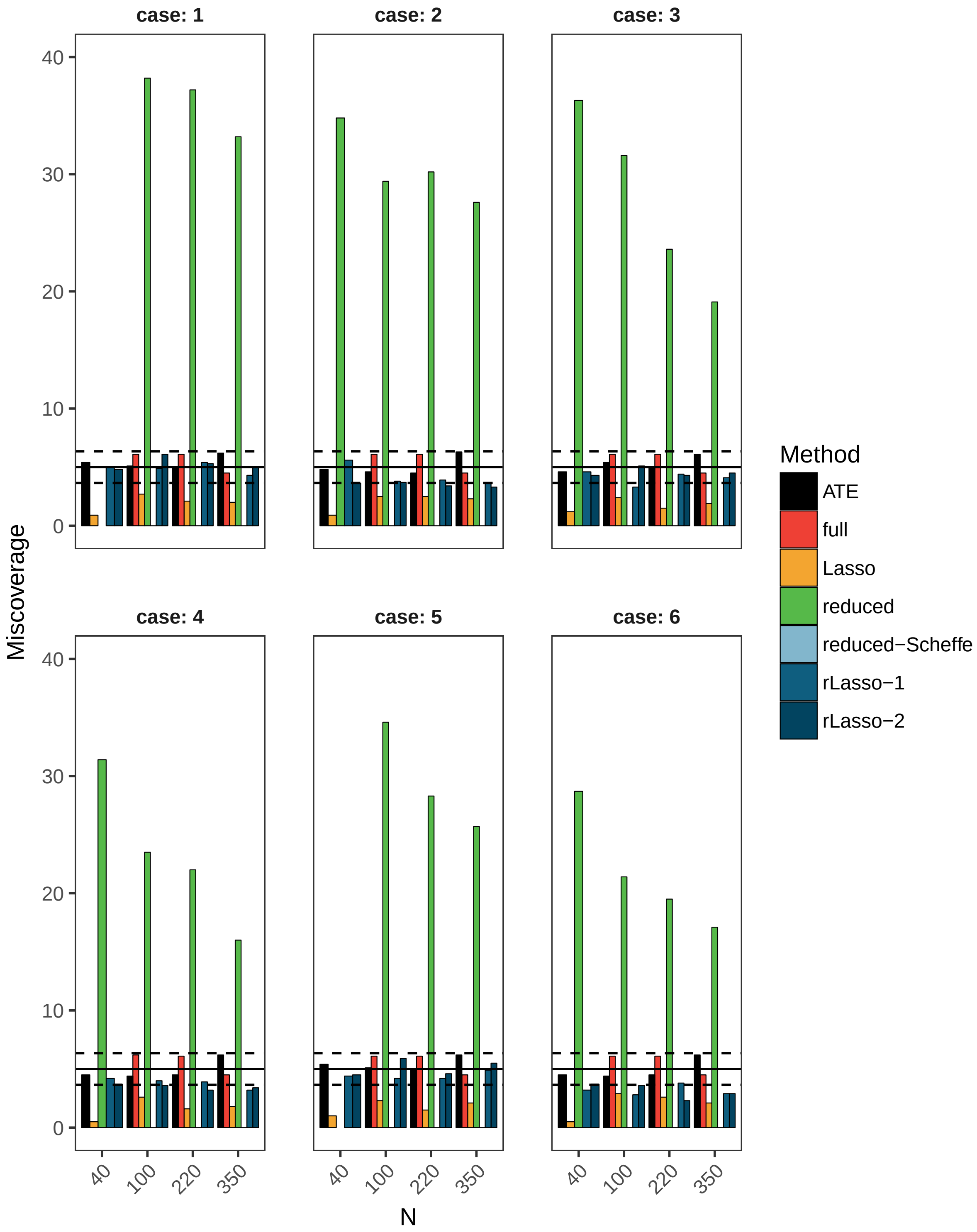

Figure 10: Average miscoverage of the confidence intervals for the PITE by method, case and sample size for 20 biomarkers. The line at 5% indicates the target miscoverage.

3.3 Bias, MSE and width

Table 8: Diagnostic measures for case 1 with 20 biomarkers. Columns 4 and 5 show the bias and the $\sqrt{MSE}$ for the point estimate of the PITE. The sixth column shows the median width of the confidence intervals for the PITE, and the last columns show the proportion of subjects in the identified subgroup when considering the using the limits of the confidence intervals and the point estimates. Since methods reduced and reduced-Scheffe have the same point estimate, bias and MSE are equal.

Case	N	Method	Bias	$\sqrt{MSE}$	Width	% in $\hat{B}_l$	% in $\hat{B}$	% in $\hat{B}_u$	% in B
1	40	ATE	0.00	0.47	1.88	2.8	50.8	97.4	0
1	40	Lasso	0.01	0.38	6.62	0.5	50.7	97.1	0
1	40	reduced	0.03	0.99	1.59	21.2	50.6	77.6	0
1	40	rLasso-1	0.01	0.96	4.13	2.8	50.3	96.1	0
1	40	rLasso-2	0.02	0.83	3.07	3.2	51.5	97.0	0
1	100	ATE	0.01	0.23	0.89	2.7	50.3	97.6	0
1	100	full	0.00	1.27	4.67	3.0	49.9	96.9	0
1	100	Lasso	0.00	0.27	4.15	1.4	47.7	98.1	0
1	100	reduced	-0.02	0.69	1.20	20.4	48.4	81.6	0
1	100	reduced-Scheffe	-0.02	0.69	5.30	0.0	48.4	99.4	0
1	100	rLasso-1	-0.02	0.68	2.66	2.5	48.9	97.2	0
1	100	rLasso-2	-0.02	0.61	2.10	2.3	49.1	95.4	0
1	220	ATE	0.00	0.14	0.56	2.3	49.1	97.3	0
1	220	full	0.00	0.71	2.67	3.0	49.2	96.9	0
1	220	Lasso	0.00	0.19	2.91	0.9	49.8	97.2	0
1	220	reduced	-0.01	0.50	0.90	18.2	48.2	79.4	0
1	220	reduced-Scheffe	-0.01	0.50	3.66	0.0	48.2	98.4	0
1	220	rLasso-1	-0.01	0.47	1.87	3.5	49.1	97.6	0
1	220	rLasso-2	-0.01	0.43	1.40	2.5	51.0	96.6	0
1	350	ATE	0.00	0.11	0.43	2.9	50.6	96.7	0
1	350	full	0.00	0.51	2.04	1.9	49.0	97.4	0
1	350	Lasso	0.00	0.15	2.40	1.1	51.5	97.6	0
1	350	reduced	0.01	0.38	0.75	17.1	50.1	82.4	0
1	350	reduced-Scheffe	0.01	0.38	2.97	0.0	50.1	98.5	0
1	350	rLasso-1	0.01	0.38	1.49	2.4	49.4	97.9	0
1	350	rLasso-2	0.01	0.35	1.15	3.1	49.2	97.7	0

Table 9: Diagnostic measures for case 2 with 20 biomarkers. Columns 4 and 5 show the bias and the $\sqrt{MSE}$ for the point estimate of the PITE. The sixth column shows the median width of the confidence intervals for the PITE, and the last columns show the proportion of subjects in the identified subgroup when considering the using the limits of the confidence intervals and the point estimates. Since methods reduced and reduced-Scheffe have the same point estimate, bias and MSE are equal.

Case	N	Method	Bias	$\sqrt{MSE}$	Width	% in $\hat{B}_l$	% in $\hat{B}$	% in $\hat{B}_u$	% in B
2	40	ATE	-0.05	0.76	1.95	1.90	50.9	97.10	54.6
2	40	Lasso	-0.03	0.59	7.06	1.60	51.7	97.50	54.6
2	40	reduced	0.02	1.04	1.69	22.90	52.8	80.50	54.6
2	40	rLasso-1	-0.01	1.01	4.52	4.50	50.7	95.40	54.6
2	40	rLasso-2	0.04	0.94	3.46	4.50	56.0	97.30	54.6
2	100	ATE	-0.02	0.61	0.92	2.80	50.6	98.20	51.1
2	100	full	0.00	1.27	4.67	4.30	50.2	96.20	51.1
2	100	Lasso	-0.02	0.40	3.62	6.20	52.9	94.50	51.1
2	100	reduced	-0.03	0.71	1.36	25.20	51.9	75.20	51.1
2	100	reduced-Scheffe	-0.03	0.71	5.92	0.00	51.9	99.60	51.1
2	100	rLasso-1	-0.03	0.69	2.90	6.21	51.4	94.09	51.1
2	100	rLasso-2	-0.04	0.68	2.28	7.30	50.2	92.40	51.1
2	220	ATE	0.00	0.60	0.58	2.30	49.8	97.80	50.1
2	220	full	0.00	0.71	2.67	6.70	49.7	92.50	50.1
2	220	Lasso	0.01	0.28	2.48	11.20	48.1	89.70	50.1
2	220	reduced	0.00	0.48	0.97	30.80	48.2	69.40	50.1
2	220	reduced-Scheffe	0.00	0.48	3.93	0.00	48.2	100.00	50.1
2	220	rLasso-1	0.00	0.47	1.97	14.21	48.9	86.69	50.1
2	220	rLasso-2	0.00	0.43	1.58	17.20	49.3	84.30	50.1
2	350	ATE	-0.05	0.59	0.45	3.20	49.4	96.90	53.5
2	350	full	-0.01	0.51	2.05	10.80	51.5	91.80	53.5
2	350	Lasso	-0.01	0.22	1.96	16.30	53.0	87.00	53.5
2	350	reduced	0.01	0.37	0.76	36.80	54.8	69.50	53.5
2	350	reduced-Scheffe	0.01	0.37	3.04	0.00	54.8	100.00	53.5
2	350	rLasso-1	0.02	0.37	1.55	18.50	53.9	85.00	53.5
2	350	rLasso-2	0.00	0.35	1.25	23.00	53.3	82.10	53.5

Table 10: Diagnostic measures for case 3 with 20 biomarkers. Columns 4 and 5 show the bias and the $\sqrt{MSE}$ for the point estimate of the PITE. The sixth column shows the median width of the confidence intervals for the PITE, and the last columns show the proportion of subjects in the identified subgroup when considering the using the limits of the confidence intervals and the point estimates. Since methods reduced and reduced-Scheffe have the same point estimate, bias and MSE are equal.

Case	N	Method	Bias	$\sqrt{MSE}$	Width	% in $\hat{B}_l$	% in $\hat{B}$	% in $\hat{B}_u$	% in B
3	40	ATE	0.01	0.54	1.90	7.60	71.2	99.2	48.7
3	40	Lasso	-0.20	0.49	7.02	0.60	55.1	98.1	48.7
3	40	reduced	-0.13	1.01	1.65	24.10	54.6	81.6	48.7
3	40	rLasso-1	-0.12	1.00	4.29	2.90	55.0	97.4	48.7
3	40	rLasso-2	-0.12	0.88	3.25	3.70	56.0	97.1	48.7
3	100	ATE	0.00	0.33	0.89	20.50	86.6	99.9	51.1
3	100	full	0.00	1.27	4.68	4.20	58.2	98.0	51.1
3	100	Lasso	-0.19	0.40	4.29	2.50	60.2	97.9	51.1
3	100	reduced	-0.08	0.75	1.30	27.90	59.0	86.1	51.1
3	100	reduced-Scheffe	-0.08	0.75	5.71	0.00	59.0	99.2	51.1
3	100	rLasso-1	-0.10	0.74	2.91	4.20	59.6	97.0	51.1
3	100	rLasso-2	-0.13	0.68	2.22	4.80	58.9	96.3	51.1
3	220	ATE	-0.01	0.29	0.56	40.60	95.3	100.0	51.3
3	220	full	0.00	0.71	2.67	8.70	62.4	98.5	51.3
3	220	Lasso	-0.16	0.31	3.06	5.10	65.1	98.9	51.3
3	220	reduced	-0.05	0.52	1.04	30.30	64.7	90.6	51.3
3	220	reduced-Scheffe	-0.05	0.52	4.25	0.00	64.7	99.7	51.3
3	220	rLasso-1	-0.06	0.52	2.00	8.41	65.6	98.2	51.3
3	220	rLasso-2	-0.09	0.49	1.52	9.60	63.5	96.9	51.3
3	350	ATE	-0.01	0.27	0.44	61.30	98.8	100.0	52.0
3	350	full	-0.01	0.51	2.05	8.50	67.9	99.0	52.0
3	350	Lasso	-0.13	0.26	2.32	8.80	72.6	98.7	52.0
3	350	reduced	-0.01	0.40	0.86	38.30	70.3	92.5	52.0
3	350	reduced-Scheffe	-0.01	0.40	3.41	0.00	70.3	99.9	52.0
3	350	rLasso-1	-0.02	0.40	1.58	12.60	70.6	97.8	52.0
3	350	rLasso-2	-0.06	0.40	1.25	16.20	66.6	96.9	52.0

Table 11: Diagnostic measures for case 4 with 20 biomarkers. Columns 4 and 5 show the bias and the $\sqrt{MSE}$ for the point estimate of the PITE. The sixth column shows the median width of the confidence intervals for the PITE, and the last columns show the proportion of subjects in the identified subgroup when considering the using the limits of the confidence intervals and the point estimates. Since methods reduced and reduced-Scheffe have the same point estimate, bias and MSE are equal.

Case	N	Method	Bias	$\sqrt{MSE}$	Width	% in $\hat{B}_l$	% in $\hat{B}$	% in $\hat{B}_u$	% in B
4	40	ATE	-0.03	0.82	1.97	7.2	70.3	98.6	65.5
4	40	Lasso	-0.22	0.67	7.42	1.9	55.0	98.1	65.5
4	40	reduced	-0.13	1.06	1.76	26.3	54.0	82.8	65.5
4	40	rLasso-1	-0.17	1.07	4.65	4.5	52.7	97.0	65.5
4	40	rLasso-2	-0.12	1.00	3.59	4.9	57.8	97.4	65.5
4	100	ATE	-0.02	0.67	0.93	18.7	85.8	99.9	63.9
4	100	full	0.00	1.27	4.67	6.9	58.6	96.9	63.9
4	100	Lasso	-0.22	0.53	4.05	6.6	57.1	95.6	63.9
4	100	reduced	-0.11	0.77	1.43	31.4	59.2	82.7	63.9
4	100	reduced-Scheffe	-0.11	0.77	6.19	0.0	59.2	99.7	63.9
4	100	rLasso-1	-0.14	0.79	3.05	7.2	58.0	94.9	63.9
4	100	rLasso-2	-0.18	0.75	2.40	8.5	56.7	93.8	63.9
4	220	ATE	-0.01	0.65	0.59	37.3	95.8	100.0	62.9
4	220	full	0.00	0.71	2.67	14.3	60.4	95.3	62.9
4	220	Lasso	-0.16	0.37	2.70	13.6	57.2	93.7	62.9
4	220	reduced	-0.05	0.53	1.09	36.4	59.7	80.3	62.9
4	220	reduced-Scheffe	-0.05	0.53	4.42	0.0	59.7	100.0	62.9
4	220	rLasso-1	-0.06	0.52	2.13	17.3	58.8	92.3	62.9
4	220	rLasso-2	-0.10	0.50	1.67	19.2	57.1	88.2	62.9
4	350	ATE	-0.06	0.64	0.45	58.2	98.1	100.0	65.8
4	350	full	-0.01	0.51	2.05	18.8	63.8	95.8	65.8
4	350	Lasso	-0.15	0.33	2.10	19.9	62.6	93.1	65.8
4	350	reduced	-0.01	0.40	0.90	43.7	65.3	84.4	65.8
4	350	reduced-Scheffe	-0.01	0.40	3.57	0.4	65.3	100.0	65.8
4	350	rLasso-1	-0.03	0.39	1.66	23.4	64.8	90.9	65.8
4	350	rLasso-2	-0.08	0.41	1.34	29.1	62.7	88.8	65.8

Table 12: Diagnostic measures for case 5 with 20 biomarkers. Columns 4 and 5 show the bias and the $\sqrt{MSE}$ for the point estimate of the PITE. The sixth column shows the median width of the confidence intervals for the PITE, and the last columns show the proportion of subjects in the identified subgroup when considering the using the limits of the confidence intervals and the point estimates. Since methods reduced and reduced-Scheffe have the same point estimate, bias and MSE are equal.

Case	N	Method	Bias	$\sqrt{MSE}$	Width	% in $\hat{B}_l$	% in $\hat{B}$	% in $\hat{B}_u$	% in B
5	40	ATE	0.00	0.47	1.88	7.20	71.4	99.4	100
5	40	Lasso	-0.20	0.44	6.44	1.00	55.3	98.1	100
5	40	reduced	-0.11	1.00	1.61	25.70	56.4	82.1	100
5	40	rLasso-1	-0.14	0.99	4.22	3.00	55.7	96.9	100
5	40	rLasso-2	-0.15	0.86	3.17	3.60	54.9	96.7	100
5	100	ATE	0.01	0.23	0.89	20.50	87.4	100.0	100
5	100	full	0.00	1.27	4.68	4.20	57.8	98.0	100
5	100	Lasso	-0.18	0.34	4.13	2.30	61.3	97.9	100
5	100	reduced	-0.08	0.72	1.27	27.90	60.0	87.9	100
5	100	reduced-Scheffe	-0.08	0.72	5.64	0.00	60.0	99.1	100
5	100	rLasso-1	-0.09	0.71	2.72	3.10	61.3	98.0	100
5	100	rLasso-2	-0.13	0.66	2.13	5.60	57.4	96.8	100
5	220	ATE	0.00	0.14	0.56	41.70	95.8	100.0	100
5	220	full	0.00	0.71	2.67	6.40	62.9	98.5	100
5	220	Lasso	-0.15	0.26	3.03	3.40	69.6	99.5	100
5	220	reduced	-0.04	0.52	0.98	32.80	66.2	92.8	100
5	220	reduced-Scheffe	-0.04	0.52	4.01	0.00	66.2	99.8	100
5	220	rLasso-1	-0.05	0.49	1.93	6.90	67.5	99.0	100
5	220	rLasso-2	-0.09	0.47	1.47	7.50	65.2	97.9	100
5	350	ATE	0.00	0.11	0.43	61.90	99.0	100.0	100
5	350	full	-0.01	0.51	2.05	6.00	69.3	99.4	100
5	350	Lasso	-0.12	0.21	2.40	5.70	79.9	99.7	100
5	350	reduced	-0.01	0.39	0.81	37.80	74.8	96.1	100
5	350	reduced-Scheffe	-0.01	0.39	3.22	0.00	74.8	99.9	100
5	350	rLasso-1	-0.01	0.39	1.52	9.21	75.5	98.7	100
5	350	rLasso-2	-0.05	0.38	1.19	12.70	72.1	98.6	100

Table 13: Diagnostic measures for case 6 with 20 biomarkers. Columns 4 and 5 show the bias and the $\sqrt{MSE}$ for the point estimate of the PITE. The sixth column shows the median width of the confidence intervals for the PITE, and the last columns show the proportion of subjects in the identified subgroup when considering the using the limits of the confidence intervals and the point estimates. Since methods reduced and reduced-Scheffe have the same point estimate, bias and MSE are equal.

Case	N	Method	Bias	$\sqrt{MSE}$	Width	% in $\hat{B}_l$	% in $\hat{B}$	% in $\hat{B}_u$	% in B
6	40	ATE	-0.03	0.82	1.97	17.00	86.0	99.8	76.9
6	40	Lasso	-0.39	0.75	8.42	2.60	63.1	98.6	76.9
6	40	reduced	-0.22	1.09	1.86	29.90	61.6	86.3	76.9
6	40	rLasso-1	-0.25	1.10	4.99	4.80	61.5	98.4	76.9
6	40	rLasso-2	-0.25	1.07	3.67	5.81	62.4	97.5	76.9
6	100	ATE	-0.02	0.67	0.93	58.80	98.3	100.0	77.4
6	100	full	0.00	1.27	4.67	10.50	65.4	98.0	77.4
6	100	Lasso	-0.31	0.58	3.70	13.50	71.4	98.4	77.4
6	100	reduced	-0.09	0.78	1.48	41.20	68.7	90.0	77.4
6	100	reduced-Scheffe	-0.09	0.78	6.36	0.00	68.7	100.0	77.4
6	100	rLasso-1	-0.12	0.80	3.13	14.80	67.9	97.6	77.4
6	100	rLasso-2	-0.18	0.78	2.49	14.50	67.7	95.6	77.4
6	220	ATE	-0.01	0.65	0.59	92.30	100.0	100.0	75.1
6	220	full	0.00	0.71	2.67	20.80	70.0	97.3	75.1
6	220	Lasso	-0.19	0.40	2.49	22.90	73.5	97.4	75.1
6	220	reduced	-0.02	0.51	1.13	47.60	71.9	90.4	75.1
6	220	reduced-Scheffe	-0.02	0.51	4.57	0.20	71.9	100.0	75.1
6	220	rLasso-1	-0.02	0.51	2.13	28.03	70.8	97.6	75.1
6	220	rLasso-2	-0.05	0.49	1.71	32.80	71.8	96.1	75.1
6	350	ATE	-0.06	0.64	0.45	98.90	100.0	100.0	79.0
6	350	full	-0.01	0.51	2.05	29.80	75.0	97.6	79.0
6	350	Lasso	-0.17	0.34	1.90	33.00	77.3	97.7	79.0
6	350	reduced	0.00	0.39	0.90	57.20	77.0	92.0	79.0
6	350	reduced-Scheffe	0.00	0.39	3.59	3.10	77.0	100.0	79.0
6	350	rLasso-1	0.00	0.38	1.65	36.90	77.7	97.2	79.0
6	350	rLasso-2	-0.02	0.40	1.37	44.30	76.6	96.5	79.0

3.4 Sensitivity and Specificity

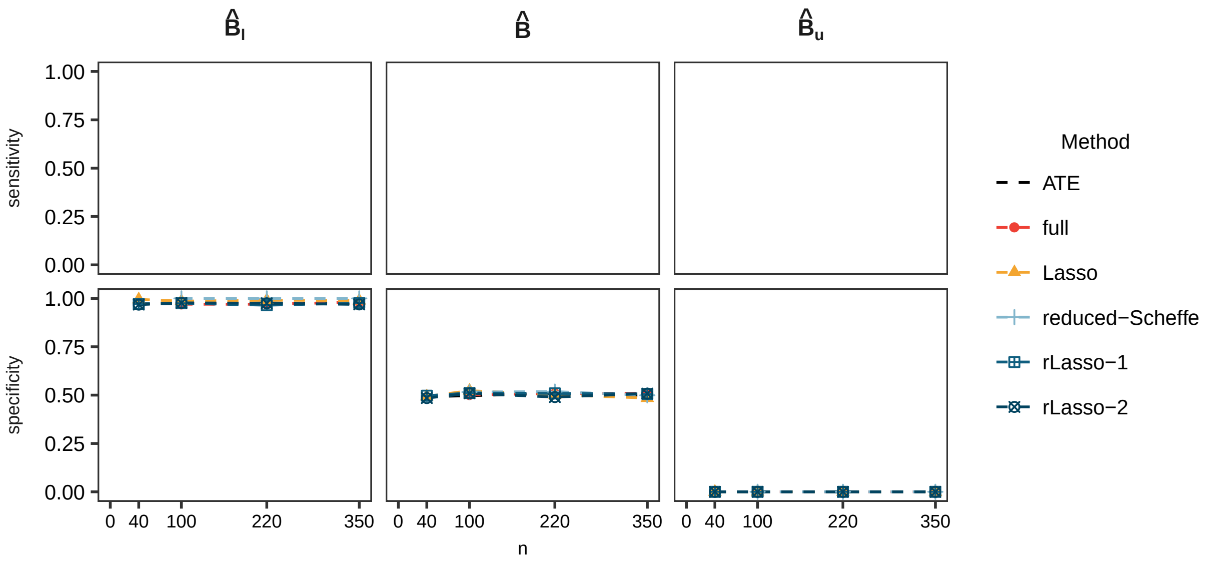

Figure 11: Specificity for identified subgroup by method and sample size for Case 1 and 20 biomarkers. Sensitivity is not shown as there are no patients with $D(X) > 0$

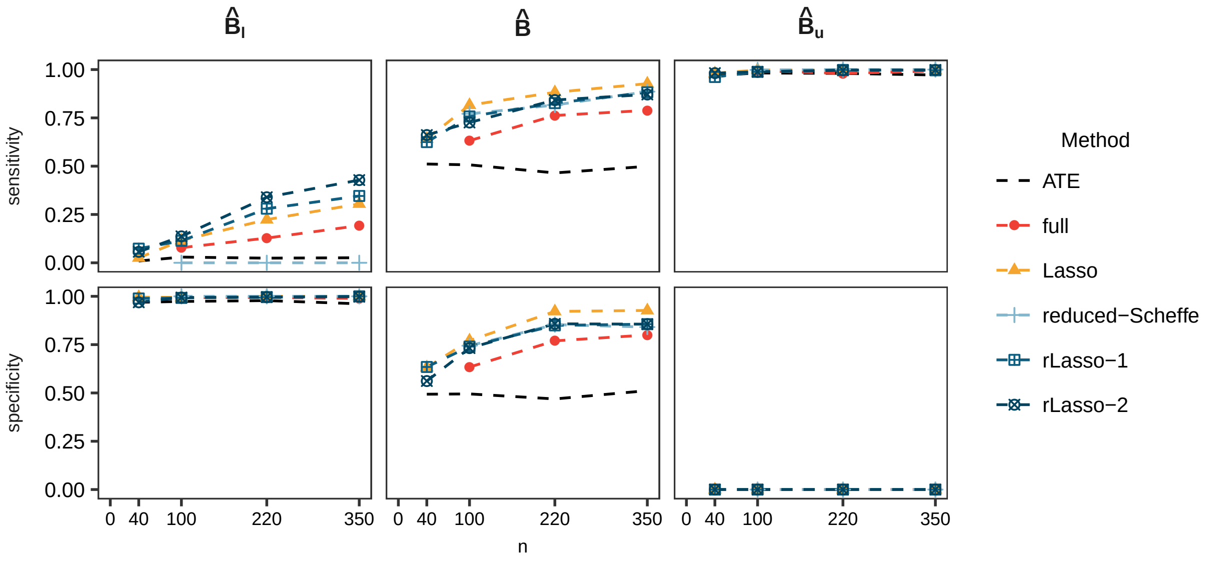

Figure 12: Sensitivity and specificity for identified subgroup by method and sample size for Case 2 and 20 biomarkers.

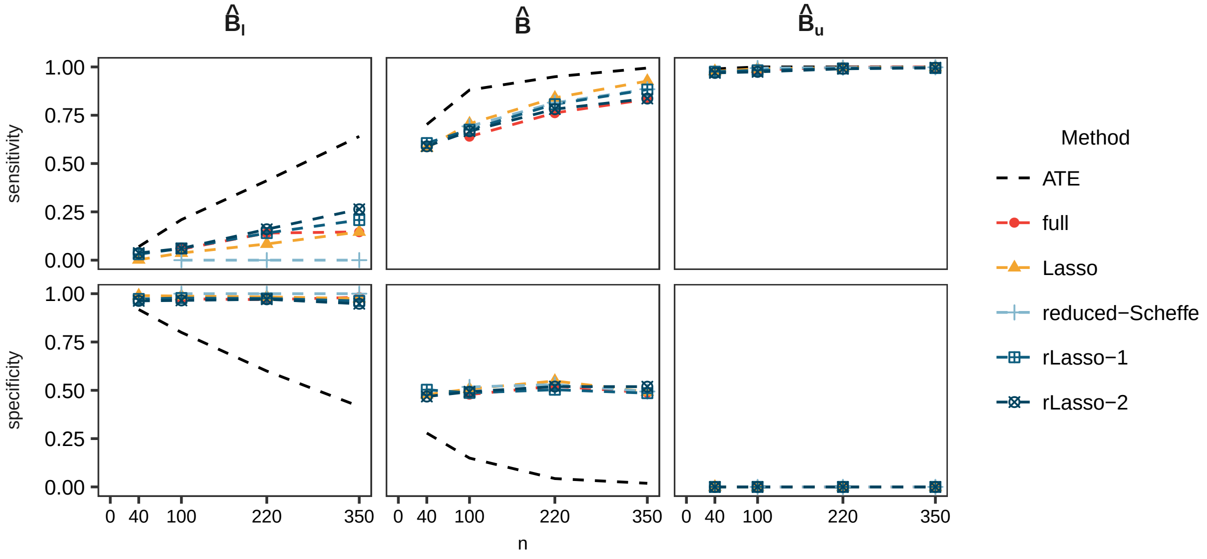

Figure 13: Sensitivity and specificity for identified subgroup by method and sample size for Case 3 and 20 biomarkers.

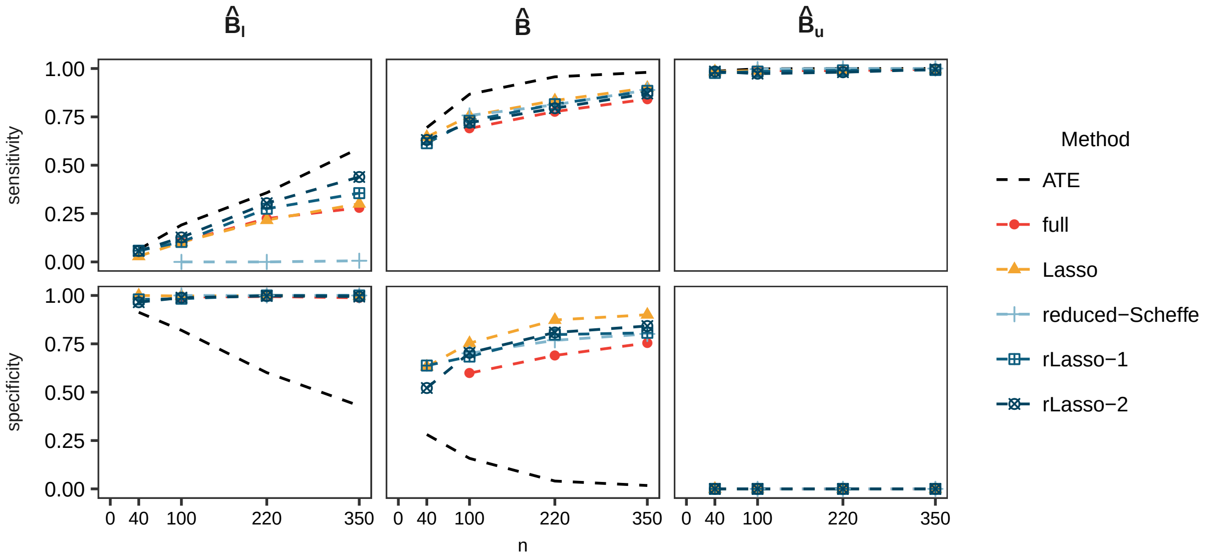

Figure 14: Sensitivity and specificity for identified subgroup by method and sample size for Case 4 and 20 biomarkers.

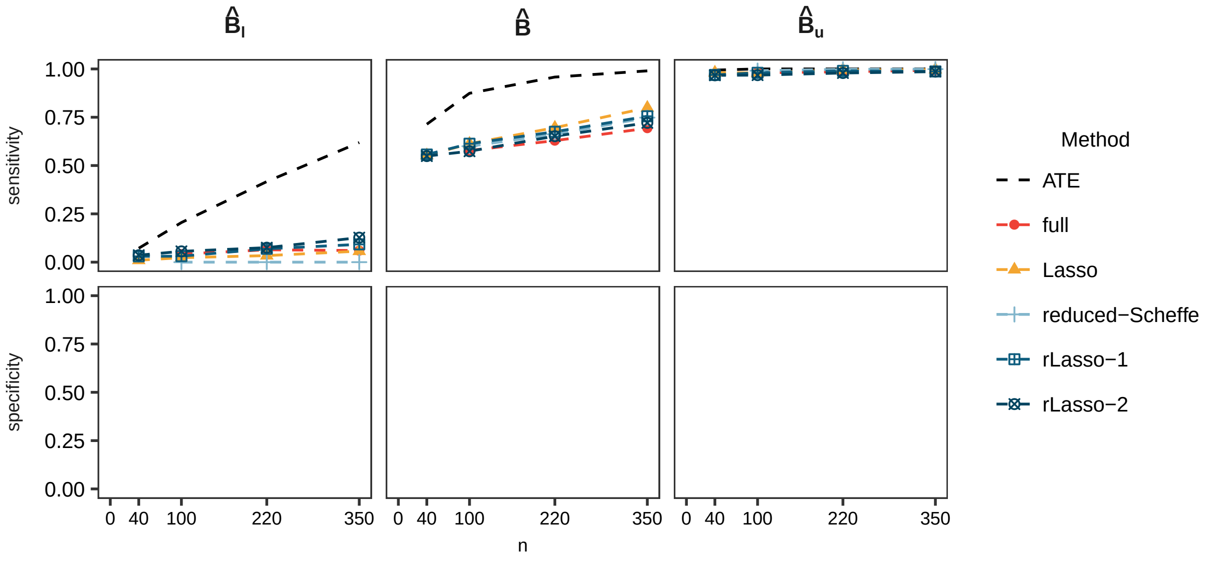

Figure 15: Sensitivity for identified subgroup by method and sample size for Case 5 and 20 biomarkers. Specificity is not shown as all patients have $D(X) > 0$

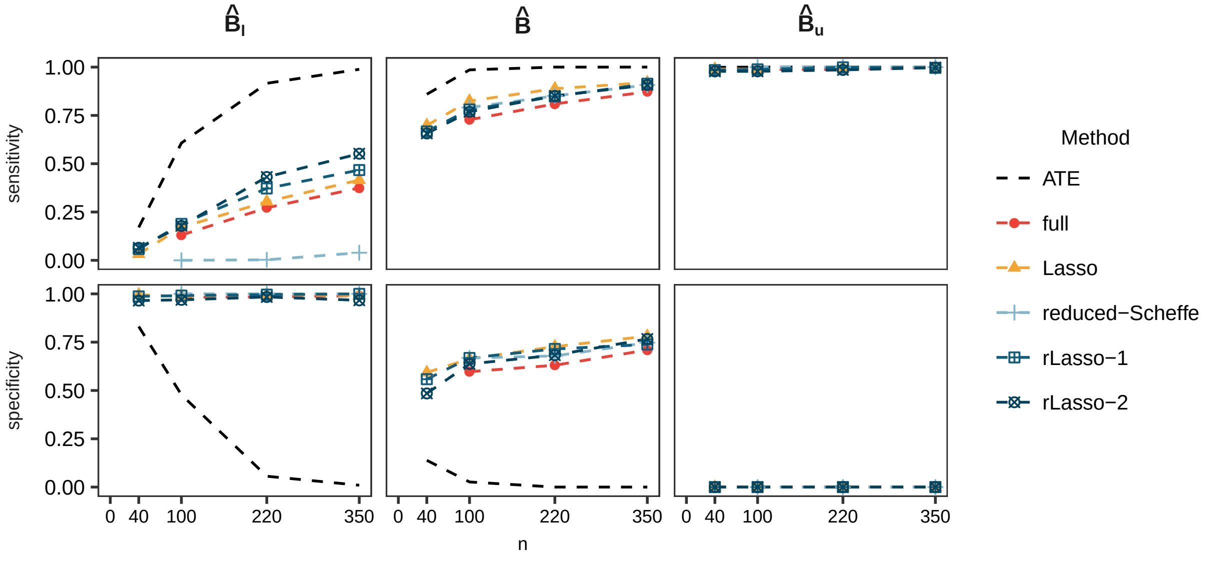

Figure 16: Sensitivity and specificity for identified subgroup by method and sample size for Case 6 and 20 biomarkers.

4 Results for 50 Biomarkers

4.1 Percent of selection

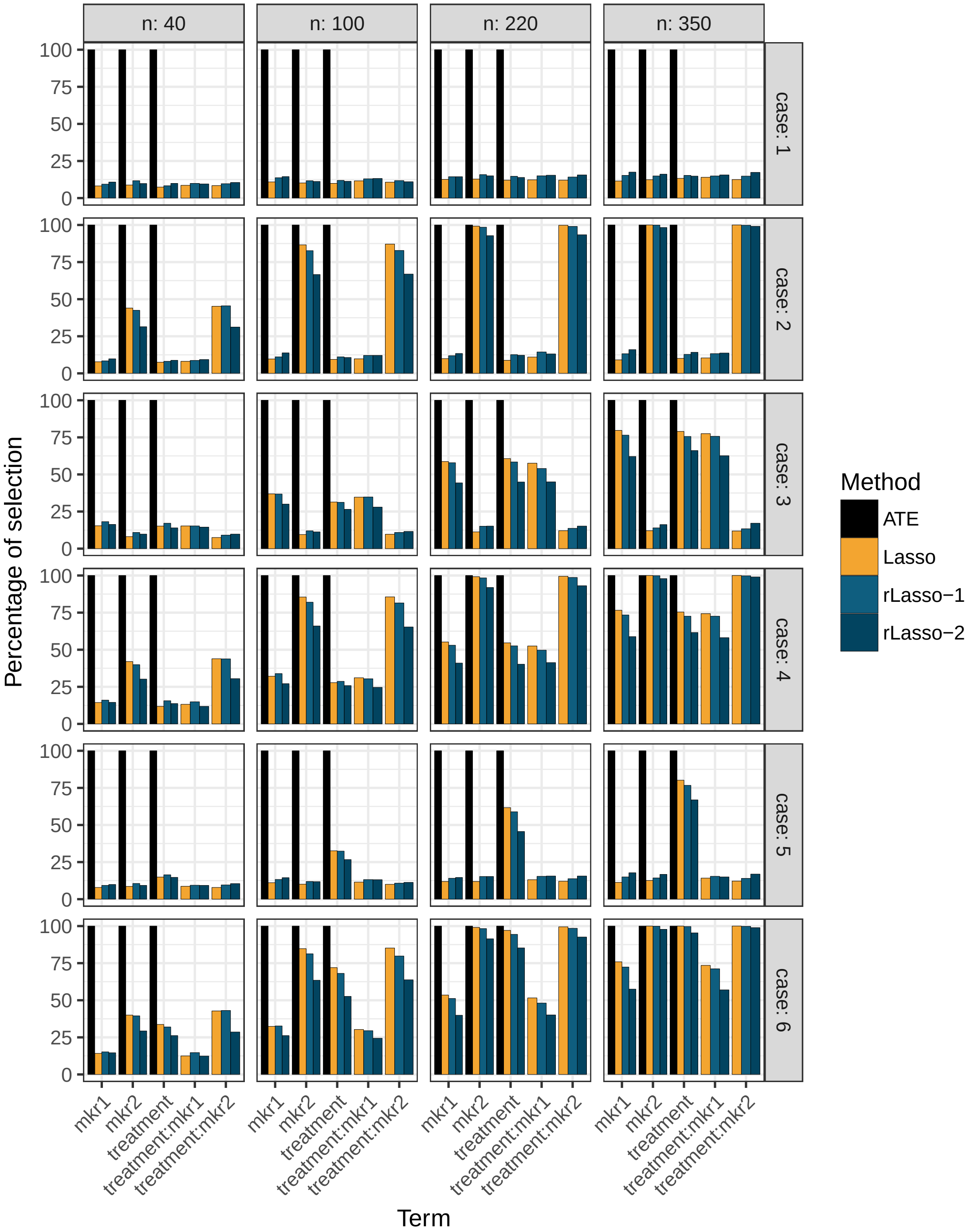

Figure 17: Selection probabilities for relevant terms in the model by method, case and sample size for 50 biomarkers. The full model is not represented here as all variables are included 100% of the times. The reduced model is not represented as it has the same values as the Lasso

4.2 Coverage of confidence intervals

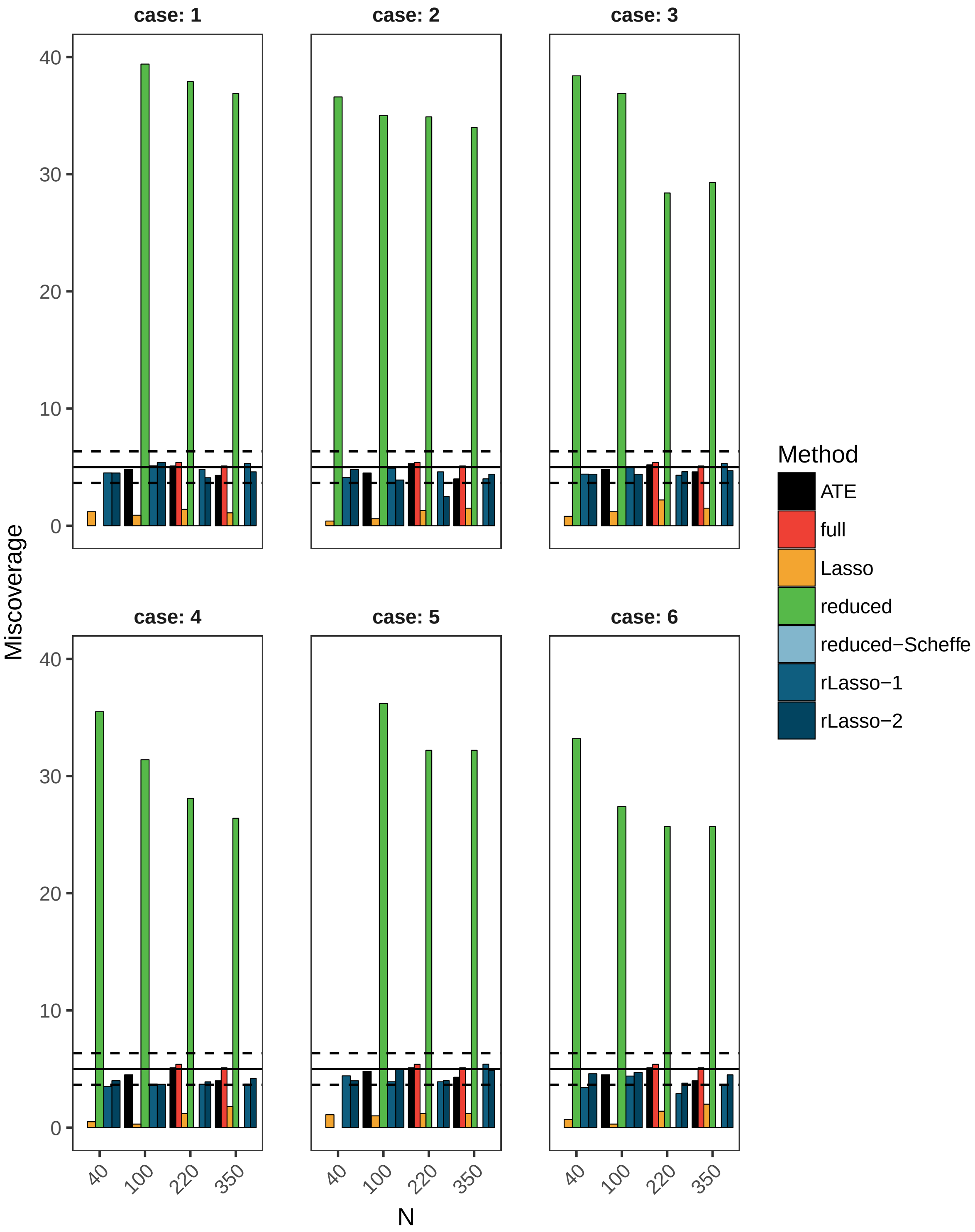

Figure 18: Average miscoverage of the confidence intervals for the PITE by method, case and sample size for 50 biomarkers. The line at 5% indicates the target miscoverage.

4.3 Bias, MSE and width

Table 14: Diagnostic measures for case 1 with 50 biomarkers. Columns 4 and 5 show the bias and the $\sqrt{MSE}$ for the point estimate of the PITE. The sixth column shows the median width of the confidence intervals for the PITE, and the last columns show the proportion of subjects in the identified subgroup when considering the using the limits of the confidence intervals and the point estimates. Since methods reduced and reduced-Scheffe have the same point estimate, bias and MSE are equal.

Case	N	Method	Bias	$\sqrt{MSE}$	Width	% in $\hat{B}_l$	% in $\hat{B}$	% in $\hat{B}_u$	% in B
1	40	ATE							0
1	40	Lasso	0.00	0.38	10.00	0.60	49.3	98.70	0
1	40	reduced	-0.02	1.02	1.69	20.60	49.7	77.50	0
1	40	rLasso-1	-0.03	0.99	5.86	1.80	49.1	97.10	0
1	40	rLasso-2	-0.04	0.93	4.16	1.80	45.4	96.80	0
1	100	ATE	-0.01	0.28	1.14	2.00	48.5	97.20	0
1	100	Lasso	0.00	0.30	6.49	0.40	48.3	99.40	0
1	100	reduced	-0.02	0.86	1.50	19.10	48.0	79.60	0
1	100	rLasso-1	0.00	0.82	4.00	2.80	51.1	97.70	0
1	100	rLasso-2	-0.03	0.75	2.90	3.00	49.4	97.60	0
1	220	ATE	0.00	0.15	0.60	2.50	51.5	97.40	0
1	220	full	0.05	1.34	5.25	3.00	52.1	97.60	0
1	220	Lasso	0.01	0.22	4.26	0.80	50.2	99.40	0
1	220	reduced	0.02	0.63	1.15	20.10	51.4	82.20	0
1	220	reduced-Scheffe	0.02	0.63	7.50	0.00	51.4	100.00	0
1	220	rLasso-1	0.02	0.65	2.71	2.61	50.3	97.79	0
1	220	rLasso-2	0.02	0.55	2.06	1.90	50.5	97.80	0
1	350	ATE	0.01	0.11	0.45	2.70	53.6	98.40	0
1	350	full	0.02	0.89	3.53	3.10	49.4	98.00	0
1	350	Lasso	0.01	0.18	3.43	0.80	51.2	99.60	0
1	350	reduced	0.02	0.53	0.97	19.60	51.7	82.60	0
1	350	reduced-Scheffe	0.02	0.53	5.97	0.00	51.7	99.90	0
1	350	rLasso-1	0.03	0.53	2.18	2.51	52.5	97.19	0
1	350	rLasso-2	0.02	0.45	1.65	2.20	53.4	97.60	0

Table 15: Diagnostic measures for case 2 with 50 biomarkers. Columns 4 and 5 show the bias and the $\sqrt{MSE}$ for the point estimate of the PITE. The sixth column shows the median width of the confidence intervals for the PITE, and the last columns show the proportion of subjects in the identified subgroup when considering the using the limits of the confidence intervals and the point estimates. Since methods reduced and reduced-Scheffe have the same point estimate, bias and MSE are equal.

Case	N	Method	Bias	$\sqrt{MSE}$	Width	% in $\hat{B}_l$	% in $\hat{B}$	% in $\hat{B}_u$	% in B
2	40	ATE							52.0
2	40	Lasso	-0.01	0.59	10.28	0.80	51.1	99.10	52.0
2	40	reduced	0.01	1.02	1.79	22.30	51.1	79.60	52.0
2	40	rLasso-1	-0.02	1.06	6.47	2.11	50.2	96.79	52.0
2	40	rLasso-2	-0.01	1.07	4.44	2.80	49.9	97.00	52.0
2	100	ATE	0.01	0.65	1.19	1.70	48.9	97.20	49.9
2	100	Lasso	0.00	0.46	6.74	1.60	49.1	98.00	49.9
2	100	reduced	-0.01	0.84	1.53	22.20	49.2	78.00	49.9
2	100	rLasso-1	-0.03	0.86	4.25	3.81	49.6	95.09	49.9
2	100	rLasso-2	-0.03	0.80	3.15	4.30	48.3	95.40	49.9
2	220	ATE	0.00	0.60	0.63	2.70	49.5	97.40	51.4
2	220	full	0.05	1.34	5.25	4.20	50.2	96.60	51.4
2	220	Lasso	0.01	0.32	3.71	4.90	49.4	95.90	51.4
2	220	reduced	0.01	0.61	1.16	24.90	51.2	74.80	51.4
2	220	reduced-Scheffe	0.01	0.61	7.44	0.00	51.2	100.00	51.4
2	220	rLasso-1	0.01	0.63	2.84	8.20	49.7	92.90	51.4
2	220	rLasso-2	0.02	0.54	2.13	9.50	50.7	90.50	51.4
2	350	ATE	0.02	0.58	0.47	2.20	53.4	98.20	48.5
2	350	full	0.02	0.89	3.53	5.20	50.5	95.30	48.5
2	350	Lasso	0.01	0.26	2.86	6.90	50.3	93.20	48.5
2	350	reduced	0.02	0.51	0.96	29.70	51.3	71.50	48.5
2	350	reduced-Scheffe	0.02	0.51	5.89	0.00	51.3	100.00	48.5
2	350	rLasso-1	0.02	0.51	2.19	9.31	51.0	90.19	48.5
2	350	rLasso-2	0.01	0.45	1.71	12.80	49.1	87.50	48.5

Table 16: Diagnostic measures for case 3 with 50 biomarkers. Columns 4 and 5 show the bias and the $\sqrt{MSE}$ for the point estimate of the PITE. The sixth column shows the median width of the confidence intervals for the PITE, and the last columns show the proportion of subjects in the identified subgroup when considering the using the limits of the confidence intervals and the point estimates. Since methods reduced and reduced-Scheffe have the same point estimate, bias and MSE are equal.

Case	N	Method	Bias	$\sqrt{MSE}$	Width	% in $\hat{B}_l$	% in $\hat{B}$	% in $\hat{B}_u$	% in B
3	40	ATE							50.2
3	40	Lasso	-0.22	0.49	10.15	1.00	51.9	99.20	50.2
3	40	reduced	-0.18	1.05	1.73	21.80	52.7	81.00	50.2
3	40	rLasso-1	-0.21	1.05	6.13	1.90	50.9	97.10	50.2
3	40	rLasso-2	-0.25	1.02	4.23	1.50	50.1	96.50	50.2
3	100	ATE	-0.02	0.39	1.15	12.20	81.6	99.80	52.1
3	100	Lasso	-0.21	0.43	6.64	0.70	58.4	98.90	52.1
3	100	reduced	-0.13	0.89	1.55	24.20	56.7	84.80	52.1
3	100	rLasso-1	-0.14	0.89	4.18	3.61	56.6	98.10	52.1
3	100	rLasso-2	-0.20	0.81	3.02	3.30	54.2	98.30	52.1
3	220	ATE	0.01	0.30	0.61	35.30	95.1	100.00	48.7
3	220	full	0.05	1.34	5.25	3.60	60.1	97.90	48.7
3	220	Lasso	-0.16	0.34	4.35	2.40	62.5	98.90	48.7
3	220	reduced	-0.05	0.66	1.23	25.80	63.0	89.80	48.7
3	220	reduced-Scheffe	-0.05	0.66	7.93	0.00	63.0	100.00	48.7
3	220	rLasso-1	-0.06	0.68	2.84	4.31	62.1	98.20	48.7
3	220	rLasso-2	-0.09	0.61	2.15	4.31	60.7	97.49	48.7
3	350	ATE	0.02	0.27	0.46	62.40	99.3	100.00	47.7
3	350	full	0.02	0.89	3.53	5.20	59.7	98.40	47.7
3	350	Lasso	-0.13	0.28	3.36	4.50	69.6	99.30	47.7
3	350	reduced	-0.01	0.54	1.05	32.00	65.9	90.20	47.7
3	350	reduced-Scheffe	-0.01	0.54	6.49	0.00	65.9	100.00	47.7
3	350	rLasso-1	0.00	0.54	2.21	8.31	66.0	97.80	47.7
3	350	rLasso-2	-0.05	0.51	1.71	9.50	64.0	98.20	47.7

Table 17: Diagnostic measures for case 4 with 50 biomarkers. Columns 4 and 5 show the bias and the $\sqrt{MSE}$ for the point estimate of the PITE. The sixth column shows the median width of the confidence intervals for the PITE, and the last columns show the proportion of subjects in the identified subgroup when considering the using the limits of the confidence intervals and the point estimates. Since methods reduced and reduced-Scheffe have the same point estimate, bias and MSE are equal.

Case	N	Method	Bias	$\sqrt{MSE}$	Width	% in $\hat{B}_l$	% in $\hat{B}$	% in $\hat{B}_u$	% in B
4	40	ATE							62.8
4	40	Lasso	-0.21	0.66	10.92	0.80	55.0	99.20	62.8
4	40	reduced	-0.17	1.09	1.86	23.30	54.2	79.90	62.8
4	40	rLasso-1	-0.18	1.10	6.64	1.71	54.9	97.19	62.8
4	40	rLasso-2	-0.23	1.10	4.64	2.80	49.8	98.40	62.8
4	100	ATE	0.00	0.69	1.19	13.20	80.1	99.70	61.8
4	100	Lasso	-0.22	0.55	6.71	1.40	56.0	98.20	61.8
4	100	reduced	-0.17	0.88	1.58	24.40	55.2	80.40	61.8
4	100	rLasso-1	-0.18	0.92	4.34	3.90	54.4	96.00	61.8
4	100	rLasso-2	-0.19	0.87	3.24	5.20	52.3	95.50	61.8
4	220	ATE	0.01	0.65	0.63	32.00	93.6	100.00	62.9
4	220	full	0.05	1.34	5.25	6.50	58.1	97.60	62.9
4	220	Lasso	-0.17	0.42	3.82	8.70	55.5	97.60	62.9
4	220	reduced	-0.06	0.65	1.23	32.50	58.5	80.90	62.9
4	220	reduced-Scheffe	-0.06	0.65	7.92	0.00	58.5	100.00	62.9
4	220	rLasso-1	-0.07	0.68	2.97	10.11	58.9	94.09	62.9
4	220	rLasso-2	-0.10	0.61	2.25	12.40	56.1	93.20	62.9
4	350	ATE	0.03	0.63	0.48	57.60	98.4	100.00	60.3
4	350	full	0.02	0.89	3.53	9.00	57.7	96.90	60.3
4	350	Lasso	-0.13	0.34	3.01	10.30	56.8	95.80	60.3
4	350	reduced	-0.02	0.52	1.06	38.10	59.6	82.30	60.3
4	350	reduced-Scheffe	-0.02	0.52	6.47	0.00	59.6	100.00	60.3
4	350	rLasso-1	-0.01	0.54	2.29	16.03	61.3	92.69	60.3
4	350	rLasso-2	-0.07	0.52	1.79	16.70	58.6	90.90	60.3

Table 18: Diagnostic measures for case 5 with 50 biomarkers. Columns 4 and 5 show the bias and the $\sqrt{MSE}$ for the point estimate of the PITE. The sixth column shows the median width of the confidence intervals for the PITE, and the last columns show the proportion of subjects in the identified subgroup when considering the using the limits of the confidence intervals and the point estimates. Since methods reduced and reduced-Scheffe have the same point estimate, bias and MSE are equal.

Case	N	Method	Bias	$\sqrt{MSE}$	Width	% in $\hat{B}_l$	% in $\hat{B}$	% in $\hat{B}_u$	% in B
5	40	ATE							100
5	40	Lasso	-0.22	0.44	10.07	0.80	52.8	98.70	100
5	40	reduced	-0.19	1.03	1.73	23.00	51.7	80.10	100
5	40	rLasso-1	-0.22	1.04	5.95	2.71	50.3	98.09	100
5	40	rLasso-2	-0.23	0.97	4.12	2.10	50.0	97.50	100
5	100	ATE	-0.01	0.28	1.14	12.70	81.3	99.70	100
5	100	Lasso	-0.20	0.37	6.52	0.60	59.8	99.70	100
5	100	reduced	-0.10	0.87	1.53	24.30	57.8	85.90	100
5	100	rLasso-1	-0.12	0.86	4.07	2.21	57.2	98.29	100
5	100	rLasso-2	-0.19	0.78	2.95	3.50	53.3	97.80	100
5	220	ATE	0.00	0.15	0.60	36.40	95.4	100.00	100
5	220	full	0.05	1.34	5.25	4.00	60.0	98.70	100
5	220	Lasso	-0.16	0.28	4.50	1.80	67.5	99.70	100
5	220	reduced	-0.03	0.63	1.19	29.00	65.1	91.50	100
5	220	reduced-Scheffe	-0.03	0.63	7.71	0.00	65.1	100.00	100
5	220	rLasso-1	-0.05	0.65	2.77	4.31	63.2	98.90	100
5	220	rLasso-2	-0.08	0.56	2.07	3.50	62.5	98.50	100
5	350	ATE	0.01	0.11	0.45	62.20	99.2	100.00	100
5	350	full	0.02	0.89	3.53	5.40	61.2	99.30	100
5	350	Lasso	-0.13	0.24	3.52	3.60	74.4	100.00	100
5	350	reduced	-0.01	0.54	1.02	34.00	69.6	93.80	100
5	350	reduced-Scheffe	-0.01	0.54	6.26	0.00	69.6	100.00	100
5	350	rLasso-1	0.00	0.54	2.18	6.51	68.7	98.70	100
5	350	rLasso-2	-0.04	0.48	1.66	9.20	66.6	99.20	100

Table 19: Diagnostic measures for case 6 with 50 biomarkers. Columns 4 and 5 show the bias and the $\sqrt{MSE}$ for the point estimate of the PITE. The sixth column shows the median width of the confidence intervals for the PITE, and the last columns show the proportion of subjects in the identified subgroup when considering the using the limits of the confidence intervals and the point estimates. Since methods reduced and reduced-Scheffe have the same point estimate, bias and MSE are equal.

Case	N	Method	Bias	$\sqrt{MSE}$	Width	% in $\hat{B}_l$	% in $\hat{B}$	% in $\hat{B}_u$	% in B
6	40	ATE							75.5
6	40	Lasso	-0.41	0.77	11.22	1.30	58.2	99.40	75.5
6	40	reduced	-0.29	1.14	1.89	27.30	59.3	84.00	75.5
6	40	rLasso-1	-0.31	1.16	6.60	3.51	58.0	97.89	75.5
6	40	rLasso-2	-0.41	1.17	4.65	3.60	55.2	97.80	75.5
6	100	ATE	0.00	0.69	1.19	35.90	94.3	100.00	75.2
6	100	Lasso	-0.34	0.63	6.80	3.10	65.2	99.50	75.2
6	100	reduced	-0.17	0.90	1.65	33.50	65.8	87.30	75.2
6	100	rLasso-1	-0.19	0.94	4.52	7.10	63.8	97.50	75.2
6	100	rLasso-2	-0.25	0.94	3.34	9.00	61.2	96.00	75.2
6	220	ATE	0.01	0.65	0.63	87.70	100.0	100.00	74.7
6	220	full	0.05	1.34	5.25	8.50	65.4	98.60	74.7
6	220	Lasso	-0.21	0.46	3.66	15.10	72.2	99.20	74.7
6	220	reduced	-0.02	0.63	1.25	45.00	71.5	91.30	74.7
6	220	reduced-Scheffe	-0.02	0.63	7.98	0.00	71.5	100.00	74.7
6	220	rLasso-1	-0.03	0.68	2.98	17.92	68.5	98.10	74.7
6	220	rLasso-2	-0.06	0.61	2.28	21.20	71.4	97.90	74.7
6	350	ATE	0.03	0.63	0.48	99.00	100.0	100.00	75.4
6	350	full	0.02	0.89	3.53	11.60	67.8	98.50	75.4
6	350	Lasso	-0.15	0.35	2.80	20.20	73.5	98.00	75.4
6	350	reduced	0.01	0.51	1.07	48.70	72.6	90.40	75.4
6	350	reduced-Scheffe	0.01	0.51	6.48	0.00	72.6	100.00	75.4
6	350	rLasso-1	0.02	0.52	2.27	26.48	72.7	97.29	75.4
6	350	rLasso-2	-0.01	0.52	1.82	30.30	71.6	97.50	75.4

4.4 Sensitivity and Specificity

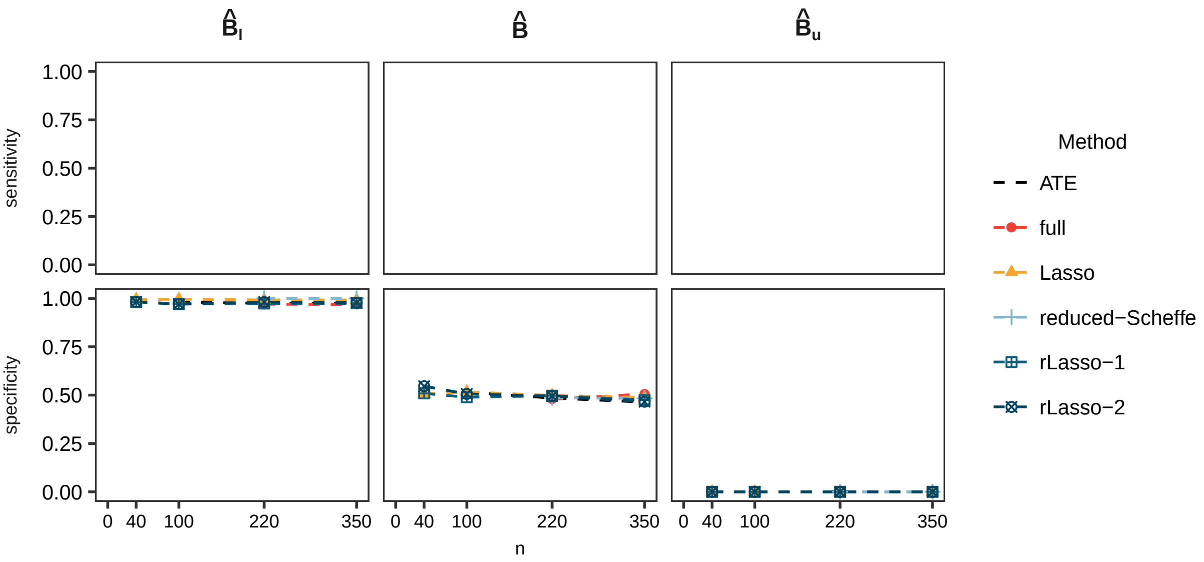

Figure 19: Specificity for identified subgroup by method and sample size for Case 1 and 50 biomarkers. Sensitivity is not shown as there are no patients with $D(X) > 0$

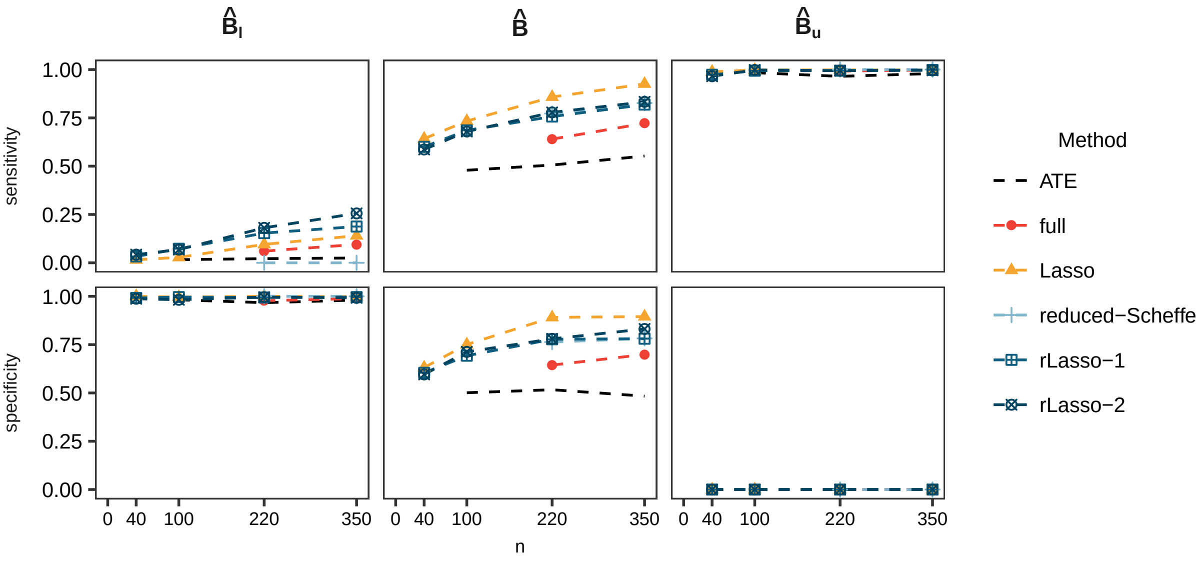

Figure 20: Sensitivity and specificity for identified subgroup by method and sample size for Case 2 and 50 biomarkers.

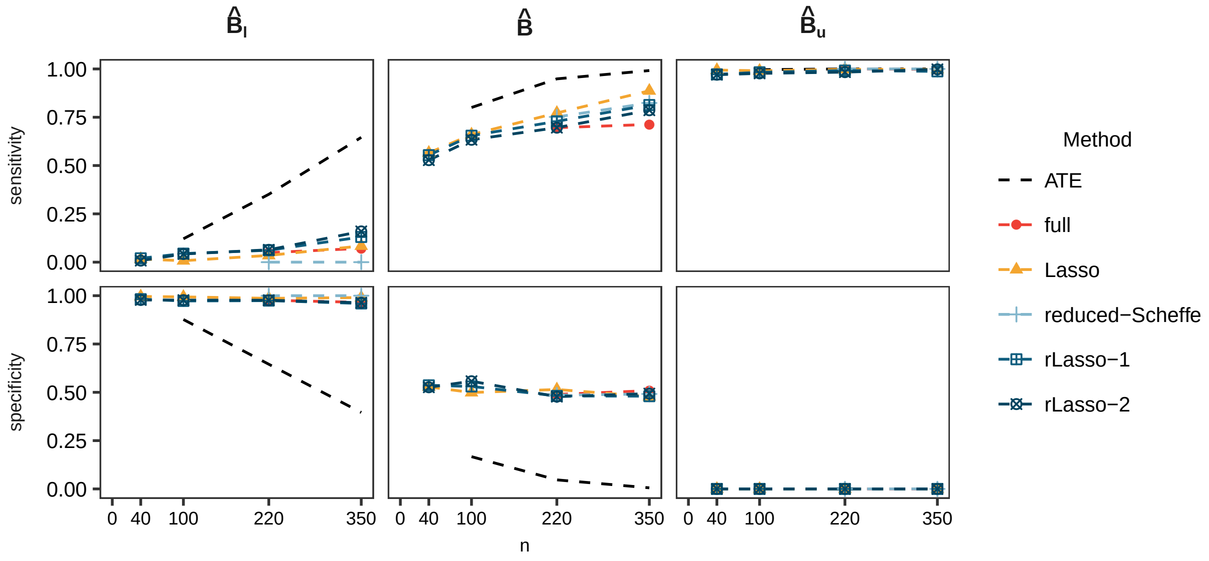

Figure 21: Sensitivity and specificity for identified subgroup by method and sample size for Case 3 and 50 biomarkers.

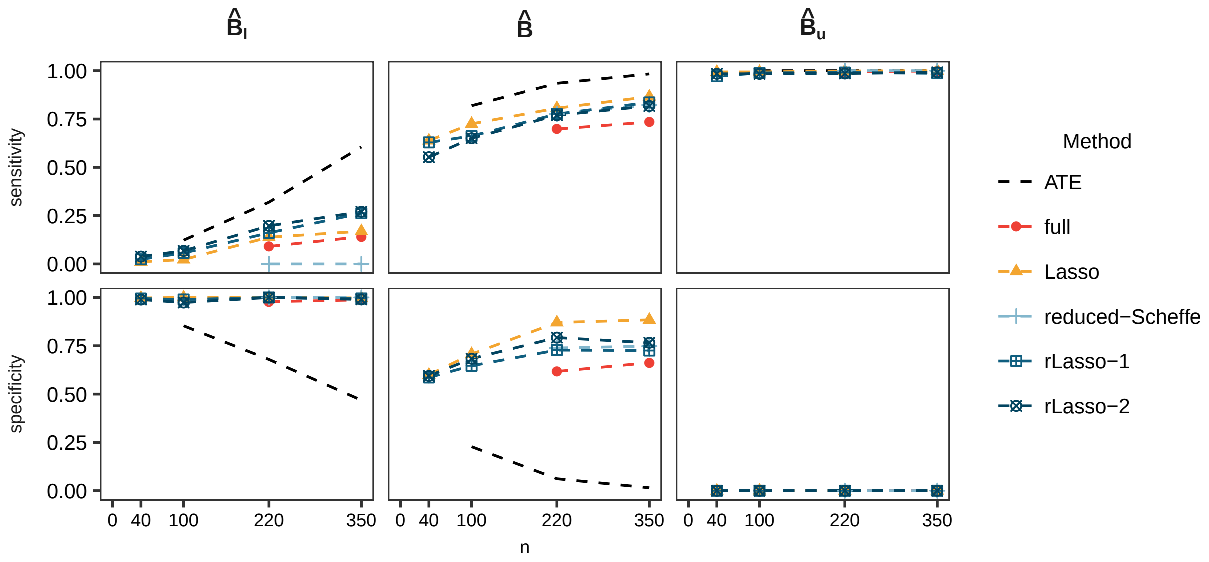

Figure 22: Sensitivity and specificity for identified subgroup by method and sample size for Case 4 and 50 biomarkers.

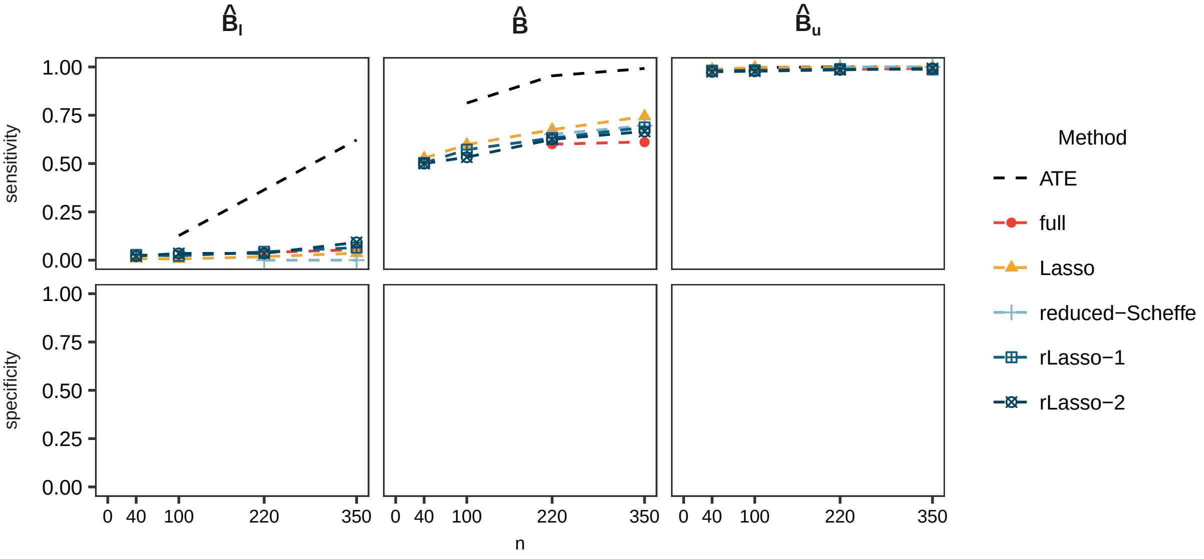

Figure 23: Sensitivity for identified subgroup by method and sample size for Case 5 and 50 biomarkers. Specificity is not shown as all patients have $D(X) > 0$

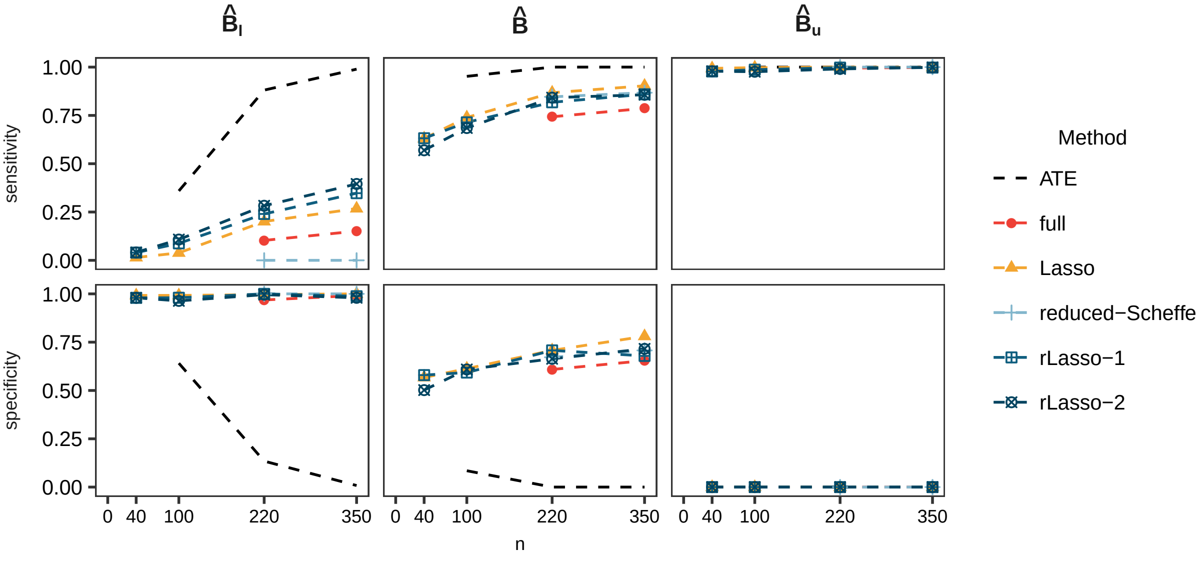

Figure 24: Sensitivity and specificity for identified subgroup by method and sample size for Case 6 and 50 biomarkers.

5 Results for 100 Biomarkers

5.1 Percent of selection

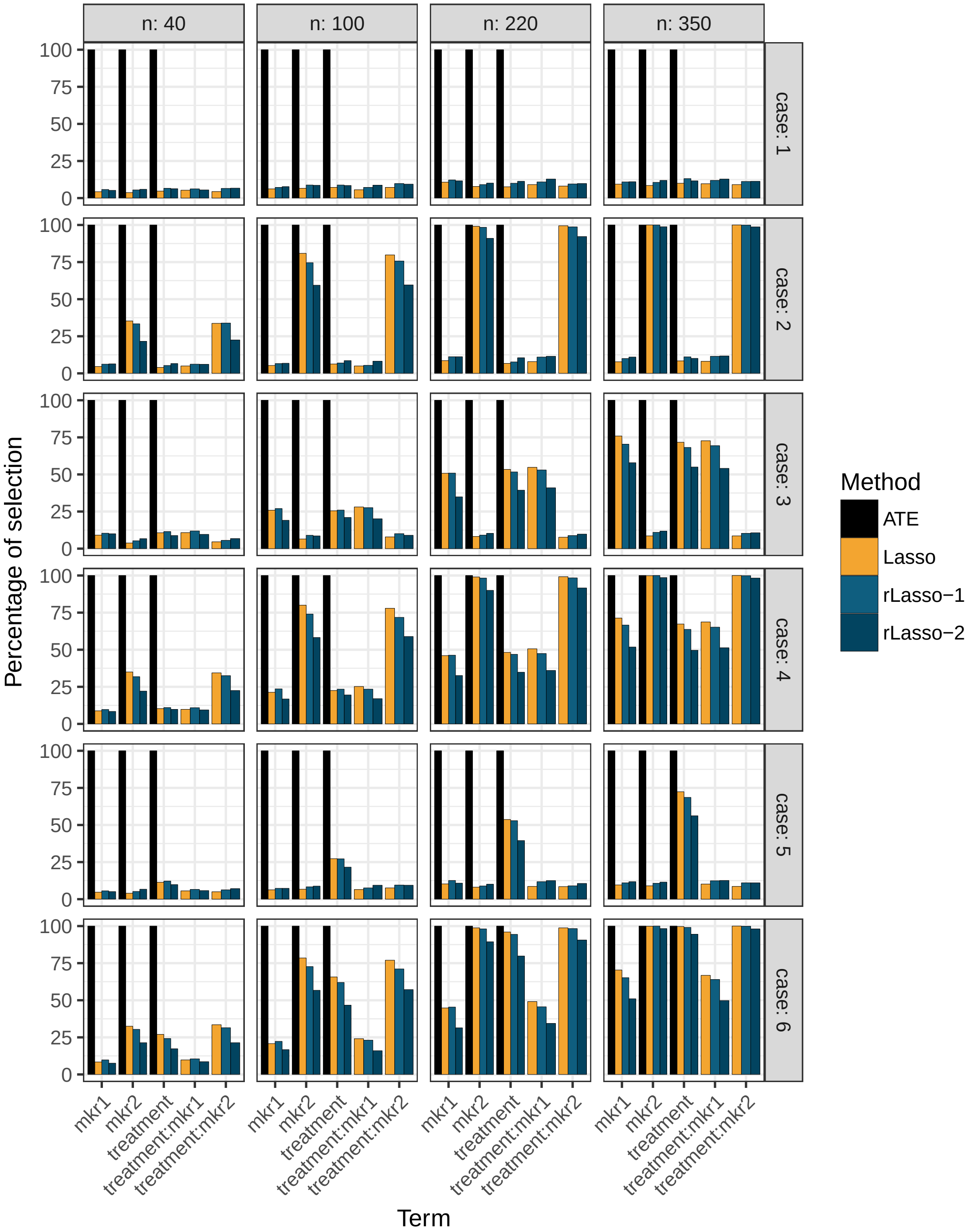

Figure 25: Selection probabilities for relevant terms in the model by method, case and sample size for 100 biomarkers. The full model is not represented here as all variables are included 100% of the times. The reduced model is not represented as it has the same values as the Lasso

5.2 Coverage of confidence intervals

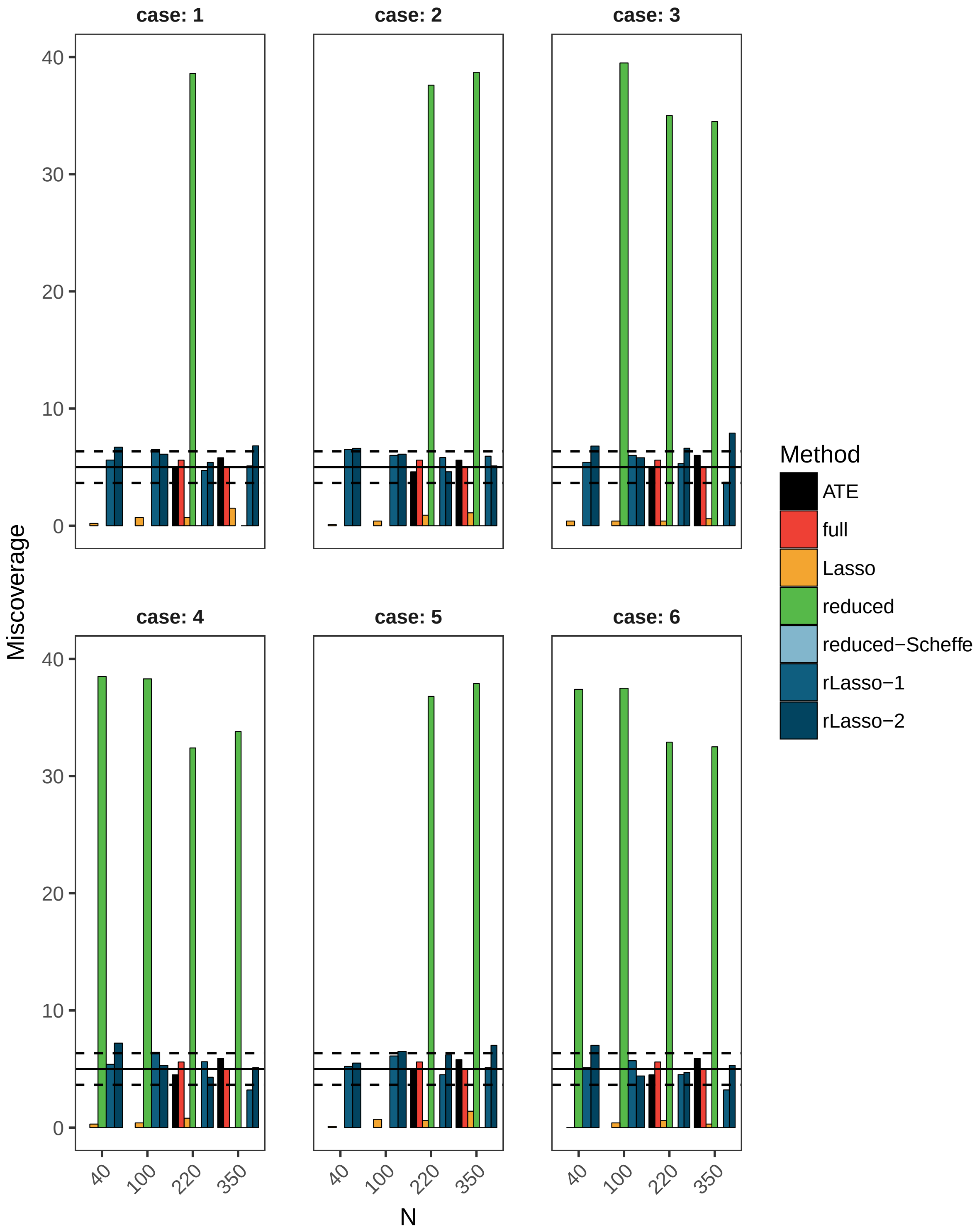

Figure 26: Average miscoverage of the confidence intervals for the PITE by method, case and sample size for 100 biomarkers. The line at 5% indicates the target miscoverage.

5.3 Bias, MSE and width

Table 20: Diagnostic measures for case 1 with 100 biomarkers. Columns 4 and 5 show the bias and the $\sqrt{MSE}$ for the point estimate of the PITE. The sixth column shows the median width of the confidence intervals for the PITE, and the last columns show the proportion of subjects in the identified subgroup when considering the using the limits of the confidence intervals and the point estimates. Since methods reduced and reduced-Scheffe have the same point estimate, bias and MSE are equal.

Case	N	Method	Bias	$\sqrt{MSE}$	Width	% in $\hat{B}_l$	% in $\hat{B}$	% in $\hat{B}_u$	% in B
1	40	ATE							0
1	40	Lasso	-0.01	0.41	11.64	0.10	50.2	99.50	0
1	40	reduced	-0.05	1.06	1.63	21.80	48.8	75.20	0
1	40	rLasso-1	-0.04	1.05	7.33	2.40	48.4	96.80	0
1	40	rLasso-2	-0.04	0.99	4.75	3.40	48.3	96.60	0
1	100	ATE							0
1	100	Lasso	0.00	0.34	8.05	0.30	50.3	99.60	0
1	100	reduced	-0.01	0.96	1.61	20.50	50.9	79.30	0
1	100	rLasso-1	-0.01	0.92	5.19	3.60	50.6	97.10	0
1	100	rLasso-2	0.00	0.86	3.74	3.70	49.7	97.60	0
1	220	ATE	0.00	0.18	0.73	3.00	50.9	98.10	0
1	220	full	-0.16	5.16	19.87	2.70	48.5	97.10	0
1	220	Lasso	0.01	0.25	5.91	0.50	50.9	99.80	0
1	220	reduced	0.01	0.77	1.37	19.90	50.4	81.30	0
1	220	reduced-Scheffe	0.01	0.77	15.13	0.00	50.4	100.00	0
1	220	rLasso-1	0.02	0.76	3.60	2.41	52.5	97.69	0
1	220	rLasso-2	0.01	0.66	2.62	2.90	50.4	97.50	0
1	350	ATE	0.00	0.13	0.50	2.60	47.7	96.80	0
1	350	full	0.06	1.64	6.46	2.80	50.3	97.80	0
1	350	Lasso	0.01	0.21	4.41	0.60	50.2	99.10	0
1	350	reduced	0.04	0.66	1.14	22.30	53.1	81.20	0
1	350	reduced-Scheffe	0.04	0.66	10.28	0.00	53.1	100.00	0
1	350	rLasso-1	0.03	0.64	2.90	2.20	53.3	97.10	0
1	350	rLasso-2	0.01	0.58	2.12	3.31	49.1	96.49	0

Table 21: Diagnostic measures for case 2 with 100 biomarkers. Columns 4 and 5 show the bias and the $\sqrt{MSE}$ for the point estimate of the PITE. The sixth column shows the median width of the confidence intervals for the PITE, and the last columns show the proportion of subjects in the identified subgroup when considering the using the limits of the confidence intervals and the point estimates. Since methods reduced and reduced-Scheffe have the same point estimate, bias and MSE are equal.

Case	N	Method	Bias	$\sqrt{MSE}$	Width	% in $\hat{B}_l$	% in $\hat{B}$	% in $\hat{B}_u$	% in B
2	40	ATE							49.8
2	40	Lasso	0.01	0.66	13.36	0.30	50.1	99.60	49.8
2	40	reduced	0.01	1.15	1.78	21.60	49.7	78.70	49.8
2	40	rLasso-1	0.03	1.23	7.89	2.80	50.5	96.60	49.8
2	40	rLasso-2	0.05	1.19	5.27	4.20	50.5	97.10	49.8
2	100	ATE							51.1
2	100	Lasso	0.01	0.53	8.35	0.70	51.5	99.60	51.1
2	100	reduced	0.04	1.00	1.63	24.20	52.6	79.10	51.1
2	100	rLasso-1	0.01	0.99	5.33	4.70	52.0	95.80	51.1
2	100	rLasso-2	0.00	0.96	3.97	5.11	50.5	94.99	51.1
2	220	ATE	0.01	0.61	0.75	2.70	50.6	98.10	48.6
2	220	full	-0.16	5.16	19.87	3.10	47.6	97.50	48.6
2	220	Lasso	0.01	0.34	5.61	2.30	50.7	98.20	48.6
2	220	reduced	0.01	0.75	1.32	25.20	50.2	75.20	48.6
2	220	reduced-Scheffe	0.01	0.75	14.62	0.00	50.2	100.00	48.6
2	220	rLasso-1	0.02	0.73	3.68	5.62	52.0	93.78	48.6
2	220	rLasso-2	0.02	0.66	2.77	7.21	49.5	93.19	48.6
2	350	ATE	-0.02	0.59	0.52	2.40	48.7	96.80	52.2
2	350	full	0.06	1.64	6.46	3.60	51.8	97.40	52.2
2	350	Lasso	0.00	0.28	4.46	1.90	53.5	97.90	52.2
2	350	reduced	0.04	0.63	1.10	29.40	53.0	74.90	52.2
2	350	reduced-Scheffe	0.04	0.63	9.67	0.00	53.0	100.00	52.2
2	350	rLasso-1	0.03	0.60	2.98	7.55	52.9	90.95	52.2
2	350	rLasso-2	0.02	0.57	2.21	8.01	51.7	89.69	52.2

Table 22: Diagnostic measures for case 3 with 100 biomarkers. Columns 4 and 5 show the bias and the $\sqrt{MSE}$ for the point estimate of the PITE. The sixth column shows the median width of the confidence intervals for the PITE, and the last columns show the proportion of subjects in the identified subgroup when considering the using the limits of the confidence intervals and the point estimates. Since methods reduced and reduced-Scheffe have the same point estimate, bias and MSE are equal.

Case	N	Method	Bias	$\sqrt{MSE}$	Width	% in $\hat{B}_l$	% in $\hat{B}$	% in $\hat{B}_u$	% in B
3	40	ATE							50.8
3	40	Lasso	-0.25	0.54	12.25	0.30	51.0	99.60	50.8
3	40	reduced	-0.25	1.10	1.66	23.60	49.8	76.80	50.8
3	40	rLasso-1	-0.23	1.12	7.28	3.21	50.6	97.09	50.8
3	40	rLasso-2	-0.22	1.09	5.03	3.30	50.7	96.50	50.8
3	100	ATE							55.1
3	100	Lasso	-0.23	0.47	8.39	0.40	56.1	99.90	55.1
3	100	reduced	-0.16	1.03	1.65	26.70	55.1	83.30	55.1
3	100	rLasso-1	-0.19	1.01	5.32	3.60	53.2	97.50	55.1
3	100	rLasso-2	-0.17	0.92	3.79	4.30	54.3	97.60	55.1
3	220	ATE	0.02	0.32	0.73	26.90	91.2	100.00	47.4
3	220	full	-0.16	5.16	19.87	2.90	50.5	97.50	47.4
3	220	Lasso	-0.17	0.37	6.18	0.70	60.6	99.70	47.4
3	220	reduced	-0.08	0.81	1.44	24.70	57.2	86.50	47.4
3	220	reduced-Scheffe	-0.08	0.81	15.67	0.00	57.2	100.00	47.4
3	220	rLasso-1	-0.08	0.80	3.74	4.40	58.3	97.50	47.4
3	220	rLasso-2	-0.12	0.71	2.71	5.21	57.6	97.09	47.4
3	350	ATE	0.00	0.28	0.50	49.20	97.0	100.00	48.3
3	350	full	0.06	1.64	6.46	4.20	57.9	98.40	48.3
3	350	Lasso	-0.14	0.32	4.69	1.80	65.7	99.80	48.3
3	350	reduced	-0.01	0.68	1.21	30.50	63.2	87.50	48.3
3	350	reduced-Scheffe	-0.01	0.68	10.74	0.00	63.2	100.00	48.3
3	350	rLasso-1	-0.03	0.64	2.98	6.65	62.4	98.49	48.3
3	350	rLasso-2	-0.07	0.63	2.19	7.51	60.0	96.80	48.3

Table 23: Diagnostic measures for case 4 with 100 biomarkers. Columns 4 and 5 show the bias and the $\sqrt{MSE}$ for the point estimate of the PITE. The sixth column shows the median width of the confidence intervals for the PITE, and the last columns show the proportion of subjects in the identified subgroup when considering the using the limits of the confidence intervals and the point estimates. Since methods reduced and reduced-Scheffe have the same point estimate, bias and MSE are equal.

Case	N	Method	Bias	$\sqrt{MSE}$	Width	% in $\hat{B}_l$	% in $\hat{B}$	% in $\hat{B}_u$	% in B
4	40	ATE							63.8
4	40	Lasso	-0.24	0.74	13.90	0.20	51.2	99.70	63.8
4	40	reduced	-0.21	1.22	1.83	21.20	51.1	80.00	63.8
4	40	rLasso-1	-0.20	1.23	8.18	2.50	52.0	96.50	63.8
4	40	rLasso-2	-0.18	1.24	5.42	4.30	52.3	96.40	63.8
4	100	ATE							65.0
4	100	Lasso	-0.23	0.63	8.89	1.60	56.3	99.40	65.0
4	100	reduced	-0.16	1.06	1.72	27.20	54.7	81.60	65.0
4	100	rLasso-1	-0.15	1.04	5.40	4.91	55.9	97.09	65.0
4	100	rLasso-2	-0.19	1.02	4.07	4.90	55.6	96.90	65.0
4	220	ATE	0.02	0.67	0.76	25.00	90.3	100.00	62.1
4	220	full	-0.16	5.16	19.87	3.20	49.7	97.80	62.1
4	220	Lasso	-0.17	0.45	6.09	2.30	55.3	98.80	62.1
4	220	reduced	-0.08	0.76	1.39	28.90	58.5	80.00	62.1
4	220	reduced-Scheffe	-0.08	0.76	15.03	0.00	58.5	100.00	62.1
4	220	rLasso-1	-0.09	0.76	3.86	7.13	56.2	95.18	62.1
4	220	rLasso-2	-0.13	0.73	2.88	8.60	54.8	94.90	62.1
4	350	ATE	-0.01	0.64	0.52	45.30	96.5	100.00	63.5
4	350	full	0.06	1.64	6.46	5.10	58.3	98.30	63.5
4	350	Lasso	-0.16	0.38	4.50	4.00	58.5	97.90	63.5
4	350	reduced	0.00	0.64	1.16	36.70	62.6	81.30	63.5
4	350	reduced-Scheffe	0.00	0.64	10.19	0.00	62.6	100.00	63.5
4	350	rLasso-1	-0.04	0.63	3.05	10.84	60.0	94.78	63.5
4	350	rLasso-2	-0.10	0.63	2.31	11.62	59.4	92.69	63.5

Table 24: Diagnostic measures for case 5 with 100 biomarkers. Columns 4 and 5 show the bias and the $\sqrt{MSE}$ for the point estimate of the PITE. The sixth column shows the median width of the confidence intervals for the PITE, and the last columns show the proportion of subjects in the identified subgroup when considering the using the limits of the confidence intervals and the point estimates. Since methods reduced and reduced-Scheffe have the same point estimate, bias and MSE are equal.

Case	N	Method	Bias	$\sqrt{MSE}$	Width	% in $\hat{B}_l$	% in $\hat{B}$	% in $\hat{B}_u$	% in B
5	40	ATE							100
5	40	Lasso	-0.24	0.47	12.21	0.00	51.8	99.90	100
5	40	reduced	-0.25	1.06	1.66	22.90	50.6	77.70	100
5	40	rLasso-1	-0.22	1.09	7.16	2.41	51.2	97.09	100
5	40	rLasso-2	-0.23	1.01	4.95	3.50	48.5	97.70	100
5	100	ATE							100
5	100	Lasso	-0.21	0.40	8.33	0.50	55.6	99.70	100
5	100	reduced	-0.14	0.97	1.63	25.60	54.6	82.20	100
5	100	rLasso-1	-0.16	0.96	5.23	4.11	54.6	97.80	100
5	100	rLasso-2	-0.17	0.89	3.77	4.30	52.4	97.30	100
5	220	ATE	0.00	0.18	0.73	26.90	91.8	100.00	100
5	220	full	-0.16	5.16	19.87	3.00	50.2	97.50	100
5	220	Lasso	-0.17	0.32	6.18	0.70	61.6	99.90	100
5	220	reduced	-0.09	0.78	1.41	26.00	57.4	87.00	100
5	220	reduced-Scheffe	-0.09	0.78	15.75	0.00	57.4	100.00	100
5	220	rLasso-1	-0.08	0.78	3.65	3.01	58.8	98.00	100
5	220	rLasso-2	-0.14	0.69	2.65	4.80	56.6	97.30	100
5	350	ATE	0.00	0.13	0.50	48.40	97.4	100.00	100
5	350	full	0.06	1.64	6.46	4.50	57.1	98.50	100
5	350	Lasso	-0.15	0.27	4.51	1.20	68.1	99.50	100
5	350	reduced	-0.01	0.67	1.19	31.50	64.4	89.30	100
5	350	reduced-Scheffe	-0.01	0.67	10.65	0.00	64.4	100.00	100
5	350	rLasso-1	-0.03	0.65	2.95	4.21	63.9	98.10	100
5	350	rLasso-2	-0.08	0.61	2.13	5.42	59.1	97.09	100

Table 25: Diagnostic measures for case 6 with 100 biomarkers. Columns 4 and 5 show the bias and the $\sqrt{MSE}$ for the point estimate of the PITE. The sixth column shows the median width of the confidence intervals for the PITE, and the last columns show the proportion of subjects in the identified subgroup when considering the using the limits of the confidence intervals and the point estimates. Since methods reduced and reduced-Scheffe have the same point estimate, bias and MSE are equal.

Case	N	Method	Bias	$\sqrt{MSE}$	Width	% in $\hat{B}_l$	% in $\hat{B}$	% in $\hat{B}_u$	% in B
6	40	ATE							77.2
6	40	Lasso	-0.43	0.84	13.86	0.30	56.3	99.60	77.2
6	40	reduced	-0.33	1.27	1.90	24.70	56.7	83.40	77.2
6	40	rLasso-1	-0.35	1.29	8.10	3.31	56.4	98.00	77.2
6	40	rLasso-2	-0.36	1.32	5.57	4.31	55.4	96.69	77.2
6	100	ATE							77.4
6	100	Lasso	-0.37	0.70	9.00	2.00	66.5	99.60	77.4
6	100	reduced	-0.18	1.08	1.74	33.80	62.5	86.90	77.4
6	100	rLasso-1	-0.21	1.08	5.65	7.41	63.2	97.80	77.4
6	100	rLasso-2	-0.25	1.06	4.21	7.41	62.9	97.50	77.4
6	220	ATE	0.02	0.67	0.76	74.10	99.8	100.00	74.5
6	220	full	-0.16	5.16	19.87	3.50	51.6	97.90	74.5
6	220	Lasso	-0.22	0.48	5.96	5.20	72.3	99.30	74.5
6	220	reduced	-0.04	0.74	1.41	40.70	69.2	88.30	74.5
6	220	reduced-Scheffe	-0.04	0.74	15.11	0.00	69.2	100.00	74.5
6	220	rLasso-1	-0.04	0.76	3.92	11.86	67.8	97.89	74.5
6	220	rLasso-2	-0.09	0.73	2.96	16.32	69.0	97.60	74.5
6	350	ATE	-0.01	0.64	0.52	95.90	100.0	100.00	74.9
6	350	full	0.06	1.64	6.46	7.10	62.9	98.40	74.9
6	350	Lasso	-0.18	0.40	4.50	8.30	75.7	99.10	74.9
6	350	reduced	0.01	0.62	1.16	47.60	73.4	90.70	74.9
6	350	reduced-Scheffe	0.01	0.62	10.16	0.00	73.4	100.00	74.9
6	350	rLasso-1	0.00	0.62	3.08	19.20	73.0	98.09	74.9
6	350	rLasso-2	-0.03	0.62	2.32	22.75	71.4	97.09	74.9

5.4 Sensitivity and Specificity

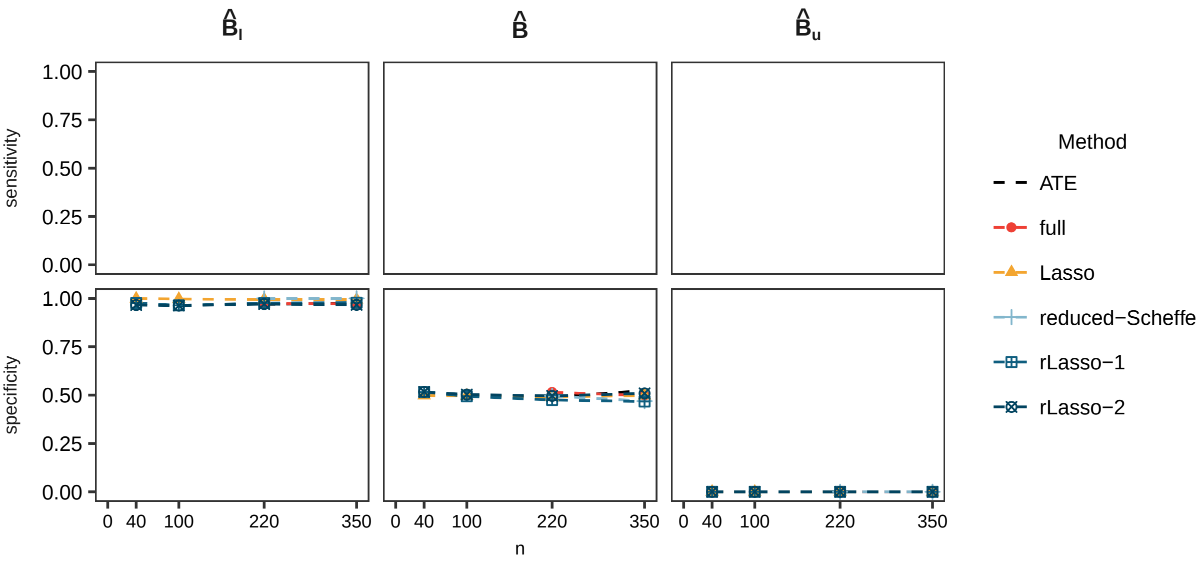

Figure 27: Specificity for identified subgroup by method and sample size for Case 1 and 100 biomarkers. Sensitivity is not shown as there are no patients with $D(X) > 0$

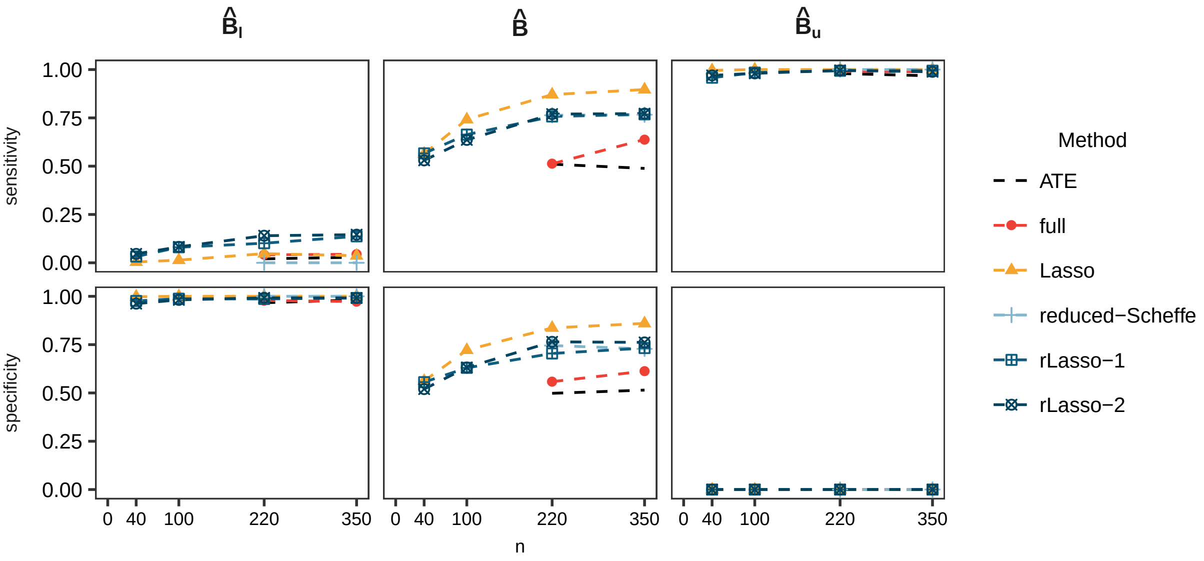

Figure 28: Sensitivity and specificity for identified subgroup by method and sample size for Case 2 and 100 biomarkers.

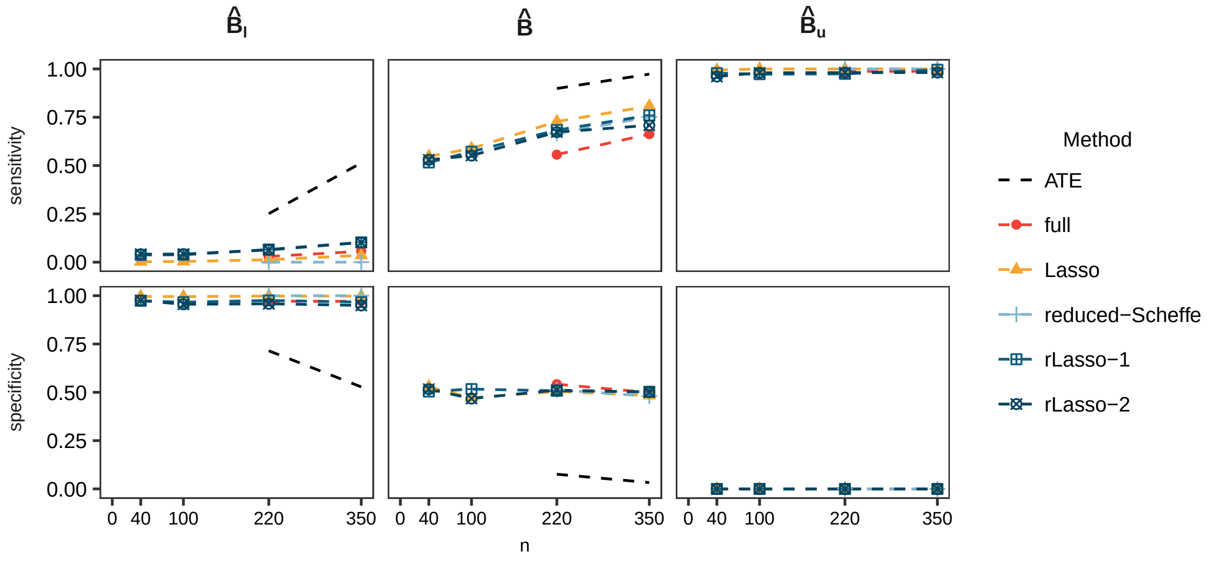

Figure 29: Sensitivity and specificity for identified subgroup by method and sample size for Case 3 and 100 biomarkers.

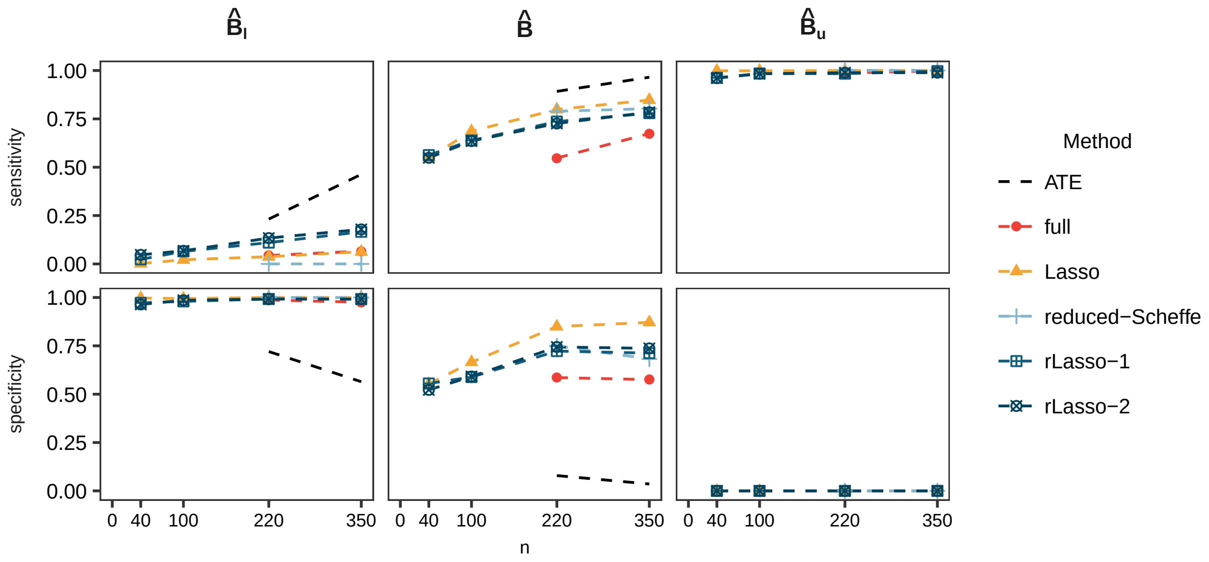

Figure 30: Sensitivity and specificity for identified subgroup by method and sample size for Case 4 and 100 biomarkers.

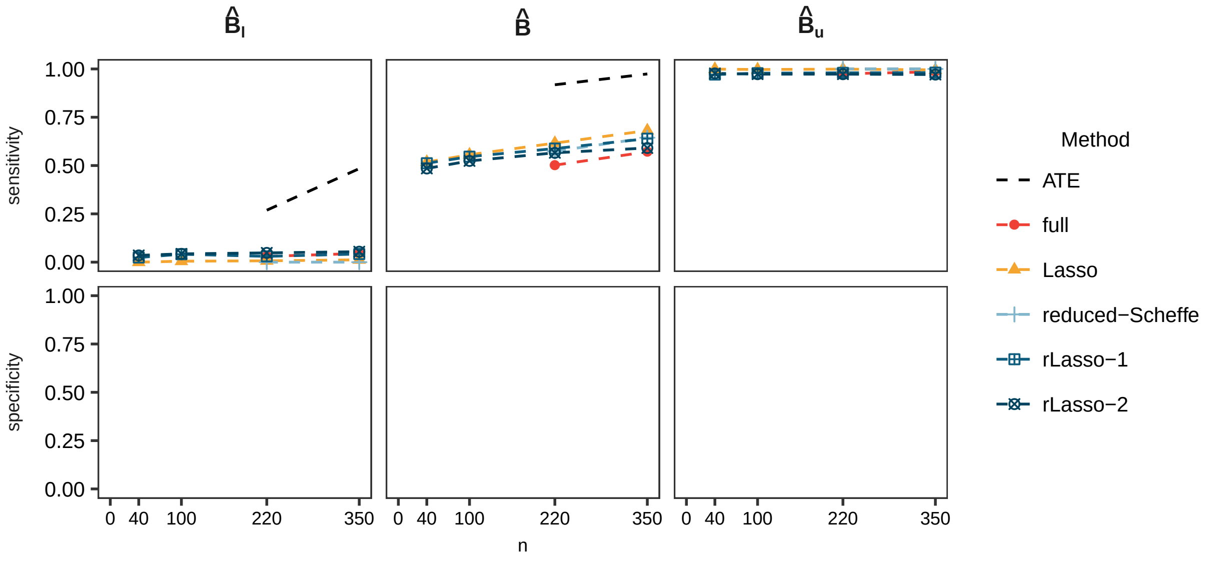

Figure 31: Sensitivity for identified subgroup by method and sample size for Case 5 and 100 biomarkers. Specificity is not shown as all patients have $D(X) > 0$

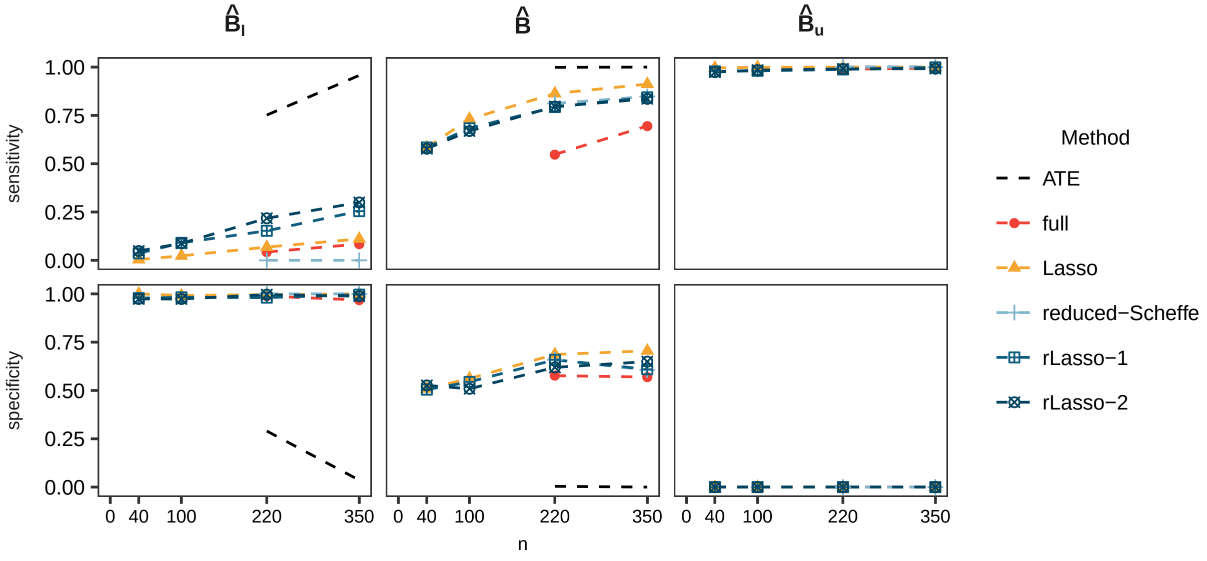

Figure 32: Sensitivity and specificity for identified subgroup by method and sample size for Case 6 and 100 biomarkers.

6 Results for 1000 Biomarkers

We now evaluate the case where $p >> n$. We increased the true effects as presented in the following table. We also increase the penalization parameter using $l = .75$.

Table 26: Choices of parameters for the simulation scenarios

Case	α	β	γ_1	γ_2	δ_1	δ_2
1: No predictive markers	0.00	0.00	0.00	0.000	0.00	0.000
2: X_2 is predictive	0.00	0.00	0.00	0.433	0.00	0.433
3: X_1 is predictive	0.25	0.25	0.25	0.000	0.25	0.000
4: X_1 and X_2 are predictive	0.25	0.25	0.25	0.433	0.25	0.433
5: Overall treatment effect	0.50	0.50	0.00	0.000	0.00	0.000
6: Overall treatment effect and X_1 and X_2 are predictive	0.75	0.75	0.25	0.433	0.25	0.433

6.1 Percent of selection

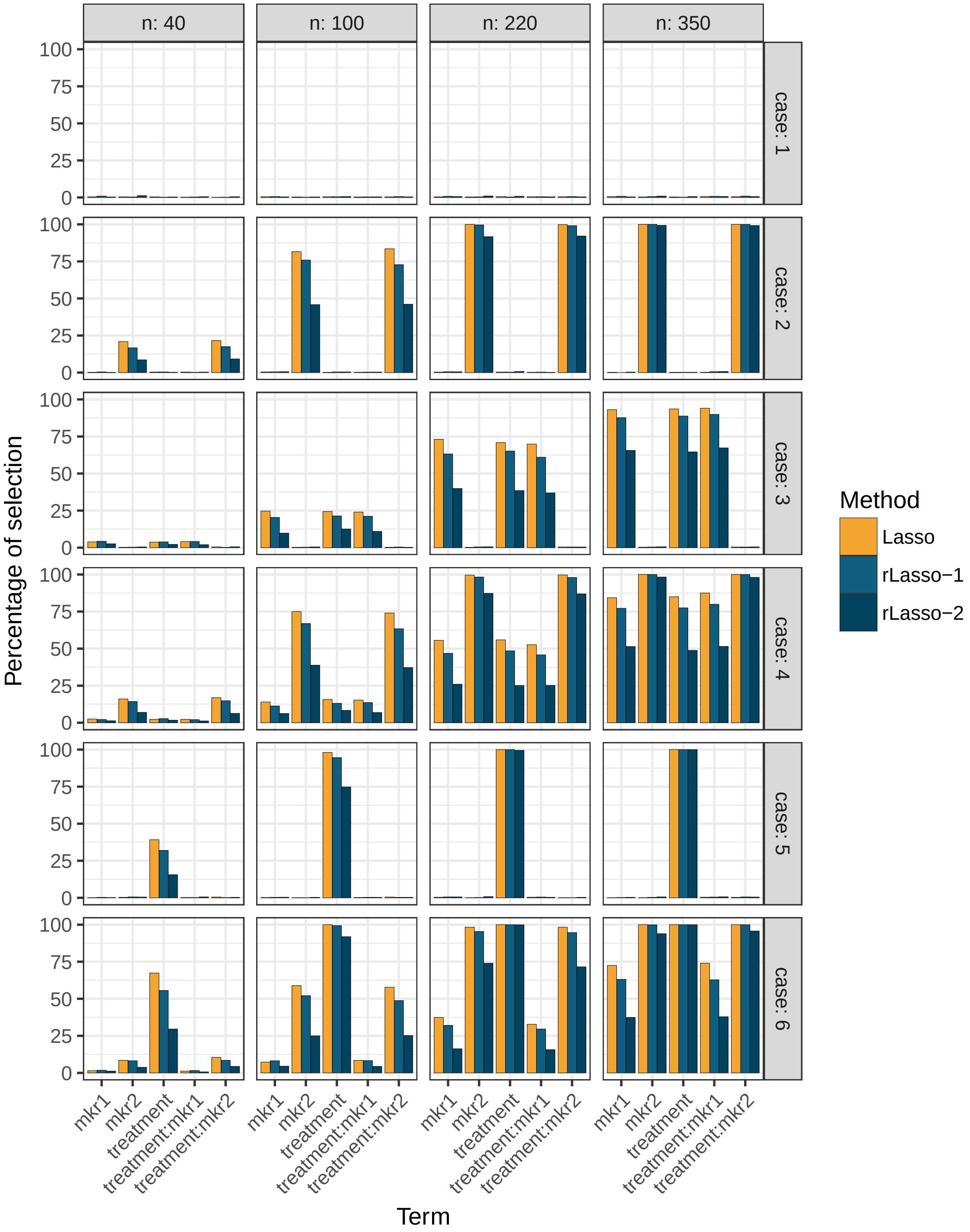

Figure 33: Selection probabilities for relevant terms in the model by method, case and sample size for 1000 biomarkers.

6.2 Coverage of confidence intervals

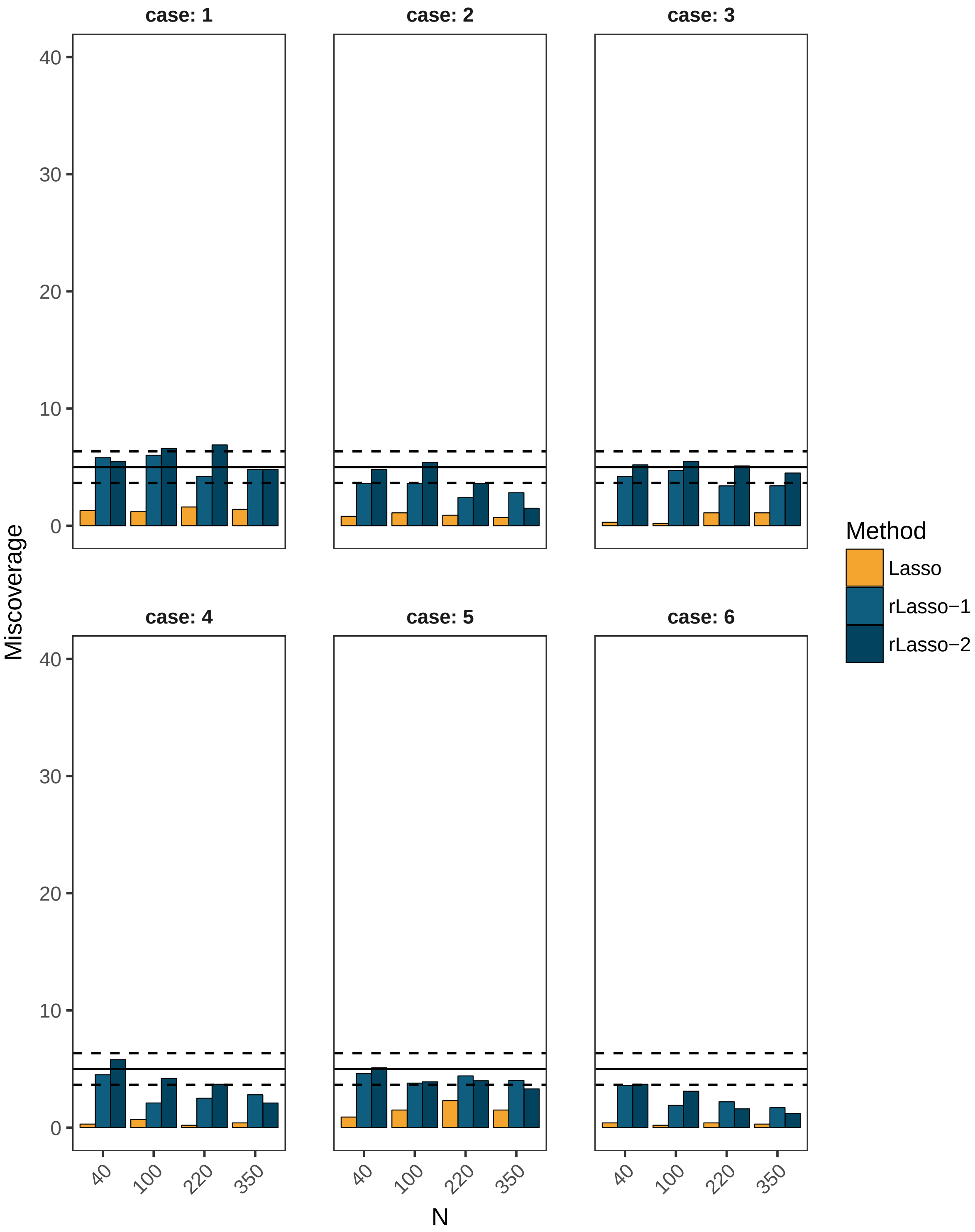

Figure 34: Average miscoverage of the confidence intervals for the PITE by method, case and sample size for 1000 biomarkers. The line at 5% indicates the target miscoverage.

6.3 Bias, MSE and width

Table 27: Diagnostic measures for case 1 with 1000 biomarkers. Columns 4 and 5 show the bias and the $\sqrt{MSE}$ for the point estimate of the PITE. The sixth column shows the median width of the confidence intervals for the PITE, and the last columns show the proportion of subjects in the identified subgroup when considering the using the limits of the confidence intervals and the point estimates. Since methods reduced and reduced-Scheffe have the same point estimate, bias and MSE are equal.

Case	N	Method	Bias	$\sqrt{MSE}$	Width	% in $\hat{B}_l$	% in $\hat{B}$	% in $\hat{B}_u$	% in B
1	40	Lasso	0.00	0.21	6.65	0.60	45.1	91.20	0
1	40	rLasso-1	-0.01	0.91	4.40	2.70	48.6	94.89	0
1	40	rLasso-2	0.05	0.80	2.97	2.70	52.1	93.20	0
1	100	Lasso	-0.01	0.17	4.89	0.40	46.0	96.20	0
1	100	rLasso-1	-0.01	0.78	3.18	3.11	48.7	95.89	0
1	100	rLasso-2	0.00	0.71	2.06	3.60	46.7	96.10	0
1	220	Lasso	-0.01	0.12	3.57	0.50	46.4	97.00	0
1	220	rLasso-1	-0.05	0.68	2.35	1.91	47.1	97.39	0
1	220	rLasso-2	-0.04	0.58	1.59	3.00	46.0	95.30	0
1	350	Lasso	0.00	0.10	3.24	0.80	50.9	97.90	0
1	350	rLasso-1	0.03	0.59	1.89	2.71	52.8	97.70	0
1	350	rLasso-2	0.01	0.51	1.32	2.40	51.2	97.20	0

Table 28: Diagnostic measures for case 2 with 1000 biomarkers. Columns 4 and 5 show the bias and the $\sqrt{MSE}$ for the point estimate of the PITE. The sixth column shows the median width of the confidence intervals for the PITE, and the last columns show the proportion of subjects in the identified subgroup when considering the using the limits of the confidence intervals and the point estimates. Since methods reduced and reduced-Scheffe have the same point estimate, bias and MSE are equal.

Case	N	Method	Bias	$\sqrt{MSE}$	Width	% in $\hat{B}_l$	% in $\hat{B}$	% in $\hat{B}_u$	% in B
2	40	Lasso	0.00	0.85	7.62	0.80	44.6	91.80	49.2
2	40	rLasso-1	0.00	1.23	4.79	2.10	48.0	94.20	49.2
2	40	rLasso-2	0.04	1.20	3.38	2.90	49.8	94.00	49.2
2	100	Lasso	0.02	0.70	4.48	7.70	48.1	91.10	48.4
2	100	rLasso-1	0.02	0.91	3.14	8.62	49.5	89.78	48.4
2	100	rLasso-2	-0.01	0.95	2.49	8.70	46.7	91.20	48.4
2	220	Lasso	-0.02	0.47	1.95	23.60	51.3	78.30	51.9
2	220	rLasso-1	-0.02	0.58	1.86	23.50	51.2	79.60	51.9
2	220	rLasso-2	-0.01	0.59	1.67	24.70	50.7	79.00	51.9
2	350	Lasso	-0.01	0.36	1.30	30.40	51.3	72.40	50.9
2	350	rLasso-1	0.02	0.46	1.44	28.04	50.9	73.17	50.9
2	350	rLasso-2	-0.01	0.45	1.32	28.70	50.6	73.00	50.9

Table 29: Diagnostic measures for case 3 with 1000 biomarkers. Columns 4 and 5 show the bias and the $\sqrt{MSE}$ for the point estimate of the PITE. The sixth column shows the median width of the confidence intervals for the PITE, and the last columns show the proportion of subjects in the identified subgroup when considering the using the limits of the confidence intervals and the point estimates. Since methods reduced and reduced-Scheffe have the same point estimate, bias and MSE are equal.

Case	N	Method	Bias	$\sqrt{MSE}$	Width	% in $\hat{B}_l$	% in $\hat{B}$	% in $\hat{B}_u$	% in B
3	40	Lasso	-0.53	0.77	7.62	0.10	48.2	92.90	53
3	40	rLasso-1	-0.50	1.20	4.89	2.00	52.2	95.50	53
3	40	rLasso-2	-0.53	1.15	3.22	2.60	47.7	93.30	53
3	100	Lasso	-0.43	0.66	5.56	1.30	57.7	96.80	47
3	100	rLasso-1	-0.31	0.99	3.51	3.91	57.3	95.79	47
3	100	rLasso-2	-0.40	1.00	2.32	5.20	51.9	96.00	47
3	220	Lasso	-0.38	0.58	3.76	7.60	66.0	98.20	48
3	220	rLasso-1	-0.23	0.80	2.46	13.21	62.1	96.60	48
3	220	rLasso-2	-0.32	0.80	1.77	13.90	58.8	95.70	48
3	350	Lasso	-0.32	0.48	2.77	17.80	71.6	99.70	49
3	350	rLasso-1	-0.08	0.62	1.93	25.65	70.5	98.70	49
3	350	rLasso-2	-0.19	0.65	1.48	26.10	67.3	95.50	49

Table 30: Diagnostic measures for case 4 with 1000 biomarkers. Columns 4 and 5 show the bias and the $\sqrt{MSE}$ for the point estimate of the PITE. The sixth column shows the median width of the confidence intervals for the PITE, and the last columns show the proportion of subjects in the identified subgroup when considering the using the limits of the confidence intervals and the point estimates. Since methods reduced and reduced-Scheffe have the same point estimate, bias and MSE are equal.

Case	N	Method	Bias	$\sqrt{MSE}$	Width	% in $\hat{B}_l$	% in $\hat{B}$	% in $\hat{B}_u$	% in B
4	40	Lasso	-0.49	1.14	7.76	1.00	48.9	92.50	64.6
4	40	rLasso-1	-0.42	1.52	5.45	3.20	50.5	94.39	64.6
4	40	rLasso-2	-0.47	1.48	3.72	2.80	50.1	93.30	64.6
4	100	Lasso	-0.42	0.97	5.68	5.50	51.8	95.80	64.8
4	100	rLasso-1	-0.33	1.12	3.66	8.81	54.1	93.69	64.8
4	100	rLasso-2	-0.40	1.24	2.67	7.00	50.9	92.70	64.8
4	220	Lasso	-0.42	0.80	3.12	17.10	58.0	90.70	67.7
4	220	rLasso-1	-0.26	0.81	2.25	24.37	59.9	86.96	67.7
4	220	rLasso-2	-0.39	0.89	1.93	23.50	56.4	85.40	67.7
4	350	Lasso	-0.37	0.65	1.83	30.00	59.5	87.60	66.2
4	350	rLasso-1	-0.13	0.56	1.69	34.70	61.6	85.50	66.2
4	350	rLasso-2	-0.28	0.67	1.53	35.70	58.1	81.60	66.2

Table 31: Diagnostic measures for case 5 with 1000 biomarkers. Columns 4 and 5 show the bias and the $\sqrt{MSE}$ for the point estimate of the PITE. The sixth column shows the median width of the confidence intervals for the PITE, and the last columns show the proportion of subjects in the identified subgroup when considering the using the limits of the confidence intervals and the point estimates. Since methods reduced and reduced-Scheffe have the same point estimate, bias and MSE are equal.

Case	N	Method	Bias	$\sqrt{MSE}$	Width	% in $\hat{B}_l$	% in $\hat{B}$	% in $\hat{B}_u$	% in B
5	40	Lasso	-0.91	0.95	7.10	3.40	61.5	93.90	100
5	40	rLasso-1	-0.79	1.26	4.89	6.41	58.4	96.19	100
5	40	rLasso-2	-0.89	1.30	3.35	4.70	53.4	94.10	100
5	100	Lasso	-0.61	0.65	3.80	29.80	96.5	99.90	100
5	100	rLasso-1	-0.22	0.83	3.06	34.70	83.7	99.60	100
5	100	rLasso-2	-0.32	0.87	2.33	37.34	81.7	99.60	100
5	220	Lasso	-0.42	0.45	2.21	51.40	99.9	100.00	100
5	220	rLasso-1	-0.10	0.60	2.00	56.67	93.7	99.70	100
5	220	rLasso-2	-0.08	0.53	1.61	66.90	95.5	100.00	100
5	350	Lasso	-0.33	0.35	1.84	59.80	100.0	100.00	100
5	350	rLasso-1	-0.04	0.50	1.55	68.54	96.9	100.00	100
5	350	rLasso-2	-0.04	0.46	1.31	79.48	97.2	100.00	100

Table 32: Diagnostic measures for case 6 with 1000 biomarkers. Columns 4 and 5 show the bias and the $\sqrt{MSE}$ for the point estimate of the PITE. The sixth column shows the median width of the confidence intervals for the PITE, and the last columns show the proportion of subjects in the identified subgroup when considering the using the limits of the confidence intervals and the point estimates. Since methods reduced and reduced-Scheffe have the same point estimate, bias and MSE are equal.

Case	N	Method	Bias	$\sqrt{MSE}$	Width	% in $\hat{B}_l$	% in $\hat{B}$	% in $\hat{B}_u$	% in B
6	40	Lasso	-1.23	1.64	9.20	8.20	78.0	98.4	90.9
6	40	rLasso-1	-0.83	1.77	5.77	13.60	69.2	97.2	90.9
6	40	rLasso-2	-1.11	1.93	4.39	11.10	58.8	95.8	90.9
6	100	Lasso	-0.72	1.19	3.43	46.30	98.3	100.0	89.8
6	100	rLasso-1	-0.13	1.14	3.36	50.90	89.5	99.6	89.8
6	100	rLasso-2	-0.23	1.25	2.90	55.66	90.2	99.6	89.8
6	220	Lasso	-0.53	0.91	1.66	67.00	99.4	100.0	91.1
6	220	rLasso-1	-0.02	0.66	1.87	67.80	92.7	99.9	91.1
6	220	rLasso-2	-0.09	0.83	1.92	69.70	93.6	100.0	91.1
6	350	Lasso	-0.43	0.73	1.39	70.60	99.5	99.9	92.5
6	350	rLasso-1	-0.04	0.45	1.34	74.40	92.5	99.6	92.5
6	350	rLasso-2	-0.04	0.56	1.47	74.40	94.4	100.0	92.5

6.4 Sensitivity and Specificity

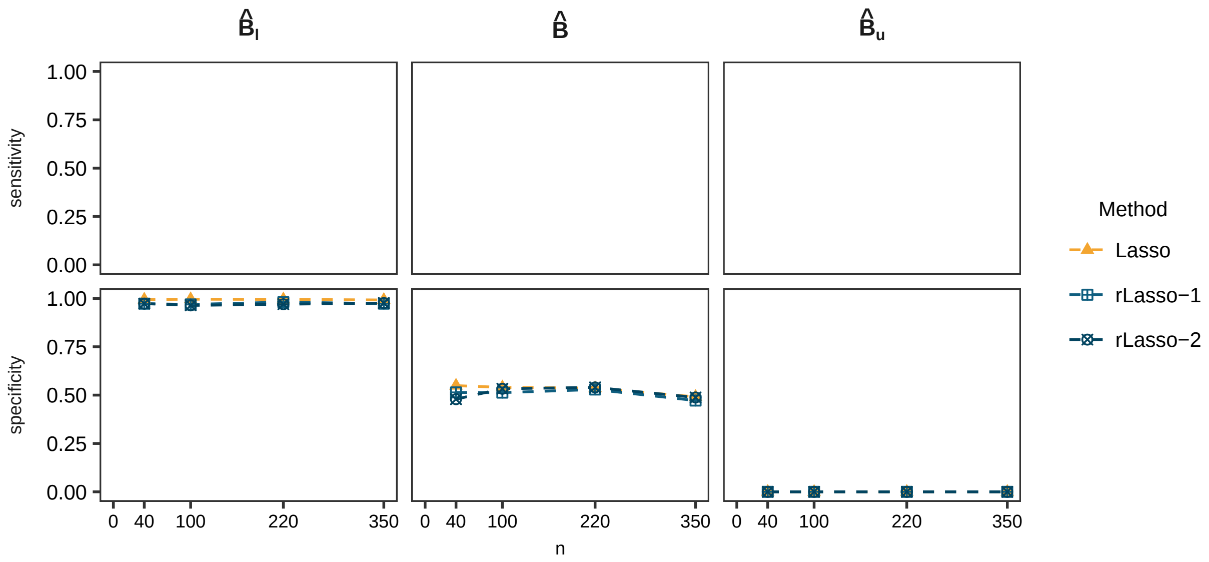

Figure 35: Specificity for identified subgroup by method and sample size for Case 1 and 1000 biomarkers. Sensitivity is not shown as there are no patients with $D(X) > 0$

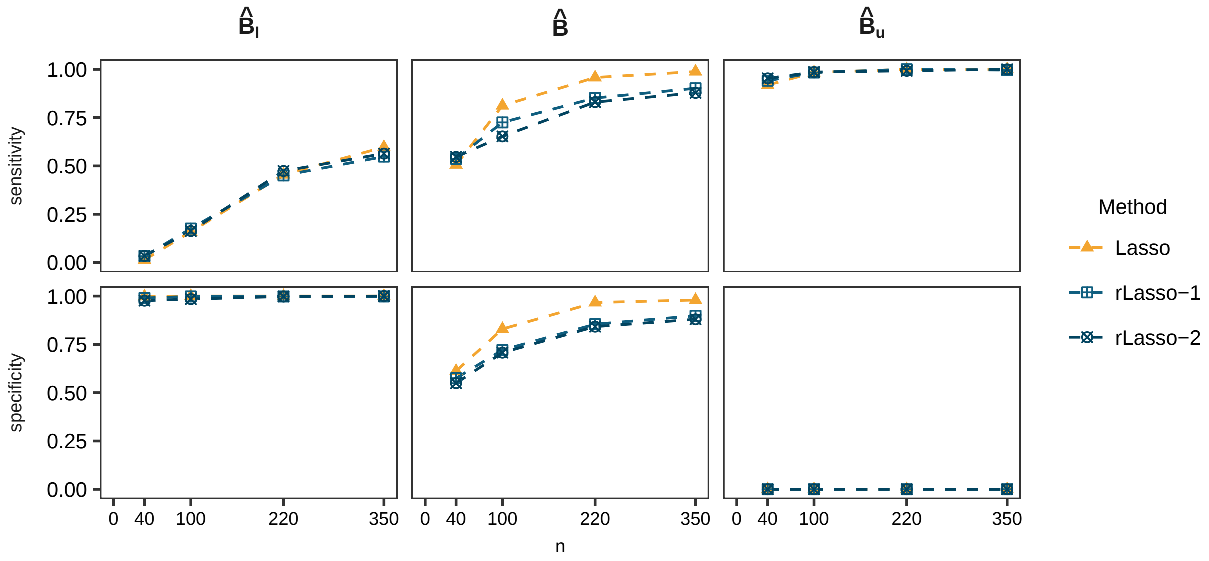

Figure 36: Sensitivity and specificity for identified subgroup by method and sample size for Case 2 and 1000 biomarkers.

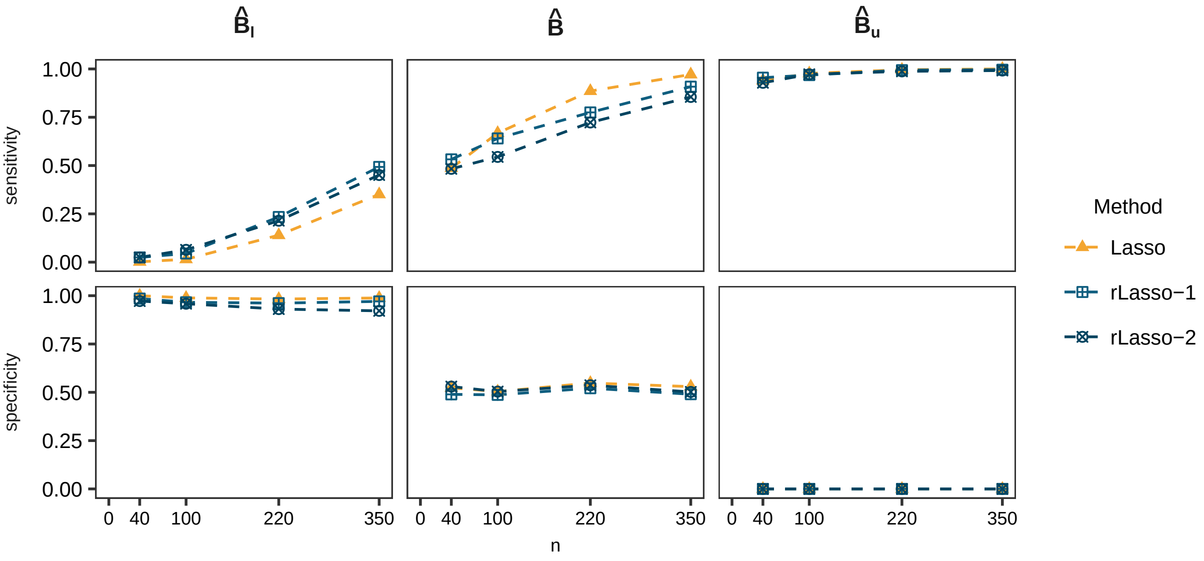

Figure 37: Sensitivity and specificity for identified subgroup by method and sample size for Case 3 and 1000 biomarkers.

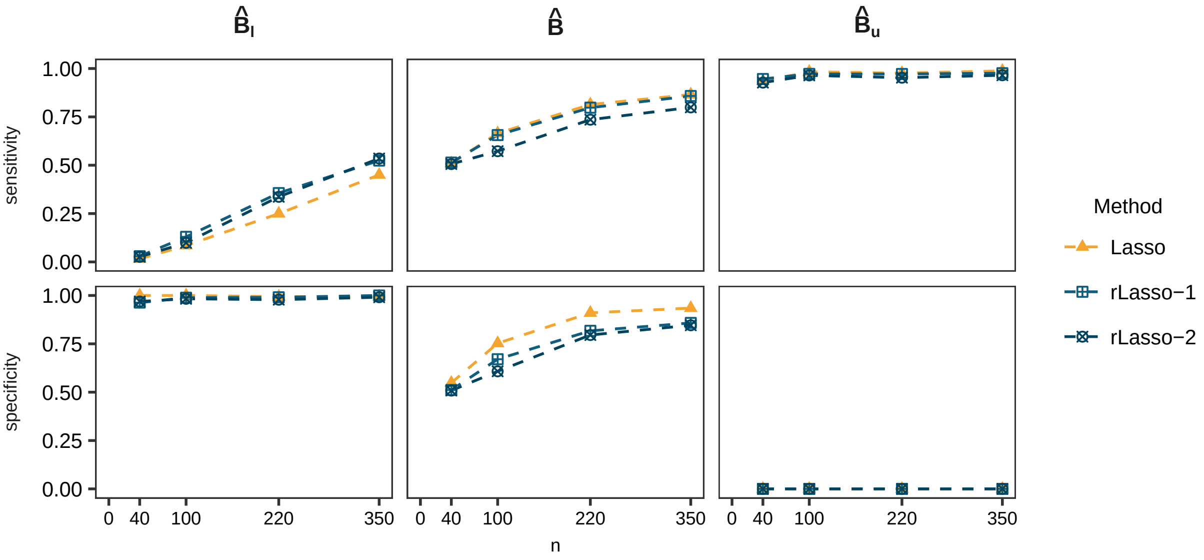

Figure 38: Sensitivity and specificity for identified subgroup by method and sample size for Case 4 and 1000 biomarkers.

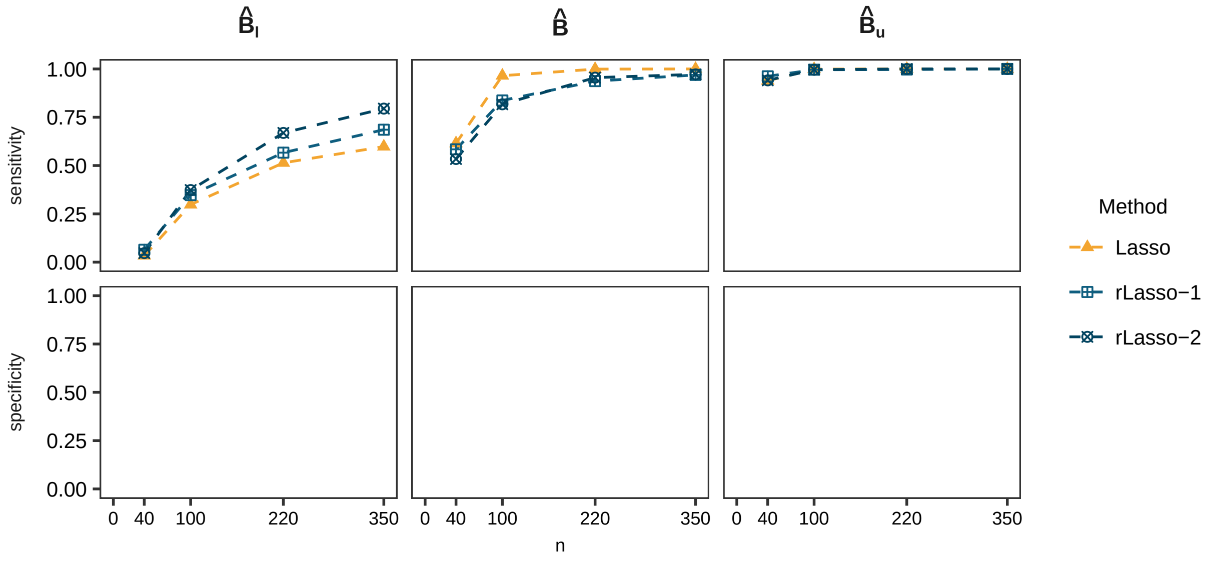

Figure 39: Sensitivity for identified subgroup by method and sample size for Case 5 and 1000 biomarkers. Specificity is not shown as all patients have $D(X) > 0$

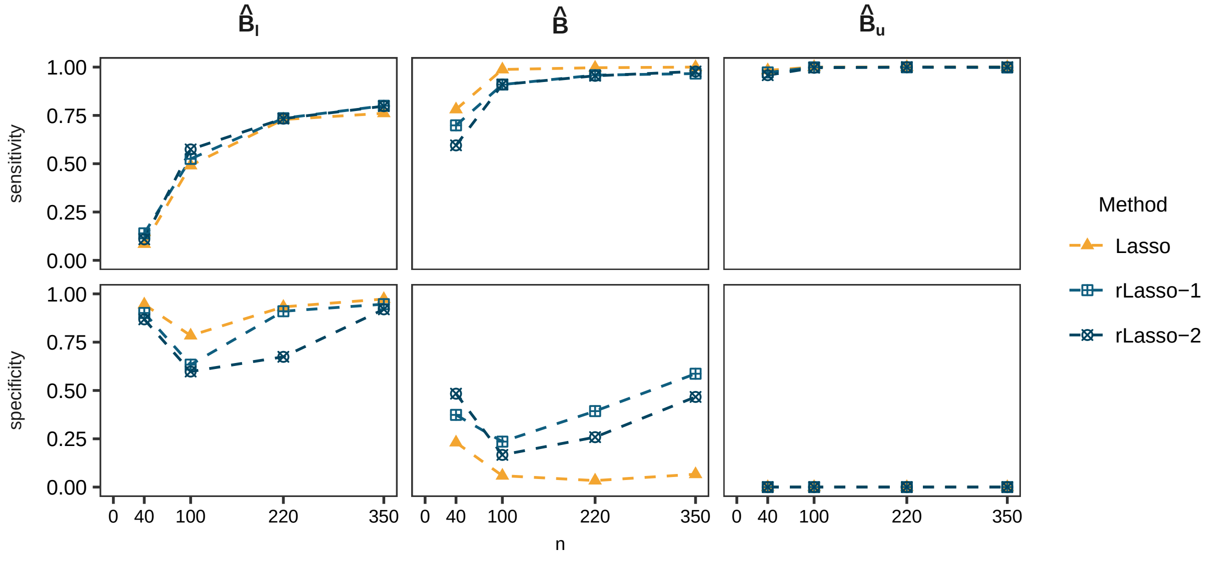

Figure 40: Sensitivity and specificity for identified subgroup by method and sample size for Case 6 and 1000 biomarkers.

7 Results for AR1 correlation structure

We now evaluate the case where the biomarkers are simulated using an AR1 correlation structure with $rho = 0.9$. In this case, all biomarkers are normally distributed. We use 10 biomarkers and the same effect as in the independent case with also 10 biomarkers.

7.1 Percent of selection

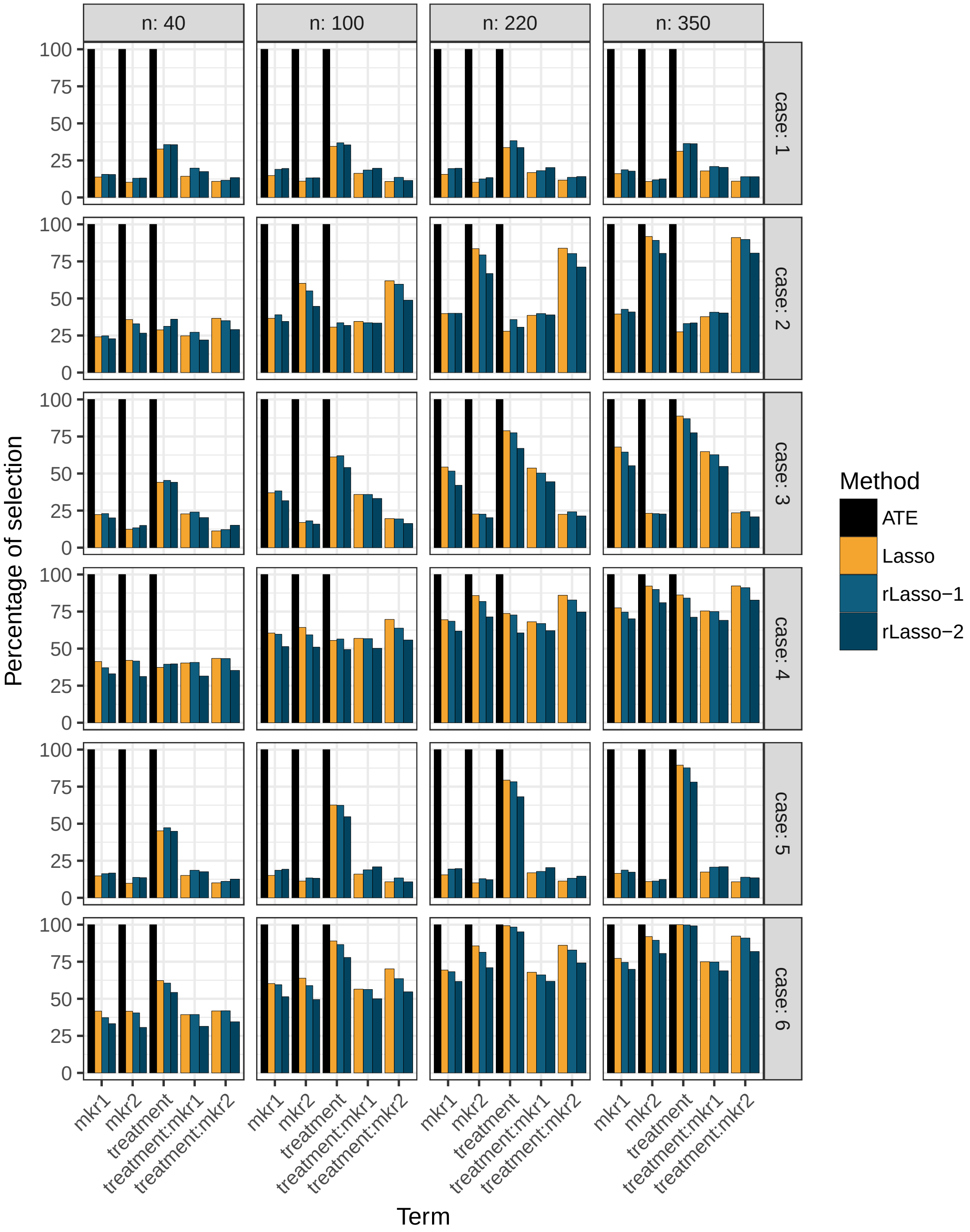

Figure 41: Selection probabilities for relevant terms in the model by method, case and sample size for 10 biomarkers. The full model is not represented here as all variables are included 100% of the times. The reduced model is not represented as it has the same values as the Lasso

7.2 Coverage of confidence intervals

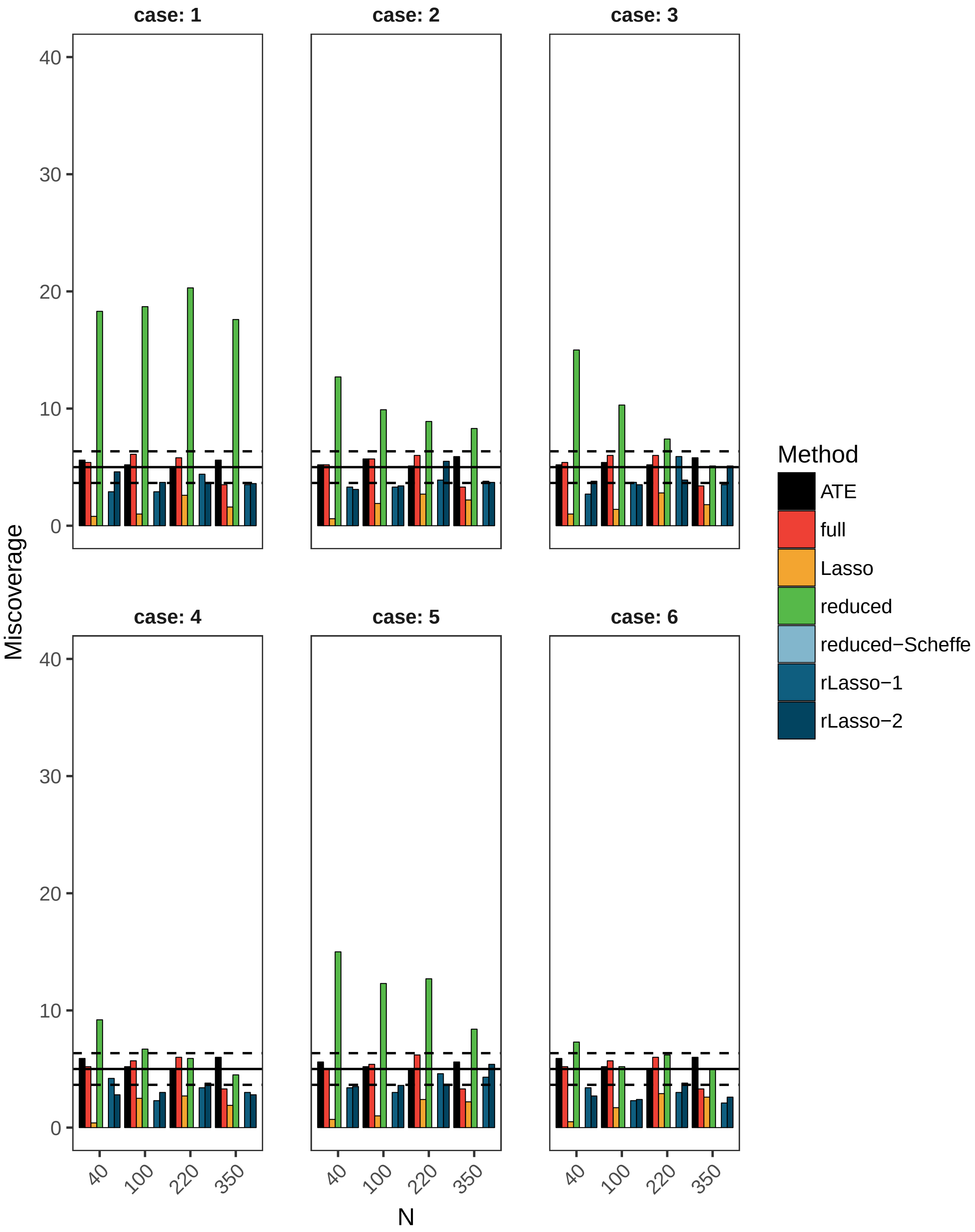

Figure 42: Average miscoverage of the confidence intervals for the PITE by method, case and sample size for 10 biomarkers. The line at 5% indicates the target miscoverage.

7.3 Bias, MSE and width

Table 33: Diagnostic measures for case 1 with 10 biomarkers. Columns 4 and 5 show the bias and the $\sqrt{MSE}$ for the point estimate of the PITE. The sixth column shows the median width of the confidence intervals for the PITE, and the last columns show the proportion of subjects in the identified subgroup when considering the using the limits of the confidence intervals and the point estimates. Since methods reduced and reduced-Scheffe have the same point estimate, bias and MSE are equal.

Case	N	Method	Bias	$\sqrt{MSE}$	Width	% in $\hat{B}_l$	% in $\hat{B}$	% in $\hat{B}_u$	% in B
1	40	ATE	0.02	0.38	1.49	3.1	52.5	97.5	0
1	40	full	-0.03	1.63	6.18	2.6	49.9	97.2	0
1	40	Lasso	0.00	0.29	3.26	0.4	44.7	82.9	0
1	40	reduced	0.01	0.62	1.17	9.7	43.5	74.7	0
1	40	reduced-Scheffe	0.01	0.62	4.00	0.0	43.5	83.3	0
1	40	rLasso-1	0.00	0.60	2.29	1.7	45.3	87.3	0
1	40	rLasso-2	-0.02	0.55	1.74	2.4	42.9	86.1	0
1	100	ATE	0.01	0.22	0.84	2.8	51.2	97.6	0
1	100	full	0.00	0.79	2.89	2.8	52.1	96.7	0
1	100	Lasso	0.00	0.17	2.03	0.5	42.7	81.4	0
1	100	reduced	0.00	0.39	0.78	8.9	43.4	72.1	0
1	100	reduced-Scheffe	0.00	0.39	2.38	0.0	43.4	81.9	0
1	100	rLasso-1	0.00	0.38	1.48	1.8	46.6	87.5	0
1	100	rLasso-2	0.01	0.35	1.10	2.0	45.8	86.0	0
1	220	ATE	0.00	0.14	0.54	2.6	49.2	97.6	0
1	220	full	0.00	0.48	1.82	2.8	50.1	97.0	0
1	220	Lasso	0.00	0.14	1.29	1.1	42.1	82.3	0
1	220	reduced	0.01	0.28	0.54	10.5	42.0	74.0	0
1	220	reduced-Scheffe	0.01	0.28	1.60	0.0	42.0	83.8	0
1	220	rLasso-1	0.01	0.28	0.99	2.3	46.6	87.0	0
1	220	rLasso-2	0.01	0.25	0.76	1.9	44.4	85.9	0
1	350	ATE	0.00	0.11	0.43	2.5	47.7	96.9	0
1	350	full	-0.01	0.36	1.38	1.8	47.3	98.3	0
1	350	Lasso	0.00	0.10	1.02	0.9	38.9	82.6	0
1	350	reduced	-0.01	0.21	0.41	8.2	38.8	73.9	0
1	350	reduced-Scheffe	-0.01	0.21	1.22	0.0	38.8	83.3	0
1	350	rLasso-1	-0.01	0.20	0.77	2.1	41.5	85.8	0
1	350	rLasso-2	-0.01	0.18	0.58	2.1	40.4	85.3	0

Table 34: Diagnostic measures for case 2 with 10 biomarkers. Columns 4 and 5 show the bias and the $\sqrt{MSE}$ for the point estimate of the PITE. The sixth column shows the median width of the confidence intervals for the PITE, and the last columns show the proportion of subjects in the identified subgroup when considering the using the limits of the confidence intervals and the point estimates. Since methods reduced and reduced-Scheffe have the same point estimate, bias and MSE are equal.

Case	N	Method	Bias	$\sqrt{MSE}$	Width	% in $\hat{B}_l$	% in $\hat{B}$	% in $\hat{B}_u$	% in B
2	40	ATE	0.04	0.71	1.55	2.6	51.8	97.4	46.5
2	40	full	-0.05	1.65	6.22	4.1	47.1	96.7	46.5
2	40	Lasso	0.02	0.44	4.34	2.8	46.0	92.0	46.5
2	40	reduced	0.00	0.64	1.41	17.8	45.7	75.0	46.5
2	40	reduced-Scheffe	0.00	0.64	4.79	0.2	45.7	94.2	46.5
2	40	rLasso-1	0.02	0.64	2.82	3.9	47.9	91.5	46.5
2	40	rLasso-2	0.00	0.63	2.04	5.5	45.5	89.6	46.5
2	100	ATE	-0.01	0.62	0.87	2.8	53.4	97.1	50.9
2	100	full	0.01	0.77	2.90	6.3	51.5	93.4	50.9
2	100	Lasso	0.00	0.32	2.75	11.1	50.4	90.7	50.9
2	100	reduced	0.01	0.42	1.03	26.9	50.8	74.3	50.9
2	100	reduced-Scheffe	0.01	0.42	3.13	0.0	50.8	99.5	50.9
2	100	rLasso-1	0.02	0.43	1.93	11.3	49.3	90.7	50.9
2	100	rLasso-2	0.02	0.43	1.43	16.9	51.2	88.0	50.9
2	220	ATE	0.01	0.62	0.57	2.6	48.9	97.5	48.8
2	220	full	0.01	0.48	1.81	10.5	51.1	88.4	48.8
2	220	Lasso	0.01	0.24	1.75	16.4	49.4	83.4	48.8
2	220	reduced	0.01	0.28	0.77	32.4	49.0	68.2	48.8
2	220	reduced-Scheffe	0.01	0.28	2.30	0.5	49.0	99.0	48.8
2	220	rLasso-1	0.01	0.29	1.34	19.1	49.6	81.1	48.8
2	220	rLasso-2	0.01	0.29	1.04	23.5	49.7	76.8	48.8
2	350	ATE	-0.02	0.61	0.44	2.7	46.5	96.8	49.4
2	350	full	-0.01	0.36	1.39	14.8	50.9	85.2	49.4
2	350	Lasso	-0.01	0.20	1.26	21.2	50.3	77.2	49.4
2	350	reduced	-0.01	0.22	0.61	35.9	50.8	64.6	49.4
2	350	reduced-Scheffe	-0.01	0.22	1.79	4.4	50.8	95.0	49.4
2	350	rLasso-1	-0.02	0.22	1.06	25.5	50.9	76.2	49.4
2	350	rLasso-2	-0.01	0.23	0.85	29.0	50.6	71.6	49.4

Table 35: Diagnostic measures for case 3 with 10 biomarkers. Columns 4 and 5 show the bias and the $\sqrt{MSE}$ for the point estimate of the PITE. The sixth column shows the median width of the confidence intervals for the PITE, and the last columns show the proportion of subjects in the identified subgroup when considering the using the limits of the confidence intervals and the point estimates. Since methods reduced and reduced-Scheffe have the same point estimate, bias and MSE are equal.

Case	N	Method	Bias	$\sqrt{MSE}$	Width	% in $\hat{B}_l$	% in $\hat{B}$	% in $\hat{B}_u$	% in B
3	40	ATE	0.03	0.47	1.51	11.20	76.9	99.50	83.0
3	40	full	-0.05	1.67	6.21	4.60	55.5	98.30	83.0
3	40	Lasso	-0.15	0.38	3.77	2.30	56.3	88.20	83.0
3	40	reduced	-0.06	0.64	1.33	18.70	56.0	82.50	83.0
3	40	reduced-Scheffe	-0.06	0.64	4.57	0.20	56.0	88.80	83.0
3	40	rLasso-1	-0.08	0.63	2.31	3.30	56.2	89.39	83.0
3	40	rLasso-2	-0.09	0.59	1.87	3.10	56.6	89.80	83.0
3	100	ATE	0.00	0.33	0.84	25.70	88.0	99.80	84.5
3	100	full	-0.01	0.77	2.90	7.20	64.2	97.80	84.5
3	100	Lasso	-0.13	0.30	2.22	5.50	68.9	94.40	84.5
3	100	reduced	-0.02	0.43	0.93	30.60	68.1	88.40	84.5
3	100	reduced-Scheffe	-0.02	0.43	2.84	0.00	68.1	95.00	84.5
3	100	rLasso-1	-0.02	0.43	1.66	8.11	69.8	94.29	84.5
3	100	rLasso-2	-0.07	0.43	1.22	9.00	65.3	92.40	84.5
3	220	ATE	0.00	0.30	0.55	43.10	96.3	100.00	81.8
3	220	full	0.01	0.48	1.80	10.80	68.8	98.40	81.8
3	220	Lasso	-0.11	0.26	1.64	12.00	74.5	97.00	81.8
3	220	reduced	-0.01	0.31	0.72	40.10	75.1	92.70	81.8
3	220	reduced-Scheffe	-0.01	0.31	2.14	0.00	75.1	98.70	81.8
3	220	rLasso-1	-0.01	0.32	1.17	16.22	73.8	96.30	81.8
3	220	rLasso-2	-0.05	0.32	0.88	18.60	70.2	93.10	81.8
3	350	ATE	-0.01	0.28	0.43	61.00	98.4	100.00	82.8
3	350	full	-0.01	0.36	1.39	14.60	72.3	98.50	82.8
3	350	Lasso	-0.11	0.22	1.22	18.90	78.6	98.30	82.8
3	350	reduced	-0.02	0.23	0.61	46.50	77.8	96.00	82.8
3	350	reduced-Scheffe	-0.02	0.23	1.80	0.30	77.8	99.50	82.8
3	350	rLasso-1	-0.03	0.24	0.93	22.82	76.8	96.90	82.8
3	350	rLasso-2	-0.05	0.25	0.74	27.20	75.8	95.30	82.8

Table 36: Diagnostic measures for case 4 with 10 biomarkers. Columns 4 and 5 show the bias and the $\sqrt{MSE}$ for the point estimate of the PITE. The sixth column shows the median width of the confidence intervals for the PITE, and the last columns show the proportion of subjects in the identified subgroup when considering the using the limits of the confidence intervals and the point estimates. Since methods reduced and reduced-Scheffe have the same point estimate, bias and MSE are equal.

Case	N	Method	Bias	$\sqrt{MSE}$	Width	% in $\hat{B}_l$	% in $\hat{B}$	% in $\hat{B}_u$	% in B
4	40	ATE	0.06	0.92	1.60	10.5	76.2	99.4	59.1
4	40	full	-0.04	1.64	6.20	5.6	52.4	96.6	59.1
4	40	Lasso	-0.14	0.54	5.40	4.8	53.9	94.7	59.1
4	40	reduced	-0.06	0.67	1.60	28.5	54.9	78.9	59.1
4	40	reduced-Scheffe	-0.06	0.67	5.42	0.4	54.9	97.6	59.1
4	40	rLasso-1	-0.06	0.68	3.37	6.0	55.1	93.2	59.1
4	40	rLasso-2	-0.09	0.69	2.44	8.3	54.5	88.7	59.1
4	100	ATE	-0.02	0.83	0.90	21.7	85.5	99.9	63.5
4	100	full	0.01	0.77	2.89	14.4	60.4	93.7	63.5
4	100	Lasso	-0.14	0.39	2.93	17.2	58.0	90.9	63.5
4	100	reduced	-0.02	0.44	1.17	39.5	61.0	80.6	63.5
4	100	reduced-Scheffe	-0.02	0.44	3.59	1.4	61.0	99.7	63.5
4	100	rLasso-1	-0.03	0.45	2.23	17.7	60.3	89.9	63.5
4	100	rLasso-2	-0.07	0.46	1.66	24.0	59.9	86.0	63.5
4	220	ATE	0.01	0.87	0.59	39.9	94.7	100.0	61.8
4	220	full	0.01	0.48	1.81	25.0	61.6	88.4	61.8
4	220	Lasso	-0.12	0.31	1.82	23.9	57.6	86.4	61.8
4	220	reduced	-0.01	0.31	0.88	43.5	61.0	76.7	61.8
4	220	reduced-Scheffe	-0.01	0.31	2.61	7.6	61.0	97.6	61.8
4	220	rLasso-1	-0.01	0.32	1.53	25.4	60.2	83.5	61.8
4	220	rLasso-2	-0.05	0.34	1.22	31.8	59.3	79.6	61.8
4	350	ATE	-0.02	0.85	0.46	54.4	98.1	100.0	60.9
4	350	full	-0.01	0.36	1.39	30.2	61.8	85.4	60.9
4	350	Lasso	-0.13	0.26	1.30	30.0	57.4	82.0	60.9
4	350	reduced	-0.03	0.24	0.73	47.8	60.5	75.2	60.9
4	350	reduced-Scheffe	-0.03	0.24	2.16	16.7	60.5	96.7	60.9
4	350	rLasso-1	-0.04	0.25	1.21	33.0	60.4	82.1	60.9
4	350	rLasso-2	-0.06	0.27	1.00	37.7	59.5	76.5	60.9

Table 37: Diagnostic measures for case 5 with 10 biomarkers. Columns 4 and 5 show the bias and the $\sqrt{MSE}$ for the point estimate of the PITE. The sixth column shows the median width of the confidence intervals for the PITE, and the last columns show the proportion of subjects in the identified subgroup when considering the using the limits of the confidence intervals and the point estimates. Since methods reduced and reduced-Scheffe have the same point estimate, bias and MSE are equal.

Case	N	Method	Bias	$\sqrt{MSE}$	Width	% in $\hat{B}_l$	% in $\hat{B}$	% in $\hat{B}_u$	% in B
5	40	ATE	0.02	0.38	1.49	11.40	76.0	99.7	100
5	40	full	-0.02	1.62	6.17	4.40	55.7	98.6	100
5	40	Lasso	-0.15	0.34	3.28	1.70	60.1	86.7	100
5	40	reduced	-0.04	0.64	1.26	17.40	58.4	82.5	100
5	40	reduced-Scheffe	-0.04	0.64	4.31	0.10	58.4	87.0	100
5	40	rLasso-1	-0.07	0.63	2.28	3.70	59.4	89.3	100
5	40	rLasso-2	-0.08	0.57	1.78	3.60	58.1	87.0	100
5	100	ATE	0.01	0.22	0.84	26.10	88.0	99.8	100
5	100	full	0.00	0.77	2.89	5.20	63.1	98.5	100
5	100	Lasso	-0.13	0.25	2.01	5.00	68.8	90.5	100
5	100	reduced	-0.03	0.42	0.85	26.90	68.3	86.8	100
5	100	reduced-Scheffe	-0.03	0.42	2.61	0.00	68.3	90.7	100
5	100	rLasso-1	-0.03	0.40	1.44	7.10	69.0	93.0	100
5	100	rLasso-2	-0.07	0.40	1.14	8.20	64.2	89.4	100
5	220	ATE	0.00	0.14	0.54	43.10	96.4	100.0	100
5	220	full	0.01	0.48	1.79	10.10	72.5	99.4	100
5	220	Lasso	-0.11	0.21	1.36	13.90	79.4	95.1	100
5	220	reduced	-0.01	0.30	0.61	40.50	78.4	93.9	100
5	220	reduced-Scheffe	-0.01	0.30	1.80	0.00	78.4	95.2	100
5	220	rLasso-1	-0.01	0.30	0.99	16.30	78.8	96.1	100
5	220	rLasso-2	-0.03	0.29	0.80	19.30	76.5	94.4	100
5	350	ATE	0.00	0.11	0.43	60.80	98.5	100.0	100
5	350	full	-0.01	0.36	1.39	10.10	74.9	99.6	100
5	350	Lasso	-0.10	0.17	0.93	19.80	86.9	96.7	100
5	350	reduced	-0.02	0.22	0.48	48.30	85.0	96.3	100
5	350	reduced-Scheffe	-0.02	0.22	1.42	0.10	85.0	96.7	100
5	350	rLasso-1	-0.03	0.22	0.77	24.42	84.0	97.0	100
5	350	rLasso-2	-0.04	0.22	0.60	25.30	81.4	94.8	100

Table 38: Diagnostic measures for case 6 with 10 biomarkers. Columns 4 and 5 show the bias and the $\sqrt{MSE}$ for the point estimate of the PITE. The sixth column shows the median width of the confidence intervals for the PITE, and the last columns show the proportion of subjects in the identified subgroup when considering the using the limits of the confidence intervals and the point estimates. Since methods reduced and reduced-Scheffe have the same point estimate, bias and MSE are equal.

Case	N	Method	Bias	$\sqrt{MSE}$	Width	% in $\hat{B}_l$	% in $\hat{B}$	% in $\hat{B}_u$	% in B
6	40	ATE	0.07	0.92	1.60	24.2	89.2	100.0	71.3
6	40	full	-0.04	1.64	6.19	6.3	59.4	97.5	71.3
6	40	Lasso	-0.26	0.61	5.90	5.7	63.6	96.5	71.3
6	40	reduced	-0.07	0.70	1.73	33.8	64.2	87.8	71.3
6	40	reduced-Scheffe	-0.07	0.70	5.84	0.5	64.2	98.2	71.3
6	40	rLasso-1	-0.09	0.72	3.48	8.5	64.5	94.9	71.3
6	40	rLasso-2	-0.13	0.73	2.48	11.6	63.1	91.2	71.3
6	100	ATE	-0.02	0.83	0.90	61.2	98.2	100.0	73.5
6	100	full	0.00	0.77	2.89	20.1	68.7	96.2	73.5
6	100	Lasso	-0.20	0.44	2.69	25.8	69.7	96.1	73.5
6	100	reduced	0.00	0.45	1.24	47.7	72.0	91.5	73.5
6	100	reduced-Scheffe	0.00	0.45	3.76	2.2	72.0	100.0	73.5
6	100	rLasso-1	-0.01	0.46	2.28	26.6	72.9	95.4	73.5
6	100	rLasso-2	-0.06	0.51	1.73	32.3	70.9	94.1	73.5
6	220	ATE	0.01	0.87	0.59	92.6	100.0	100.0	71.0
6	220	full	0.01	0.48	1.81	34.2	71.3	92.5	71.0
6	220	Lasso	-0.14	0.33	1.63	37.7	70.1	92.4	71.0
6	220	reduced	0.01	0.30	0.91	55.0	71.7	86.5	71.0
6	220	reduced-Scheffe	0.01	0.30	2.71	16.6	71.7	99.9	71.0
6	220	rLasso-1	0.01	0.31	1.51	40.4	72.0	92.6	71.0
6	220	rLasso-2	0.00	0.33	1.23	45.4	72.1	91.1	71.0
6	350	ATE	-0.02	0.85	0.46	98.9	100.0	100.0	72.7
6	350	full	-0.01	0.36	1.39	44.0	71.6	91.8	72.7
6	350	Lasso	-0.13	0.27	1.19	44.2	69.5	89.0	72.7
6	350	reduced	-0.02	0.23	0.75	57.7	71.7	85.0	72.7
6	350	reduced-Scheffe	-0.02	0.23	2.20	25.5	71.7	99.0	72.7
6	350	rLasso-1	-0.02	0.24	1.18	47.2	71.3	90.9	72.7
6	350	rLasso-2	-0.01	0.24	1.03	51.9	72.6	89.3	72.7

7.4 Sensitivity and Specificity

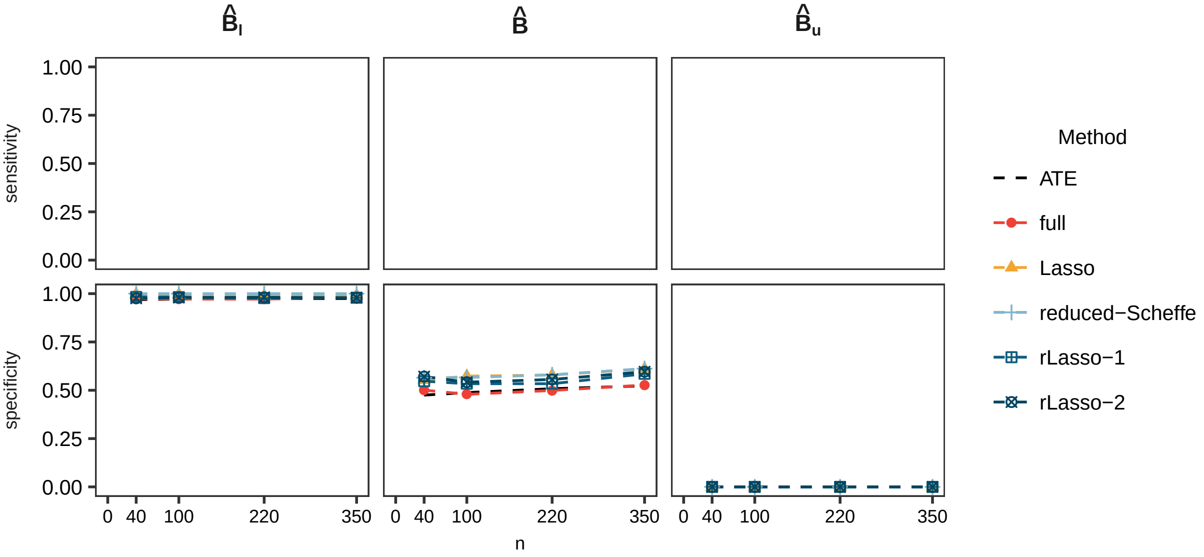

Figure 43: Specificity for identified subgroup by method and sample size for Case 1 and 10 biomarkers. Sensitivity is not shown as there are no patients with $D(X) > 0$

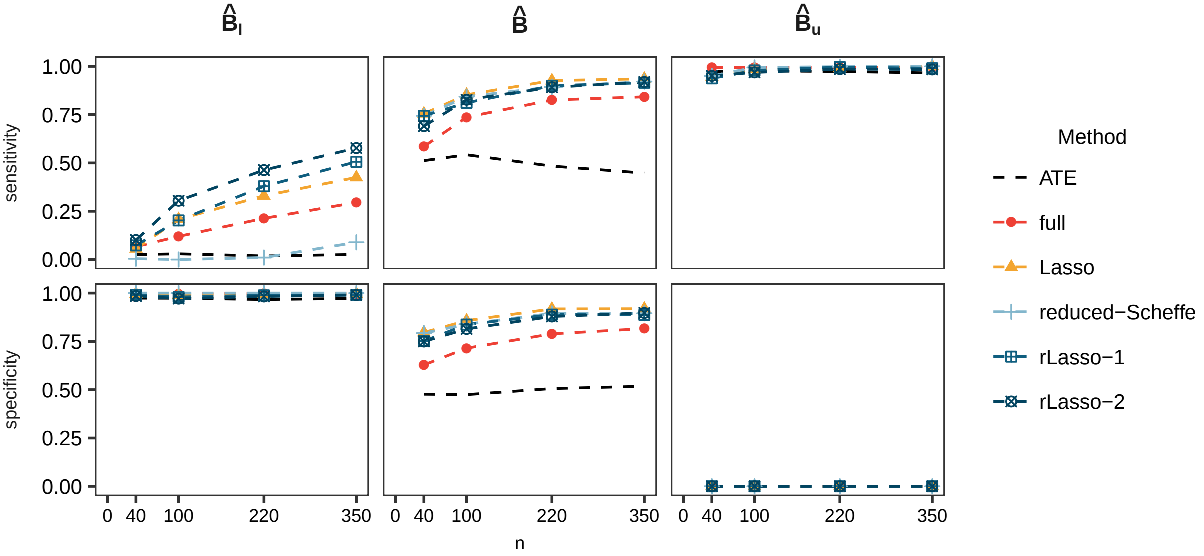

Figure 44: Sensitivity and specificity for identified subgroup by method and sample size for Case 2 and 10 biomarkers.

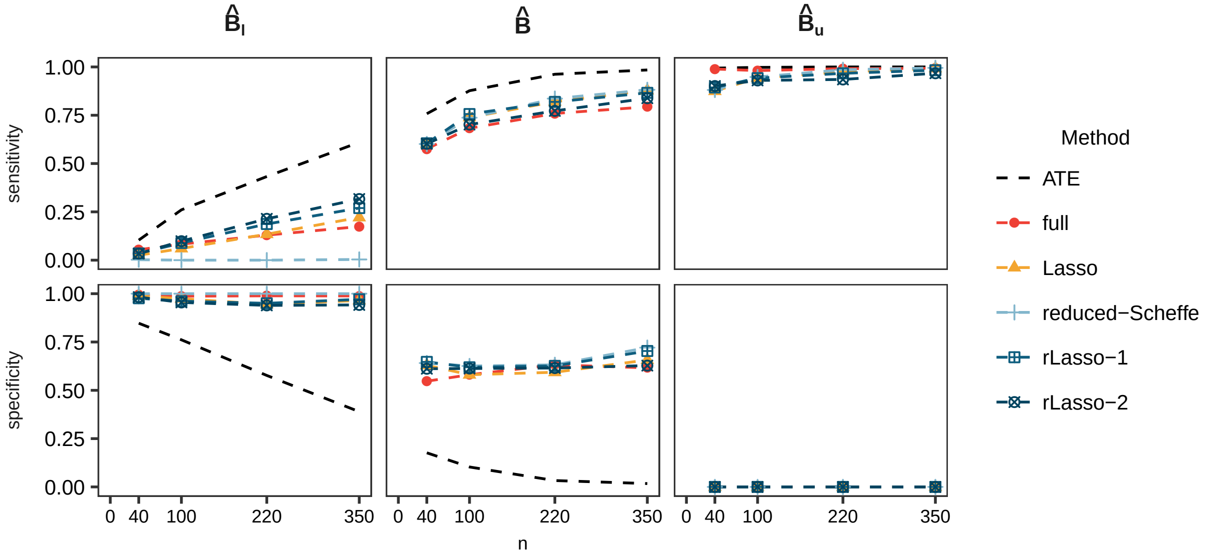

Figure 45: Sensitivity and specificity for identified subgroup by method and sample size for Case 3 and 10 biomarkers.

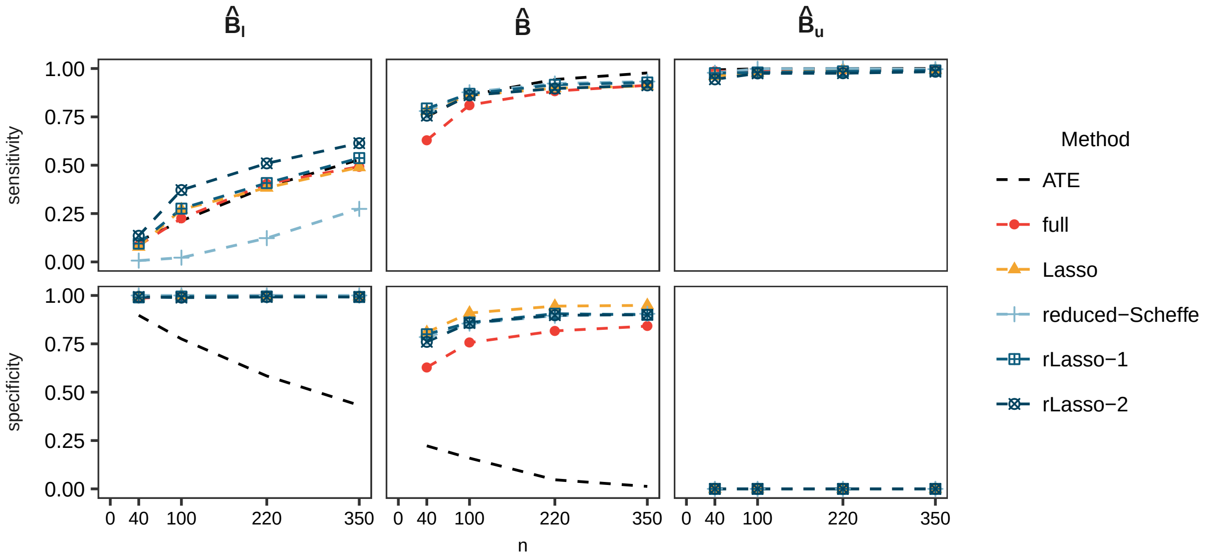

Figure 46: Sensitivity and specificity for identified subgroup by method and sample size for Case 4 and 10 biomarkers.

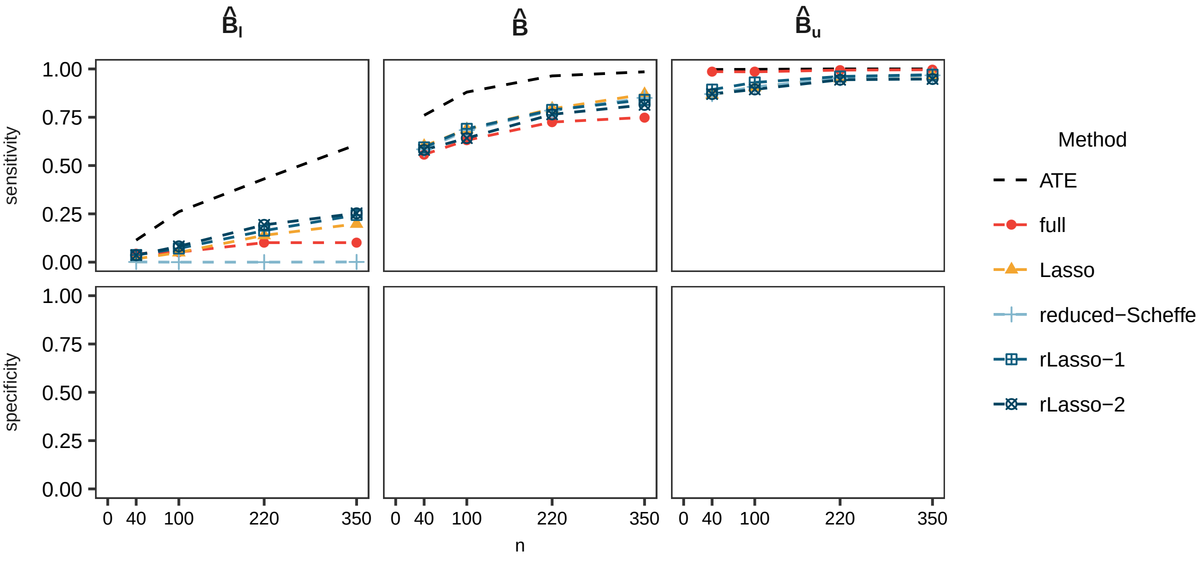

Figure 47: Sensitivity for identified subgroup by method and sample size for Case 5 and 10 biomarkers. Specificity is not shown as all patients have $D(X) > 0$

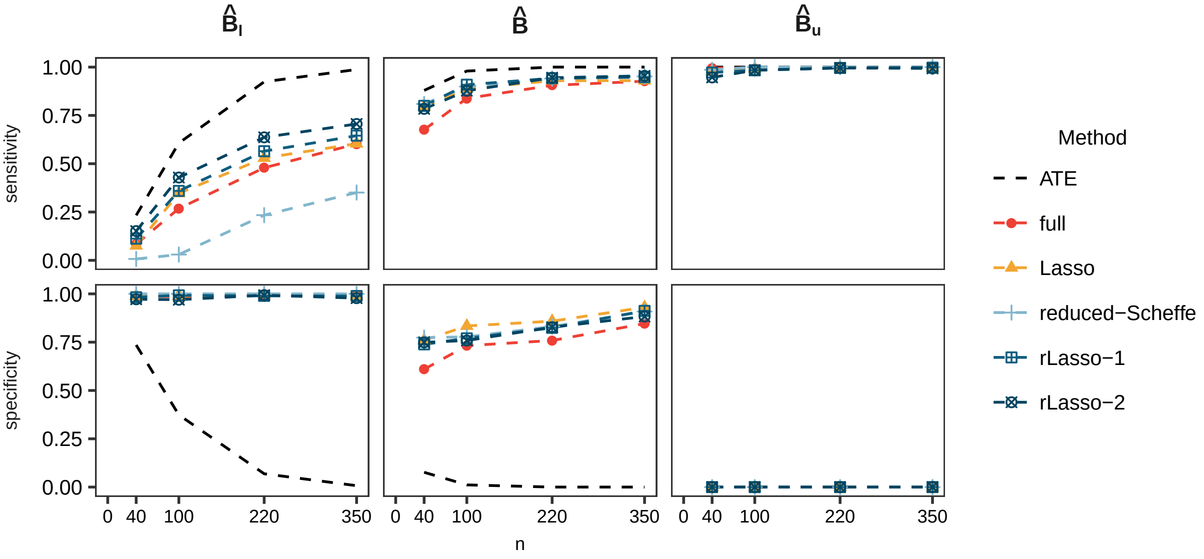

Figure 48: Sensitivity and specificity for identified subgroup by method and sample size for Case 6 and 10 biomarkers.

8 Results for block correlation structure

We now evaluate the case where the biomarkers are simulated using an blocked correlation structure. In this case, all biomarkers are normally distributed. We use 10 biomarkers and the same effect as in the independent case with also 10 biomarkers. There are two blocks of 5 biomarkers each. The correlation within each block is 0.5 for pairwise correlations.

8.1 Percent of selection

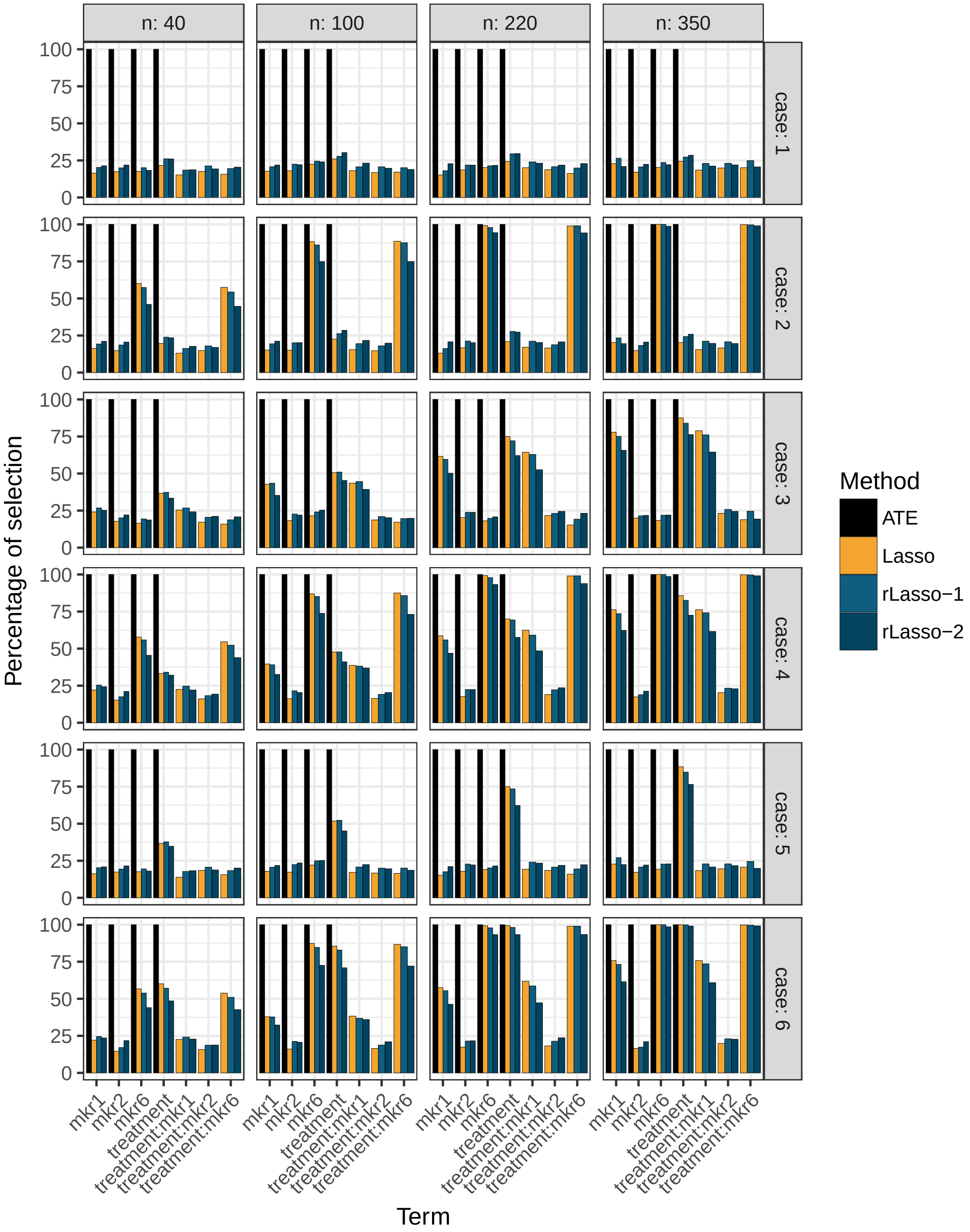

Figure 49: Selection probabilities for relevant terms in the model by method, case and sample size for 10 biomarkers. The full model is not represented here as all variables are included 100% of the times. The reduced model is not represented as it has the same values as the Lasso

8.2 Coverage of confidence intervals

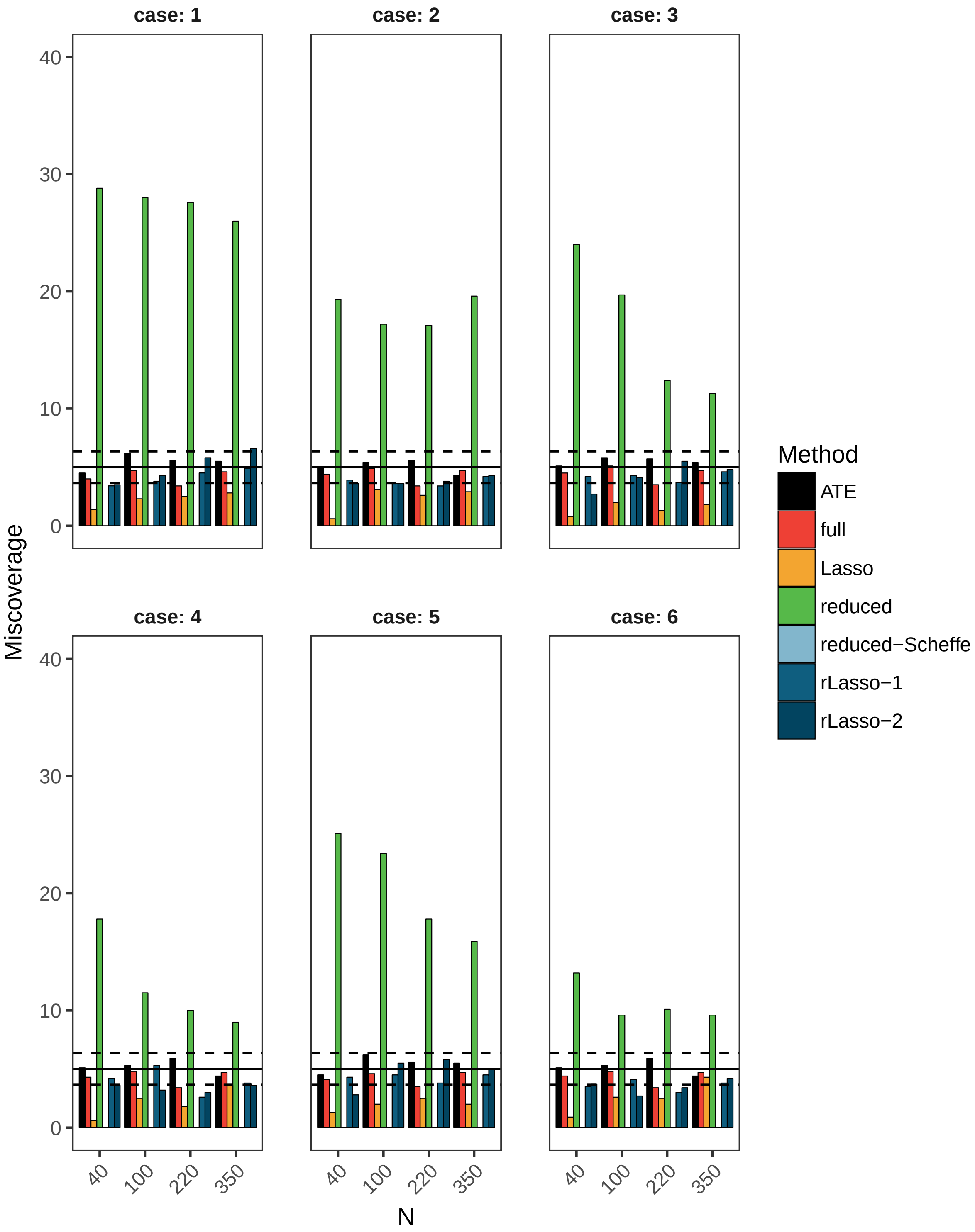

Figure 50: Average miscoverage of the confidence intervals for the PITE by method, case and sample size for 10 biomarkers. The line at 5% indicates the target miscoverage.

8.3 Bias, MSE and width

Table 39: Diagnostic measures for case 1 with 10 biomarkers. Columns 4 and 5 show the bias and the $\sqrt{MSE}$ for the point estimate of the PITE. The sixth column shows the median width of the confidence intervals for the PITE, and the last columns show the proportion of subjects in the identified subgroup when considering the using the limits of the confidence intervals and the point estimates. Since methods reduced and reduced-Scheffe have the same point estimate, bias and MSE are equal.

Case	N	Method	Bias	$\sqrt{MSE}$	Width	% in $\hat{B}_l$	% in $\hat{B}$	% in $\hat{B}_u$	% in B
1	40	ATE	0.01	0.37	1.46	2.30	51.3	97.80	0
1	40	full	0.06	1.61	6.16	2.10	50.8	98.10	0
1	40	Lasso	0.00	0.31	4.10	0.50	47.0	90.70	0
1	40	reduced	0.01	0.73	1.31	15.10	47.9	77.90	0
1	40	reduced-Scheffe	0.01	0.73	4.76	0.00	47.9	91.60	0
1	40	rLasso-1	0.01	0.74	2.91	1.60	47.4	93.29	0
1	40	rLasso-2	0.00	0.65	2.24	1.50	46.9	92.60	0
1	100	ATE	-0.01	0.22	0.83	3.20	50.0	97.00	0
1	100	full	0.03	0.74	2.94	3.10	52.3	98.40	0
1	100	Lasso	0.01	0.22	2.89	1.50	46.1	89.50	0
1	100	reduced	0.03	0.52	0.93	15.70	45.7	78.00	0
1	100	reduced-Scheffe	0.03	0.52	2.88	0.00	45.7	90.30	0
1	100	rLasso-1	0.02	0.51	1.92	1.80	48.1	92.90	0
1	100	rLasso-2	0.02	0.45	1.42	1.70	46.9	92.40	0
1	220	ATE	0.00	0.14	0.54	3.00	50.5	97.40	0
1	220	full	0.00	0.46	1.80	1.30	51.1	97.90	0
1	220	Lasso	0.00	0.15	1.77	1.10	45.4	88.80	0
1	220	reduced	0.00	0.34	0.63	14.50	46.2	77.10	0
1	220	reduced-Scheffe	0.00	0.34	1.90	0.00	46.2	90.20	0
1	220	rLasso-1	-0.01	0.34	1.26	2.30	47.5	93.39	0
1	220	rLasso-2	0.01	0.32	0.93	2.90	49.7	92.10	0
1	350	ATE	0.00	0.11	0.43	2.50	49.9	97.00	0
1	350	full	0.02	0.37	1.36	2.70	49.8	98.10	0
1	350	Lasso	0.00	0.12	1.47	1.40	43.6	90.30	0
1	350	reduced	0.00	0.26	0.50	12.80	43.3	78.50	0
1	350	reduced-Scheffe	0.00	0.26	1.49	0.00	43.3	91.70	0
1	350	rLasso-1	0.00	0.26	0.97	2.51	45.0	92.89	0
1	350	rLasso-2	0.01	0.23	0.74	3.20	48.5	90.90	0

Table 40: Diagnostic measures for case 2 with 10 biomarkers. Columns 4 and 5 show the bias and the $\sqrt{MSE}$ for the point estimate of the PITE. The sixth column shows the median width of the confidence intervals for the PITE, and the last columns show the proportion of subjects in the identified subgroup when considering the using the limits of the confidence intervals and the point estimates. Since methods reduced and reduced-Scheffe have the same point estimate, bias and MSE are equal.

Case	N	Method	Bias	$\sqrt{MSE}$	Width	% in $\hat{B}_l$	% in $\hat{B}$	% in $\hat{B}_u$	% in B
2	40	ATE	0.00	0.68	1.53	2.9	51.2	97.9	50.2
2	40	full	0.07	1.65	6.22	2.9	51.1	97.0	50.2
2	40	Lasso	0.00	0.49	5.13	1.2	50.2	92.8	50.2
2	40	reduced	0.04	0.77	1.49	20.7	50.1	77.2	50.2
2	40	reduced-Scheffe	0.04	0.77	5.20	0.0	50.1	94.5	50.2
2	40	rLasso-1	0.02	0.80	3.35	3.4	50.1	92.8	50.2
2	40	rLasso-2	-0.01	0.77	2.49	3.7	49.2	92.3	50.2
2	100	ATE	0.00	0.65	0.87	2.6	51.2	97.2	48.9
2	100	full	0.03	0.74	2.94	6.8	50.1	95.0	48.9
2	100	Lasso	0.01	0.38	2.84	11.1	48.7	90.7	48.9
2	100	reduced	0.01	0.53	1.12	25.6	49.7	72.9	48.9
2	100	reduced-Scheffe	0.01	0.53	3.46	0.0	49.7	99.2	48.9
2	100	rLasso-1	0.02	0.53	2.20	9.9	49.5	89.9	48.9
2	100	rLasso-2	0.02	0.51	1.70	9.4	49.0	89.2	48.9
2	220	ATE	0.03	0.61	0.57	3.3	51.5	97.7	47.2
2	220	full	0.00	0.46	1.80	10.9	47.3	89.9	47.2
2	220	Lasso	0.01	0.24	1.61	16.3	48.5	80.3	47.2
2	220	reduced	0.00	0.34	0.79	32.0	47.7	64.7	47.2
2	220	reduced-Scheffe	0.00	0.34	2.36	1.4	47.7	98.8	47.2
2	220	rLasso-1	0.00	0.34	1.46	16.9	48.6	82.8	47.2
2	220	rLasso-2	0.02	0.33	1.13	20.2	49.0	78.3	47.2
2	350	ATE	0.00	0.61	0.44	1.7	49.2	97.4	49.9
2	350	full	0.02	0.37	1.37	15.8	48.9	85.8	49.9
2	350	Lasso	0.00	0.20	1.31	21.1	47.9	76.9	49.9
2	350	reduced	0.00	0.26	0.61	33.8	48.2	64.5	49.9
2	350	reduced-Scheffe	0.00	0.26	1.80	5.3	48.2	94.9	49.9
2	350	rLasso-1	0.00	0.26	1.10	23.2	48.3	76.5	49.9
2	350	rLasso-2	0.01	0.25	0.87	27.1	49.3	72.6	49.9

Table 41: Diagnostic measures for case 3 with 10 biomarkers. Columns 4 and 5 show the bias and the $\sqrt{MSE}$ for the point estimate of the PITE. The sixth column shows the median width of the confidence intervals for the PITE, and the last columns show the proportion of subjects in the identified subgroup when considering the using the limits of the confidence intervals and the point estimates. Since methods reduced and reduced-Scheffe have the same point estimate, bias and MSE are equal.

Case	N	Method	Bias	$\sqrt{MSE}$	Width	% in $\hat{B}_l$	% in $\hat{B}$	% in $\hat{B}_u$	% in B
3	40	ATE	0.02	0.45	1.47	10.0	77.4	99.3	83.8
3	40	full	0.09	1.62	6.16	3.6	57.7	98.7	83.8
3	40	Lasso	-0.18	0.43	4.69	0.9	57.2	92.4	83.8
3	40	reduced	-0.06	0.79	1.43	23.6	58.2	82.6	83.8
3	40	reduced-Scheffe	-0.06	0.79	5.02	0.0	58.2	92.8	83.8
3	40	rLasso-1	-0.08	0.78	3.05	2.2	56.7	93.2	83.8
3	40	rLasso-2	-0.11	0.73	2.42	2.5	55.8	93.7	83.8
3	100	ATE	-0.01	0.33	0.84	22.1	85.5	100.0	83.2
3	100	full	0.01	0.75	2.94	6.3	63.1	98.6	83.2
3	100	Lasso	-0.15	0.34	3.13	4.3	65.3	95.6	83.2
3	100	reduced	-0.03	0.56	1.07	27.3	64.3	89.0	83.2
3	100	reduced-Scheffe	-0.03	0.56	3.32	0.0	64.3	96.6	83.2
3	100	rLasso-1	-0.05	0.55	2.11	6.0	62.9	96.1	83.2
3	100	rLasso-2	-0.07	0.52	1.54	7.2	62.7	94.6	83.2
3	220	ATE	0.00	0.29	0.55	43.3	96.0	100.0	84.4
3	220	full	0.00	0.46	1.80	10.8	68.3	99.2	84.4
3	220	Lasso	-0.13	0.27	1.99	7.4	71.5	97.2	84.4
3	220	reduced	-0.02	0.38	0.84	37.0	71.8	91.9	84.4
3	220	reduced-Scheffe	-0.02	0.38	2.50	0.0	71.8	98.5	84.4
3	220	rLasso-1	-0.04	0.38	1.41	10.5	70.4	96.8	84.4
3	220	rLasso-2	-0.06	0.37	1.12	16.3	70.0	96.5	84.4
3	350	ATE	0.00	0.27	0.43	61.4	98.9	100.0	83.3
3	350	full	0.02	0.37	1.37	15.6	73.3	98.3	83.3
3	350	Lasso	-0.11	0.23	1.51	13.3	75.3	98.7	83.3
3	350	reduced	-0.01	0.29	0.69	42.1	74.7	94.8	83.3
3	350	reduced-Scheffe	-0.01	0.29	2.05	0.4	74.7	99.8	83.3
3	350	rLasso-1	-0.02	0.29	1.11	17.6	73.4	97.5	83.3
3	350	rLasso-2	-0.03	0.28	0.87	22.7	74.2	96.2	83.3

Table 42: Diagnostic measures for case 4 with 10 biomarkers. Columns 4 and 5 show the bias and the $\sqrt{MSE}$ for the point estimate of the PITE. The sixth column shows the median width of the confidence intervals for the PITE, and the last columns show the proportion of subjects in the identified subgroup when considering the using the limits of the confidence intervals and the point estimates. Since methods reduced and reduced-Scheffe have the same point estimate, bias and MSE are equal.

Case	N	Method	Bias	$\sqrt{MSE}$	Width	% in $\hat{B}_l$	% in $\hat{B}$	% in $\hat{B}_u$	% in B
4	40	ATE	0.01	0.74	1.54	10.0	76.9	99.4	64.9
4	40	full	0.09	1.63	6.15	4.2	59.9	98.0	64.9
4	40	Lasso	-0.19	0.59	5.54	1.4	57.5	95.1	64.9
4	40	reduced	-0.07	0.84	1.58	25.5	57.7	82.4	64.9
4	40	reduced-Scheffe	-0.07	0.84	5.53	0.0	57.7	96.7	64.9
4	40	rLasso-1	-0.09	0.85	3.57	3.9	57.2	92.6	64.9
4	40	rLasso-2	-0.13	0.83	2.60	4.5	53.4	92.0	64.9
4	100	ATE	-0.01	0.69	0.88	20.7	85.0	100.0	65.4
4	100	full	0.03	0.74	2.94	11.6	60.8	97.5	65.4
4	100	Lasso	-0.16	0.46	2.89	10.4	57.4	94.3	65.4
4	100	reduced	-0.04	0.57	1.27	31.4	59.9	83.5	65.4
4	100	reduced-Scheffe	-0.04	0.57	3.92	0.5	59.9	99.9	65.4
4	100	rLasso-1	-0.05	0.58	2.38	11.5	59.2	93.3	65.4
4	100	rLasso-2	-0.10	0.58	1.84	12.0	58.2	91.6	65.4
4	220	ATE	0.04	0.67	0.57	41.8	96.0	100.0	64.7
4	220	full	0.00	0.46	1.80	19.9	60.6	93.4	64.7
4	220	Lasso	-0.13	0.33	1.94	16.3	57.7	92.5	64.7
4	220	reduced	-0.03	0.38	0.96	37.7	60.4	81.2	64.7
4	220	reduced-Scheffe	-0.03	0.38	2.86	1.6	60.4	99.8	64.7
4	220	rLasso-1	-0.03	0.38	1.63	19.2	60.7	90.2	64.7
4	220	rLasso-2	-0.06	0.39	1.30	23.4	59.1	86.6	64.7
4	350	ATE	0.00	0.67	0.45	58.1	98.4	100.0	64.4
4	350	full	0.02	0.37	1.37	25.9	63.2	91.3	64.4
4	350	Lasso	-0.12	0.29	1.45	22.8	58.8	88.9	64.4
4	350	reduced	-0.01	0.29	0.79	42.3	63.3	83.6	64.4
4	350	reduced-Scheffe	-0.01	0.29	2.33	6.8	63.3	99.3	64.4
4	350	rLasso-1	-0.03	0.30	1.24	25.3	60.9	87.6	64.4
4	350	rLasso-2	-0.04	0.31	1.06	31.6	60.6	85.2	64.4

Table 43: Diagnostic measures for case 5 with 10 biomarkers. Columns 4 and 5 show the bias and the $\sqrt{MSE}$ for the point estimate of the PITE. The sixth column shows the median width of the confidence intervals for the PITE, and the last columns show the proportion of subjects in the identified subgroup when considering the using the limits of the confidence intervals and the point estimates. Since methods reduced and reduced-Scheffe have the same point estimate, bias and MSE are equal.

Case	N	Method	Bias	$\sqrt{MSE}$	Width	% in $\hat{B}_l$	% in $\hat{B}$	% in $\hat{B}_u$	% in B
5	40	ATE	0.01	0.37	1.46	10.4	76.8	99.5	100
5	40	full	0.09	1.61	6.15	3.1	58.4	99.3	100
5	40	Lasso	-0.18	0.37	4.50	0.7	56.9	92.7	100
5	40	reduced	-0.07	0.75	1.37	23.1	58.3	84.6	100
5	40	reduced-Scheffe	-0.07	0.75	4.91	0.0	58.3	93.4	100
5	40	rLasso-1	-0.08	0.76	3.04	2.7	58.6	93.4	100
5	40	rLasso-2	-0.10	0.68	2.27	2.2	56.4	93.8	100
5	100	ATE	-0.01	0.22	0.83	21.7	85.7	100.0	100
5	100	full	0.03	0.74	2.94	5.7	64.2	99.2	100
5	100	Lasso	-0.14	0.28	2.79	3.0	65.9	93.4	100
5	100	reduced	-0.02	0.53	1.00	26.6	64.4	87.3	100
5	100	reduced-Scheffe	-0.02	0.53	3.08	0.0	64.4	93.9	100
5	100	rLasso-1	-0.04	0.54	1.97	3.9	65.5	95.7	100
5	100	rLasso-2	-0.08	0.48	1.46	5.0	61.1	93.7	100
5	220	ATE	0.00	0.14	0.54	43.9	96.1	100.0	100
5	220	full	0.00	0.46	1.80	8.4	70.0	99.8	100
5	220	Lasso	-0.13	0.22	1.90	8.1	77.4	97.1	100
5	220	reduced	-0.02	0.36	0.73	40.1	75.0	94.3	100
5	220	reduced-Scheffe	-0.02	0.36	2.19	0.0	75.0	97.6	100
5	220	rLasso-1	-0.04	0.36	1.28	11.3	73.8	97.3	100
5	220	rLasso-2	-0.05	0.34	1.01	14.7	73.3	97.1	100
5	350	ATE	0.00	0.11	0.43	62.5	98.9	100.0	100
5	350	full	0.02	0.37	1.37	11.4	77.8	99.6	100
5	350	Lasso	-0.11	0.18	1.46	12.2	84.6	98.4	100
5	350	reduced	-0.01	0.28	0.58	44.5	81.3	97.0	100
5	350	reduced-Scheffe	-0.01	0.28	1.72	0.0	81.3	98.7	100
5	350	rLasso-1	-0.01	0.28	1.02	16.1	79.7	98.4	100
5	350	rLasso-2	-0.03	0.26	0.79	20.5	80.0	97.1	100

Table 44: Diagnostic measures for case 6 with 10 biomarkers. Columns 4 and 5 show the bias and the $\sqrt{MSE}$ for the point estimate of the PITE. The sixth column shows the median width of the confidence intervals for the PITE, and the last columns show the proportion of subjects in the identified subgroup when considering the using the limits of the confidence intervals and the point estimates. Since methods reduced and reduced-Scheffe have the same point estimate, bias and MSE are equal.

Case	N	Method	Bias	$\sqrt{MSE}$	Width	% in $\hat{B}_l$	% in $\hat{B}$	% in $\hat{B}_u$	% in B
6	40	ATE	0.01	0.74	1.54	27.3	90.8	100.0	78.0
6	40	full	0.10	1.64	6.15	5.7	64.7	98.7	78.0
6	40	Lasso	-0.32	0.67	6.01	3.0	69.3	97.4	78.0
6	40	reduced	-0.10	0.86	1.67	32.9	69.3	90.2	78.0
6	40	reduced-Scheffe	-0.10	0.86	5.85	0.0	69.3	98.1	78.0
6	40	rLasso-1	-0.13	0.88	3.65	5.3	67.8	95.9	78.0
6	40	rLasso-2	-0.20	0.88	2.73	7.5	64.7	94.9	78.0
6	100	ATE	-0.01	0.69	0.88	60.8	98.4	100.0	78.6
6	100	full	0.03	0.74	2.94	18.4	70.3	98.7	78.6
6	100	Lasso	-0.23	0.52	2.85	17.7	71.4	97.3	78.6
6	100	reduced	-0.03	0.59	1.36	41.7	69.3	93.3	78.6
6	100	reduced-Scheffe	-0.03	0.59	4.18	0.7	69.3	100.0	78.6
6	100	rLasso-1	-0.03	0.60	2.40	19.4	70.2	96.9	78.6
6	100	rLasso-2	-0.09	0.61	1.92	22.2	69.8	95.6	78.6
6	220	ATE	0.04	0.67	0.57	92.3	100.0	100.0	76.3
6	220	full	0.00	0.46	1.80	31.7	72.4	96.1	76.3
6	220	Lasso	-0.15	0.35	1.76	31.2	75.2	97.2	76.3
6	220	reduced	0.00	0.37	0.99	50.6	74.6	92.6	76.3
6	220	reduced-Scheffe	0.00	0.37	2.95	8.7	74.6	100.0	76.3
6	220	rLasso-1	0.00	0.37	1.60	33.5	74.7	96.6	76.3
6	220	rLasso-2	-0.01	0.38	1.35	38.4	75.6	96.7	76.3
6	350	ATE	0.00	0.67	0.45	99.4	100.0	100.0	78.1
6	350	full	0.02	0.37	1.37	39.1	76.3	96.5	78.1
6	350	Lasso	-0.13	0.30	1.23	39.8	77.7	95.7	78.1
6	350	reduced	0.00	0.28	0.80	55.9	75.0	92.5	78.1
6	350	reduced-Scheffe	0.00	0.28	2.35	17.5	75.0	100.0	78.1
6	350	rLasso-1	-0.01	0.28	1.23	42.8	75.3	96.0	78.1
6	350	rLasso-2	0.00	0.29	1.07	47.1	76.1	95.8	78.1

8.4 Sensitivity and Specificity

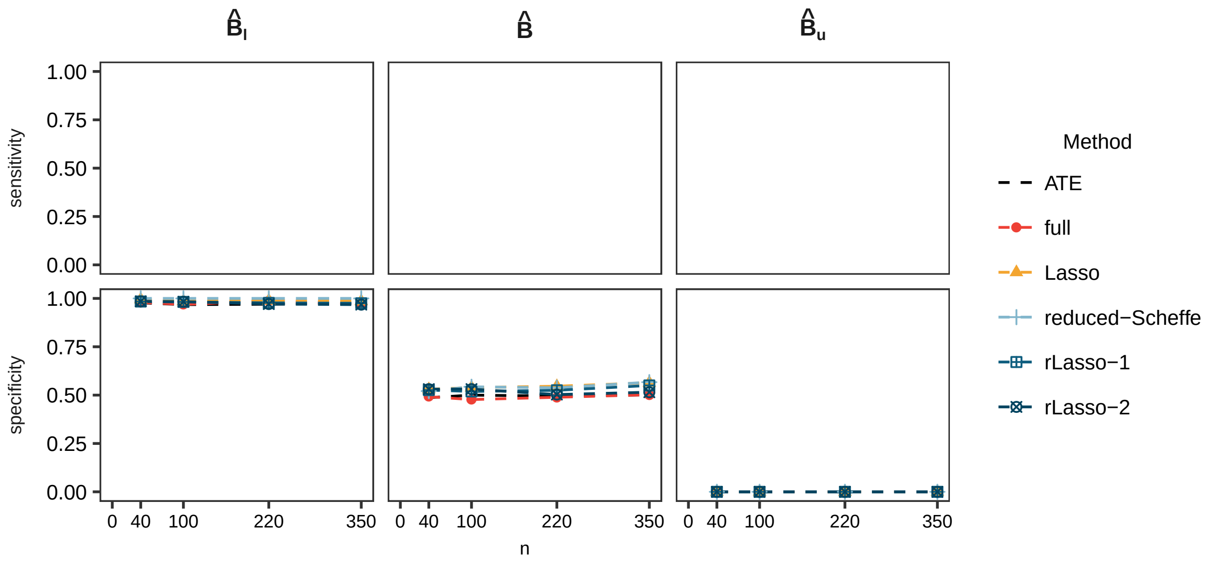

Figure 51: Specificity for identified subgroup by method and sample size for Case 1 and 10 biomarkers. Sensitivity is not shown as there are no patients with $D(X) > 0$

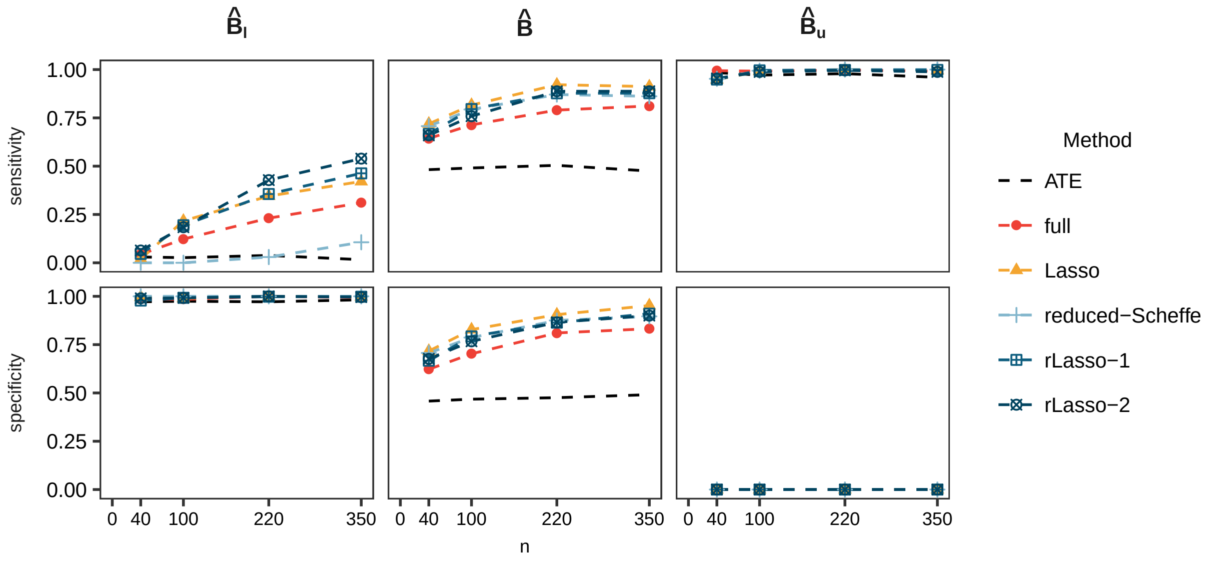

Figure 52: Sensitivity and specificity for identified subgroup by method and sample size for Case 2 and 10 biomarkers.

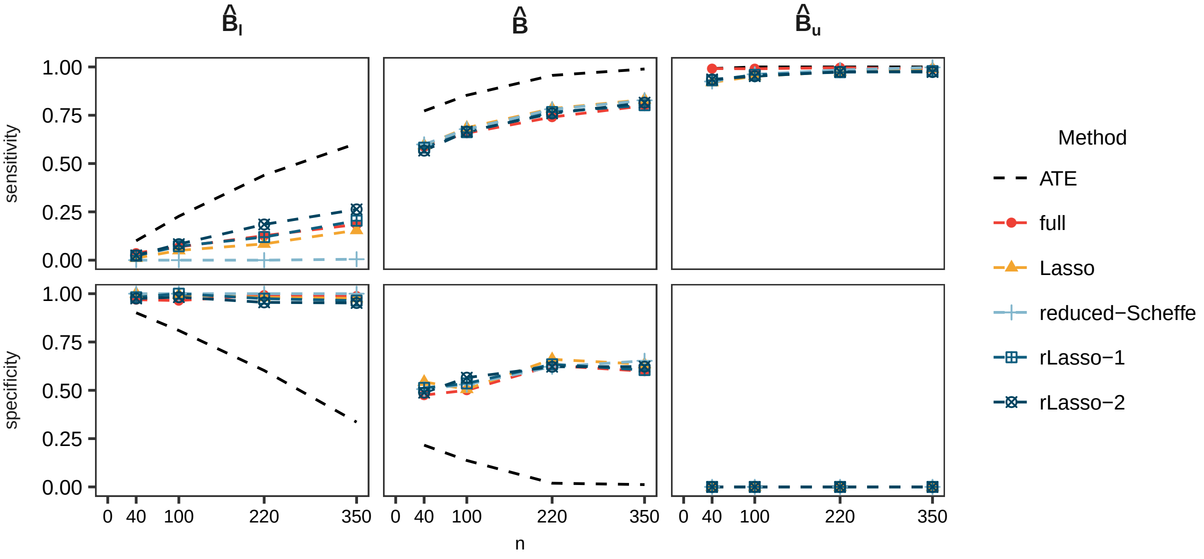

Figure 53: Sensitivity and specificity for identified subgroup by method and sample size for Case 3 and 10 biomarkers.

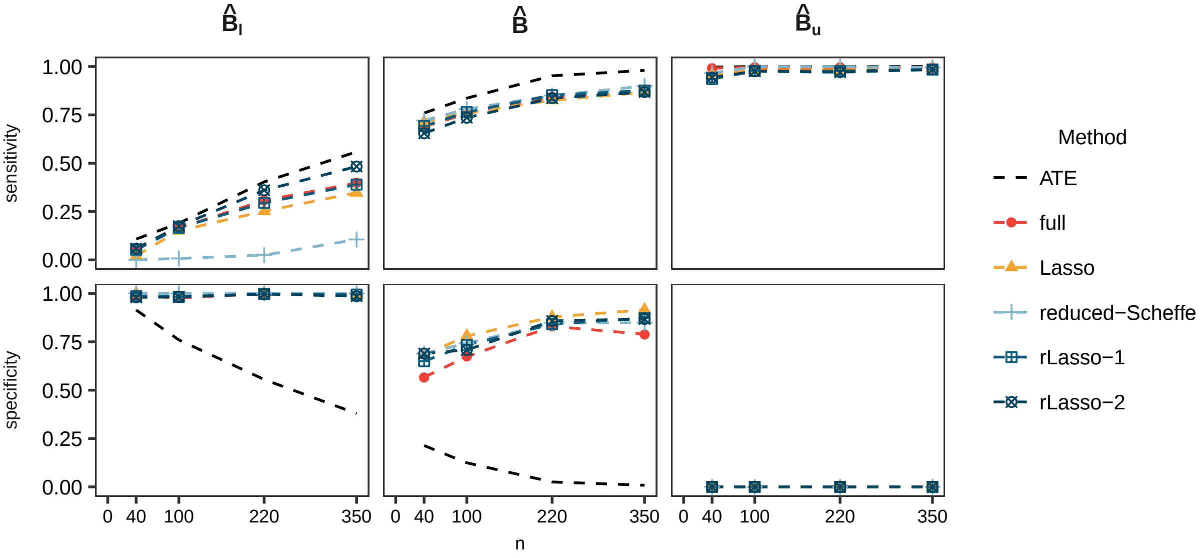

Figure 54: Sensitivity and specificity for identified subgroup by method and sample size for Case 4 and 10 biomarkers.

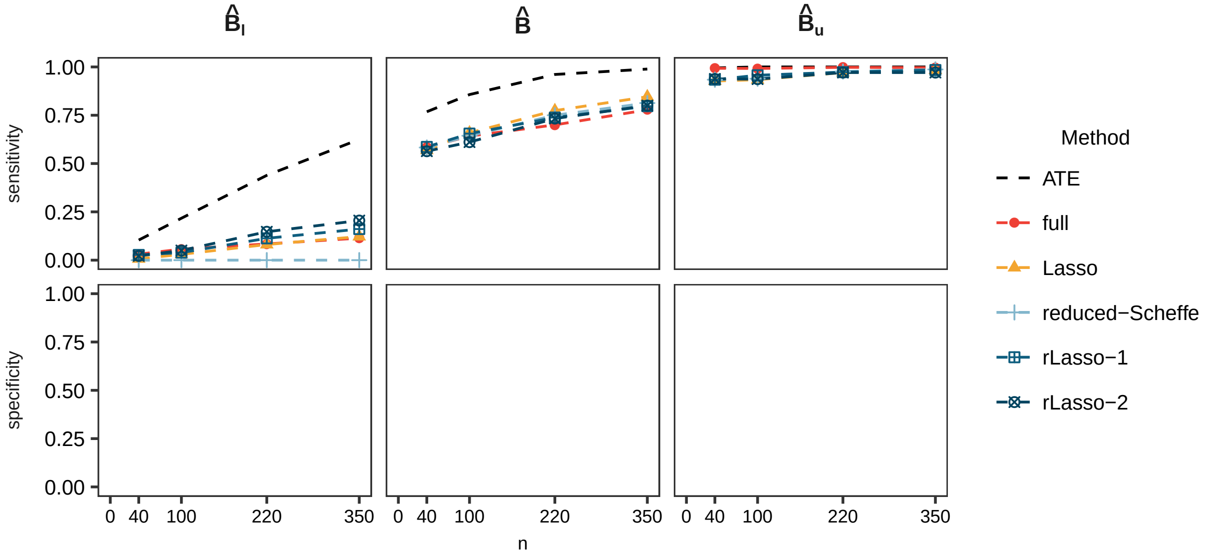

Figure 55: Sensitivity for identified subgroup by method and sample size for Case 5 and 10 biomarkers. Specificity is not shown as all patients have $D(X) > 0$

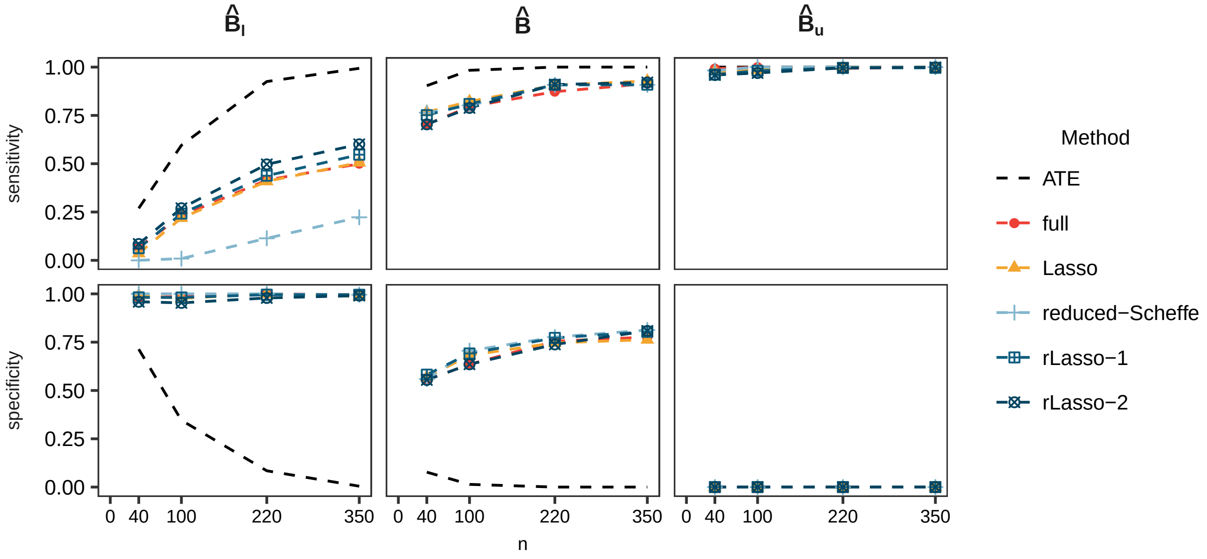

Figure 56: Sensitivity and specificity for identified subgroup by method and sample size for Case 6 and 10 biomarkers.

9 Results for 10 Biomarkers. Default tol.beta parameter in selectiveInference

9.1 Percent of selection

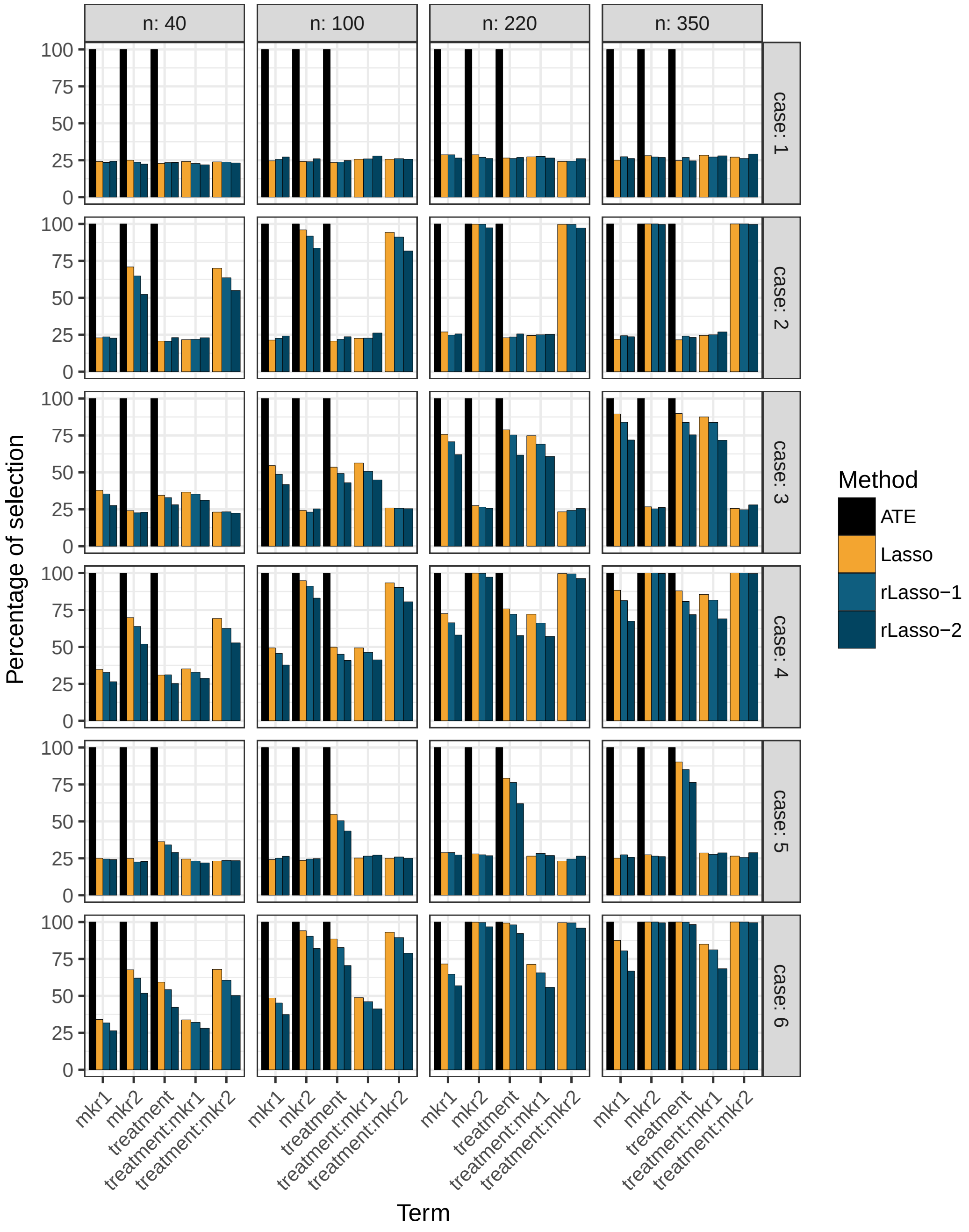

Figure 57: Selection probabilities for relevant terms in the model by method, case and sample size for 10 biomarkers. The full model is not represented here as all variables are included 100% of the times. The reduced model is not represented as it has the same values as the Lasso

9.2 Coverage of confidence intervals

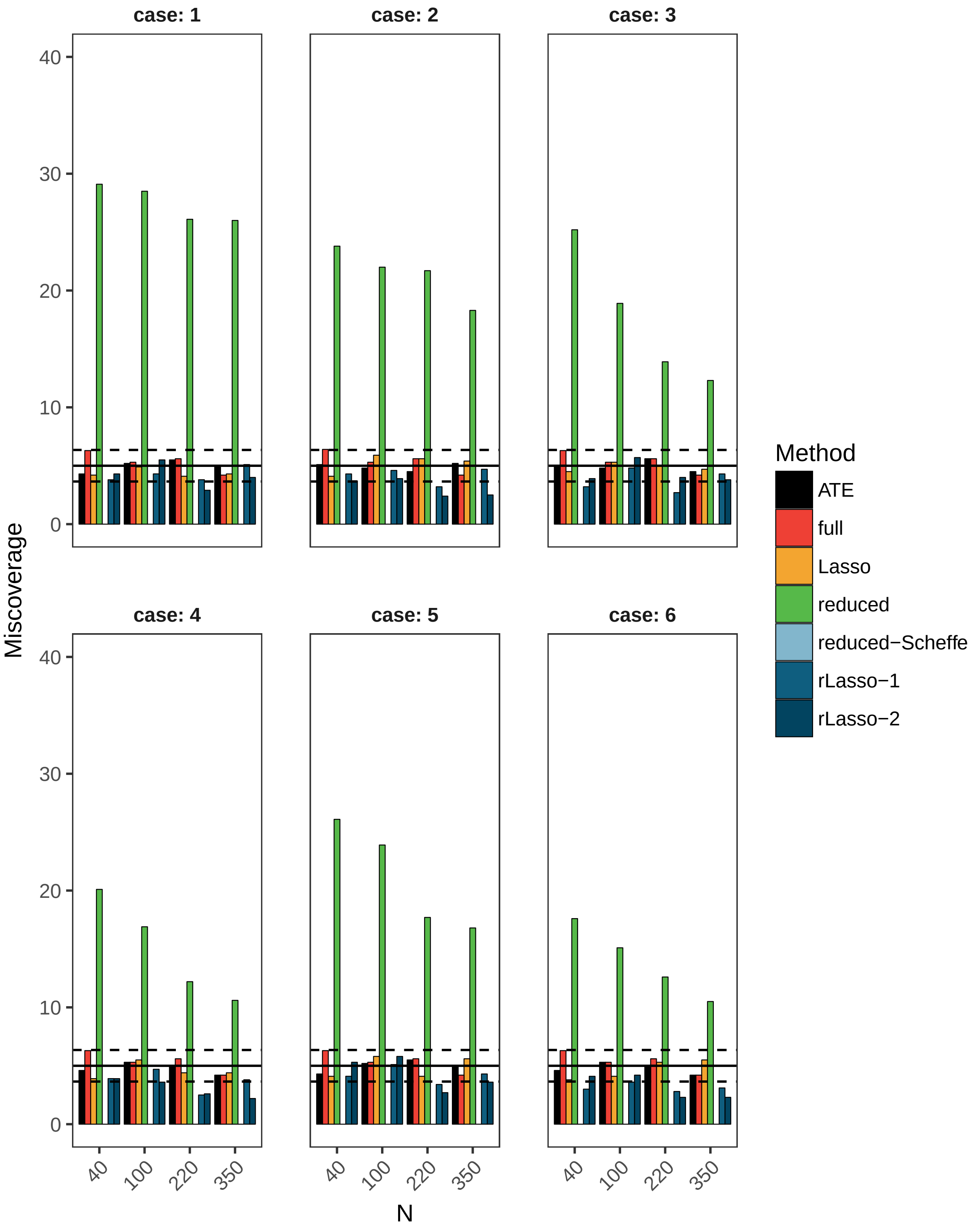

Figure 58: Average miscoverage of the confidence intervals for the PITE by method, case and sample size for 10 biomarkers. The line at 5% indicates the target miscoverage.

9.3 Bias, MSE and width

Table 45: Diagnostic measures for case 1 with 10 biomarkers. Columns 4 and 5 show the bias and the $\sqrt{MSE}$ for the point estimate of the PITE. The sixth column shows the median width of the confidence intervals for the PITE, and the last columns show the proportion of subjects in the identified subgroup when considering the using the limits of the confidence intervals and the point estimates. Since methods reduced and reduced-Scheffe have the same point estimate, bias and MSE are equal.

Case	N	Method	Bias	$\sqrt{MSE}$	Width	% in $\hat{B}_l$	% in $\hat{B}$	% in $\hat{B}_u$	% in B
1	40	ATE	0.01	0.36	1.48	2.5	50.6	98.20	0
1	40	full	-0.06	1.60	6.19	2.2	50.1	95.90	0
1	40	Lasso	0.01	0.33	10.33	2.6	51.5	94.80	0
1	40	reduced	0.01	0.86	1.66	14.2	50.8	81.50	0
1	40	reduced-Scheffe	0.01	0.86	6.16	0.0	50.8	96.40	0
1	40	rLasso-1	0.00	0.82	3.37	2.0	49.8	94.40	0
1	40	rLasso-2	-0.01	0.75	2.47	2.0	47.9	93.90	0
1	100	ATE	-0.01	0.21	0.83	2.0	49.6	96.80	0
1	100	full	0.00	0.78	2.88	2.1	50.8	96.80	0
1	100	Lasso	0.00	0.24	5.78	2.2	47.1	92.80	0
1	100	reduced	0.00	0.58	1.10	14.1	47.6	81.10	0
1	100	reduced-Scheffe	0.00	0.58	3.50	0.0	47.6	95.50	0
1	100	rLasso-1	-0.01	0.56	2.14	1.7	47.4	94.49	0
1	100	rLasso-2	-0.01	0.47	1.57	2.0	48.8	94.30	0
1	220	ATE	0.01	0.14	0.54	3.2	52.7	97.70	0
1	220	full	0.00	0.48	1.79	2.7	49.9	97.10	0
1	220	Lasso	0.00	0.15	3.80	2.0	49.8	94.70	0
1	220	reduced	0.00	0.39	0.78	12.5	48.9	83.20	0
1	220	reduced-Scheffe	0.00	0.39	2.35	0.0	48.9	96.80	0
1	220	rLasso-1	0.00	0.37	1.46	2.1	49.4	95.50	0
1	220	rLasso-2	0.00	0.32	1.10	1.5	45.3	94.40	0
1	350	ATE	0.00	0.10	0.43	2.8	49.4	97.90	0
1	350	full	0.02	0.36	1.39	2.5	54.5	98.30	0
1	350	Lasso	0.01	0.12	2.91	1.9	51.8	94.90	0
1	350	reduced	0.02	0.30	0.62	13.8	51.9	85.10	0
1	350	reduced-Scheffe	0.02	0.30	1.85	0.0	51.9	97.30	0
1	350	rLasso-1	0.02	0.28	1.11	2.6	50.3	94.20	0
1	350	rLasso-2	0.01	0.26	0.83	2.1	48.1	94.60	0

Table 46: Diagnostic measures for case 2 with 10 biomarkers. Columns 4 and 5 show the bias and the $\sqrt{MSE}$ for the point estimate of the PITE. The sixth column shows the median width of the confidence intervals for the PITE, and the last columns show the proportion of subjects in the identified subgroup when considering the using the limits of the confidence intervals and the point estimates. Since methods reduced and reduced-Scheffe have the same point estimate, bias and MSE are equal.

Case	N	Method	Bias	$\sqrt{MSE}$	Width	% in $\hat{B}_l$	% in $\hat{B}$	% in $\hat{B}_u$	% in B
2	40	ATE	0.00	0.69	1.55	2.7	50.3	97.60	50.2
2	40	full	-0.06	1.62	6.21	2.4	50.1	95.20	50.2
2	40	Lasso	0.00	0.51	9.92	3.8	50.5	95.10	50.2
2	40	reduced	0.02	0.86	1.75	20.7	50.2	79.20	50.2
2	40	reduced-Scheffe	0.02	0.86	6.41	0.0	50.2	97.80	50.2
2	40	rLasso-1	-0.01	0.83	3.69	4.1	47.9	94.10	50.2
2	40	rLasso-2	-0.01	0.85	2.79	3.9	49.3	93.19	50.2
2	100	ATE	-0.01	0.61	0.87	1.8	49.3	97.00	50.2
2	100	full	-0.01	0.78	2.88	7.0	50.7	92.80	50.2
2	100	Lasso	-0.02	0.38	4.76	7.6	49.4	89.40	50.2
2	100	reduced	-0.01	0.60	1.25	24.6	50.2	73.80	50.2
2	100	reduced-Scheffe	-0.01	0.60	3.93	0.0	50.2	99.50	50.2
2	100	rLasso-1	-0.02	0.58	2.39	8.0	49.6	90.10	50.2
2	100	rLasso-2	-0.02	0.52	1.79	9.2	49.3	87.70	50.2
2	220	ATE	0.00	0.60	0.56	2.5	52.0	98.00	50.7
2	220	full	0.00	0.48	1.79	10.9	52.4	89.40	50.7
2	220	Lasso	0.00	0.25	3.37	14.7	51.3	88.20	50.7
2	220	reduced	0.00	0.40	0.91	30.6	52.2	71.00	50.7
2	220	reduced-Scheffe	0.00	0.40	2.72	1.2	52.2	99.00	50.7
2	220	rLasso-1	0.01	0.37	1.55	17.6	52.2	82.60	50.7
2	220	rLasso-2	0.00	0.34	1.22	21.2	51.0	79.40	50.7
2	350	ATE	-0.01	0.57	0.44	2.4	49.6	97.20	51.1
2	350	full	0.02	0.36	1.39	17.7	51.6	85.10	51.1
2	350	Lasso	0.00	0.19	2.35	17.0	50.6	81.80	51.1
2	350	reduced	0.02	0.29	0.68	34.9	51.5	66.60	51.1
2	350	reduced-Scheffe	0.02	0.29	2.03	4.0	51.5	96.50	51.1
2	350	rLasso-1	0.01	0.28	1.18	24.0	52.3	77.80	51.1
2	350	rLasso-2	0.01	0.26	0.93	27.4	50.2	74.80	51.1

Table 47: Diagnostic measures for case 3 with 10 biomarkers. Columns 4 and 5 show the bias and the $\sqrt{MSE}$ for the point estimate of the PITE. The sixth column shows the median width of the confidence intervals for the PITE, and the last columns show the proportion of subjects in the identified subgroup when considering the using the limits of the confidence intervals and the point estimates. Since methods reduced and reduced-Scheffe have the same point estimate, bias and MSE are equal.

Case	N	Method	Bias	$\sqrt{MSE}$	Width	% in $\hat{B}_l$	% in $\hat{B}$	% in $\hat{B}_u$	% in B
3	40	ATE	-0.01	0.45	1.49	8.7	76.8	99.7	53.0
3	40	full	-0.07	1.61	6.23	3.1	56.8	97.3	53.0
3	40	Lasso	-0.19	0.45	10.41	2.6	60.0	95.1	53.0
3	40	reduced	-0.08	0.89	1.75	21.6	59.5	87.2	53.0
3	40	reduced-Scheffe	-0.08	0.89	6.51	0.0	59.5	97.5	53.0
3	40	rLasso-1	-0.11	0.87	3.53	2.8	56.1	95.3	53.0
3	40	rLasso-2	-0.14	0.81	2.65	3.0	55.8	94.6	53.0
3	100	ATE	0.00	0.32	0.84	19.5	88.4	99.8	49.3
3	100	full	-0.01	0.78	2.88	6.1	61.6	97.8	49.3
3	100	Lasso	-0.17	0.36	5.95	4.4	61.7	96.5	49.3
3	100	reduced	-0.05	0.62	1.26	24.7	62.2	89.2	49.3
3	100	reduced-Scheffe	-0.05	0.62	3.96	0.0	62.2	98.8	49.3
3	100	rLasso-1	-0.07	0.60	2.32	4.4	61.6	95.8	49.3
3	100	rLasso-2	-0.10	0.55	1.73	5.3	60.3	96.1	49.3
3	220	ATE	0.00	0.28	0.55	45.4	96.1	100.0	51.3
3	220	full	0.00	0.48	1.78	10.8	69.1	98.3	51.3
3	220	Lasso	-0.13	0.28	3.83	7.6	71.3	96.1	51.3
3	220	reduced	-0.02	0.42	0.95	32.6	70.0	94.5	51.3
3	220	reduced-Scheffe	-0.02	0.42	2.87	0.0	70.0	99.3	51.3
3	220	rLasso-1	-0.03	0.41	1.58	8.9	69.3	98.0	51.3
3	220	rLasso-2	-0.07	0.41	1.18	14.5	67.1	95.7	51.3
3	350	ATE	-0.01	0.27	0.43	64.0	99.3	100.0	51.5
3	350	full	0.02	0.36	1.39	18.6	72.4	99.3	51.5
3	350	Lasso	-0.11	0.23	2.80	13.4	74.8	97.9	51.5
3	350	reduced	0.01	0.31	0.77	40.5	74.0	95.9	51.5
3	350	reduced-Scheffe	0.01	0.31	2.27	0.5	74.0	100.0	51.5
3	350	rLasso-1	0.01	0.31	1.17	19.4	72.6	97.3	51.5
3	350	rLasso-2	-0.03	0.31	0.95	22.9	73.8	97.5	51.5

Table 48: Diagnostic measures for case 4 with 10 biomarkers. Columns 4 and 5 show the bias and the $\sqrt{MSE}$ for the point estimate of the PITE. The sixth column shows the median width of the confidence intervals for the PITE, and the last columns show the proportion of subjects in the identified subgroup when considering the using the limits of the confidence intervals and the point estimates. Since methods reduced and reduced-Scheffe have the same point estimate, bias and MSE are equal.

Case	N	Method	Bias	$\sqrt{MSE}$	Width	% in $\hat{B}_l$	% in $\hat{B}$	% in $\hat{B}_u$	% in B
4	40	ATE	-0.02	0.75	1.56	8.50	75.2	99.70	64.1
4	40	full	-0.08	1.62	6.21	4.10	56.0	96.30	64.1
4	40	Lasso	-0.21	0.61	11.67	3.10	56.7	96.60	64.1
4	40	reduced	-0.11	0.93	1.85	24.60	57.2	85.00	64.1
4	40	reduced-Scheffe	-0.11	0.93	6.88	0.00	57.2	99.00	64.1
4	40	rLasso-1	-0.14	0.91	3.81	4.00	55.0	94.79	64.1
4	40	rLasso-2	-0.18	0.92	2.85	5.10	53.9	93.60	64.1
4	100	ATE	-0.01	0.68	0.87	19.50	88.9	99.80	61.4
4	100	full	-0.01	0.78	2.88	11.80	59.6	94.90	61.4
4	100	Lasso	-0.18	0.48	5.31	8.90	56.3	94.20	61.4
4	100	reduced	-0.08	0.65	1.40	30.10	58.6	82.50	61.4
4	100	reduced-Scheffe	-0.08	0.65	4.37	0.10	58.6	99.80	61.4
4	100	rLasso-1	-0.09	0.62	2.51	10.60	58.1	92.30	61.4
4	100	rLasso-2	-0.12	0.59	1.96	10.80	57.7	90.60	61.4
4	220	ATE	0.00	0.65	0.57	42.30	96.4	100.00	63.1
4	220	full	0.00	0.48	1.79	21.10	63.8	93.40	63.1
4	220	Lasso	-0.14	0.36	3.16	15.90	59.5	92.60	63.1
4	220	reduced	-0.01	0.42	1.03	38.60	63.9	82.60	63.1
4	220	reduced-Scheffe	-0.01	0.42	3.10	0.90	63.9	99.70	63.1
4	220	rLasso-1	-0.03	0.42	1.69	20.60	60.9	90.00	63.1
4	220	rLasso-2	-0.08	0.43	1.34	25.10	60.3	86.10	63.1
4	350	ATE	-0.02	0.63	0.45	60.20	98.7	100.00	64.8
4	350	full	0.02	0.36	1.39	29.90	64.7	90.60	64.8
4	350	Lasso	-0.12	0.29	2.20	21.10	60.0	91.20	64.8
4	350	reduced	0.01	0.32	0.83	43.50	64.2	82.10	64.8
4	350	reduced-Scheffe	0.01	0.32	2.46	7.40	64.2	99.00	64.8
4	350	rLasso-1	0.00	0.32	1.26	28.03	63.6	88.79	64.8
4	350	rLasso-2	-0.03	0.33	1.05	31.90	63.1	85.40	64.8

Table 49: Diagnostic measures for case 5 with 10 biomarkers. Columns 4 and 5 show the bias and the $\sqrt{MSE}$ for the point estimate of the PITE. The sixth column shows the median width of the confidence intervals for the PITE, and the last columns show the proportion of subjects in the identified subgroup when considering the using the limits of the confidence intervals and the point estimates. Since methods reduced and reduced-Scheffe have the same point estimate, bias and MSE are equal.

Case	N	Method	Bias	$\sqrt{MSE}$	Width	% in $\hat{B}_l$	% in $\hat{B}$	% in $\hat{B}_u$	% in B
5	40	ATE	0.01	0.36	1.48	9.1	76.7	99.70	100
5	40	full	-0.05	1.61	6.20	2.8	56.9	97.30	100
5	40	Lasso	-0.17	0.39	10.70	3.4	58.9	95.40	100
5	40	reduced	-0.07	0.88	1.72	20.0	58.3	86.50	100
5	40	reduced-Scheffe	-0.07	0.88	6.35	0.0	58.3	96.90	100
5	40	rLasso-1	-0.10	0.84	3.41	2.8	56.9	94.89	100
5	40	rLasso-2	-0.13	0.79	2.52	3.0	54.8	93.80	100
5	100	ATE	-0.01	0.21	0.83	19.5	88.2	99.90	100
5	100	full	0.00	0.78	2.89	4.9	64.9	98.50	100
5	100	Lasso	-0.17	0.31	6.05	4.3	65.6	96.60	100
5	100	reduced	-0.06	0.61	1.19	23.0	65.7	90.40	100
5	100	reduced-Scheffe	-0.06	0.61	3.75	0.0	65.7	98.70	100
5	100	rLasso-1	-0.07	0.58	2.28	4.1	64.3	96.60	100
5	100	rLasso-2	-0.11	0.51	1.63	5.1	61.2	95.60	100
5	220	ATE	0.01	0.14	0.54	46.8	96.8	100.00	100
5	220	full	0.00	0.48	1.79	8.8	70.9	99.20	100
5	220	Lasso	-0.12	0.22	3.78	6.6	78.2	98.00	100
5	220	reduced	-0.02	0.41	0.88	31.9	74.6	94.70	100
5	220	reduced-Scheffe	-0.02	0.41	2.65	0.0	74.6	99.00	100
5	220	rLasso-1	-0.02	0.39	1.55	9.2	73.0	99.00	100
5	220	rLasso-2	-0.06	0.36	1.11	9.8	70.5	97.40	100
5	350	ATE	0.00	0.10	0.43	65.5	99.2	100.00	100
5	350	full	0.02	0.36	1.39	10.6	77.8	100.00	100
5	350	Lasso	-0.11	0.18	2.97	9.9	85.3	97.90	100
5	350	reduced	0.01	0.31	0.70	41.8	81.1	98.30	100
5	350	reduced-Scheffe	0.01	0.31	2.09	0.0	81.1	99.70	100
5	350	rLasso-1	0.00	0.30	1.14	13.6	81.2	98.90	100
5	350	rLasso-2	-0.03	0.29	0.92	17.1	80.2	98.50	100

Table 50: Diagnostic measures for case 6 with 10 biomarkers. Columns 4 and 5 show the bias and the $\sqrt{MSE}$ for the point estimate of the PITE. The sixth column shows the median width of the confidence intervals for the PITE, and the last columns show the proportion of subjects in the identified subgroup when considering the using the limits of the confidence intervals and the point estimates. Since methods reduced and reduced-Scheffe have the same point estimate, bias and MSE are equal.

Case	N	Method	Bias	$\sqrt{MSE}$	Width	% in $\hat{B}_l$	% in $\hat{B}$	% in $\hat{B}_u$	% in B
6	40	ATE	-0.02	0.75	1.56	22.90	91.4	100.0	77.0
6	40	full	-0.07	1.62	6.23	5.80	62.8	97.3	77.0
6	40	Lasso	-0.35	0.69	12.57	4.20	65.9	98.0	77.0
6	40	reduced	-0.13	0.95	2.00	31.40	65.1	91.0	77.0
6	40	reduced-Scheffe	-0.13	0.95	7.23	0.00	65.1	99.7	77.0
6	40	rLasso-1	-0.20	0.95	4.12	5.30	64.1	96.4	77.0
6	40	rLasso-2	-0.26	0.98	2.99	6.80	61.5	95.1	77.0
6	100	ATE	-0.01	0.68	0.87	60.30	98.5	100.0	73.7
6	100	full	0.00	0.78	2.88	17.10	68.0	97.0	73.7
6	100	Lasso	-0.26	0.54	5.27	14.40	70.5	96.9	73.7
6	100	reduced	-0.05	0.66	1.46	40.90	70.0	90.9	73.7
6	100	reduced-Scheffe	-0.05	0.66	4.56	0.30	70.0	100.0	73.7
6	100	rLasso-1	-0.08	0.65	2.59	16.50	69.2	95.9	73.7
6	100	rLasso-2	-0.12	0.63	2.07	17.60	69.2	95.0	73.7
6	220	ATE	0.00	0.65	0.57	93.40	100.0	100.0	74.8
6	220	full	0.00	0.48	1.79	31.20	73.3	96.3	74.8
6	220	Lasso	-0.16	0.38	2.87	26.10	74.2	98.1	74.8
6	220	reduced	0.01	0.41	1.06	51.80	74.0	93.5	74.8
6	220	reduced-Scheffe	0.01	0.41	3.17	5.40	74.0	100.0	74.8
6	220	rLasso-1	-0.01	0.41	1.68	35.00	74.0	97.4	74.8
6	220	rLasso-2	-0.03	0.43	1.38	38.80	75.5	95.6	74.8
6	350	ATE	-0.02	0.63	0.45	99.20	100.0	100.0	76.6
6	350	full	0.02	0.36	1.39	42.00	76.2	96.7	76.6
6	350	Lasso	-0.13	0.30	2.01	35.20	77.3	96.2	76.6
6	350	reduced	0.02	0.31	0.83	56.70	77.3	91.5	76.6
6	350	reduced-Scheffe	0.02	0.31	2.48	16.60	77.3	100.0	76.6
6	350	rLasso-1	0.02	0.30	1.24	43.94	77.2	96.5	76.6
6	350	rLasso-2	0.01	0.31	1.07	48.70	77.1	96.1	76.6

9.4 Sensitivity and Specificity

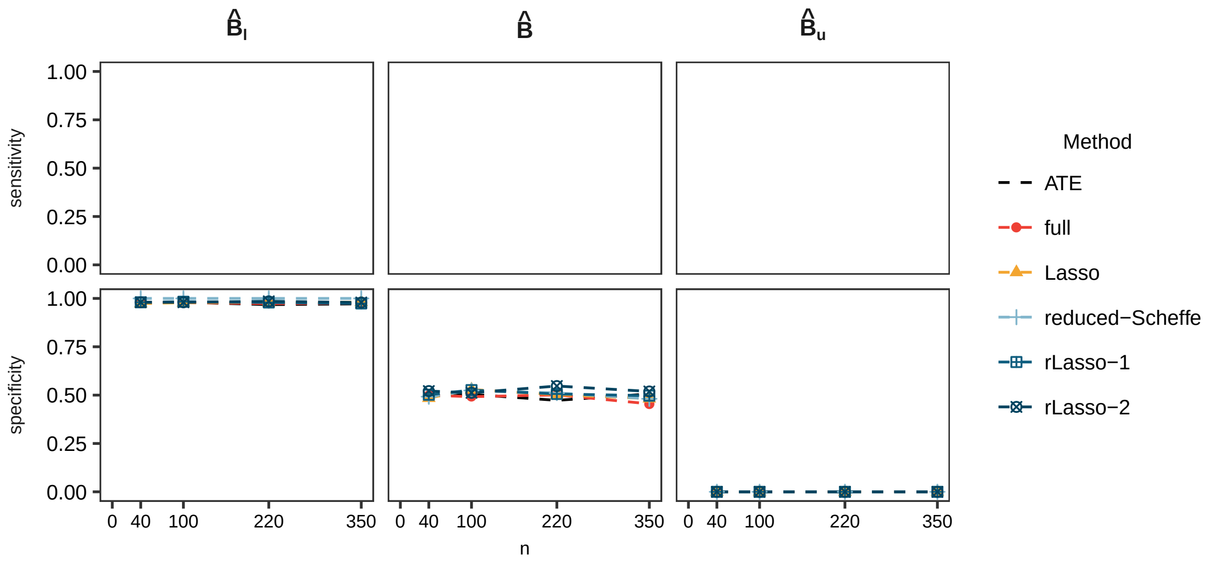

Figure 59: Specificity for identified subgroup by method and sample size for Case 1 and 10 biomarkers. Sensitivity is not shown as there are no patients with $D(X) > 0$

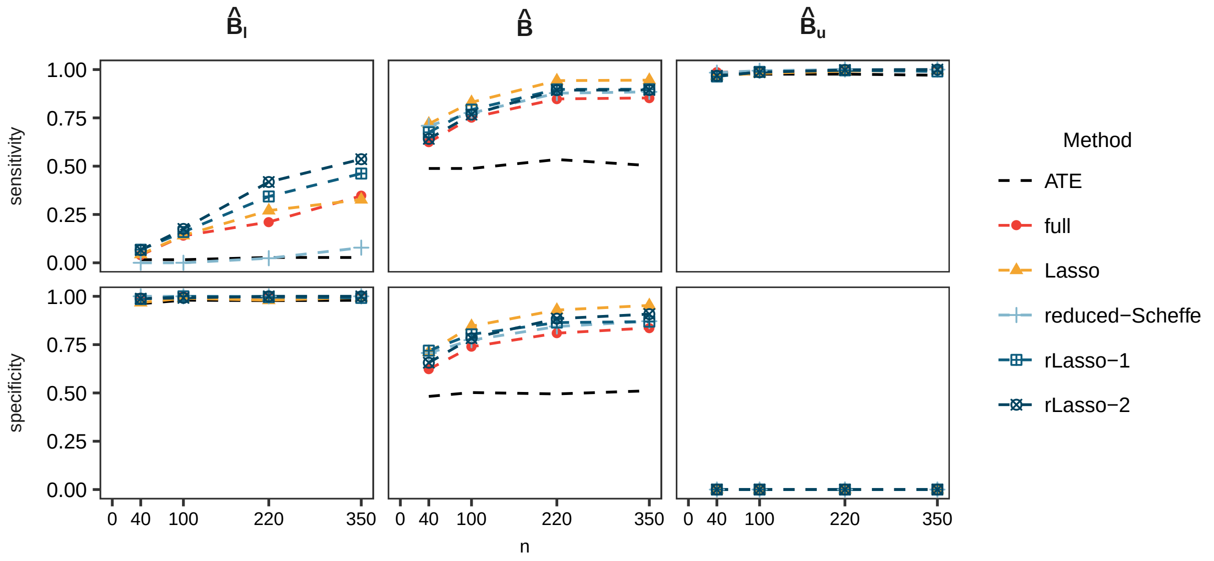

Figure 60: Sensitivity and specificity for identified subgroup by method and sample size for Case 2 and 10 biomarkers.

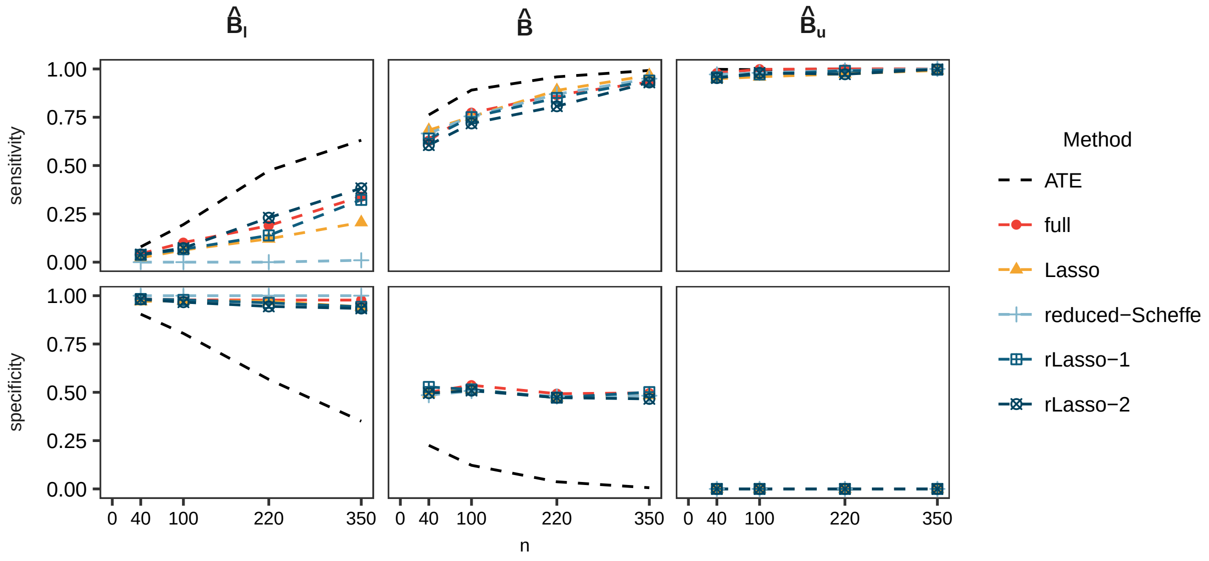

Figure 61: Sensitivity and specificity for identified subgroup by method and sample size for Case 3 and 10 biomarkers.

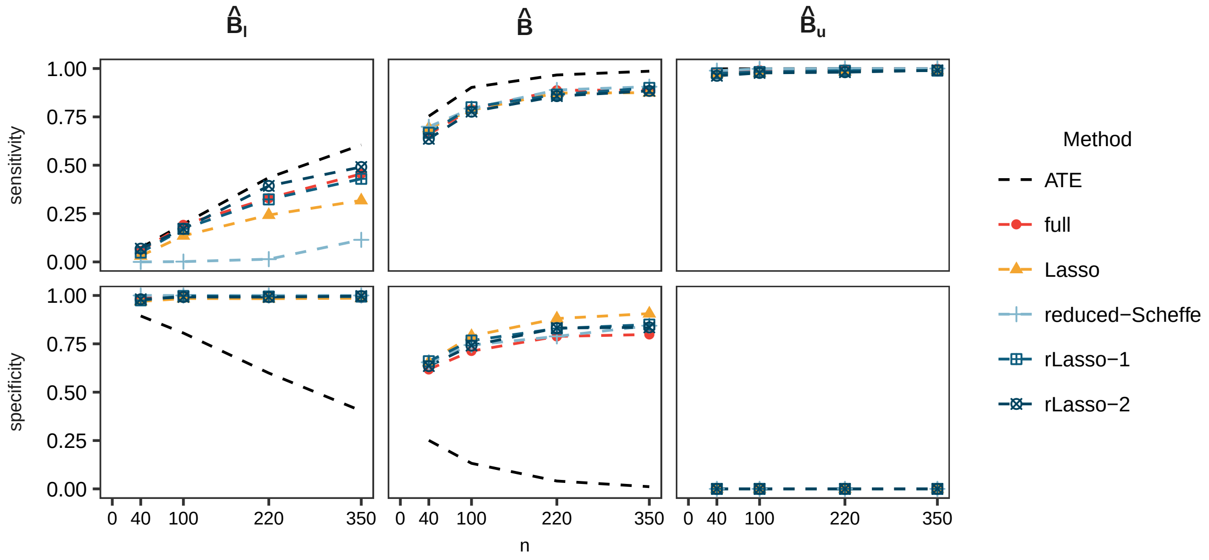

Figure 62: Sensitivity and specificity for identified subgroup by method and sample size for Case 4 and 10 biomarkers.

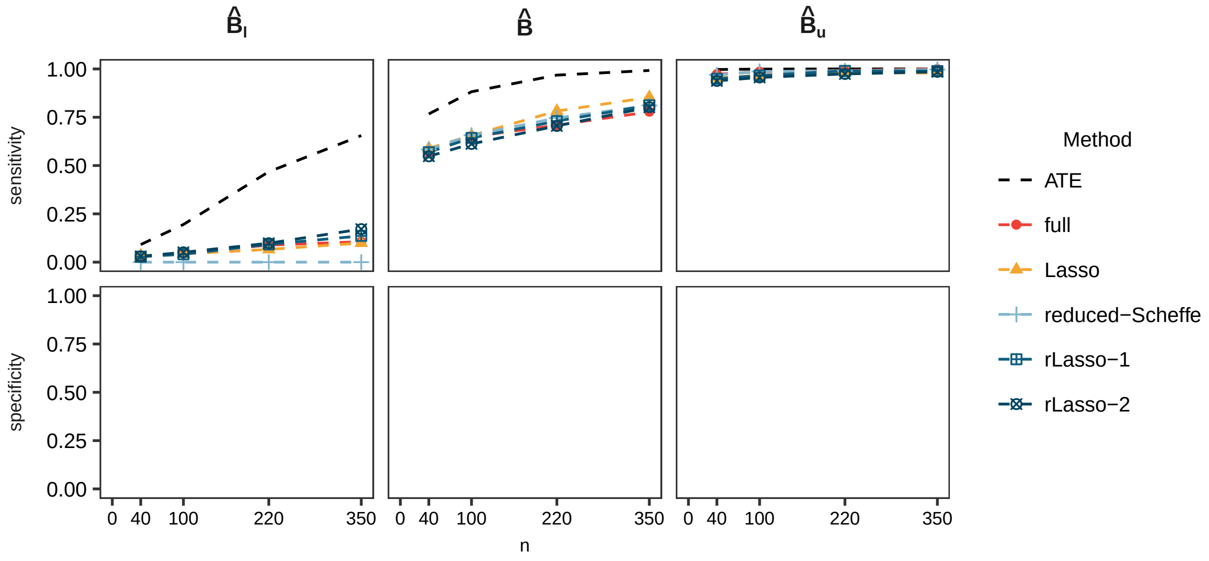

Figure 63: Sensitivity for identified subgroup by method and sample size for Case 5 and 10 biomarkers. Specificity is not shown as all patients have $D(X) > 0$

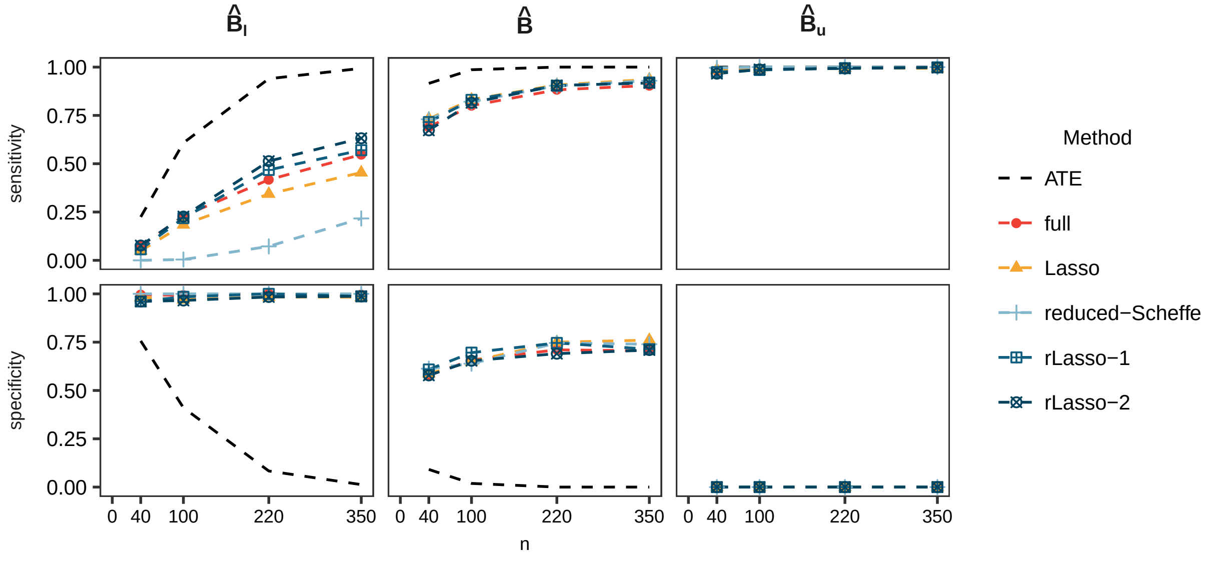

Figure 64: Sensitivity and specificity for identified subgroup by method and sample size for Case 6 and 10 biomarkers.

10 Results for 20 Biomarkers. Default tol.beta parameter in selectiveInference

10.1 Percent of selection

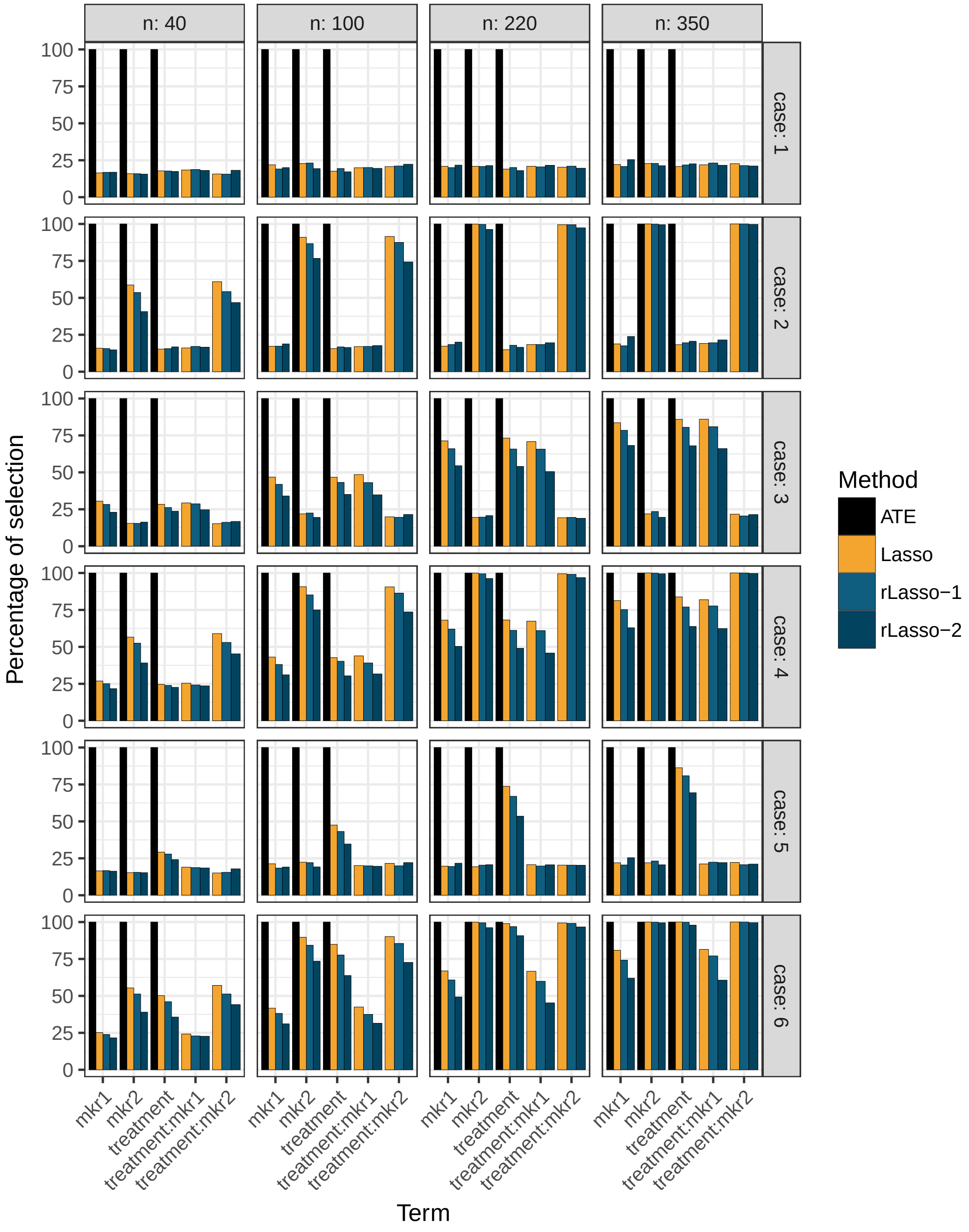

Figure 65: Selection probabilities for relevant terms in the model by method, case and sample size for 20 biomarkers. The full model is not represented here as all variables are included 100% of the times. The reduced model is not represented as it has the same values as the Lasso

10.2 Coverage of confidence intervals

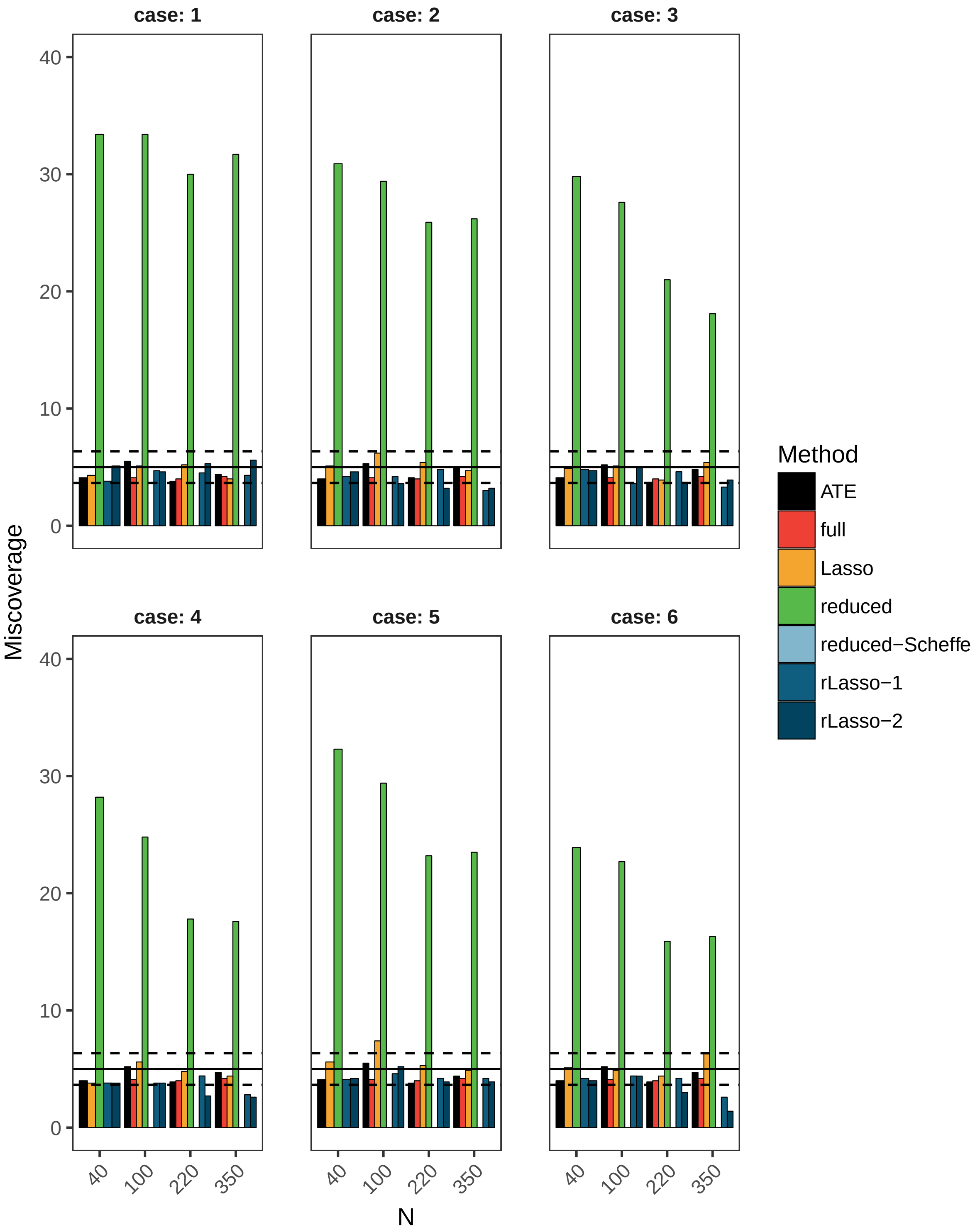

Figure 66: Average miscoverage of the confidence intervals for the PITE by method, case and sample size for 20 biomarkers. The line at 5% indicates the target miscoverage.

10.3 Bias, MSE and width

Table 51: Diagnostic measures for case 1 with 20 biomarkers. Columns 4 and 5 show the bias and the $\sqrt{MSE}$ for the point estimate of the PITE. The sixth column shows the median width of the confidence intervals for the PITE, and the last columns show the proportion of subjects in the identified subgroup when considering the using the limits of the confidence intervals and the point estimates. Since methods reduced and reduced-Scheffe have the same point estimate, bias and MSE are equal.

Case	N	Method	Bias	$\sqrt{MSE}$	Width	% in $\hat{B}_l$	% in $\hat{B}$	% in $\hat{B}_u$	% in B
1	40	ATE	-0.01	0.47	1.92	1.9	50.7	97.80	0
1	40	Lasso	0.00	0.38	17.38	2.0	52.1	96.60	0
1	40	reduced	-0.01	0.97	1.87	16.1	50.6	81.60	0
1	40	rLasso-1	0.00	0.97	4.39	1.8	50.8	97.10	0
1	40	rLasso-2	0.01	0.88	3.30	2.8	49.2	96.50	0
1	100	ATE	-0.01	0.22	0.89	2.5	47.9	97.00	0
1	100	full	0.04	1.21	4.72	2.1	50.7	98.00	0
1	100	Lasso	0.01	0.28	9.45	1.5	50.9	95.20	0
1	100	reduced	0.03	0.76	1.41	17.4	52.2	82.80	0
1	100	reduced-Scheffe	0.03	0.76	6.20	0.0	52.2	98.80	0
1	100	rLasso-1	0.01	0.71	2.89	2.2	50.3	96.40	0
1	100	rLasso-2	0.02	0.62	2.06	2.1	51.4	97.00	0
1	220	ATE	0.00	0.14	0.56	2.1	50.1	98.30	0
1	220	full	0.04	0.65	2.65	2.1	53.8	98.10	0
1	220	Lasso	0.00	0.17	6.85	2.6	50.2	96.30	0
1	220	reduced	0.01	0.48	0.98	15.5	49.9	84.40	0
1	220	reduced-Scheffe	0.01	0.48	4.03	0.0	49.9	98.90	0
1	220	rLasso-1	0.02	0.44	1.88	2.3	52.4	96.89	0
1	220	rLasso-2	0.01	0.39	1.41	2.4	50.6	96.30	0
1	350	ATE	0.00	0.11	0.43	1.9	49.1	97.50	0
1	350	full	0.01	0.50	2.02	1.8	51.9	97.60	0
1	350	Lasso	0.00	0.14	4.95	2.2	50.7	97.20	0
1	350	reduced	0.01	0.39	0.80	16.9	48.8	84.20	0
1	350	reduced-Scheffe	0.01	0.39	3.20	0.0	48.8	99.00	0
1	350	rLasso-1	0.02	0.37	1.51	1.9	52.1	96.80	0
1	350	rLasso-2	0.00	0.33	1.10	3.7	50.0	97.80	0

Table 52: Diagnostic measures for case 2 with 20 biomarkers. Columns 4 and 5 show the bias and the $\sqrt{MSE}$ for the point estimate of the PITE. The sixth column shows the median width of the confidence intervals for the PITE, and the last columns show the proportion of subjects in the identified subgroup when considering the using the limits of the confidence intervals and the point estimates. Since methods reduced and reduced-Scheffe have the same point estimate, bias and MSE are equal.

Case	N	Method	Bias	$\sqrt{MSE}$	Width	% in $\hat{B}_l$	% in $\hat{B}$	% in $\hat{B}_u$	% in B
2	40	ATE	-0.04	0.74	2.00	2.30	48.6	98.30	51.2
2	40	Lasso	-0.01	0.56	18.53	3.60	52.7	96.90	51.2
2	40	reduced	-0.01	1.03	2.00	20.50	51.6	79.40	51.2
2	40	rLasso-1	-0.02	1.05	4.94	2.90	49.8	96.00	51.2
2	40	rLasso-2	-0.02	0.99	3.52	3.40	49.8	95.60	51.2
2	100	ATE	-0.01	0.62	0.92	2.40	47.6	97.10	47.9
2	100	full	0.04	1.21	4.72	2.90	50.7	96.20	47.9
2	100	Lasso	0.01	0.43	8.51	6.20	50.6	94.50	47.9
2	100	reduced	0.03	0.74	1.49	22.20	50.9	79.70	47.9
2	100	reduced-Scheffe	0.03	0.74	6.55	0.00	50.9	99.90	47.9
2	100	rLasso-1	0.01	0.74	3.07	5.10	50.8	93.90	47.9
2	100	rLasso-2	0.01	0.68	2.28	8.20	49.4	92.00	47.9
2	220	ATE	0.00	0.59	0.58	2.30	50.8	98.20	50.2
2	220	full	0.04	0.65	2.65	6.30	52.1	93.90	50.2
2	220	Lasso	0.01	0.27	5.20	8.20	49.5	90.60	50.2
2	220	reduced	0.02	0.46	1.02	28.70	52.6	72.90	50.2
2	220	reduced-Scheffe	0.02	0.46	4.15	0.00	52.6	100.00	50.2
2	220	rLasso-1	0.02	0.43	1.95	14.31	51.3	87.19	50.2
2	220	rLasso-2	0.01	0.40	1.54	17.50	51.4	84.90	50.2
2	350	ATE	0.03	0.57	0.45	2.40	50.2	97.50	48.1
2	350	full	0.01	0.50	2.02	10.50	48.0	91.60	48.1
2	350	Lasso	0.01	0.22	4.27	9.80	47.0	89.60	48.1
2	350	reduced	0.01	0.38	0.82	29.00	48.3	68.30	48.1
2	350	reduced-Scheffe	0.01	0.38	3.28	0.00	48.3	99.80	48.1
2	350	rLasso-1	0.02	0.36	1.57	15.32	48.5	82.38	48.1
2	350	rLasso-2	0.00	0.33	1.21	19.60	46.6	80.10	48.1

Table 53: Diagnostic measures for case 3 with 20 biomarkers. Columns 4 and 5 show the bias and the $\sqrt{MSE}$ for the point estimate of the PITE. The sixth column shows the median width of the confidence intervals for the PITE, and the last columns show the proportion of subjects in the identified subgroup when considering the using the limits of the confidence intervals and the point estimates. Since methods reduced and reduced-Scheffe have the same point estimate, bias and MSE are equal.

Case	N	Method	Bias	$\sqrt{MSE}$	Width	% in $\hat{B}_l$	% in $\hat{B}$	% in $\hat{B}_u$	% in B
3	40	ATE	0.01	0.53	1.93	7.40	68.2	99.1	46.9
3	40	Lasso	-0.18	0.48	17.30	3.00	56.1	97.4	46.9
3	40	reduced	-0.10	1.00	1.90	21.40	55.6	86.0	46.9
3	40	rLasso-1	-0.11	0.98	4.61	3.40	55.6	96.4	46.9
3	40	rLasso-2	-0.15	0.93	3.36	3.30	54.2	97.2	46.9
3	100	ATE	-0.01	0.34	0.90	18.00	87.1	100.0	49.3
3	100	full	0.04	1.21	4.72	3.60	59.0	98.9	49.3
3	100	Lasso	-0.17	0.40	10.30	2.80	59.8	97.6	49.3
3	100	reduced	-0.04	0.80	1.51	24.60	60.6	88.8	49.3
3	100	reduced-Scheffe	-0.04	0.80	6.71	0.00	60.6	99.7	49.3
3	100	rLasso-1	-0.07	0.76	3.02	3.00	57.9	97.4	49.3
3	100	rLasso-2	-0.13	0.69	2.18	5.60	59.1	96.8	49.3
3	220	ATE	0.02	0.29	0.56	42.70	96.9	100.0	47.5
3	220	full	0.04	0.65	2.65	6.40	68.1	99.3	47.5
3	220	Lasso	-0.14	0.30	6.21	4.40	67.9	97.9	47.5
3	220	reduced	-0.01	0.49	1.12	28.60	66.5	92.7	47.5
3	220	reduced-Scheffe	-0.01	0.49	4.56	0.00	66.5	99.9	47.5
3	220	rLasso-1	-0.02	0.48	2.05	7.82	65.6	97.9	47.5
3	220	rLasso-2	-0.07	0.45	1.50	8.30	65.1	97.1	47.5
3	350	ATE	0.01	0.28	0.44	62.30	99.0	100.0	48.6
3	350	full	0.01	0.50	2.02	9.90	67.1	99.1	48.6
3	350	Lasso	-0.12	0.25	5.06	7.60	73.6	97.7	48.6
3	350	reduced	0.00	0.40	0.92	33.40	69.8	94.5	48.6
3	350	reduced-Scheffe	0.00	0.40	3.68	0.00	69.8	100.0	48.6
3	350	rLasso-1	-0.01	0.38	1.59	10.90	70.5	98.4	48.6
3	350	rLasso-2	-0.05	0.38	1.20	13.80	68.5	97.7	48.6

Table 54: Diagnostic measures for case 4 with 20 biomarkers. Columns 4 and 5 show the bias and the $\sqrt{MSE}$ for the point estimate of the PITE. The sixth column shows the median width of the confidence intervals for the PITE, and the last columns show the proportion of subjects in the identified subgroup when considering the using the limits of the confidence intervals and the point estimates. Since methods reduced and reduced-Scheffe have the same point estimate, bias and MSE are equal.

Case	N	Method	Bias	$\sqrt{MSE}$	Width	% in $\hat{B}_l$	% in $\hat{B}$	% in $\hat{B}_u$	% in B
4	40	ATE	-0.02	0.78	2.00	7.20	67.6	99.60	64.3
4	40	Lasso	-0.21	0.64	19.26	2.10	55.2	97.60	64.3
4	40	reduced	-0.10	1.07	2.09	23.00	55.8	83.50	64.3
4	40	rLasso-1	-0.11	1.07	5.10	3.20	55.9	96.50	64.3
4	40	rLasso-2	-0.18	1.06	3.67	3.80	53.5	97.20	64.3
4	100	ATE	0.00	0.66	0.93	16.30	86.3	100.00	62.4
4	100	full	0.04	1.21	4.72	4.60	57.9	97.50	62.4
4	100	Lasso	-0.18	0.52	8.64	4.30	56.5	95.90	62.4
4	100	reduced	-0.05	0.80	1.57	24.80	61.3	84.30	62.4
4	100	reduced-Scheffe	-0.05	0.80	6.91	0.00	61.3	100.00	62.4
4	100	rLasso-1	-0.10	0.79	3.22	6.30	57.3	95.00	62.4
4	100	rLasso-2	-0.15	0.76	2.42	8.40	55.6	92.80	62.4
4	220	ATE	0.01	0.64	0.59	38.20	96.8	100.00	62.2
4	220	full	0.04	0.65	2.65	12.10	61.3	96.30	62.2
4	220	Lasso	-0.15	0.37	5.35	8.40	58.0	94.50	62.2
4	220	reduced	-0.02	0.49	1.14	33.40	63.2	83.10	62.2
4	220	reduced-Scheffe	-0.02	0.49	4.64	0.00	63.2	100.00	62.2
4	220	rLasso-1	-0.04	0.48	2.11	15.53	61.0	91.28	62.2
4	220	rLasso-2	-0.08	0.47	1.64	18.40	59.6	88.10	62.2
4	350	ATE	0.03	0.63	0.45	57.90	99.1	100.00	59.3
4	350	full	0.01	0.50	2.02	16.90	59.1	96.00	59.3
4	350	Lasso	-0.12	0.31	3.95	11.80	55.6	92.40	59.3
4	350	reduced	-0.01	0.40	0.93	36.40	59.9	80.70	59.3
4	350	reduced-Scheffe	-0.01	0.40	3.72	0.00	59.9	100.00	59.3
4	350	rLasso-1	-0.02	0.39	1.66	20.42	57.9	89.69	59.3
4	350	rLasso-2	-0.06	0.39	1.31	24.40	57.1	86.70	59.3

Table 55: Diagnostic measures for case 5 with 20 biomarkers. Columns 4 and 5 show the bias and the $\sqrt{MSE}$ for the point estimate of the PITE. The sixth column shows the median width of the confidence intervals for the PITE, and the last columns show the proportion of subjects in the identified subgroup when considering the using the limits of the confidence intervals and the point estimates. Since methods reduced and reduced-Scheffe have the same point estimate, bias and MSE are equal.

Case	N	Method	Bias	$\sqrt{MSE}$	Width	% in $\hat{B}_l$	% in $\hat{B}$	% in $\hat{B}_u$	% in B
5	40	ATE	-0.01	0.47	1.92	7.40	68.4	99.20	100
5	40	Lasso	-0.20	0.43	16.85	3.00	58.1	96.70	100
5	40	reduced	-0.11	0.98	1.89	21.30	58.7	85.50	100
5	40	rLasso-1	-0.13	0.97	4.42	3.21	56.0	97.49	100
5	40	rLasso-2	-0.16	0.89	3.24	3.00	54.0	97.20	100
5	100	ATE	-0.01	0.22	0.89	18.20	86.8	100.00	100
5	100	full	0.03	1.21	4.72	3.90	60.1	98.90	100
5	100	Lasso	-0.18	0.35	10.23	4.50	62.4	96.50	100
5	100	reduced	-0.05	0.77	1.46	23.40	63.0	89.60	100
5	100	reduced-Scheffe	-0.05	0.77	6.50	0.00	63.0	99.50	100
5	100	rLasso-1	-0.08	0.74	2.93	3.10	59.6	96.50	100
5	100	rLasso-2	-0.12	0.65	2.09	5.00	59.7	97.60	100
5	220	ATE	0.00	0.14	0.56	43.10	96.9	100.00	100
5	220	full	0.04	0.65	2.65	6.00	67.6	99.50	100
5	220	Lasso	-0.15	0.24	6.79	3.80	71.5	97.80	100
5	220	reduced	-0.02	0.48	1.06	28.40	69.0	95.10	100
5	220	reduced-Scheffe	-0.02	0.48	4.31	0.00	69.0	99.50	100
5	220	rLasso-1	-0.03	0.45	1.96	6.01	69.1	98.60	100
5	220	rLasso-2	-0.07	0.42	1.45	6.50	66.2	98.30	100
5	350	ATE	0.00	0.11	0.43	61.60	98.9	100.00	100
5	350	full	0.01	0.50	2.02	6.40	69.1	99.60	100
5	350	Lasso	-0.12	0.20	4.89	6.70	80.7	98.40	100
5	350	reduced	-0.01	0.39	0.86	36.10	75.1	97.10	100
5	350	reduced-Scheffe	-0.01	0.39	3.44	0.00	75.1	99.90	100
5	350	rLasso-1	-0.01	0.37	1.52	9.30	76.4	99.10	100
5	350	rLasso-2	-0.05	0.36	1.15	12.50	73.5	99.10	100

Table 56: Diagnostic measures for case 6 with 20 biomarkers. Columns 4 and 5 show the bias and the $\sqrt{MSE}$ for the point estimate of the PITE. The sixth column shows the median width of the confidence intervals for the PITE, and the last columns show the proportion of subjects in the identified subgroup when considering the using the limits of the confidence intervals and the point estimates. Since methods reduced and reduced-Scheffe have the same point estimate, bias and MSE are equal.

Case	N	Method	Bias	$\sqrt{MSE}$	Width	% in $\hat{B}_l$	% in $\hat{B}$	% in $\hat{B}_u$	% in B
6	40	ATE	-0.02	0.78	2.00	15.00	84.8	99.90	74.7
6	40	Lasso	-0.37	0.73	18.82	4.30	64.5	96.80	74.7
6	40	reduced	-0.17	1.11	2.13	27.90	61.7	88.20	74.7
6	40	rLasso-1	-0.18	1.09	5.36	5.00	63.1	97.40	74.7
6	40	rLasso-2	-0.30	1.13	3.78	5.20	58.9	96.80	74.7
6	100	ATE	0.00	0.66	0.93	52.80	98.2	100.00	75.1
6	100	full	0.04	1.21	4.72	8.50	65.5	98.40	75.1
6	100	Lasso	-0.28	0.58	8.22	8.40	69.4	98.00	75.1
6	100	reduced	-0.05	0.81	1.64	35.90	68.6	90.70	75.1
6	100	reduced-Scheffe	-0.05	0.81	7.19	0.00	68.6	100.00	75.1
6	100	rLasso-1	-0.09	0.80	3.37	11.01	67.9	97.80	75.1
6	100	rLasso-2	-0.16	0.79	2.48	14.60	67.5	95.80	75.1
6	220	ATE	0.01	0.64	0.59	93.40	100.0	100.00	73.6
6	220	full	0.04	0.65	2.65	18.70	71.6	98.30	73.6
6	220	Lasso	-0.18	0.39	4.93	16.60	73.2	97.10	73.6
6	220	reduced	0.01	0.48	1.17	48.00	73.6	92.10	73.6
6	220	reduced-Scheffe	0.01	0.48	4.73	0.10	73.6	100.00	73.6
6	220	rLasso-1	0.01	0.46	2.11	28.56	74.0	96.39	73.6
6	220	rLasso-2	-0.03	0.47	1.69	31.20	73.2	96.10	73.6
6	350	ATE	0.03	0.63	0.45	99.50	100.0	100.00	74.9
6	350	full	0.01	0.50	2.02	26.30	70.8	98.80	74.9
6	350	Lasso	-0.13	0.32	3.61	21.50	74.1	96.40	74.9
6	350	reduced	0.00	0.40	0.93	49.10	72.5	91.90	74.9
6	350	reduced-Scheffe	0.00	0.40	3.71	1.60	72.5	100.00	74.9
6	350	rLasso-1	0.01	0.37	1.62	33.20	73.4	97.00	74.9
6	350	rLasso-2	-0.01	0.37	1.35	38.80	73.0	95.70	74.9

10.4 Sensitivity and Specificity

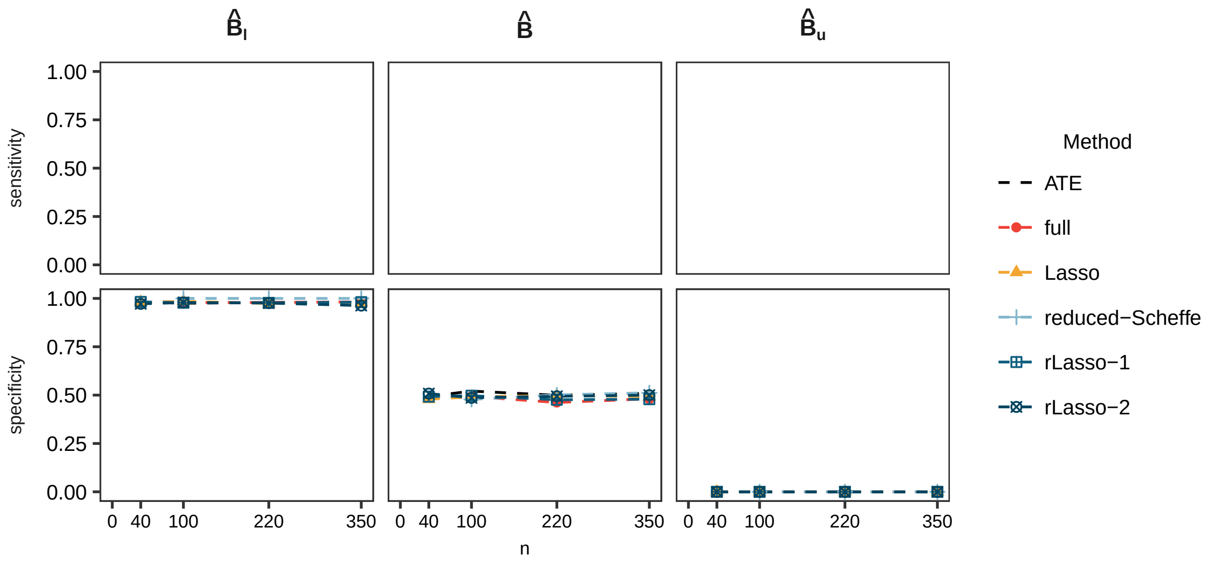

Figure 67: Specificity for identified subgroup by method and sample size for Case 1 and 20 biomarkers. Sensitivity is not shown as there are no patients with $D(X) > 0$

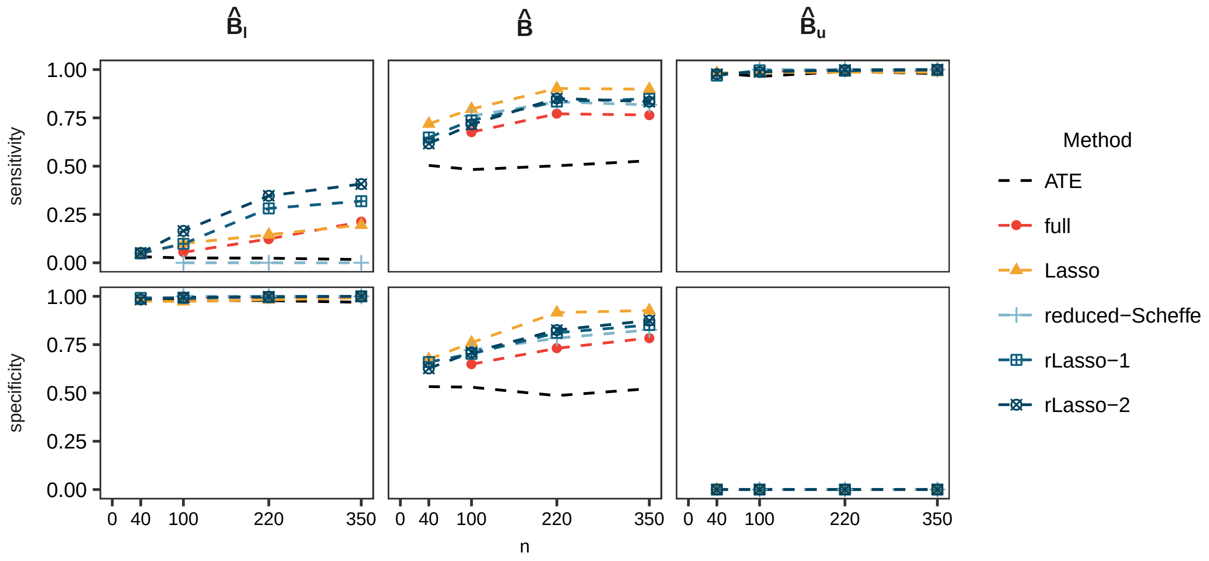

Figure 68: Sensitivity and specificity for identified subgroup by method and sample size for Case 2 and 20 biomarkers.

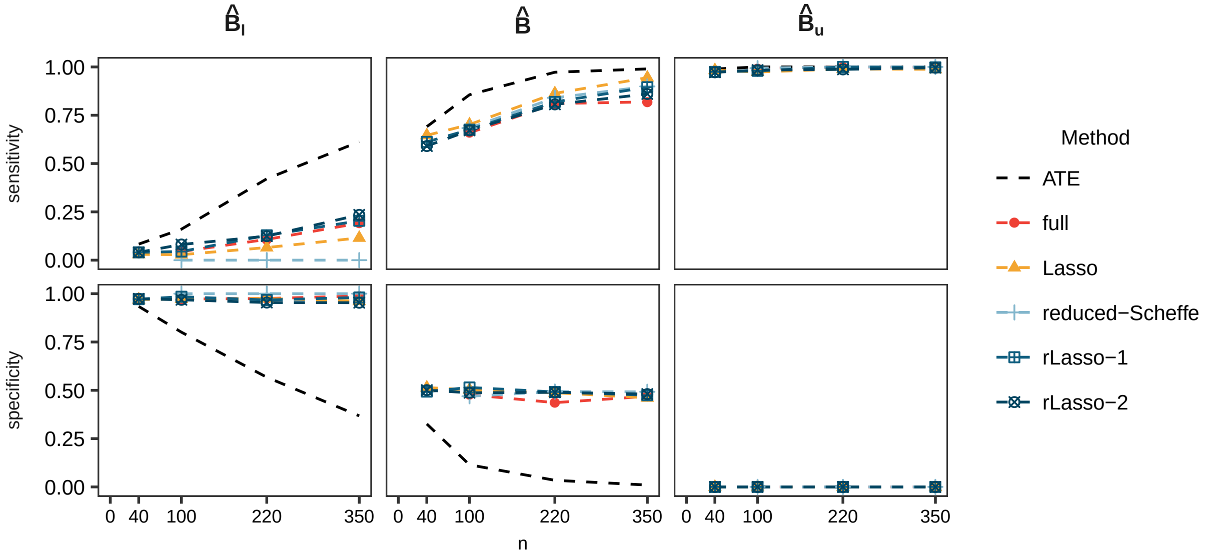

Figure 69: Sensitivity and specificity for identified subgroup by method and sample size for Case 3 and 20 biomarkers.

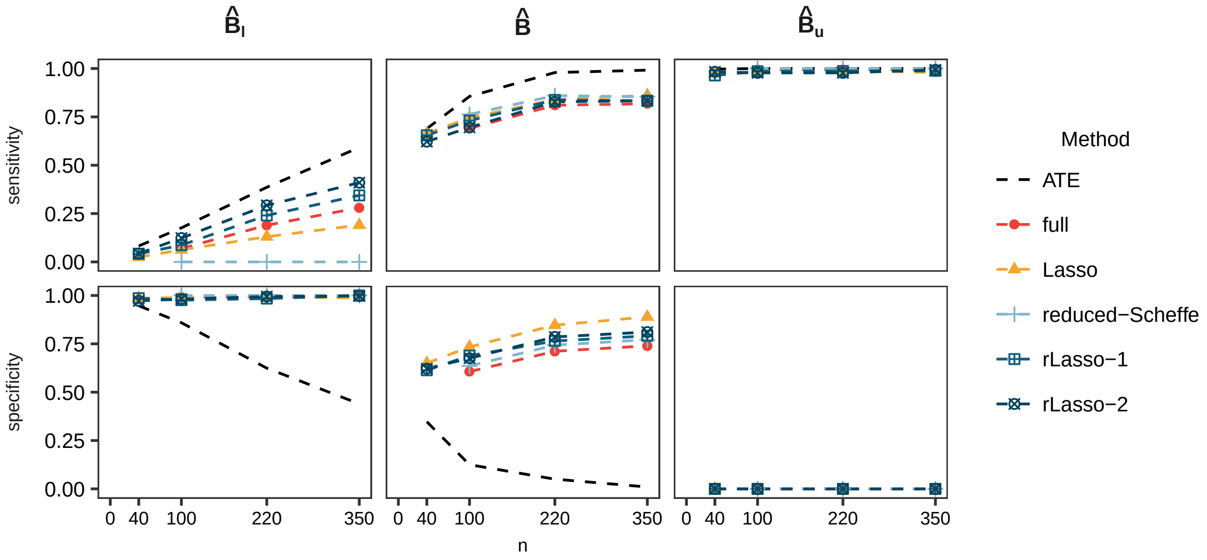

Figure 70: Sensitivity and specificity for identified subgroup by method and sample size for Case 4 and 20 biomarkers.

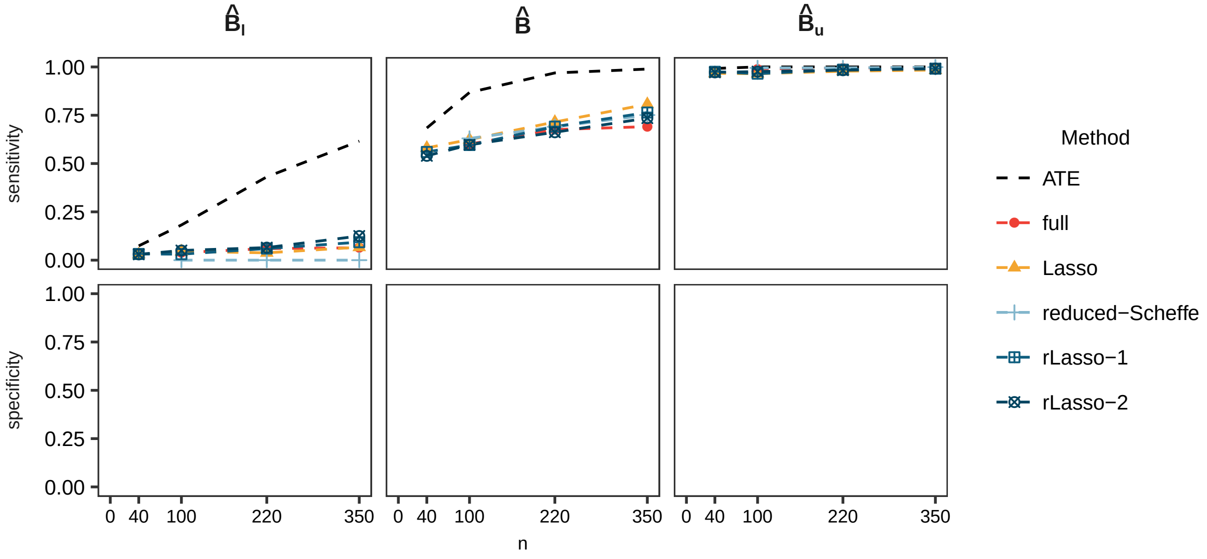

Figure 71: Sensitivity for identified subgroup by method and sample size for Case 5 and 20 biomarkers. Specificity is not shown as all patients have $D(X) > 0$

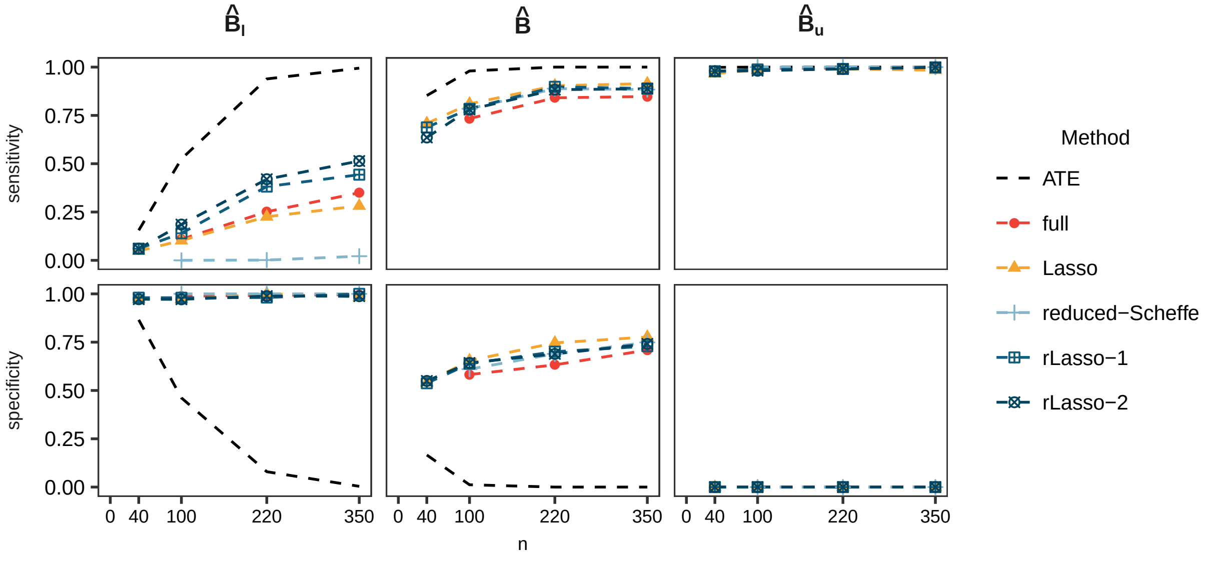

Figure 72: Sensitivity and specificity for identified subgroup by method and sample size for Case 6 and 20 biomarkers.

11 Results for 50 Biomarkers. Default tol.beta parameter in selectiveInference

11.1 Percent of selection

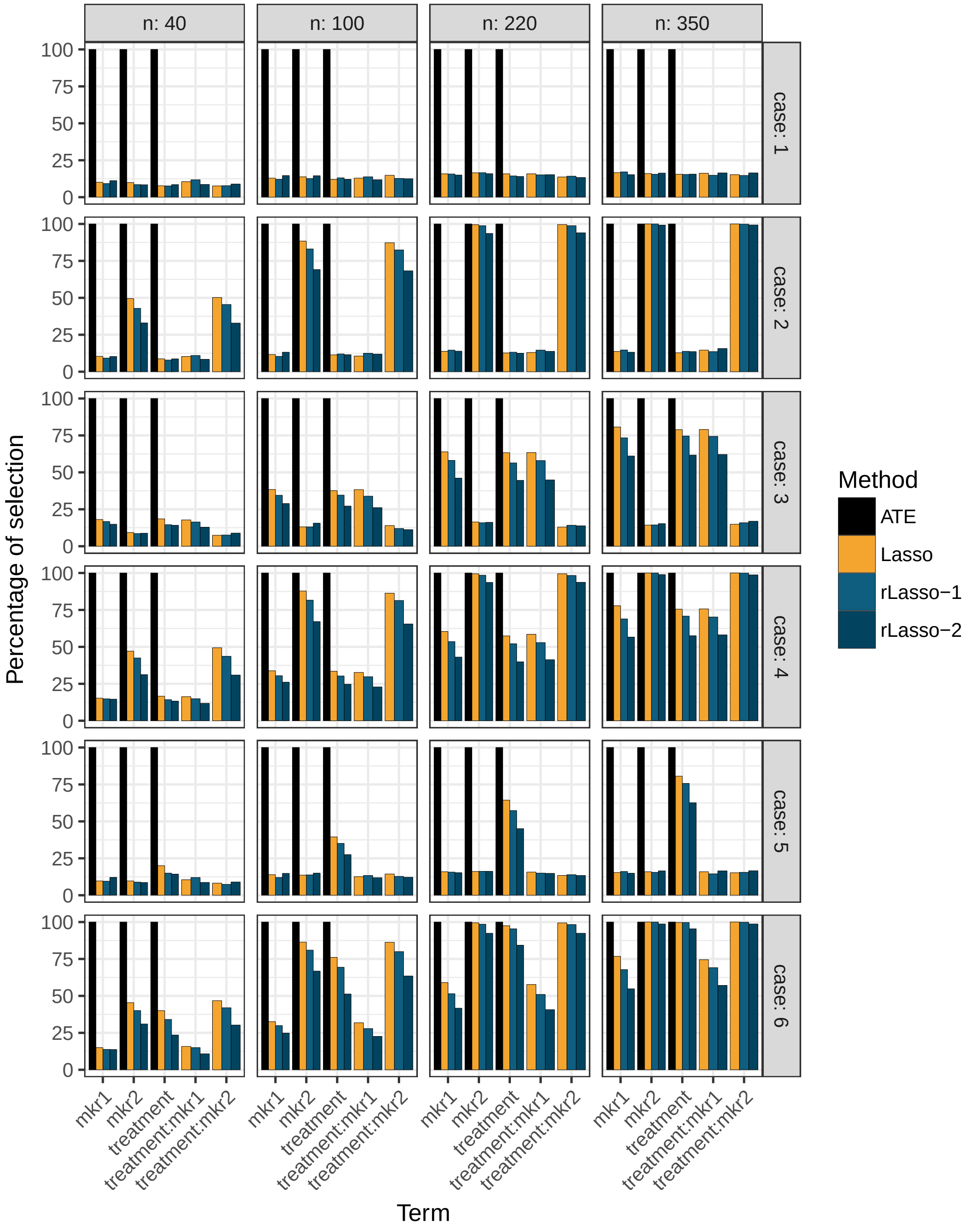

Figure 73: Selection probabilities for relevant terms in the model by method, case and sample size for 50 biomarkers. The full model is not represented here as all variables are included 100% of the times. The reduced model is not represented as it has the same values as the Lasso

11.2 Coverage of confidence intervals

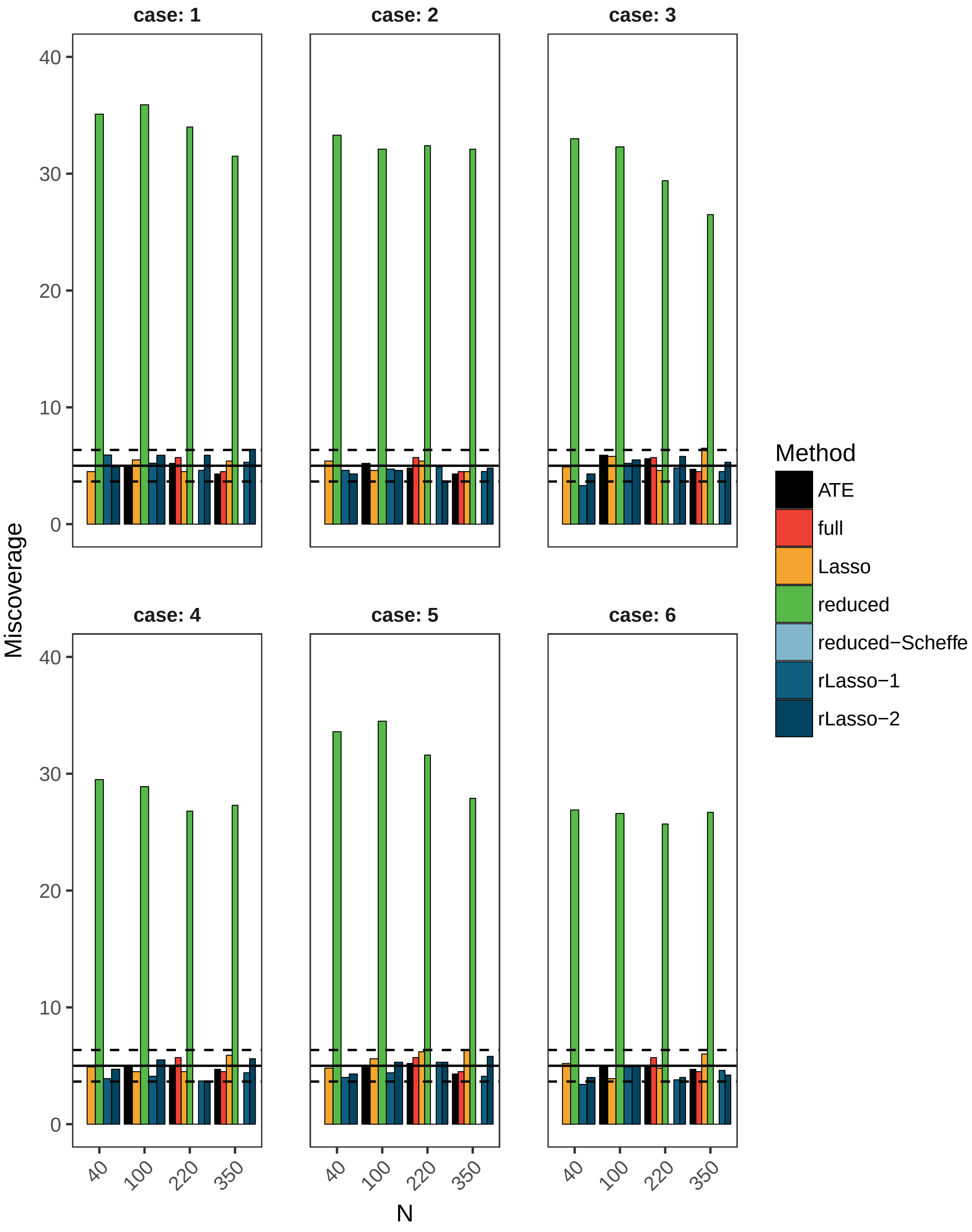

Figure 74: Average miscoverage of the confidence intervals for the PITE by method, case and sample size for 50 biomarkers. The line at 5% indicates the target miscoverage.

11.3 Bias, MSE and width

Table 57: Diagnostic measures for case 1 with 50 biomarkers. Columns 4 and 5 show the bias and the $\sqrt{MSE}$ for the point estimate of the PITE. The sixth column shows the median width of the confidence intervals for the PITE, and the last columns show the proportion of subjects in the identified subgroup when considering the using the limits of the confidence intervals and the point estimates. Since methods reduced and reduced-Scheffe have the same point estimate, bias and MSE are equal.

Case	N	Method	Bias	$\sqrt{MSE}$	Width	% in $\hat{B}_l$	% in $\hat{B}$	% in $\hat{B}_u$	% in B
1	40	ATE							0
1	40	Lasso	-0.01	0.38	29.61	2.10	51.4	97.30	0
1	40	reduced	-0.01	1.06	1.84	17.90	49.7	82.50	0
1	40	rLasso-1	-0.02	1.03	5.92	3.01	50.1	96.89	0
1	40	rLasso-2	0.01	0.96	4.15	2.50	50.6	96.90	0
1	100	ATE	0.00	0.29	1.14	2.20	50.3	97.30	0
1	100	Lasso	0.01	0.32	20.03	3.00	51.0	97.50	0
1	100	reduced	0.01	0.89	1.66	17.60	50.2	81.70	0
1	100	rLasso-1	0.00	0.83	3.93	3.01	48.3	97.79	0
1	100	rLasso-2	-0.02	0.77	2.89	3.10	49.5	97.20	0
1	220	ATE	0.00	0.15	0.60	2.50	50.0	97.30	0
1	220	full	-0.01	1.32	5.18	2.50	50.1	96.80	0
1	220	Lasso	0.01	0.23	14.12	2.40	50.9	97.90	0
1	220	reduced	0.00	0.69	1.30	16.90	51.1	82.90	0
1	220	reduced-Scheffe	0.00	0.69	8.50	0.00	51.1	100.00	0
1	220	rLasso-1	-0.01	0.64	2.72	2.30	49.3	97.60	0
1	220	rLasso-2	0.00	0.57	2.03	2.60	51.4	96.70	0
1	350	ATE	0.00	0.11	0.45	2.40	51.0	98.10	0
1	350	full	0.06	0.91	3.55	2.70	52.4	98.20	0
1	350	Lasso	0.00	0.18	9.38	2.60	51.1	97.20	0
1	350	reduced	0.02	0.52	1.07	16.20	50.3	84.70	0
1	350	reduced-Scheffe	0.02	0.52	6.63	0.00	50.3	100.00	0
1	350	rLasso-1	0.01	0.52	2.15	3.30	52.2	98.00	0
1	350	rLasso-2	-0.01	0.47	1.62	3.01	47.7	96.59	0

Table 58: Diagnostic measures for case 2 with 50 biomarkers. Columns 4 and 5 show the bias and the $\sqrt{MSE}$ for the point estimate of the PITE. The sixth column shows the median width of the confidence intervals for the PITE, and the last columns show the proportion of subjects in the identified subgroup when considering the using the limits of the confidence intervals and the point estimates. Since methods reduced and reduced-Scheffe have the same point estimate, bias and MSE are equal.

Case	N	Method	Bias	$\sqrt{MSE}$	Width	% in $\hat{B}_l$	% in $\hat{B}$	% in $\hat{B}_u$	% in B
2	40	ATE							48.9
2	40	Lasso	0.03	0.60	30.47	2.80	51.1	97.10	48.9
2	40	reduced	0.03	1.10	1.97	21.00	51.7	81.40	48.9
2	40	rLasso-1	0.03	1.13	6.30	2.70	50.6	97.20	48.9
2	40	rLasso-2	0.03	1.04	4.47	2.50	49.3	96.20	48.9
2	100	ATE	0.03	0.62	1.18	2.40	49.7	97.20	47.9
2	100	Lasso	0.02	0.48	19.83	3.50	49.0	97.40	47.9
2	100	reduced	0.00	0.92	1.68	19.60	50.0	78.10	47.9
2	100	rLasso-1	0.01	0.87	4.17	4.71	49.6	95.99	47.9
2	100	rLasso-2	-0.01	0.84	3.15	4.20	48.1	94.10	47.9
2	220	ATE	-0.03	0.60	0.63	2.30	49.0	97.50	54.2
2	220	full	-0.01	1.32	5.18	3.10	52.2	95.60	54.2
2	220	Lasso	0.00	0.32	9.76	6.00	53.9	97.00	54.2
2	220	reduced	-0.01	0.65	1.29	24.50	52.4	78.80	54.2
2	220	reduced-Scheffe	-0.01	0.65	8.33	0.00	52.4	100.00	54.2
2	220	rLasso-1	0.00	0.61	2.81	8.20	52.6	93.30	54.2
2	220	rLasso-2	-0.02	0.57	2.21	10.30	51.2	90.60	54.2
2	350	ATE	0.04	0.59	0.47	1.90	50.7	97.60	47.5
2	350	full	0.06	0.91	3.55	4.70	50.6	94.60	47.5
2	350	Lasso	0.02	0.26	8.07	5.30	47.7	93.30	47.5
2	350	reduced	0.02	0.52	1.04	26.10	49.5	72.60	47.5
2	350	reduced-Scheffe	0.02	0.52	6.44	0.00	49.5	100.00	47.5
2	350	rLasso-1	0.01	0.51	2.23	9.71	49.2	88.59	47.5
2	350	rLasso-2	-0.01	0.45	1.72	12.00	48.6	84.90	47.5

Table 59: Diagnostic measures for case 3 with 50 biomarkers. Columns 4 and 5 show the bias and the $\sqrt{MSE}$ for the point estimate of the PITE. The sixth column shows the median width of the confidence intervals for the PITE, and the last columns show the proportion of subjects in the identified subgroup when considering the using the limits of the confidence intervals and the point estimates. Since methods reduced and reduced-Scheffe have the same point estimate, bias and MSE are equal.

Case	N	Method	Bias	$\sqrt{MSE}$	Width	% in $\hat{B}_l$	% in $\hat{B}$	% in $\hat{B}_u$	% in B
3	40	ATE							51.2
3	40	Lasso	-0.24	0.51	32.66	2.00	52.5	96.6	51.2
3	40	reduced	-0.20	1.11	1.98	19.10	51.2	84.8	51.2
3	40	rLasso-1	-0.21	1.10	6.04	1.80	52.0	98.6	51.2
3	40	rLasso-2	-0.20	1.04	4.27	2.40	54.9	97.1	51.2
3	100	ATE	-0.01	0.38	1.14	13.10	79.9	99.9	51.5
3	100	Lasso	-0.19	0.44	20.68	4.10	57.3	97.9	51.5
3	100	reduced	-0.09	0.94	1.74	21.30	57.3	86.8	51.5
3	100	rLasso-1	-0.12	0.90	4.14	4.61	56.3	97.9	51.5
3	100	rLasso-2	-0.17	0.84	2.99	4.30	54.2	97.9	51.5
3	220	ATE	0.01	0.29	0.61	35.60	95.1	100.0	48.5
3	220	full	-0.01	1.32	5.18	4.20	58.2	98.0	48.5
3	220	Lasso	-0.15	0.34	13.26	3.20	64.2	98.3	48.5
3	220	reduced	-0.05	0.70	1.38	24.70	59.9	89.9	48.5
3	220	reduced-Scheffe	-0.05	0.70	8.93	0.00	59.9	100.0	48.5
3	220	rLasso-1	-0.06	0.65	2.89	5.41	62.3	98.5	48.5
3	220	rLasso-2	-0.09	0.62	2.14	7.90	61.1	97.9	48.5
3	350	ATE	0.01	0.27	0.46	58.50	98.5	100.0	48.8
3	350	full	0.06	0.91	3.55	6.20	62.1	98.8	48.8
3	350	Lasso	-0.13	0.29	10.55	5.40	67.3	97.0	48.8
3	350	reduced	-0.02	0.54	1.14	28.90	63.5	91.9	48.8
3	350	reduced-Scheffe	-0.02	0.54	7.05	0.00	63.5	100.0	48.8
3	350	rLasso-1	-0.02	0.54	2.22	8.70	65.3	97.8	48.8
3	350	rLasso-2	-0.08	0.50	1.73	9.60	60.9	96.7	48.8

Table 60: Diagnostic measures for case 4 with 50 biomarkers. Columns 4 and 5 show the bias and the $\sqrt{MSE}$ for the point estimate of the PITE. The sixth column shows the median width of the confidence intervals for the PITE, and the last columns show the proportion of subjects in the identified subgroup when considering the using the limits of the confidence intervals and the point estimates. Since methods reduced and reduced-Scheffe have the same point estimate, bias and MSE are equal.

Case	N	Method	Bias	$\sqrt{MSE}$	Width	% in $\hat{B}_l$	% in $\hat{B}$	% in $\hat{B}_u$	% in B
4	40	ATE							63.4
4	40	Lasso	-0.22	0.68	36.28	2.70	52.5	97.30	63.4
4	40	reduced	-0.17	1.16	2.07	19.50	51.0	84.80	63.4
4	40	rLasso-1	-0.16	1.16	6.48	3.10	53.3	97.50	63.4
4	40	rLasso-2	-0.21	1.14	4.66	3.00	52.1	97.10	63.4
4	100	ATE	0.02	0.66	1.19	12.70	79.7	99.60	61.9
4	100	Lasso	-0.19	0.57	21.49	2.90	51.6	97.60	61.9
4	100	reduced	-0.11	0.97	1.75	22.70	54.4	83.50	61.9
4	100	rLasso-1	-0.13	0.94	4.34	5.41	53.6	97.00	61.9
4	100	rLasso-2	-0.15	0.91	3.22	5.21	52.7	95.90	61.9
4	220	ATE	-0.02	0.66	0.63	32.40	94.1	100.00	65.0
4	220	full	-0.01	1.32	5.18	5.00	58.6	96.50	65.0
4	220	Lasso	-0.17	0.42	10.67	6.30	60.4	97.70	65.0
4	220	reduced	-0.07	0.67	1.38	31.50	60.1	84.90	65.0
4	220	reduced-Scheffe	-0.07	0.67	8.86	0.00	60.1	100.00	65.0
4	220	rLasso-1	-0.09	0.65	2.98	9.21	60.4	94.49	65.0
4	220	rLasso-2	-0.13	0.62	2.31	13.30	58.4	93.40	65.0
4	350	ATE	0.05	0.65	0.48	55.40	98.2	100.00	60.0
4	350	full	0.06	0.91	3.55	9.10	59.7	96.50	60.0
4	350	Lasso	-0.13	0.34	8.80	8.10	56.8	96.10	60.0
4	350	reduced	-0.02	0.54	1.14	33.80	58.0	80.90	60.0
4	350	reduced-Scheffe	-0.02	0.54	6.95	0.00	58.0	100.00	60.0
4	350	rLasso-1	-0.03	0.53	2.30	14.61	57.5	92.19	60.0
4	350	rLasso-2	-0.09	0.50	1.82	16.52	55.7	89.29	60.0

Table 61: Diagnostic measures for case 5 with 50 biomarkers. Columns 4 and 5 show the bias and the $\sqrt{MSE}$ for the point estimate of the PITE. The sixth column shows the median width of the confidence intervals for the PITE, and the last columns show the proportion of subjects in the identified subgroup when considering the using the limits of the confidence intervals and the point estimates. Since methods reduced and reduced-Scheffe have the same point estimate, bias and MSE are equal.

Case	N	Method	Bias	$\sqrt{MSE}$	Width	% in $\hat{B}_l$	% in $\hat{B}$	% in $\hat{B}_u$	% in B
5	40	ATE							100
5	40	Lasso	-0.23	0.46	32.53	2.30	52.9	96.9	100
5	40	reduced	-0.20	1.11	1.93	19.30	52.8	84.5	100
5	40	rLasso-1	-0.21	1.07	5.94	1.70	52.6	97.3	100
5	40	rLasso-2	-0.17	1.00	4.30	2.20	53.6	97.2	100
5	100	ATE	0.00	0.29	1.14	13.30	80.4	99.9	100
5	100	Lasso	-0.19	0.38	19.65	2.90	58.7	97.0	100
5	100	reduced	-0.09	0.92	1.69	22.50	57.7	86.1	100
5	100	rLasso-1	-0.11	0.84	4.06	3.90	57.8	98.7	100
5	100	rLasso-2	-0.16	0.79	2.94	4.20	54.3	98.4	100
5	220	ATE	0.00	0.15	0.60	35.40	95.3	100.0	100
5	220	full	-0.01	1.32	5.18	3.40	56.4	97.9	100
5	220	Lasso	-0.16	0.29	14.01	4.50	66.4	98.1	100
5	220	reduced	-0.06	0.70	1.34	25.10	61.5	88.9	100
5	220	reduced-Scheffe	-0.06	0.70	8.78	0.00	61.5	100.0	100
5	220	rLasso-1	-0.08	0.65	2.79	5.70	62.1	98.3	100
5	220	rLasso-2	-0.11	0.59	2.07	6.20	62.3	98.1	100
5	350	ATE	0.00	0.11	0.45	59.20	98.7	100.0	100
5	350	full	0.06	0.91	3.55	5.10	62.4	99.2	100
5	350	Lasso	-0.14	0.24	10.05	3.80	72.8	97.6	100
5	350	reduced	-0.02	0.54	1.12	30.50	66.1	93.6	100
5	350	reduced-Scheffe	-0.02	0.54	6.94	0.00	66.1	100.0	100
5	350	rLasso-1	-0.03	0.53	2.19	5.90	67.0	98.9	100
5	350	rLasso-2	-0.08	0.49	1.67	8.91	62.6	98.2	100

Table 62: Diagnostic measures for case 6 with 50 biomarkers. Columns 4 and 5 show the bias and the $\sqrt{MSE}$ for the point estimate of the PITE. The sixth column shows the median width of the confidence intervals for the PITE, and the last columns show the proportion of subjects in the identified subgroup when considering the using the limits of the confidence intervals and the point estimates. Since methods reduced and reduced-Scheffe have the same point estimate, bias and MSE are equal.

Case	N	Method	Bias	$\sqrt{MSE}$	Width	% in $\hat{B}_l$	% in $\hat{B}$	% in $\hat{B}_u$	% in B
6	40	ATE							75.8
6	40	Lasso	-0.41	0.78	36.74	2.50	60.0	96.70	75.8
6	40	reduced	-0.28	1.23	2.11	23.40	58.7	88.10	75.8
6	40	rLasso-1	-0.27	1.21	6.57	3.81	59.7	98.20	75.8
6	40	rLasso-2	-0.37	1.20	4.67	3.60	55.8	97.70	75.8
6	100	ATE	0.02	0.66	1.19	36.00	95.0	100.00	75.7
6	100	Lasso	-0.31	0.63	21.71	3.70	65.9	98.30	75.7
6	100	reduced	-0.12	0.97	1.80	30.80	63.4	89.40	75.7
6	100	rLasso-1	-0.16	0.96	4.51	9.30	63.1	98.10	75.7
6	100	rLasso-2	-0.21	0.96	3.31	9.10	60.7	96.20	75.7
6	220	ATE	-0.02	0.66	0.63	87.30	99.9	100.00	75.1
6	220	full	-0.01	1.32	5.18	7.30	65.7	97.60	75.1
6	220	Lasso	-0.22	0.46	10.14	10.30	74.2	98.80	75.1
6	220	reduced	-0.03	0.66	1.38	43.00	71.5	91.70	75.1
6	220	reduced-Scheffe	-0.03	0.66	8.84	0.00	71.5	100.00	75.1
6	220	rLasso-1	-0.05	0.64	2.98	18.20	71.3	98.30	75.1
6	220	rLasso-2	-0.09	0.64	2.36	23.02	70.6	97.50	75.1
6	350	ATE	0.05	0.65	0.48	98.60	100.0	100.00	72.9
6	350	full	0.06	0.91	3.55	12.70	68.1	98.30	72.9
6	350	Lasso	-0.15	0.36	7.65	12.40	71.6	97.20	72.9
6	350	reduced	0.00	0.52	1.13	44.90	69.6	89.10	72.9
6	350	reduced-Scheffe	0.00	0.52	6.90	0.00	69.6	100.00	72.9
6	350	rLasso-1	0.00	0.51	2.33	25.03	70.3	97.60	72.9
6	350	rLasso-2	-0.03	0.49	1.89	29.96	69.3	96.49	72.9

11.4 Sensitivity and Specificity

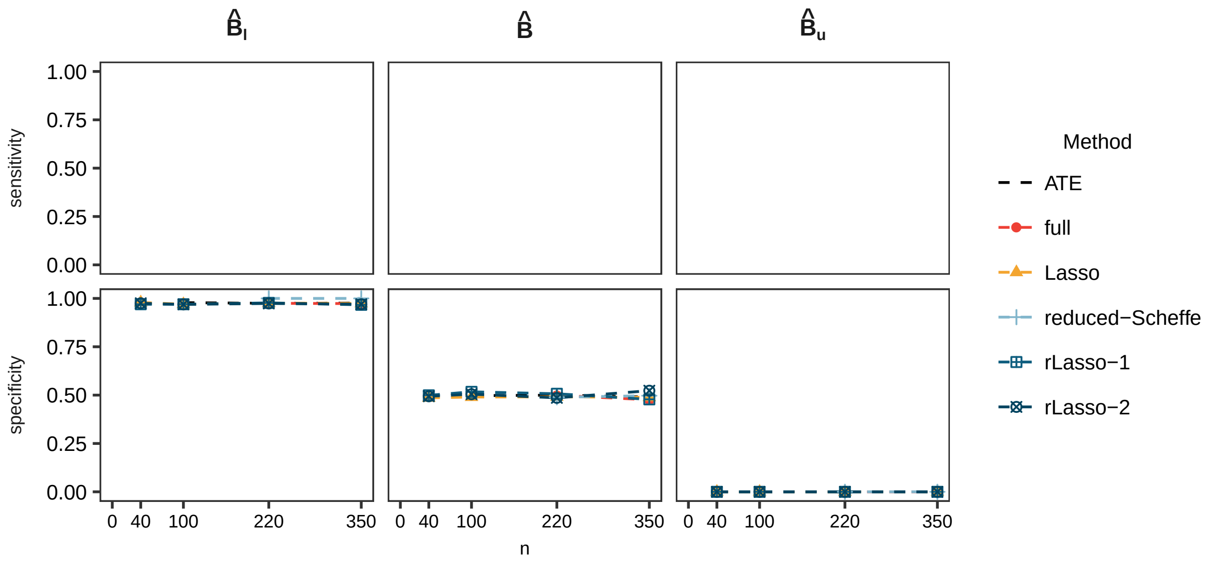

Figure 75: Specificity for identified subgroup by method and sample size for Case 1 and 50 biomarkers. Sensitivity is not shown as there are no patients with $D(X) > 0$

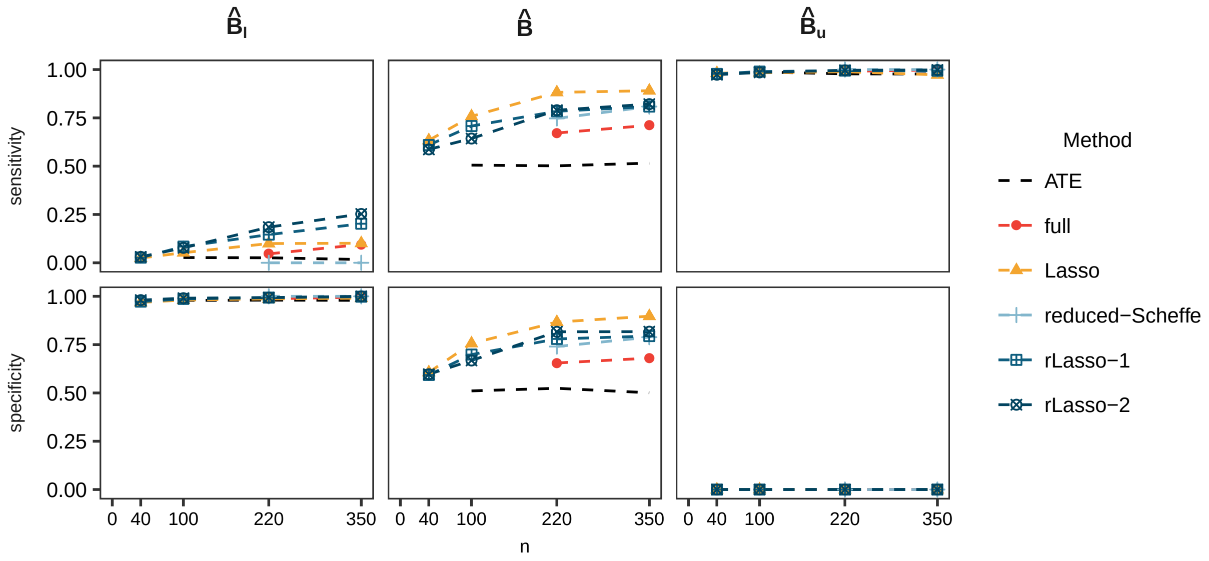

Figure 76: Sensitivity and specificity for identified subgroup by method and sample size for Case 2 and 50 biomarkers.

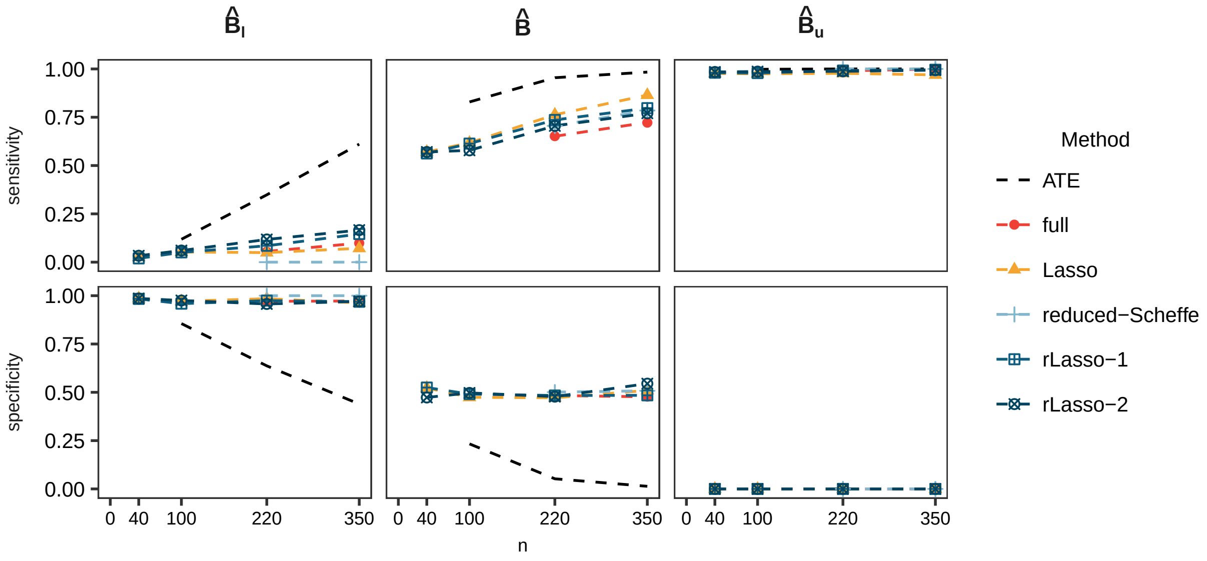

Figure 77: Sensitivity and specificity for identified subgroup by method and sample size for Case 3 and 50 biomarkers.

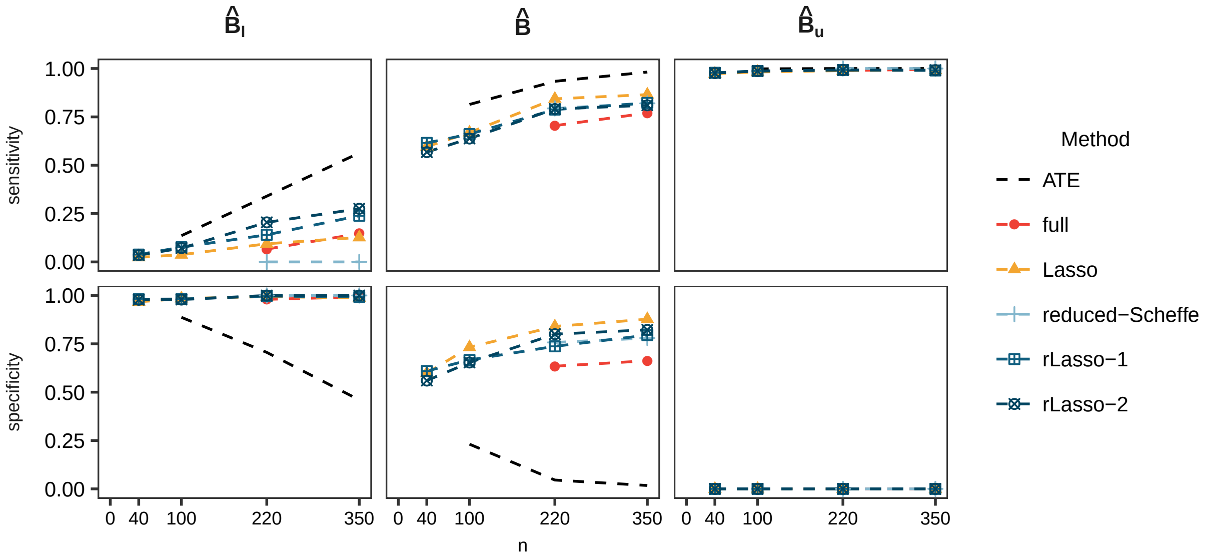

Figure 78: Sensitivity and specificity for identified subgroup by method and sample size for Case 4 and 50 biomarkers.

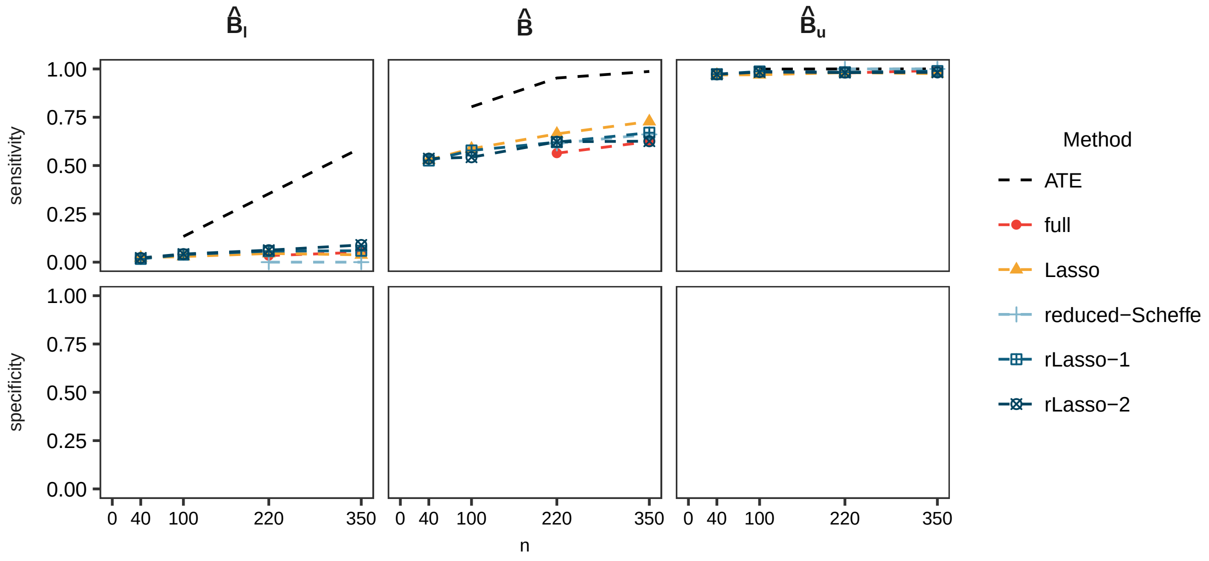

Figure 79: Sensitivity for identified subgroup by method and sample size for Case 5 and 50 biomarkers. Specificity is not shown as all patients have $D(X) > 0$

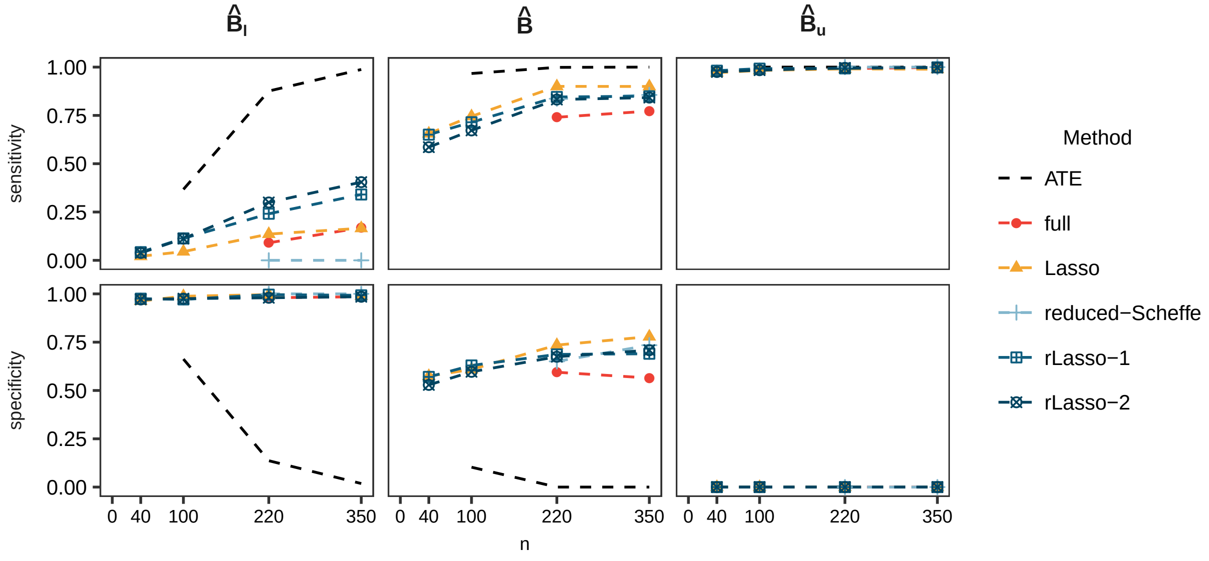

Figure 80: Sensitivity and specificity for identified subgroup by method and sample size for Case 6 and 50 biomarkers.

12 Results for 100 Biomarkers. Default tol.beta parameter in selectiveInference

12.1 Percent of selection

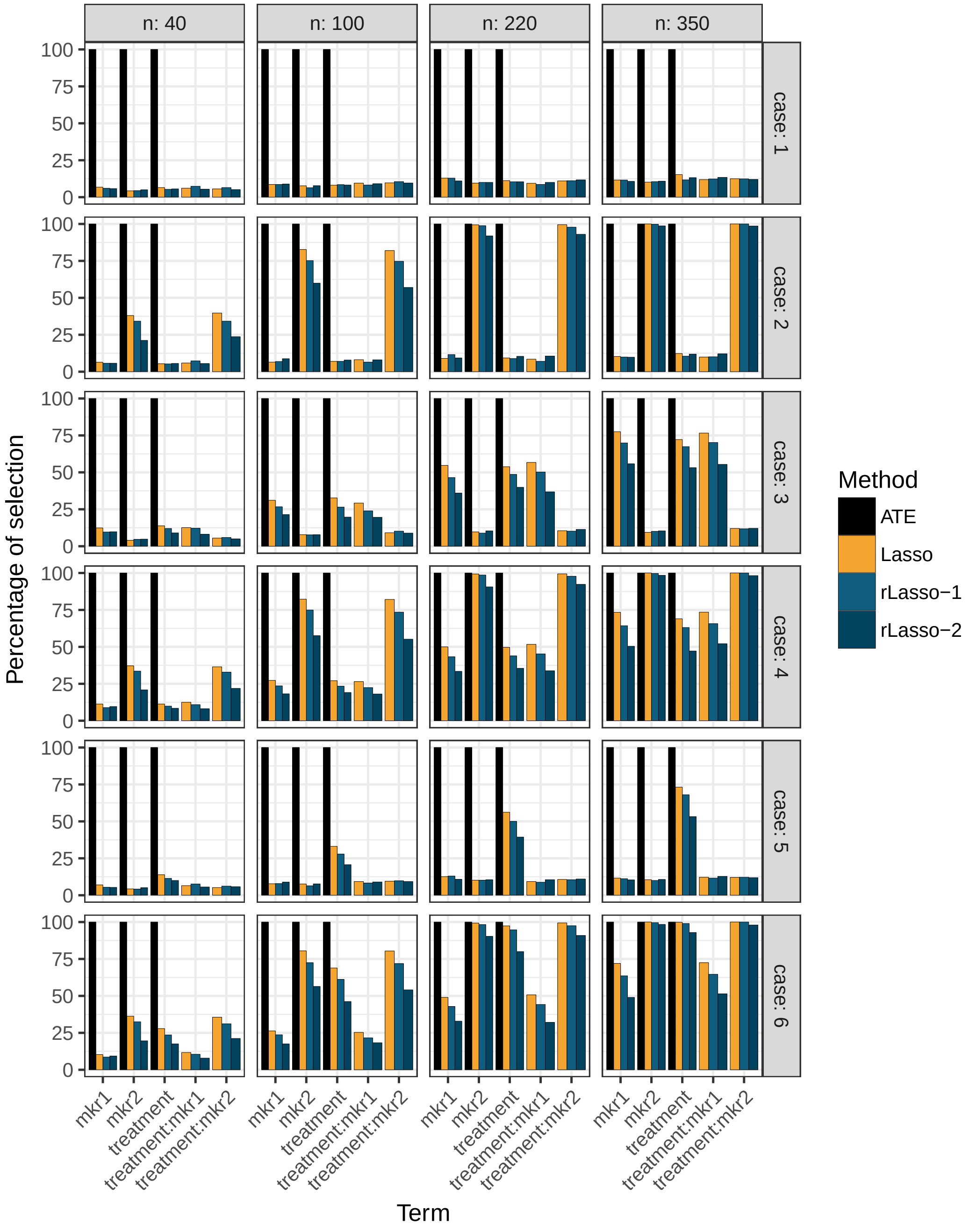

Figure 81: Selection probabilities for relevant terms in the model by method, case and sample size for 100 biomarkers. The full model is not represented here as all variables are included 100% of the times. The reduced model is not represented as it has the same values as the Lasso

12.2 Coverage of confidence intervals

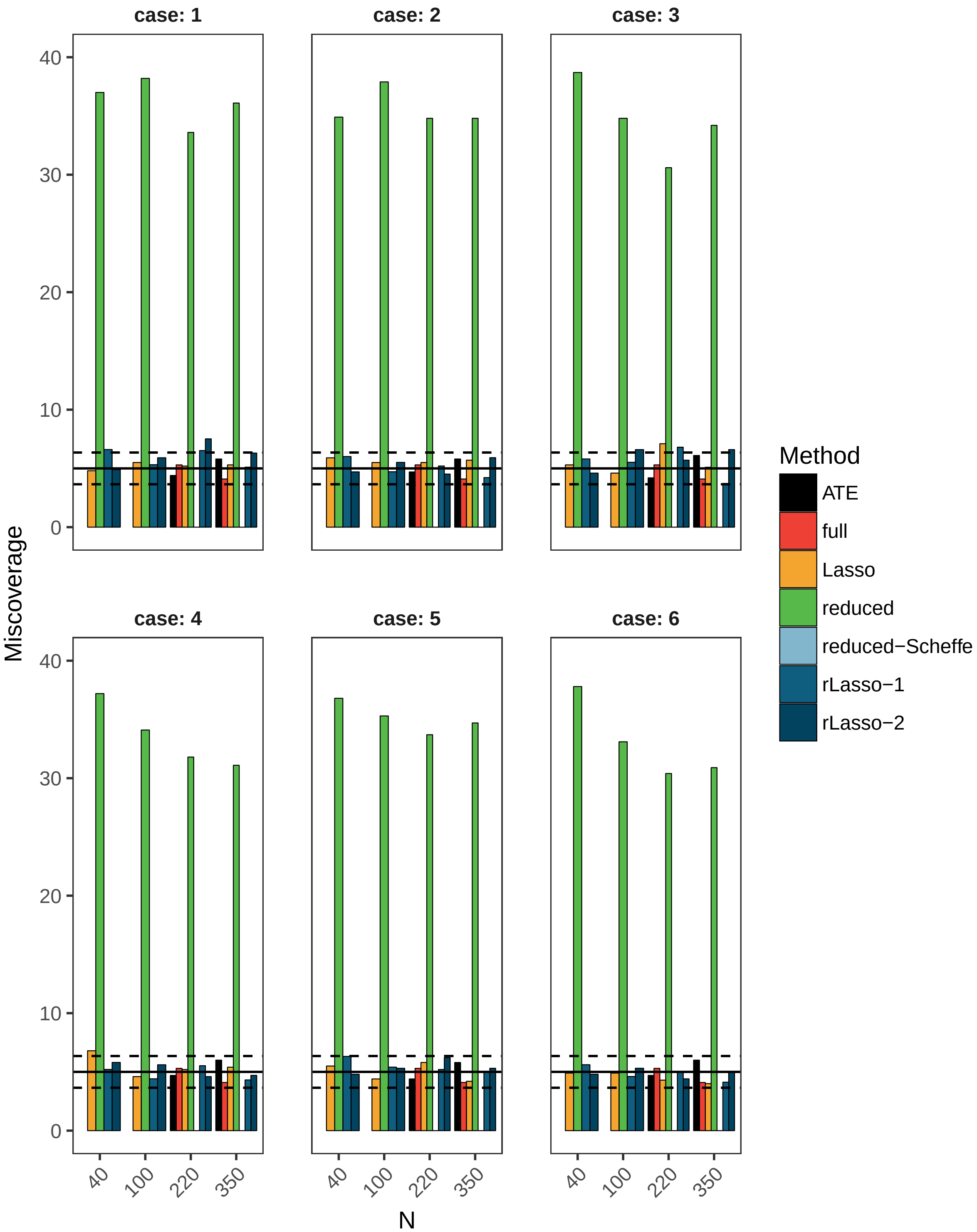

Figure 82: Average miscoverage of the confidence intervals for the PITE by method, case and sample size for 100 biomarkers. The line at 5% indicates the target miscoverage.

12.3 Bias, MSE and width

Table 63: Diagnostic measures for case 1 with 100 biomarkers. Columns 4 and 5 show the bias and the $\sqrt{MSE}$ for the point estimate of the PITE. The sixth column shows the median width of the confidence intervals for the PITE, and the last columns show the proportion of subjects in the identified subgroup when considering the using the limits of the confidence intervals and the point estimates. Since methods reduced and reduced-Scheffe have the same point estimate, bias and MSE are equal.

Case	N	Method	Bias	$\sqrt{MSE}$	Width	% in $\hat{B}_l$	% in $\hat{B}$	% in $\hat{B}_u$	% in B
1	40	ATE							0
1	40	Lasso	0.02	0.40	45.68	2.50	50.3	97.60	0
1	40	reduced	-0.02	1.05	1.81	18.20	48.9	81.10	0
1	40	rLasso-1	0.04	1.05	7.02	3.50	53.1	96.70	0
1	40	rLasso-2	0.01	1.00	4.88	2.40	48.5	97.50	0
1	100	ATE							0
1	100	Lasso	-0.02	0.34	31.75	2.70	46.4	97.10	0
1	100	reduced	-0.05	1.02	1.79	18.50	48.3	80.20	0
1	100	rLasso-1	-0.05	0.95	5.18	2.41	46.4	97.09	0
1	100	rLasso-2	-0.05	0.86	3.61	3.20	45.8	97.30	0
1	220	ATE	0.00	0.18	0.72	2.30	49.1	97.90	0
1	220	full	0.01	5.07	19.58	2.30	52.3	97.00	0
1	220	Lasso	0.02	0.27	21.90	2.80	51.4	97.60	0
1	220	reduced	0.01	0.80	1.51	16.60	51.7	83.00	0
1	220	reduced-Scheffe	0.01	0.80	17.39	0.00	51.7	100.00	0
1	220	rLasso-1	0.02	0.75	3.55	4.31	48.6	97.79	0
1	220	rLasso-2	0.04	0.69	2.65	4.91	51.8	97.39	0
1	350	ATE	-0.01	0.13	0.50	2.80	47.9	97.00	0
1	350	full	-0.05	1.62	6.51	1.80	46.8	97.70	0
1	350	Lasso	0.00	0.21	18.94	2.70	49.1	97.40	0
1	350	reduced	-0.02	0.67	1.32	17.20	48.5	81.10	0
1	350	reduced-Scheffe	-0.02	0.67	11.86	0.00	48.5	100.00	0
1	350	rLasso-1	-0.02	0.64	2.92	3.21	48.5	98.10	0
1	350	rLasso-2	-0.04	0.57	2.11	3.10	46.3	96.80	0

Table 64: Diagnostic measures for case 2 with 100 biomarkers. Columns 4 and 5 show the bias and the $\sqrt{MSE}$ for the point estimate of the PITE. The sixth column shows the median width of the confidence intervals for the PITE, and the last columns show the proportion of subjects in the identified subgroup when considering the using the limits of the confidence intervals and the point estimates. Since methods reduced and reduced-Scheffe have the same point estimate, bias and MSE are equal.

Case	N	Method	Bias	$\sqrt{MSE}$	Width	% in $\hat{B}_l$	% in $\hat{B}$	% in $\hat{B}_u$	% in B
2	40	ATE							53.0
2	40	Lasso	-0.02	0.64	46.72	3.90	52.0	97.90	53.0
2	40	reduced	-0.04	1.19	1.94	18.50	50.9	81.30	53.0
2	40	rLasso-1	0.00	1.16	7.66	2.51	53.0	96.29	53.0
2	40	rLasso-2	-0.05	1.19	5.44	2.60	50.1	97.60	53.0
2	100	ATE							52.3
2	100	Lasso	-0.05	0.53	33.27	2.60	47.3	96.20	52.3
2	100	reduced	-0.09	1.04	1.82	19.50	47.3	78.20	52.3
2	100	rLasso-1	-0.06	1.00	5.40	3.41	51.1	96.09	52.3
2	100	rLasso-2	-0.07	0.95	3.95	4.41	48.4	94.89	52.3
2	220	ATE	0.01	0.61	0.75	2.30	49.1	97.60	50.5
2	220	full	0.01	5.07	19.58	2.40	52.1	97.50	50.5
2	220	Lasso	0.02	0.36	21.22	3.50	51.7	97.20	50.5
2	220	reduced	0.04	0.78	1.47	24.20	51.4	78.00	50.5
2	220	reduced-Scheffe	0.04	0.78	16.34	0.00	51.4	100.00	50.5
2	220	rLasso-1	0.04	0.74	3.67	6.61	52.2	94.59	50.5
2	220	rLasso-2	0.05	0.69	2.81	7.83	51.1	91.67	50.5
2	350	ATE	-0.01	0.59	0.52	2.40	47.9	96.60	50.6
2	350	full	-0.05	1.62	6.51	2.10	48.0	97.30	50.6
2	350	Lasso	0.00	0.29	17.60	4.30	51.2	96.70	50.6
2	350	reduced	-0.02	0.65	1.24	23.50	50.1	75.10	50.6
2	350	reduced-Scheffe	-0.02	0.65	11.18	0.00	50.1	100.00	50.6
2	350	rLasso-1	-0.01	0.59	2.95	6.73	49.5	94.08	50.6
2	350	rLasso-2	-0.02	0.56	2.24	8.32	48.9	87.88	50.6

Table 65: Diagnostic measures for case 3 with 100 biomarkers. Columns 4 and 5 show the bias and the $\sqrt{MSE}$ for the point estimate of the PITE. The sixth column shows the median width of the confidence intervals for the PITE, and the last columns show the proportion of subjects in the identified subgroup when considering the using the limits of the confidence intervals and the point estimates. Since methods reduced and reduced-Scheffe have the same point estimate, bias and MSE are equal.

Case	N	Method	Bias	$\sqrt{MSE}$	Width	% in $\hat{B}_l$	% in $\hat{B}$	% in $\hat{B}_u$	% in B
3	40	ATE							51.4
3	40	Lasso	-0.22	0.54	45.85	3.60	53.0	97.80	51.4
3	40	reduced	-0.21	1.13	1.82	21.20	52.0	81.50	51.4
3	40	rLasso-1	-0.18	1.12	7.17	2.50	53.3	96.30	51.4
3	40	rLasso-2	-0.22	1.03	4.94	2.90	51.6	98.10	51.4
3	100	ATE							52.5
3	100	Lasso	-0.25	0.48	36.92	2.00	52.4	97.40	52.5
3	100	reduced	-0.21	1.07	1.83	20.20	52.0	82.20	52.5
3	100	rLasso-1	-0.25	1.03	5.27	2.41	50.5	96.79	52.5
3	100	rLasso-2	-0.23	0.92	3.77	3.30	51.2	95.80	52.5
3	220	ATE	-0.01	0.31	0.73	25.20	92.0	100.00	50.8
3	220	full	0.01	5.07	19.58	2.60	54.0	97.40	50.8
3	220	Lasso	-0.17	0.38	23.40	4.10	60.2	96.60	50.8
3	220	reduced	-0.08	0.82	1.57	22.50	58.7	88.20	50.8
3	220	reduced-Scheffe	-0.08	0.82	18.00	0.00	58.7	100.00	50.8
3	220	rLasso-1	-0.07	0.80	3.70	6.31	58.7	97.30	50.8
3	220	rLasso-2	-0.10	0.74	2.76	6.10	58.2	97.90	50.8
3	350	ATE	-0.01	0.28	0.50	47.70	96.6	100.00	50.6
3	350	full	-0.05	1.62	6.51	2.80	54.0	97.90	50.6
3	350	Lasso	-0.16	0.33	17.29	2.70	64.0	97.40	50.6
3	350	reduced	-0.07	0.70	1.34	25.70	60.1	87.60	50.6
3	350	reduced-Scheffe	-0.07	0.70	12.14	0.00	60.1	100.00	50.6
3	350	rLasso-1	-0.08	0.67	3.00	4.72	59.4	98.59	50.6
3	350	rLasso-2	-0.12	0.63	2.22	7.20	59.7	97.60	50.6

Table 66: Diagnostic measures for case 4 with 100 biomarkers. Columns 4 and 5 show the bias and the $\sqrt{MSE}$ for the point estimate of the PITE. The sixth column shows the median width of the confidence intervals for the PITE, and the last columns show the proportion of subjects in the identified subgroup when considering the using the limits of the confidence intervals and the point estimates. Since methods reduced and reduced-Scheffe have the same point estimate, bias and MSE are equal.

Case	N	Method	Bias	$\sqrt{MSE}$	Width	% in $\hat{B}_l$	% in $\hat{B}$	% in $\hat{B}_u$	% in B
4	40	ATE							67.4
4	40	Lasso	-0.27	0.75	53.41	3.30	52.8	96.50	67.4
4	40	reduced	-0.25	1.24	1.94	21.10	53.0	80.90	67.4
4	40	rLasso-1	-0.22	1.24	7.79	2.91	53.3	97.60	67.4
4	40	rLasso-2	-0.27	1.23	5.60	3.50	51.4	97.40	67.4
4	100	ATE							65.2
4	100	Lasso	-0.27	0.64	34.48	2.70	51.8	97.80	65.2
4	100	reduced	-0.24	1.08	1.85	21.20	51.7	82.00	65.2
4	100	rLasso-1	-0.26	1.08	5.56	3.31	51.8	96.49	65.2
4	100	rLasso-2	-0.26	1.03	4.01	4.50	52.9	95.50	65.2
4	220	ATE	0.00	0.66	0.76	23.80	90.6	100.00	62.8
4	220	full	0.01	5.07	19.58	2.60	53.6	97.70	62.8
4	220	Lasso	-0.17	0.45	22.75	3.00	56.3	97.30	62.8
4	220	reduced	-0.06	0.80	1.54	28.40	56.8	84.70	62.8
4	220	reduced-Scheffe	-0.06	0.80	17.02	0.00	56.8	100.00	62.8
4	220	rLasso-1	-0.07	0.78	3.82	8.94	56.6	95.28	62.8
4	220	rLasso-2	-0.10	0.75	2.94	9.40	56.5	93.80	62.8
4	350	ATE	-0.01	0.64	0.52	45.70	95.7	100.00	63.8
4	350	full	-0.05	1.62	6.51	3.50	54.4	97.90	63.8
4	350	Lasso	-0.17	0.39	17.14	3.40	57.4	96.70	63.8
4	350	reduced	-0.07	0.66	1.30	31.60	58.8	81.70	63.8
4	350	reduced-Scheffe	-0.07	0.66	11.57	0.00	58.8	100.00	63.8
4	350	rLasso-1	-0.08	0.65	3.03	10.44	56.6	95.88	63.8
4	350	rLasso-2	-0.12	0.63	2.33	12.31	56.0	91.89	63.8

Table 67: Diagnostic measures for case 5 with 100 biomarkers. Columns 4 and 5 show the bias and the $\sqrt{MSE}$ for the point estimate of the PITE. The sixth column shows the median width of the confidence intervals for the PITE, and the last columns show the proportion of subjects in the identified subgroup when considering the using the limits of the confidence intervals and the point estimates. Since methods reduced and reduced-Scheffe have the same point estimate, bias and MSE are equal.

Case	N	Method	Bias	$\sqrt{MSE}$	Width	% in $\hat{B}_l$	% in $\hat{B}$	% in $\hat{B}_u$	% in B
5	40	ATE							100
5	40	Lasso	-0.22	0.47	38.94	2.90	54.1	97.40	100
5	40	reduced	-0.22	1.07	1.81	19.20	51.1	82.20	100
5	40	rLasso-1	-0.18	1.05	7.21	3.21	53.8	96.79	100
5	40	rLasso-2	-0.18	1.02	5.02	2.80	51.9	97.80	100
5	100	ATE							100
5	100	Lasso	-0.23	0.42	32.92	1.60	50.6	97.10	100
5	100	reduced	-0.19	1.05	1.82	21.10	52.8	83.20	100
5	100	rLasso-1	-0.23	0.99	5.22	2.70	50.1	97.10	100
5	100	rLasso-2	-0.24	0.90	3.64	2.70	50.7	97.00	100
5	220	ATE	0.00	0.18	0.72	26.20	92.1	100.00	100
5	220	full	0.01	5.07	19.58	2.50	54.4	97.40	100
5	220	Lasso	-0.17	0.32	23.52	2.50	62.1	97.20	100
5	220	reduced	-0.07	0.82	1.53	23.00	59.6	87.30	100
5	220	reduced-Scheffe	-0.07	0.82	17.60	0.00	59.6	100.00	100
5	220	rLasso-1	-0.08	0.79	3.61	4.30	57.2	98.10	100
5	220	rLasso-2	-0.09	0.72	2.66	6.21	58.3	98.10	100
5	350	ATE	-0.01	0.13	0.50	48.10	96.7	100.00	100
5	350	full	-0.05	1.62	6.51	2.60	53.5	98.10	100
5	350	Lasso	-0.16	0.27	18.42	2.40	65.2	98.30	100
5	350	reduced	-0.07	0.69	1.32	25.00	59.9	90.10	100
5	350	reduced-Scheffe	-0.07	0.69	12.03	0.00	59.9	100.00	100
5	350	rLasso-1	-0.09	0.66	2.96	4.22	60.1	98.59	100
5	350	rLasso-2	-0.13	0.60	2.19	4.40	59.6	98.40	100

Table 68: Diagnostic measures for case 6 with 100 biomarkers. Columns 4 and 5 show the bias and the $\sqrt{MSE}$ for the point estimate of the PITE. The sixth column shows the median width of the confidence intervals for the PITE, and the last columns show the proportion of subjects in the identified subgroup when considering the using the limits of the confidence intervals and the point estimates. Since methods reduced and reduced-Scheffe have the same point estimate, bias and MSE are equal.

Case	N	Method	Bias	$\sqrt{MSE}$	Width	% in $\hat{B}_l$	% in $\hat{B}$	% in $\hat{B}_u$	% in B
6	40	ATE							79.1
6	40	Lasso	-0.47	0.86	52.86	2.20	57.8	97.20	79.1
6	40	reduced	-0.41	1.31	1.99	23.60	56.6	82.80	79.1
6	40	rLasso-1	-0.37	1.33	8.01	3.41	57.3	96.59	79.1
6	40	rLasso-2	-0.47	1.32	5.57	3.20	54.2	97.60	79.1
6	100	ATE							77.1
6	100	Lasso	-0.41	0.72	33.66	2.90	63.3	97.70	77.1
6	100	reduced	-0.29	1.11	1.88	27.20	59.0	87.40	77.1
6	100	rLasso-1	-0.30	1.12	5.71	6.01	58.8	97.80	77.1
6	100	rLasso-2	-0.35	1.08	4.18	7.82	57.8	97.09	77.1
6	220	ATE	0.00	0.66	0.76	72.50	99.7	100.00	75.4
6	220	full	0.01	5.07	19.58	2.90	55.6	97.80	75.4
6	220	Lasso	-0.23	0.48	23.05	5.20	71.9	98.80	75.4
6	220	reduced	-0.03	0.78	1.54	38.20	68.2	90.50	75.4
6	220	reduced-Scheffe	-0.03	0.78	17.07	0.00	68.2	100.00	75.4
6	220	rLasso-1	-0.02	0.77	3.88	13.84	69.1	98.19	75.4
6	220	rLasso-2	-0.08	0.76	2.97	15.93	67.2	96.89	75.4
6	350	ATE	-0.01	0.64	0.52	95.20	100.0	100.00	75.7
6	350	full	-0.05	1.62	6.51	4.70	59.6	98.20	75.7
6	350	Lasso	-0.20	0.41	16.15	6.10	74.1	98.20	75.7
6	350	reduced	-0.04	0.65	1.29	43.50	69.6	91.10	75.7
6	350	reduced-Scheffe	-0.04	0.65	11.56	0.00	69.6	100.00	75.7
6	350	rLasso-1	-0.05	0.63	3.02	16.70	70.7	98.89	75.7
6	350	rLasso-2	-0.07	0.61	2.34	21.64	70.9	97.19	75.7

12.4 Sensitivity and Specificity

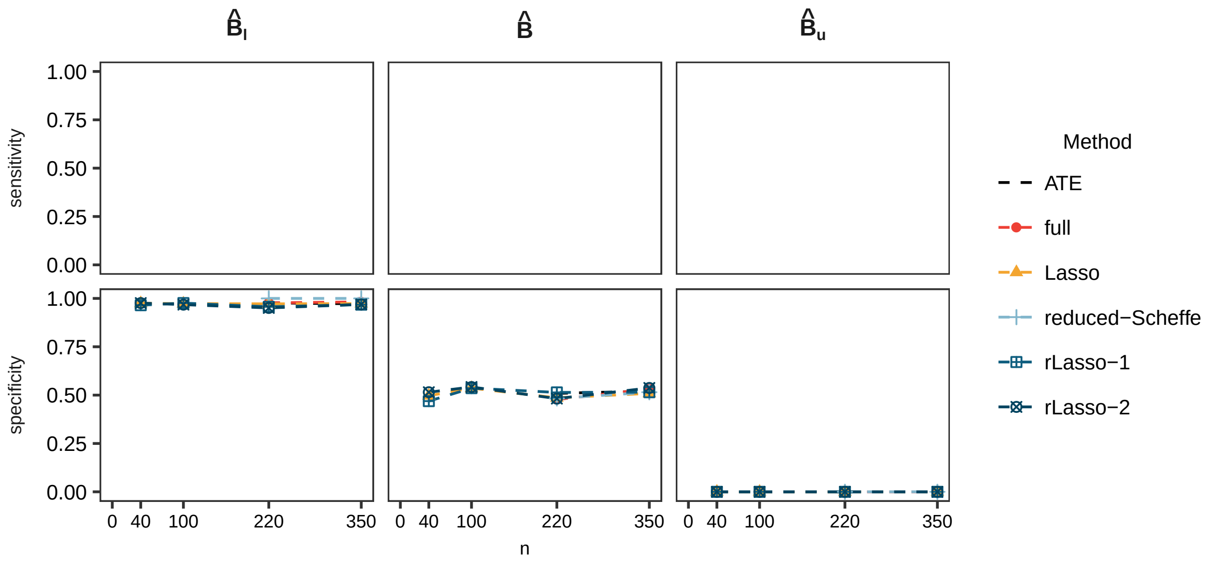

Figure 83: Specificity for identified subgroup by method and sample size for Case 1 and 100 biomarkers. Sensitivity is not shown as there are no patients with $D(X) > 0$

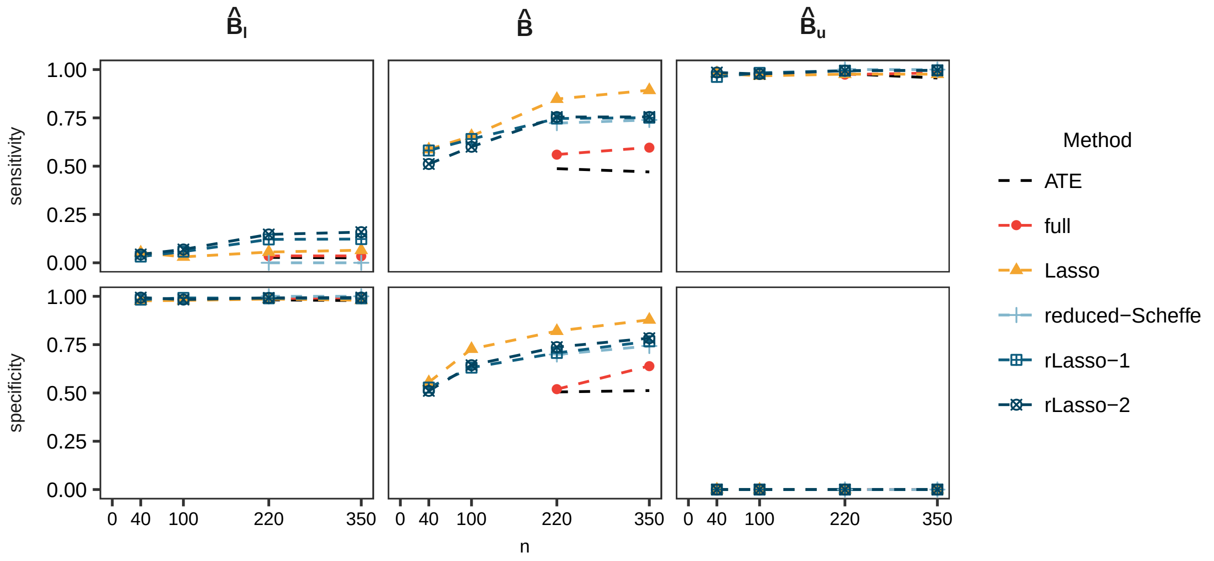

Figure 84: Sensitivity and specificity for identified subgroup by method and sample size for Case 2 and 100 biomarkers.

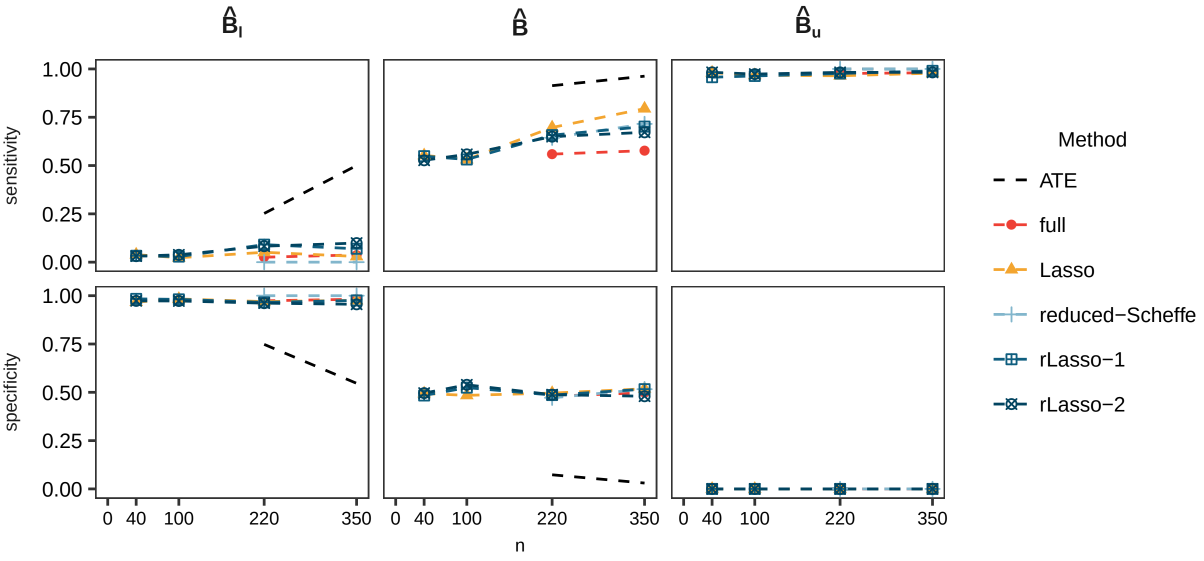

Figure 85: Sensitivity and specificity for identified subgroup by method and sample size for Case 3 and 100 biomarkers.

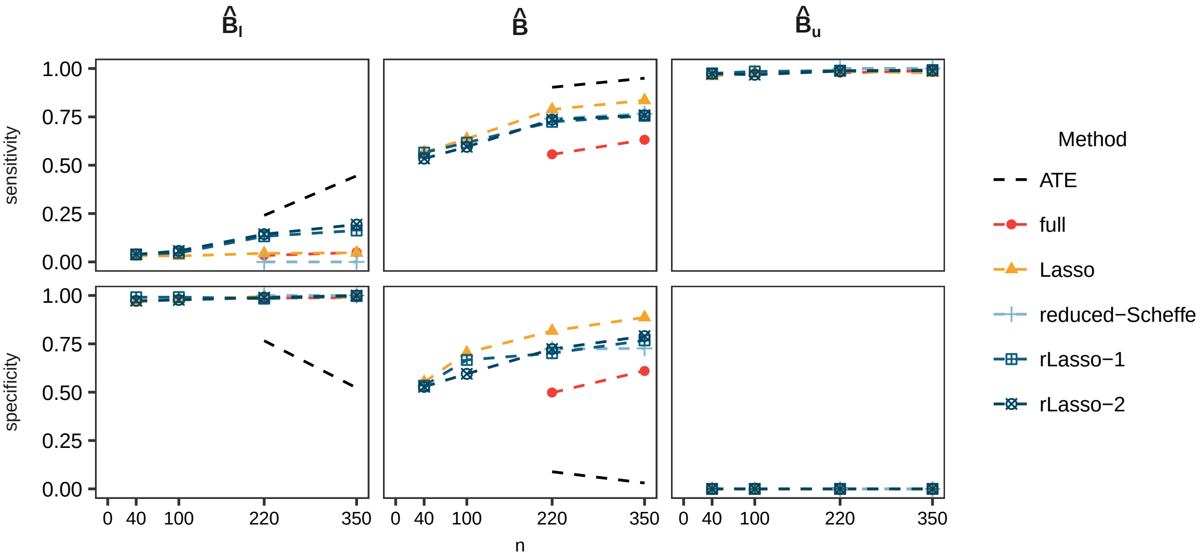

Figure 86: Sensitivity and specificity for identified subgroup by method and sample size for Case 4 and 100 biomarkers.

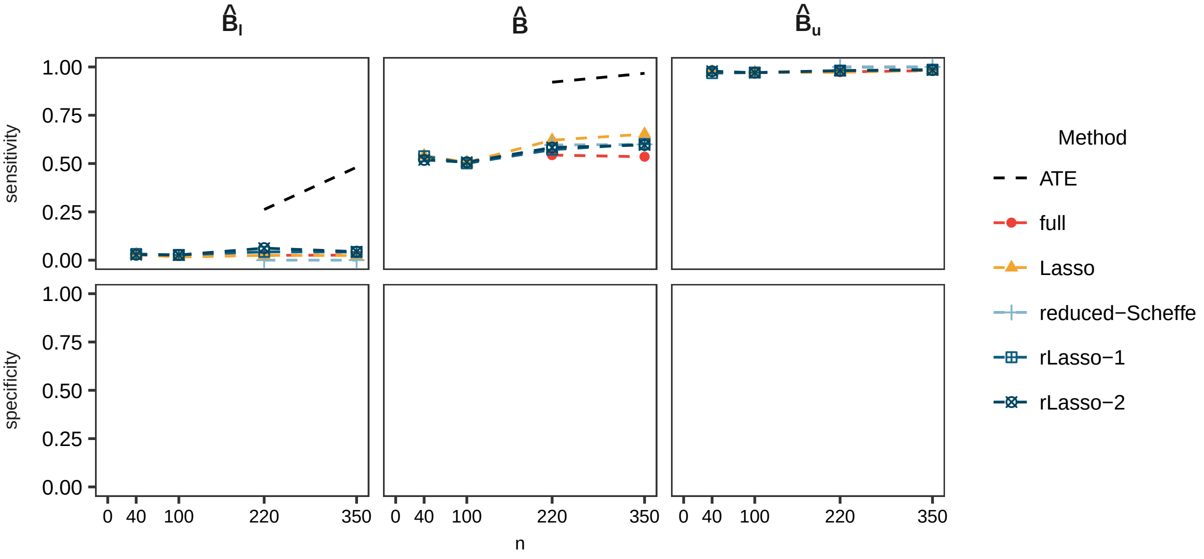

Figure 87: Sensitivity for identified subgroup by method and sample size for Case 5 and 100 biomarkers. Specificity is not shown as all patients have $D(X) > 0$

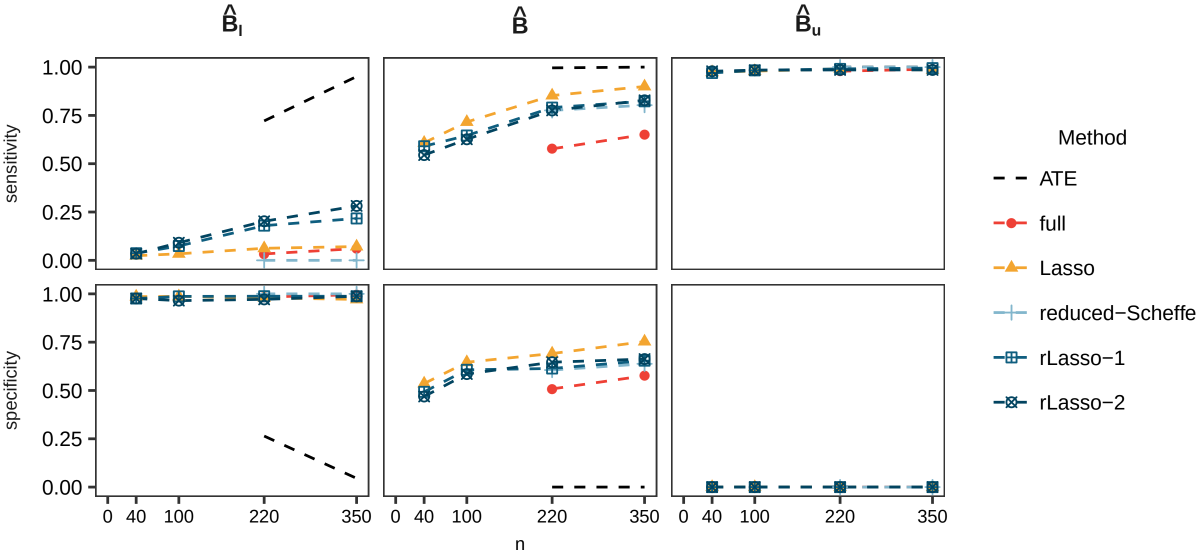

Figure 88: Sensitivity and specificity for identified subgroup by method and sample size for Case 6 and 100 biomarkers.
