# Supplementary figures and images for: Subgroup identification in clinical trials via the predicted individual treatment effect
Source: PLoS One. 2018 Oct 18;13(10):e0205971. doi: 10.1371/journal.pone.0205971 (PMC6193713; doi:10.1371/journal.pone.0205971)

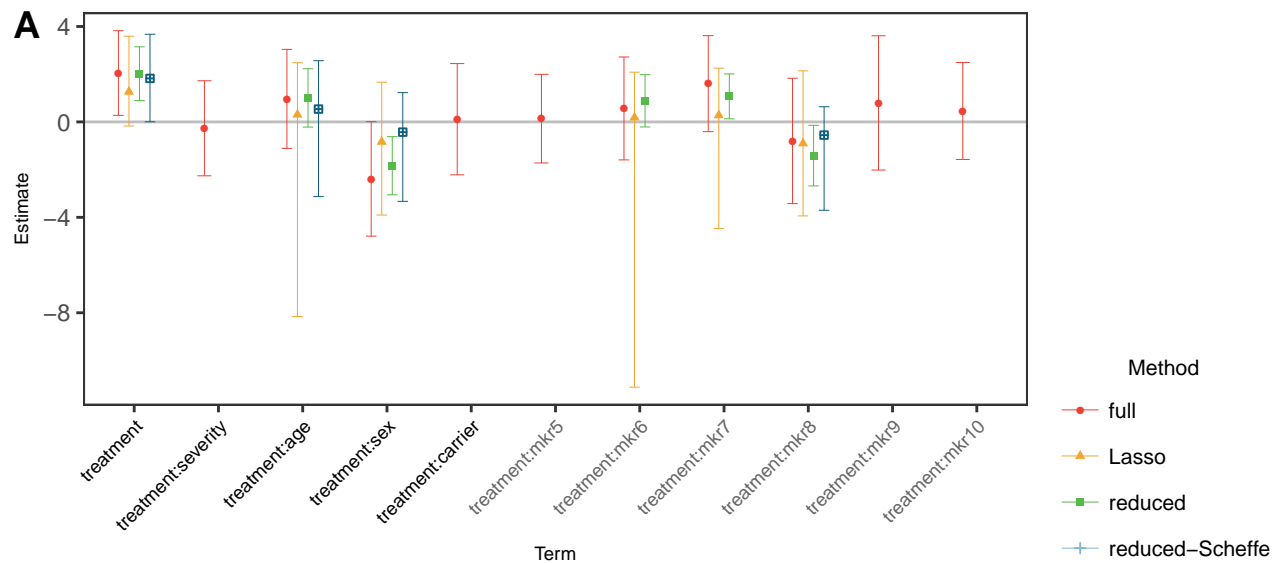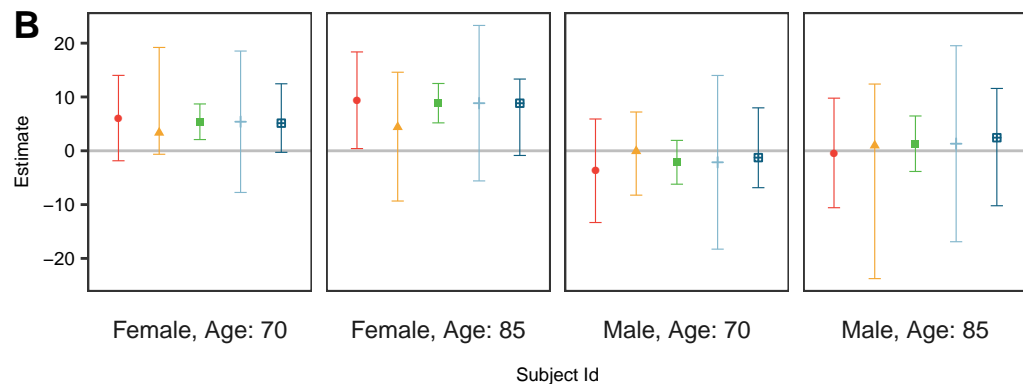

Supplement: S2 File — Code to reproduce the results of this article. (ZIP) [file pone.0205971.s002.zip › PLOS-2018-PITE-master/paper/figures/Fig1.pdf]

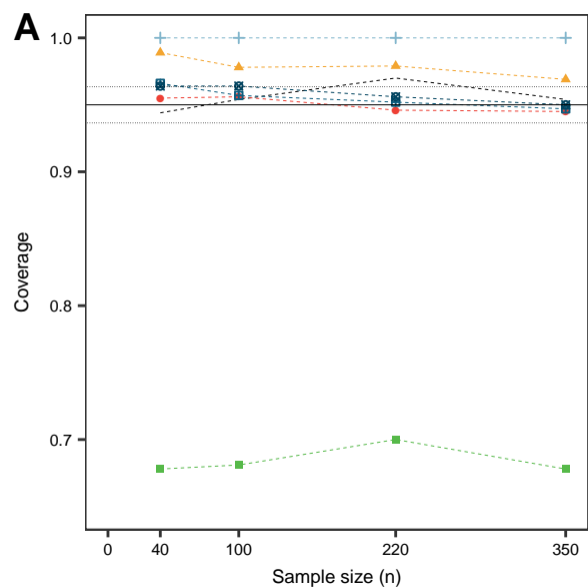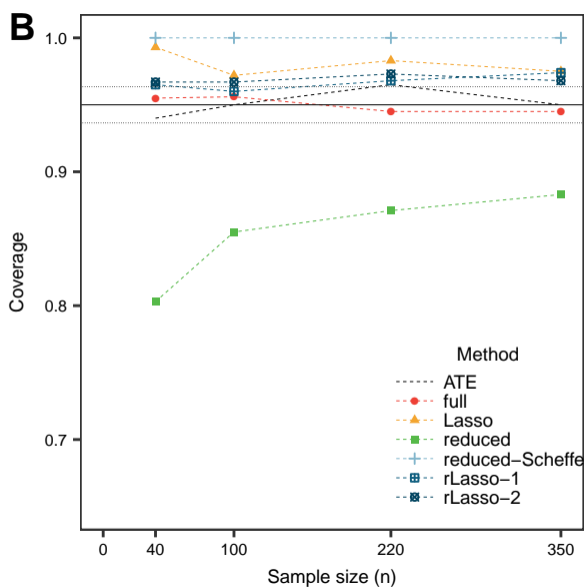

Supplement: S2 File — Code to reproduce the results of this article. (ZIP) [file pone.0205971.s002.zip › PLOS-2018-PITE-master/paper/figures/Fig2.pdf]

**A**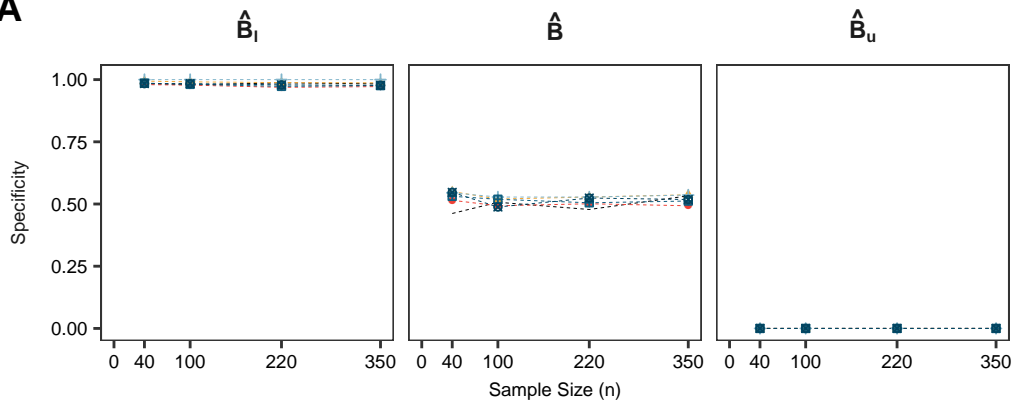**B**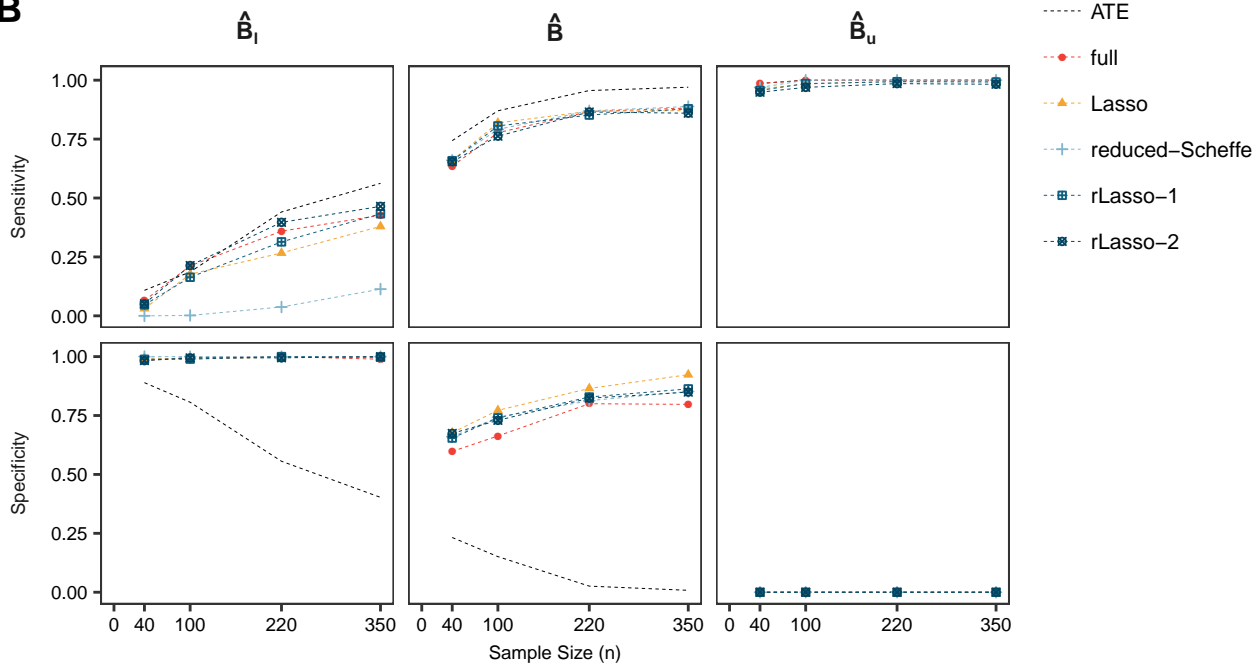

Supplement: S2 File — Code to reproduce the results of this article. (ZIP) [file pone.0205971.s002.zip › PLOS-2018-PITE-master/paper/figures/Fig3.pdf]

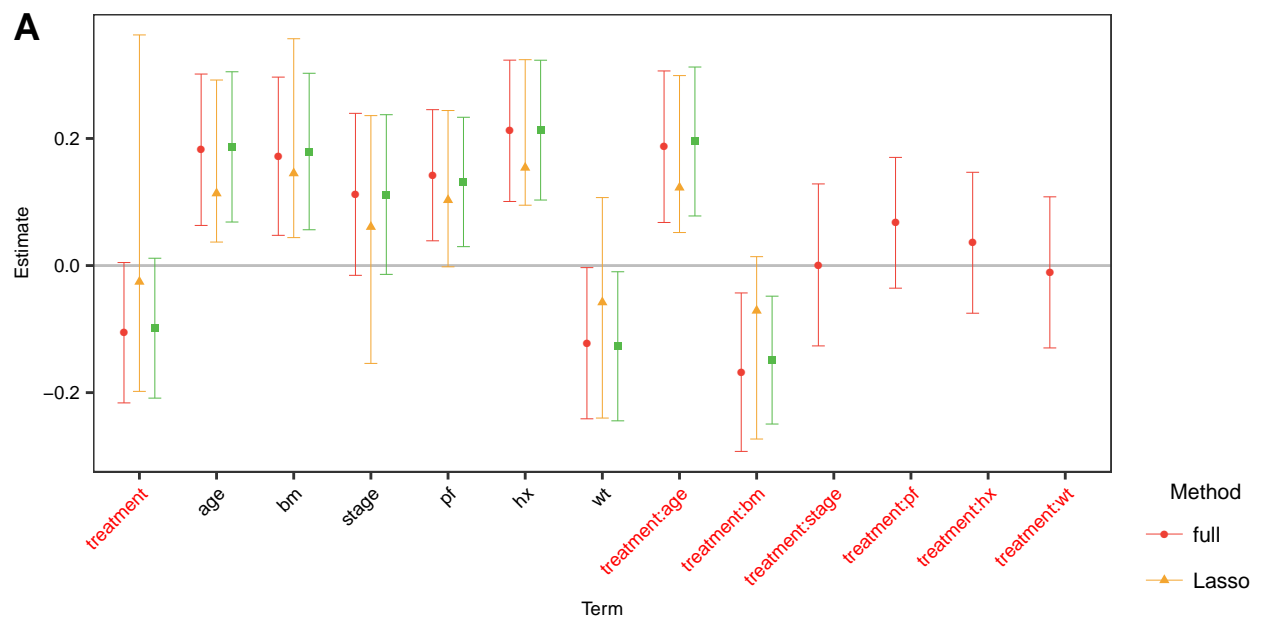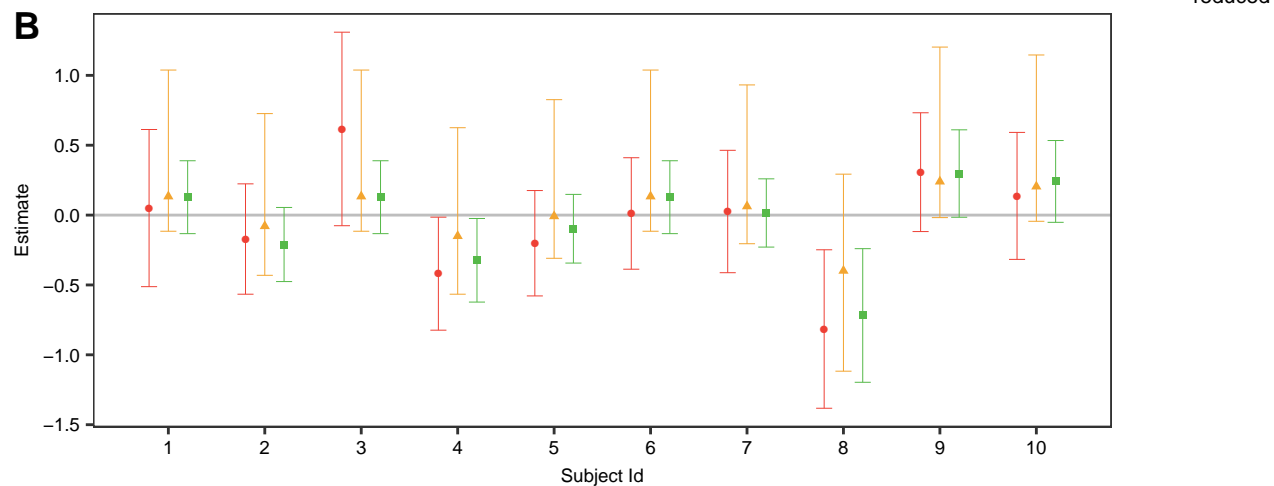

Supplement: S2 File — Code to reproduce the results of this article. (ZIP) [file pone.0205971.s002.zip › PLOS-2018-PITE-master/paper/figures/Fig4.pdf]

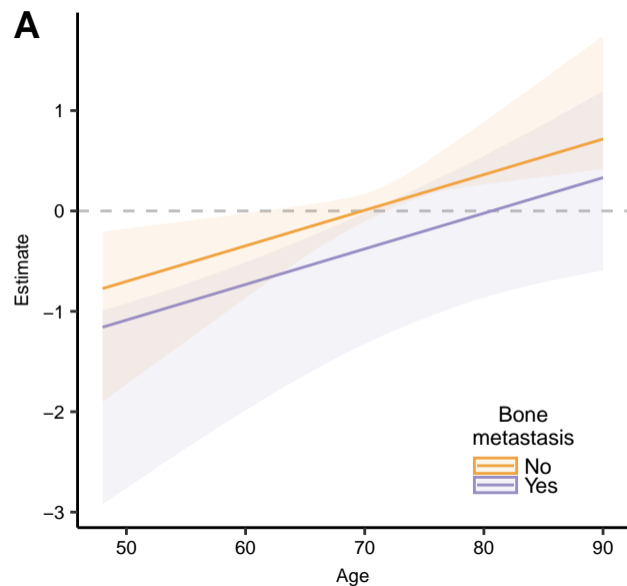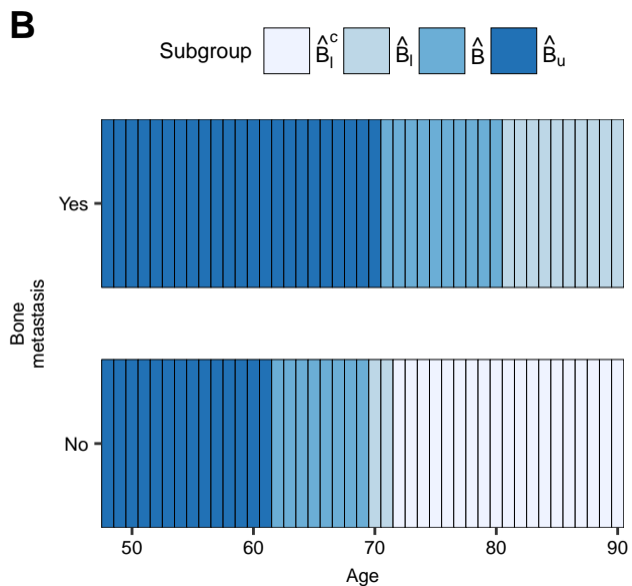

Supplement: S2 File — Code to reproduce the results of this article. (ZIP) [file pone.0205971.s002.zip › PLOS-2018-PITE-master/paper/figures/Fig5.pdf]

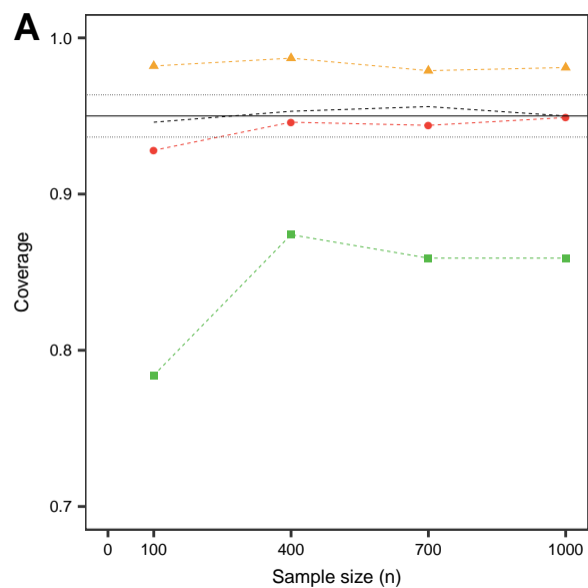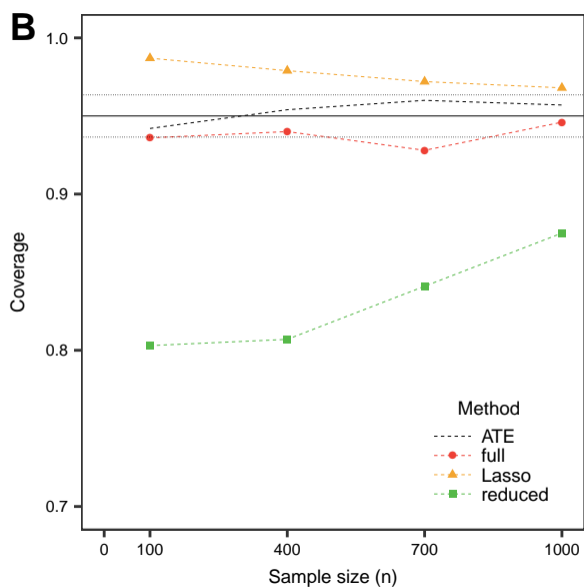

Supplement: S2 File — Code to reproduce the results of this article. (ZIP) [file pone.0205971.s002.zip › PLOS-2018-PITE-master/paper/figures/Fig6.pdf]

**A**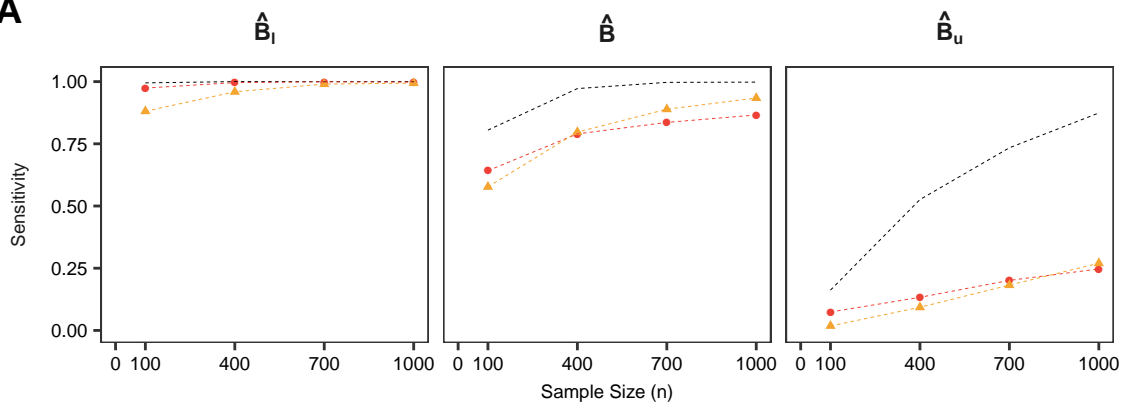**B**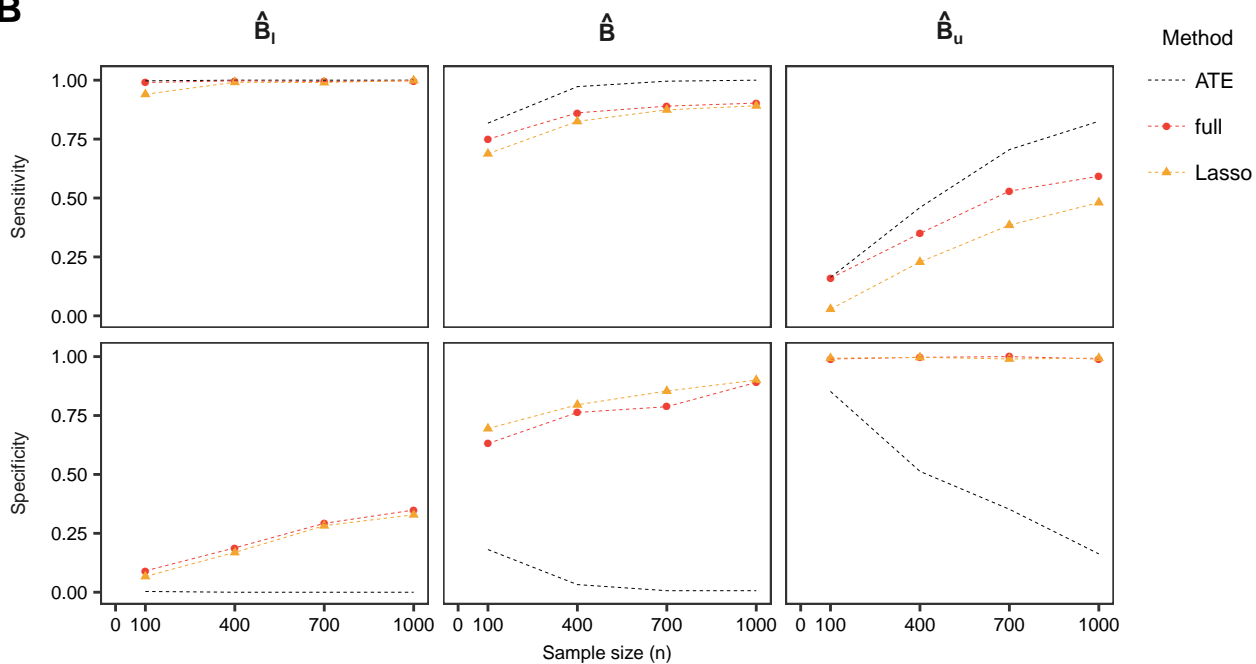

Supplement: S2 File — Code to reproduce the results of this article. (ZIP) [file pone.0205971.s002.zip › PLOS-2018-PITE-master/paper/figures/Fig7.pdf]
